# Diastereoselective synthesis of substituted cyclopentanones *via* [3 + 2] cycloaddition of sulfoxonium ylides with alkenes

**DOI:** 10.1039/d6sc05806j

**Published:** 2026-09-21

**Authors:** Jihyeon Yeo, Sebastian M. Krajewski, Jonathan A. Ellman

**Affiliations:** a Department of Chemistry, Yale University 226 Prospect St. New Haven CT 06520 USA jonathan.ellman@yale.edu

## Abstract

We report a straightforward one-step, highly diastereoselective [3 + 2] cycloaddition for the synthesis of cyclopentanones incorporating up to four stereogenic centres. Sulfoxonium ylides are readily available, safe-to-use reagents and were utilised as three-carbon synthons to introduce alkyl, aryl, and heteroatom substituents at the α-positions of the cyclopentanone. A variety of terminal and internal alkenes serve as two-carbon synthons for installation of alkyl, alkenyl, and (hetero)aryl substituents at the β-positions. Depending on the reactant combination, the cycloaddition is best performed either in hexafluoroisopropanol as the reaction solvent or with ZnCl_2_ as a Lewis acid in trifluoroethanol. Stereochemical analyses of the reaction products using *trans*- and *cis*-1,2-disubstituted alkenes support a stepwise cyclisation mechanism. The cyclopentanone products are versatile intermediates to other valuable compound classes such as diastereomerically pure δ-lactones and cyclopentanols.

## Abstract 2

Diastereoselective synthesis of cyclopentanones with readily available reagents.

## Graphical abstract

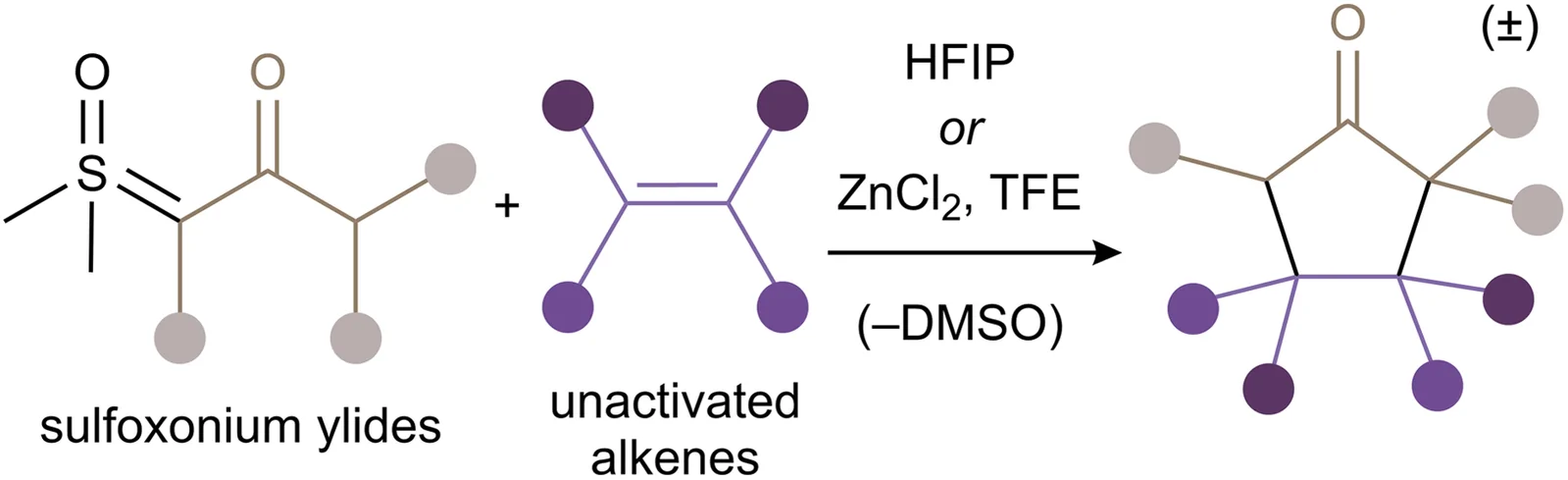

## Introduction

**Scheme 1:**
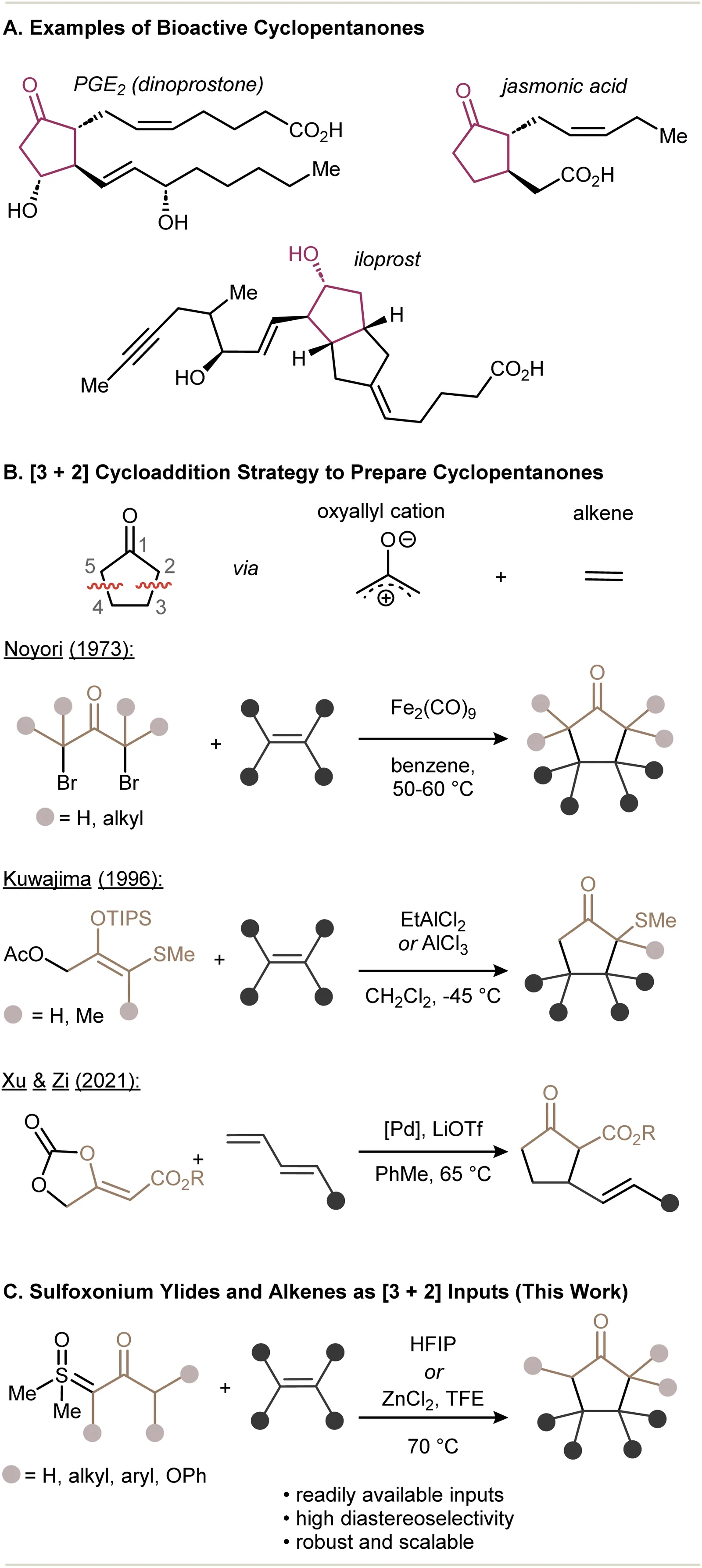
Bioactive examples and [3 + 2] synthesis routes to cyclopentanones.

Cyclopentanones are commonly found in natural products such as steroids and prostaglandins as well as in approved drugs as exemplified by estrone, dinoprostone, and iloprost (Scheme 1A).^1^ These structures are also important to the flavour and fragrance industries, as illustrated by jasmonic acid.^2^ Due to their prevalence and importance, numerous synthetic approaches to these structures have been developed, including, but not limited to, functionalisation of the cyclopentenone core,^3^ ring expansion and/or rearrangement,^4^ intramolecular cyclisation,^5^ intermolecular cascade cyclisation,^6^ and photochemically^7^ or thermally^8^ induced cycloaddition. One notable strategy is the convergent synthesis of substituted cyclopentanones *via* [3 + 2] cycloaddition using an oxyallyl cation precursor as a three-carbon synthon and an alkene as a two-carbon synthon (Scheme 1B).^9–13^ With the vast number of synthetically accessible alkenes, a broad array of substituents can theoretically be introduced at the C3 and C4 positions of the cyclopentanone core. Depending on the types of three-carbon synthon, a variety of substitutions at the C2 and C5 positions might also be incorporated.

In a seminal study on this [3 + 2] cycloaddition approach, Noyori reported on the stoichiometric activation of symmetric alkyl-substituted α,α′-dibromo ketones with Fe_2_(CO)_9_ to generate an oxyallyl-iron species that then reacts with aryl-substituted alkenes to diastereoselectively produce substituted cyclopentanones.^10^ Kuwajima demonstrated specific access to α-thioether-substituted cyclopentanones by utilising allyl acetates that in the presence of Lewis acids provide 1-(methylthio)-2-siloxyallyl cationic species.^11^ These species then underwent annulation with vinyl sulfides, vinyl methyl ether, styrenes, and trialkyl-substituted alkenes in a moderate to excellent diastereoselectivity.

More recently, inspired by Trost's studies on Pd-oxyallyl species,^14^ Xu and Zi devised a Pd-catalysed diastereoselective synthesis of cyclopentanone carboxylate esters using methylene ethylidene carbonates and 1,3-dienes.^12^ Despite these innovative precedents, the chemistry community would benefit from the development of a straightforward and general method for the synthesis of cyclopentanones enabling the facile variation of substituents at all four ring positions from readily available two- and three-carbon synthons.

Our lab has an active program on C–H functionalisation approaches based upon C–H bond activation and sequential addition to two different coupling partners.^15^ During our investigation of sequential addition to alkenes and sulfoxonium ylides,^16^ we serendipitously discovered the straightforward annulation of sulfoxonium ylides with alkenes (Scheme 1C). This method expands the scope of accessible multi-substituted cyclopentanones by coupling mono- and di-substituted sulfoxonium ylides bearing alkyl, aryl, or phenoxy groups at the α-C(sp^3^) position with unactivated internal and terminal alkenes in high diastereoselectivity. A wide array of substrates were effective inputs by using two complementary reaction conditions: (1) hexafluoroisopropanol (HFIP) as the solvent and (2) ZnCl_2_ in trifluoroethanol (TFE). Notably, sulfoxonium ylides are popular and increasingly used reagents in synthesis due to their stability, crystallinity, scalability, and straightforward preparation in only one or two steps.^17–19^ Their annulation with alkenes releases non-volatile dimethyl sulfoxide (DMSO) as a byproduct as well. Given these advantages, we envisioned that this operationally simple and robust method would be practically useful for the diastereoselective synthesis of substituted cyclopentanones and their derivatives.

## Results and discussion

### Optimisation and reaction conditions

The first set of conditions (conditions A) was optimised with the sulfoxonium ylide 1a and 1-methoxy-4-vinylbenzene (2a) in HFIP at 70 °C, which resulted in complete consumption of 1a to provide cyclopentanone 3a in 72% yield (entry 1 of Table 1). The cyclopentanone was not obtained when HFIP was replaced with other common solvents such as 1,2-dichloroethane (DCE), TFE, DMSO, or water (entry 2). The well-documented mild acidity, hydrogen-bond donating ability, and low nucleophilicity of HFIP are likely important factors in enabling the desired transformation.^20^

**Table 1:** Optimisation table for conditions A using 1a and 2a^a^

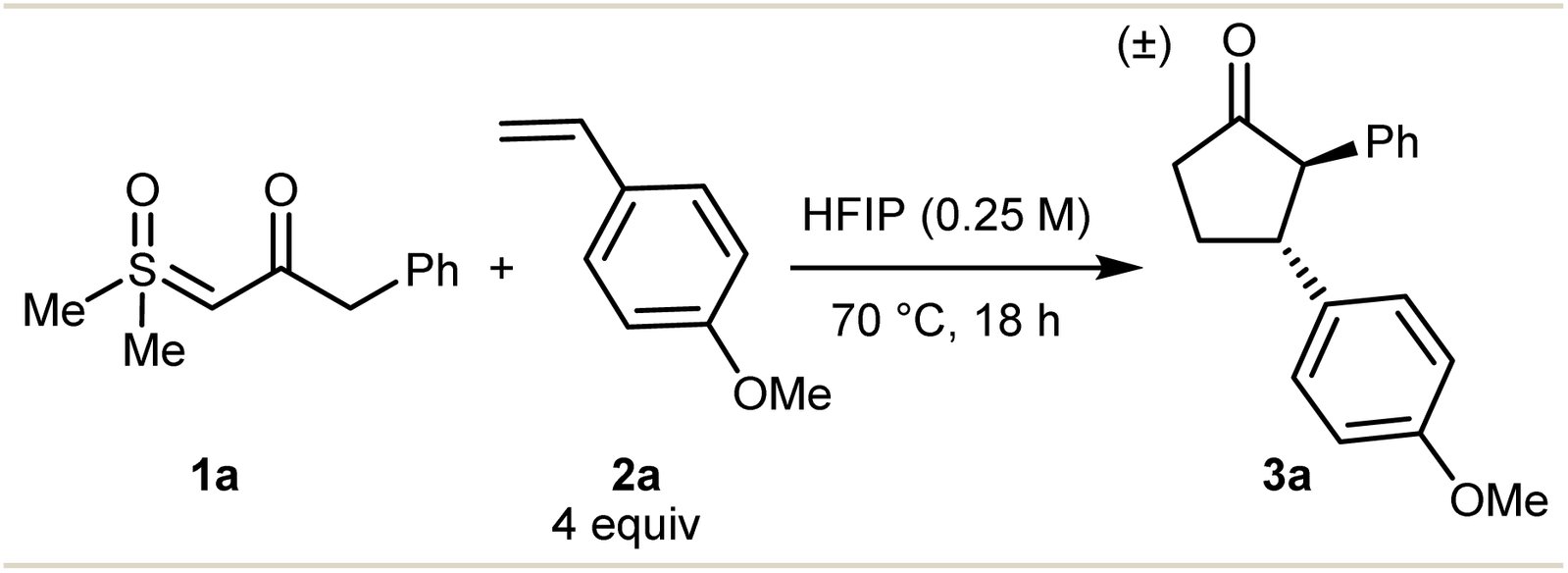		
Entry	Variation from standard conditions	Yield of 3a^b^ (%)
1	None	72
2	DCE, TFE, DMSO, or H_2_O as solvent	0
3	Performed at 50 °C	56
4	Performed at 90 °C	75
5	Performed for 6 h	75
6	K_2_CO_3_ (1 equiv.) as additive	73
7	ZnCl_2_ (1 equiv.) as additive	15
8^c^	ZnCl_2_ (1 equiv.) as additive	50
9^d^	ZnCl_2_ (1 equiv.) as additive	47
10^d^	Diphenyl phosphate (0.2 equiv.) as additive	48
11	H_2_O (5 equiv.) as additive	70
12	Benchtop set-up, air instead of N_2_	70

a Reaction conditions A: 1 (0.1 mmol), 2 (0.4 mmol) in HFIP ([1] = 0.25 M) at 70 °C for 18 h.

b Yield determined by crude ^1^H NMR spectroscopic analysis relative to trimethyl(phenyl)silane as standard.

c DCE as solvent.

d TFE as solvent.

A lower yield of 3a was observed upon reducing the reaction temperature to 50 °C (entry 3). In contrast, increasing the reaction temperature from 70 to 90 °C resulted in a comparable yield of 3a (entry 4). For 1a and 2a at 0.1 mmol scale, the reaction was complete within the first six hours (entry 5). However, we maintained an 18 h reaction time for evaluating substrate scope because the timing of reaction completion varied across substrates. Moreover, the desired products did not decompose under the reaction conditions.

While base has been reported to promote the formation of oxyallyl cations in the presence of certain leaving groups,^8*d*,*f*,19,21^ K_2_CO_3_ had no effect in our system (entry 6). While adding a Lewis acid such as ZnCl_2_ significantly reduced the yield to 15% (entry 7), this additive facilitated the desired reactivity when other solvents such as DCE^22^ and TFE were employed (entries 8 and 9). As noted previously, cyclopentanone 3a was not obtained when these solvents were used without an additive (entry 2). In TFE, replacing ZnCl_2_ with a catalytic amount of the Brønsted acid, diphenyl phosphate, resulted in a comparable yield of 3a (entry 10). Significantly, we did not observe any reduction in yield of 3a when an excess amount of water was added (entry 11) or when the reaction was set up in air (entry 12), attesting to the robustness of our method.

While conditions A were effective for unhindered electron-rich alkenes such as 2a, we observed lower yields for electron-neutral, electron-deficient, and sterically hindered alkenes. However, we were able to develop a second set of conditions to achieve acceptable yields for these less reactive alkene inputs (conditions B, Table 2). For sulfoxonium ylide 1a and 1-methyl-4-vinylbenzene (2b), treatment with ZnCl_2_ in TFE afforded 3b in 70% yield (entry 1). ZnCl_2_ was a crucial additive, as TFE alone did not yield any 3b (entry 2). While conditions A were more effective than conditions B for the synthesis of 3a (entry 1 *vs.* 9 of Table 1), for the synthesis of 3b, conditions B were clearly superior to conditions A (entry 1 *vs.* 3 of Table 2), demonstrating the complementarity of the two conditions. The Lewis acid additive likely effectively activates the sulfoxonium ylide in TFE to promote the desired reactivity with less reactive alkenes, while more reactive alkenes were found to decompose under these conditions (see Mechanistic experiments in the SI).

**Table 2:** Optimisation table for conditions B using 1a and 2b^a^

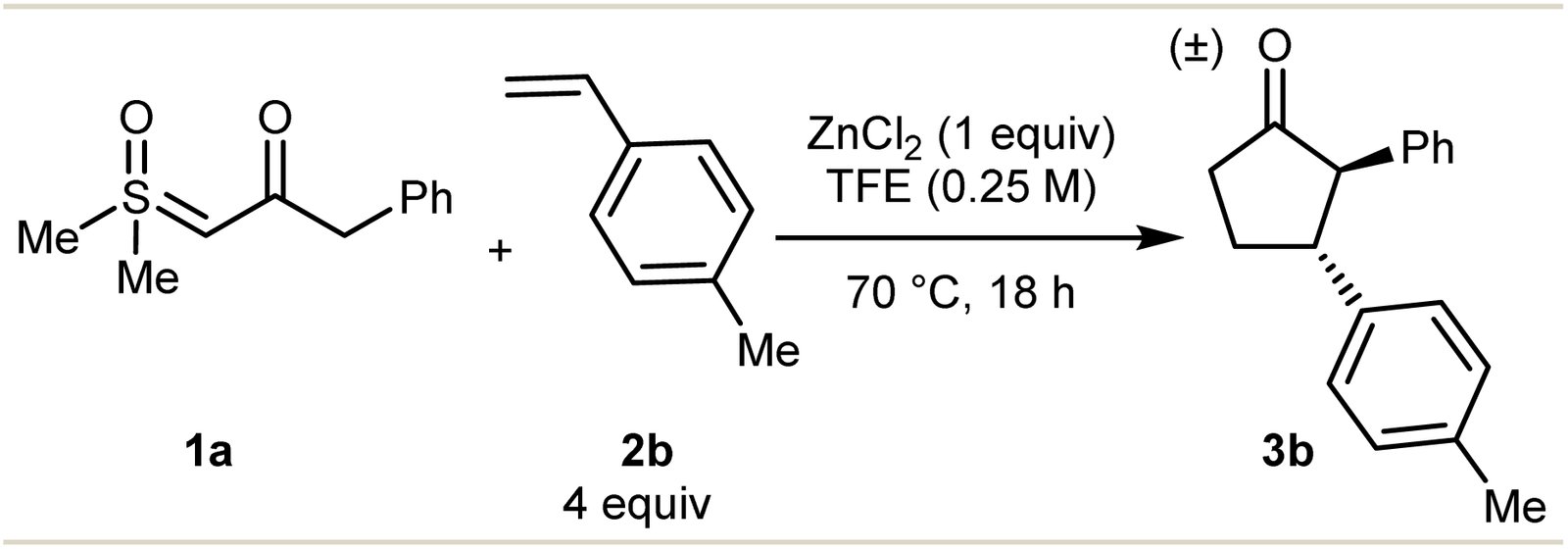		
Entry	Variation from standard conditions	Yield of 3b^b^ (%)
1	None	70
2	No ZnCl_2_	0
3	No ZnCl_2_, HFIP as solvent	47
4	HFIP as solvent	43
5	DCE as solvent	46
6	0.5 equiv. of ZnCl_2_	62
7	K_2_CO_3_ instead of ZnCl_2_	0
8	Zn(OTf)_2_ instead of ZnCl_2_	67
9^c^	Diphenyl phosphate instead of ZnCl_2_	71

a Reaction conditions B: 1 (0.1 mmol), 2 (0.4 mmol), ZnCl_2_ (0.1 mmol) in TFE ([1] = 0.25 M) at 70 °C for 18 h.

b Yield determined by crude ^1^H NMR spectroscopic analysis relative to trimethyl(phenyl)silane as standard.

c 0.2 equiv. of additive. Tf = triflyl.

Utilising ZnCl_2_ in either HFIP or DCE instead of TFE resulted in a lower yield of 3b (entries 4 and 5). With 0.5 instead of 1 equivalent of ZnCl_2_, a slight reduction in the yield of 3b was observed (entry 6). The base additive K_2_CO_3_ was not competent here (entry 7). Zn(OTf)_2_, which has less coordinating triflate relative to chloride counterions, provided similar results (entry 8). A catalytic amount of diphenyl phosphate could also be used in place of ZnCl_2_ to maintain a comparable yield of 3b (entry 9).

### Substrate scope

**Scheme 2:**
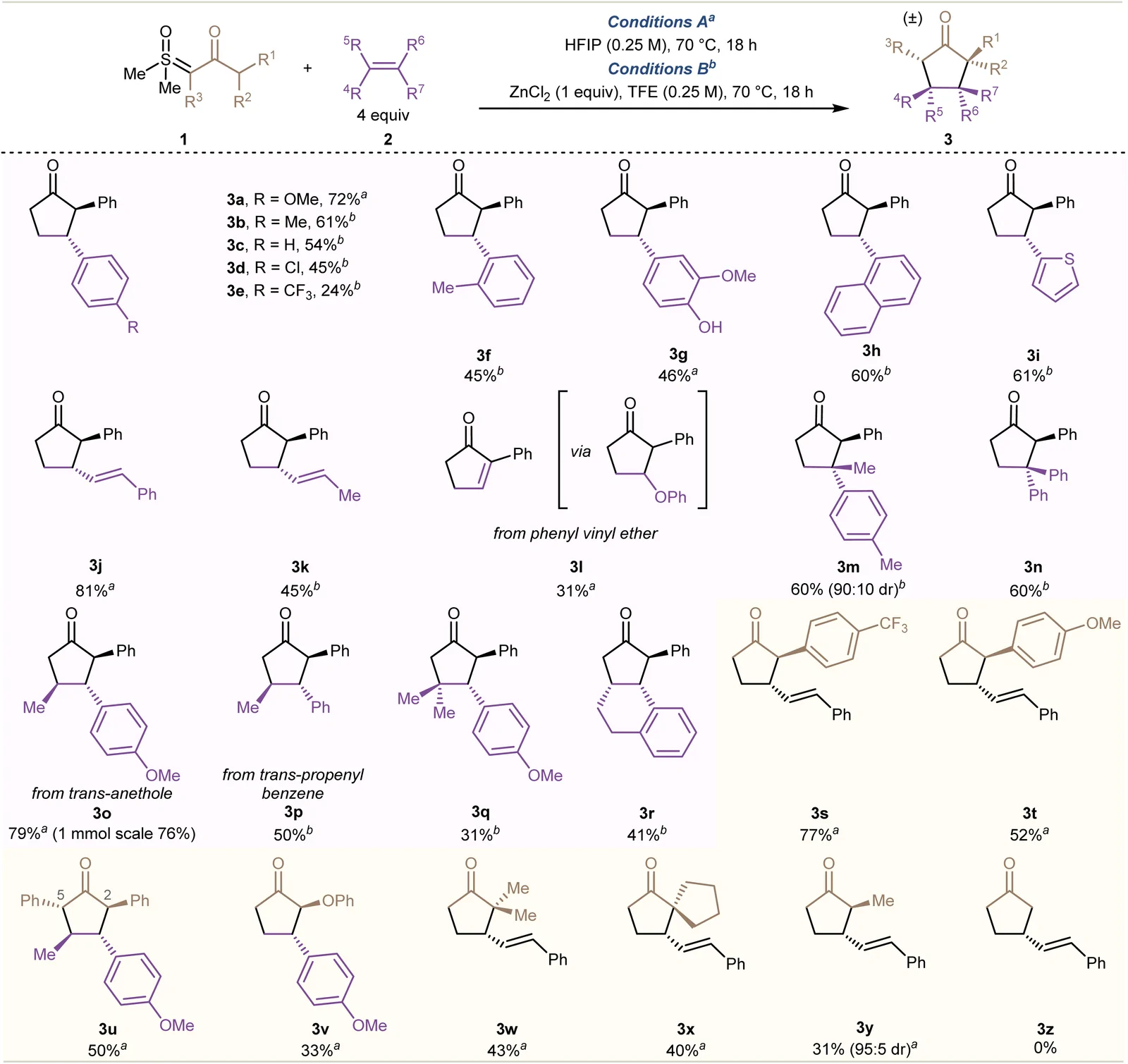
Substrate scope table. Isolated yields reported (>95 : 5 dr unless otherwise noted). ^*a*^ Reaction conditions A: 1 (0.2 mmol), 2 (0.8 mmol) in HFIP ([1] = 0.25 M) at 70 °C for 18 h. ^*b*^ Reaction conditions B: 1 (0.2 mmol), 2 (0.8 mmol), ZnCl_2_ (0.2 mmol) in TFE ([1] = 0.25 M) at 70 °C for 18 h.

Various sulfoxonium ylides and alkenes were evaluated with the two sets of optimised conditions (Scheme 2). We started with the terminally monosubstituted alkenes (3a–3l), with each affording the disubstituted cyclopentanone products with high *trans* diastereoselectivity of >95 : 5 dr as determined by NMR analysis with the relative stereochemistry based on previously reported assignments for α- and β-diaryl cyclopentanones. In general, the alkenes bearing electron-rich substituents provided higher yields. While methoxy-substituted 3a was obtained in 72% yield, a moderate reduction in yield was observed upon substitution of the *para*-methoxy group with methyl (3b), H (3c), chloro (3d), and trifluoromethyl (3e) substituents. The sterics introduced by an *ortho*-methyl substituent (3f) decreased the yield relative to the electronically similar *para*-methyl derivative (3b) but still delivered the desired product in 45% yield. A vanillin-derived alkene, 2-methoxy-4-vinylphenol (2g), gave rise to 3g, establishing that phenols are compatible with our reaction conditions. Other aromatic systems, such as 1-naphthyl (3h) and 2-thiophene (3i), were readily incorporated in good yields.

Conjugated 1,3-dienes were also effective inputs, selectively providing [3 + 2] cycloaddition products instead of the [4 + 3] products that are the primary products for other cycloaddition approaches.^8*c*,12,23^ Both aryl and alkyl monosubstituted butadienes were effective reactants. While phenyl substituted butadiene provided 3j in an excellent 81% yield, the methyl substituted derivative gave 3k in moderate yield. Interestingly, upon utilising phenyl vinyl ether (2l) as the alkene input, we did not detect the presence of the expected product 3-phenoxy-2-phenylcyclopentan-1-one but instead isolated cyclopentenone 3l*via in situ* elimination of the phenolate group.

Next, terminal 1,1-disubstituted alkenes were examined. Using 1-methyl-4-(prop-1-en-2-yl)benzene (2m), we confirmed the *trans*-disposition of the alkyl and aryl groups for the major diastereomer 3m as determined by 1D NOESY analysis (see SI). For this substrate, under conditions A, we also noticed competing byproduct formation, in which the 1-phenylpropan-2-one motif from 1a undergoes an allylic addition to the methyl group of 2m. In contrast, conditions B not only improved the yield of 3m but also suppressed the undesired reactivity. Additionally, 1,1-diphenylethylene provided cyclopentanone 3n with three phenyl substituents in good yield.

The internal 1,2-disubstituted alkenes were viable substrates as well. Notably, *trans*-anethole delivered 3o, which has three stereogenic centres, as a single diastereomer in 79% yield. The *trans* stereochemistry of the alkene was retained in the product; by 1D NOESY, the α-methyl proton at 2.4 ppm interacted with the α-phenyl proton at 3.5 ppm but not with the α-methoxyphenyl proton at 2.9 ppm. At a higher 1.0 mmol scale, 3o was obtained in 76% yield, demonstrating the scalability of the reaction. The analogous product 3p with an unsubstituted phenyl substituent was obtained in 50% yield. A more hindered internal trisubstituted alkene was also exemplified, resulting in product 3q. Furthermore, 1,2-dihydronaphthalene, an endocyclic alkene, afforded 3r with a bicyclo[4.3.0]nonane core. We did not observe synthetically useful yields for aliphatic alkenes and acrylates (see Unsuccessful substrates in the SI).

After establishing a broad alkene scope, we investigated a range of sulfoxonium ylides. The *para*-trifluoromethyl substituted input (3s) gave a comparable yield of 77% as the parent substrate (3j). However, the methoxy substituent (3t) led to a noticeable decrease in yield to 52%, indicating that more electron deficient aromatic substituents at the α-C(sp^3^) position of the ylide are preferred. A sulfoxonium ylide with two phenyl groups allowed for substitution at both α-carbon positions of the cyclopentanone product and generated a tetra-substituted cyclopentanone (3u) upon reacting with *trans*-anethole. NOESY again confirmed the configuration as the α-phenyl proton of C2 at 3.6 ppm interacted with the α-methyl proton at 2.5 ppm, and the α-phenyl proton of C5 at 3.2 ppm interacted with the α-methoxyphenyl proton at 3.0 ppm. Apart from the aryl groups, a heteroatom component, such as an α-phenoxy group (3v), was introduced with a phenoxy-substituted ylide.

Alkyl substituents on the sulfoxonium ylides could also be employed. We were pleased to find that the dialkyl substituted ylides (3w and 3x) gave rise to challenging to introduce all-carbon quaternary centres.^24^ Significantly, the ylide incorporating a cyclopentyl group provided access to the spirocyclic cyclopentanone 3x. The simple 2-methylcyclopentanone 3y was obtained, albeit in only a modest yield, and a sulfoxonium ylide without any substituents at the α-C(sp^3^) position was not a viable input with none of the desired product 3z detected.

### Mechanistic studies and proposed mechanism

**Scheme 3:**
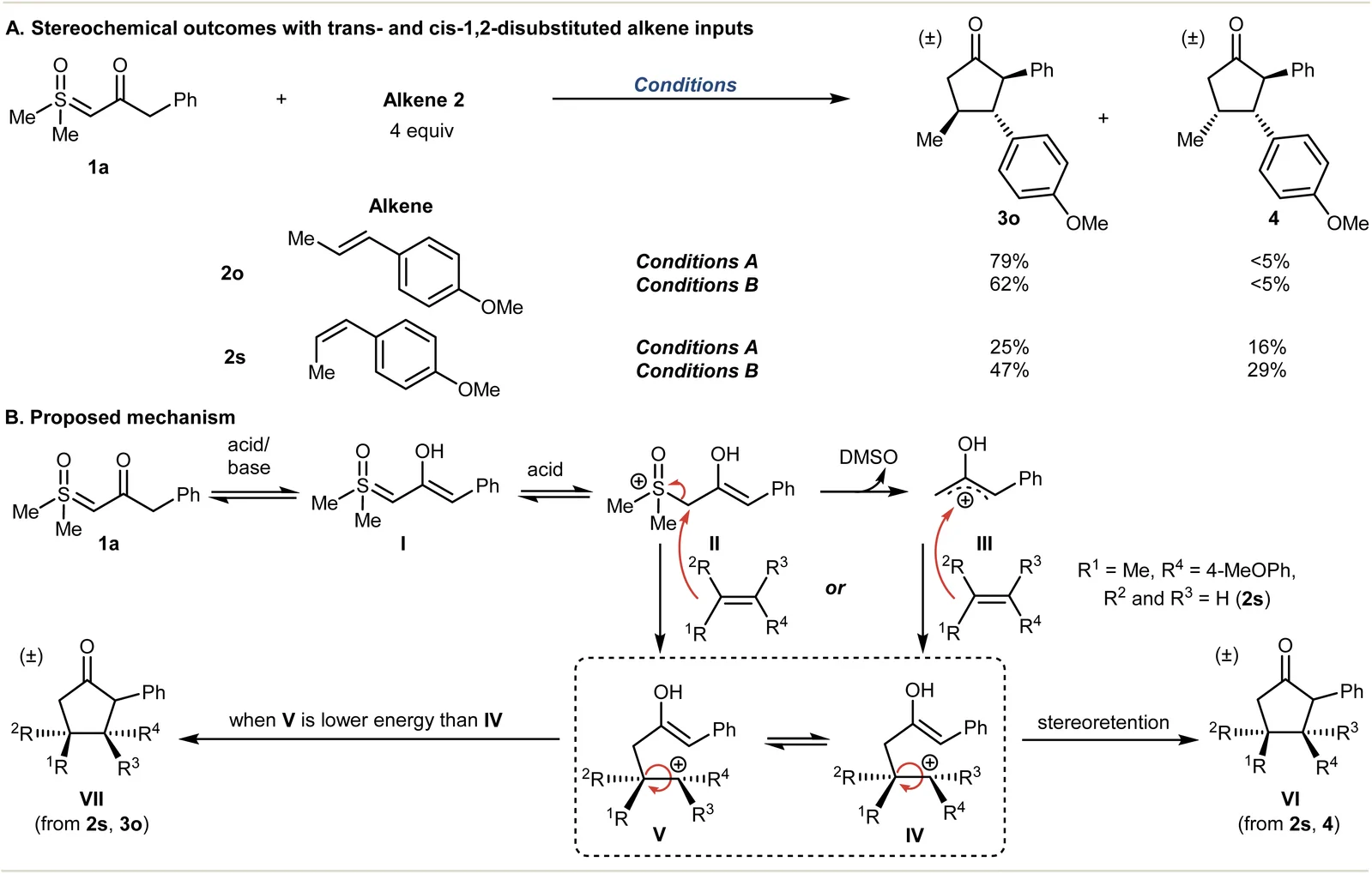
Mechanistic studies. (A) Stereochemical outcomes of the products using 2o and 2s as starting substrates. The products of 2s were isolated as diastereomeric mixtures, and the ratio of 3o : 4 (62 : 38) was determined by ^1^H NMR integration. (B) Proposed mechanism.

From the substrate scope table, we noticed that the stereochemistry of *trans*-1,2-disubstituted alkenes was conserved in the cyclopentanone products (3o and 3p). We therefore investigated *cis*-anethole (2s) to ascertain whether product 4 would be obtained in a stereospecific manner and to assess whether the results would be consistent across the two conditions, A and B (Scheme 3A). To address these questions, we ran four sets of experiments: using *trans*-anethole (2o) under conditions A and conditions B and using *cis*-anethole (2s) under conditions A and B. With 2o, we obtained a high yield of 3o under both conditions and did not detect any of the diastereomeric product 4. In contrast, 2s resulted in a 62 : 38 diastereomeric mixture of products 3o and 4 under both conditions, clearly indicating that the stereochemistry of the starting alkene had not been retained in the product. We also performed an analogous set of experiments with *trans*- and *cis*-propenylbenzene under conditions B and observed a qualitatively similar trend (see SI). As an important control, we established that *trans*- and *cis*-anetholes and propenylbenzenes do not isomerize under the reaction conditions when the sulfoxonium ylides are not present (see SI).

Collectively, these findings support a stepwise cycloaddition pathway (Scheme 3B). It should be noted that a thermal cycloaddition of an allyl cation with an alkene is a [_π_2_s_ + _π_2_s_] process, which is symmetry-forbidden by the Woodward–Hoffman rules;^25^ therefore, a stepwise mechanism rather than a concerted one would be expected. Our proposed mechanism starts with the equilibration of sulfoxonium ylide 1a to its enol tautomer I. Upon protonation of I to give II under acidic conditions, the alkene could either directly attack II in an S_N_2 fashion or attack the oxyallyl cation species III generated upon release of DMSO.^19^ Either pathway would first lead to IV, which could rapidly cyclise to the stereoretentive cyclopentanone product VI. However, competing bond rotation from IV to V occurs when the cationic species V is more thermodynamically stable, resulting in the diastereomeric cyclopentanone VII. The 62 : 38 diastereomeric mixture of cyclopentanones 3o and 4 obtained for *cis*-anethole (2s) is consistent with competing rotation of the cationic species IV to the more stable V prior to cyclisation.

The proposed mechanism is also supported by the substrate-reactivity trends in Scheme 2, in that substituents at the α-position of the sulfoxonium ylide that provide a more favourable enol tautomer equilibrium result in higher product yields. Additionally, a more electron-rich substituent on the alkene renders the formation of IV and V more favourable.

Because diphenyl phosphate was as an effective acid catalyst (entry 10 of Table 1 and entry 9 of Table 2), we also tested whether a chiral phosphoric acid, (*S*)-TRIP, enables an enantioselective transformation (see SI). However, when (*S*)-TRIP was utilised as the acid additive, racemic cyclopentanone 3a was obtained regardless of whether TFE or DCE was used as solvent.

### Synthetic elaborations of the cyclopentanone product

**Scheme 4:**
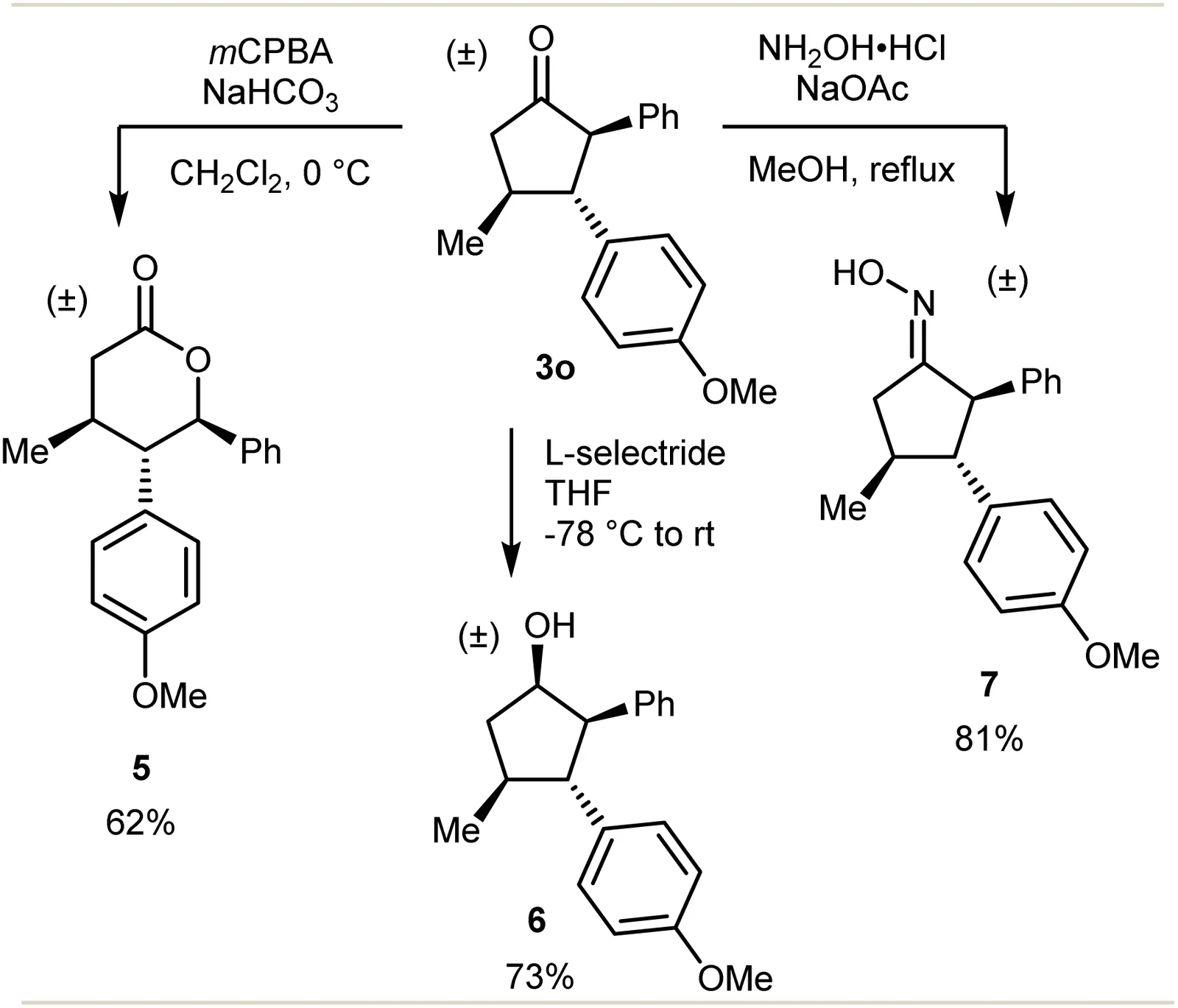
Synthetic elaborations of the cyclopentanone 3o.

We performed various transformations on cyclopentanone 3o to demonstrate the versatility of the ketone functionality for accessing other useful product classes (Scheme 4). For example, a Baeyer–Villiger reaction employing *m*CPBA and NaHCO_3_ enabled ring expansion of the cyclopentanone to give the δ-lactone 5. The diastereoselective reduction of 3o with l-selectride afforded the diastereomerically pure cyclopentanol 6, with X-ray crystallography providing rigorous confirmation of the depicted relative configuration. Condensation of 3o with hydroxylamine also furnished the cyclopentanone oxime, and its *trans* isomer 7 could be isolated as a single stereoisomer in 81% yield.

## Conclusions

We report a [3 + 2] cycloaddition approach for the highly diastereoselective synthesis of multi-substituted cyclopentanones from sulfoxonium ylides and alkenes. This reaction utilises readily available starting materials and a simple reaction set-up that does not require any special precautions toward moisture or air. The two sets of reaction conditions complement one another to establish a broad substrate scope. Mechanistic studies were conducted using *trans*- and *cis*-1,2-disubstituted alkenes with only the *cis*-alkenes providing a diastereomeric product mixture, suggesting a stepwise mechanism for the cycloaddition. A cyclopentanone product was converted into other valuable product classes in diastereomerically pure form, including a δ-lactone, a cyclopentanol, and a cyclopentanone oxime. Overall, the reported method offers a straightforward and robust route to cyclopentanones and their derivatives, which are prevalent in natural products and are useful intermediates and end products in a variety of industrial contexts.

## Author contributions

J. Y. and J. A. E. conceived the project. J. Y. performed the experiments and collected the data. S. M. K. solved the X-ray crystal structure of compound 6. J. A. E. supervised the project and acquired funding for the project.

## Conflicts of interest

There are no conflicts to declare.

## Supplementary Material

SC-OLF-D6SC05806J-s001

SC-OLF-D6SC05806J-s002

SC-OLF-D6SC05806J-s003

SC-OLF-D6SC05806J-s004

## Data Availability

CCDC 2566079 (compound 6) contains the supplementary crystallographic data for this paper.^26^ Experimental procedures, analytical data, NMR and HPLC spectra, X-ray crystallographic data, and primary data (NMR and HRMS) have been uploaded as part of the supplementary information (SI). Supplementary information is available. See DOI: https://doi.org/10.1039/d6sc05806j.
