# Supplementary material for: Diastereoselective synthesis of substituted cyclopentanones via [3 + 2] cycloaddition of sulfoxonium ylides with alkenes
Source: Chem Sci. 2026 Sep 21;17(42):20636–42. doi: 10.1039/d6sc05806j (PMC13591502; doi:10.1039/d6sc05806j)
Supplement: Supplementary file 2 [file SC-017-D6SC05806J-s001.zip › HRMS/3b-MS.pdf]

Spectrum

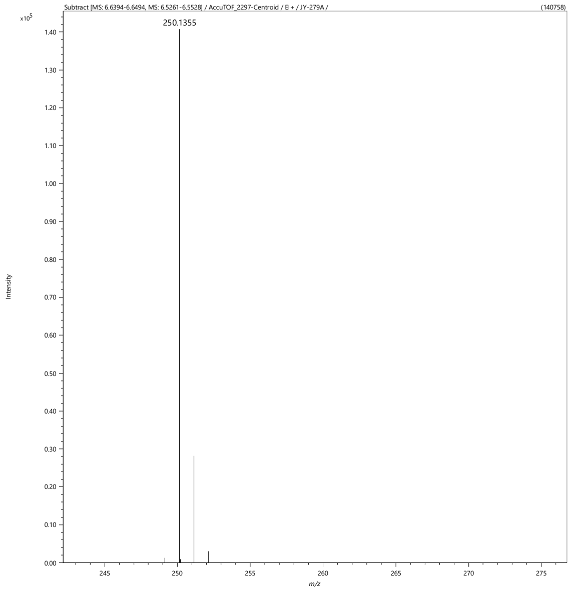

Elemental Composition

Parameters

| | |
|---|---|
| Tolerance: | ±5.00 ppm |
| Electron: | Odd/Even |
| Charge: | +1 |
| DBE: | -1.5 - 40.0 |

Elements Set 1:

| Symbol | C | H | O | N |
|---|---|---|---|---|
| Min | 0 | 0 | 0 | 0 |
| Max | 60 | 100 | 10 | 5 |

Results

| Mass | Intensity | Formula | Charge | Calculated Mass | Mass Difference [mDa] | Mass Difference [ppm] | DBE |
|---|---|---|---|---|---|---|---|
| 250.13549 | 140757.50 | C18 H18 O | +1 | 250.13522 | 0.27 | 1.08 | 10.0 |
