# Supplementary material for: Diastereoselective synthesis of substituted cyclopentanones via [3 + 2] cycloaddition of sulfoxonium ylides with alkenes
Source: Chem Sci. 2026 Sep 21;17(42):20636–42. doi: 10.1039/d6sc05806j (PMC13591502; doi:10.1039/d6sc05806j)
Supplement: Supplementary file 2 [file SC-017-D6SC05806J-s001.zip › HRMS/3d-MS.pdf]

Spectrum

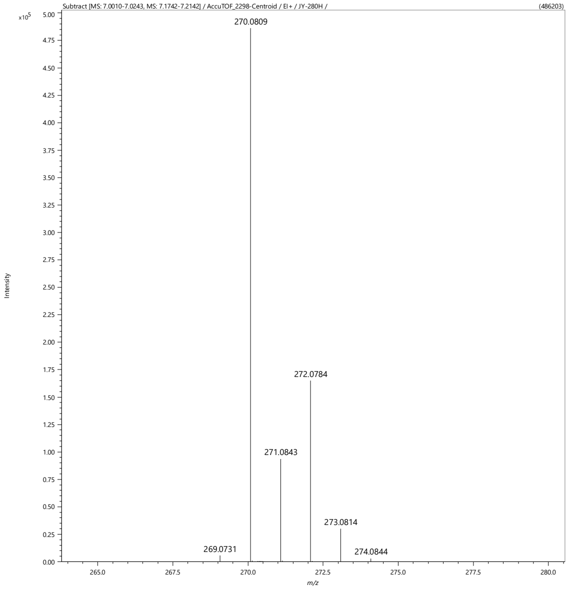

Elemental Composition

Parameters

Tolerance:	±5.00 ppm
Electron:	Odd/Even
Charge:	+1
DBE:	-1.5 - 40.0

Elements Set 1:

Symbol	C	H	O	N	Cl
Min	0	0	0	0	0
Max	60	100	10	5	1

Results

Mass	Intensity	Formula	Charge	Calculated Mass	Mass Difference [mDa]	Mass Difference [ppm]	DBE
270.08094	486202.94	C6 H14 N4 O8	+1	270.08061	0.32	1.20	2.0
		C17 H15 Cl O	+1	270.08059	0.34	1.27	10.0
		C8 H16 N O9	+1	270.08196	-1.02	-3.78	1.5
