# Supplementary material for: Diastereoselective synthesis of substituted cyclopentanones via [3 + 2] cycloaddition of sulfoxonium ylides with alkenes
Source: Chem Sci. 2026 Sep 21;17(42):20636–42. doi: 10.1039/d6sc05806j (PMC13591502; doi:10.1039/d6sc05806j)
Supplement: Supplementary file 2 [file SC-017-D6SC05806J-s001.zip › HRMS/3k-MS.pdf]

Spectrum

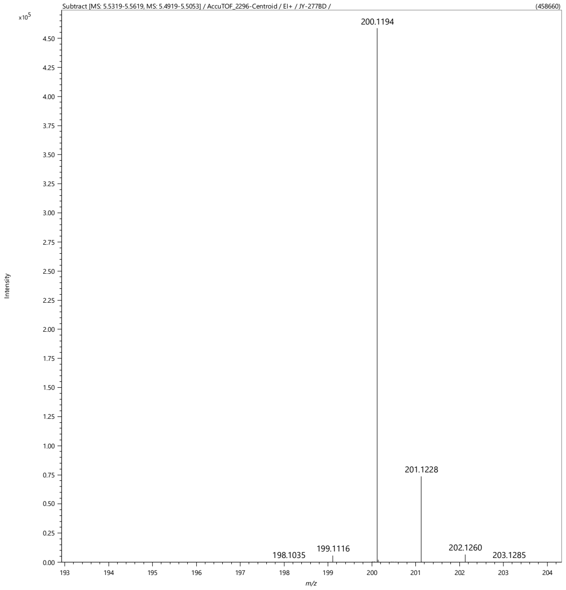

Elemental Composition

Parameters

Tolerance:	±5.00 ppm
Electron:	Odd/Even
Charge:	+1
DBE:	-1.5 - 40.0

Elements Set 1:

Symbol	C	H	O	N
Min	0	0	0	0
Max	60	100	10	5

Results

Mass	Intensity	Formula	Charge	Calculated Mass	Mass Difference [mDa]	Mass Difference [ppm]	DBE
200.11936	458659.80	C14 H16 O	+1	200.11957	-0.20	-1.01	7.0
