# Supplementary material for: Diastereoselective synthesis of substituted cyclopentanones via [3 + 2] cycloaddition of sulfoxonium ylides with alkenes
Source: Chem Sci. 2026 Sep 21;17(42):20636–42. doi: 10.1039/d6sc05806j (PMC13591502; doi:10.1039/d6sc05806j)
Supplement: Supplementary file 2 [file SC-017-D6SC05806J-s001.zip › HRMS/3n-MS.pdf]

Spectrum

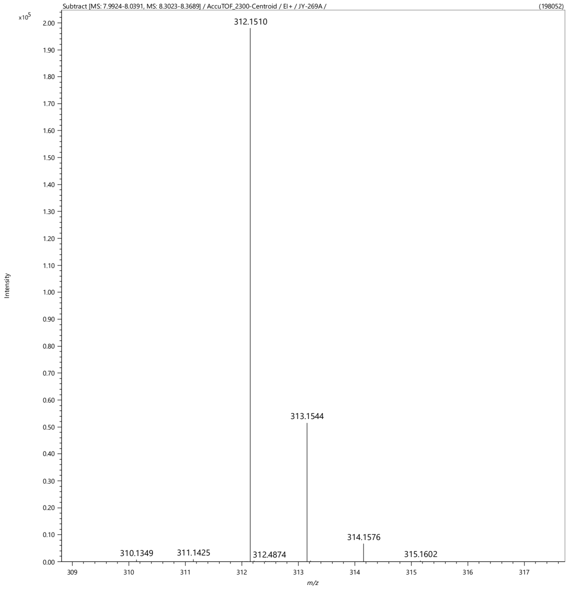

Elemental Composition

Parameters		Elements Set 1:				
Tolerance:	±5.00 ppm	Symbol	C	H	O	N
Electron:	Odd/Even	Min	0	0	0	0
Charge:	+1	Max	60	100	10	5
DBE:	-1.5 - 40.0					

Results

Mass	Intensity	Formula	Charge	Calculated Mass	Mass Difference [mDa]	Mass Difference [ppm]	DBE
312.15103	198052.07	C23 H20 O	+1	312.15087	0.16	0.52	14.0
		C9 H22 N5 O7	+1	312.15137	-0.35	-1.11	1.5
		C21 H18 N3	+1	312.14952	1.50	4.82	14.5
