# Supplementary material for: Diastereoselective synthesis of substituted cyclopentanones via [3 + 2] cycloaddition of sulfoxonium ylides with alkenes
Source: Chem Sci. 2026 Sep 21;17(42):20636–42. doi: 10.1039/d6sc05806j (PMC13591502; doi:10.1039/d6sc05806j)
Supplement: Supplementary file 2 [file SC-017-D6SC05806J-s001.zip › HRMS/3o-MS.pdf]

Spectrum

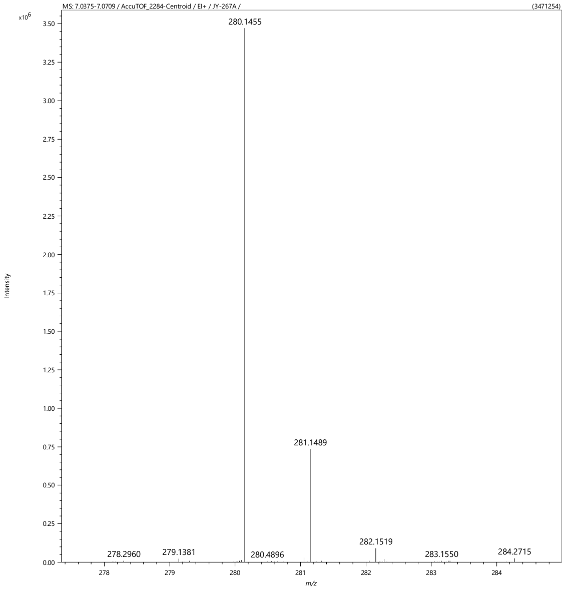

Elemental Composition

Parameters

Tolerance:	±5.00 ppm
Electron:	Odd/Even
Charge:	+1
DBE:	-1.5 - 40.0

Elements Set 1:

Symbol	C	H	O	N
Min	0	0	0	0
Max	60	100	10	5

Results

Mass	Intensity	Formula	Charge	Calculated Mass	Mass Difference [mDa]	Mass Difference [ppm]	DBE
280.14546	3471253.67	C19 H20 O2	+1	280.14578	-0.32	-1.14	10.0
		C17 H18 N3 O	+1	280.14444	1.02	3.65	10.5
