# Supplementary material for: Diastereoselective synthesis of substituted cyclopentanones via [3 + 2] cycloaddition of sulfoxonium ylides with alkenes
Source: Chem Sci. 2026 Sep 21;17(42):20636–42. doi: 10.1039/d6sc05806j (PMC13591502; doi:10.1039/d6sc05806j)
Supplement: Supplementary file 2 [file SC-017-D6SC05806J-s001.zip › HRMS/3s-MS.pdf]

Spectrum

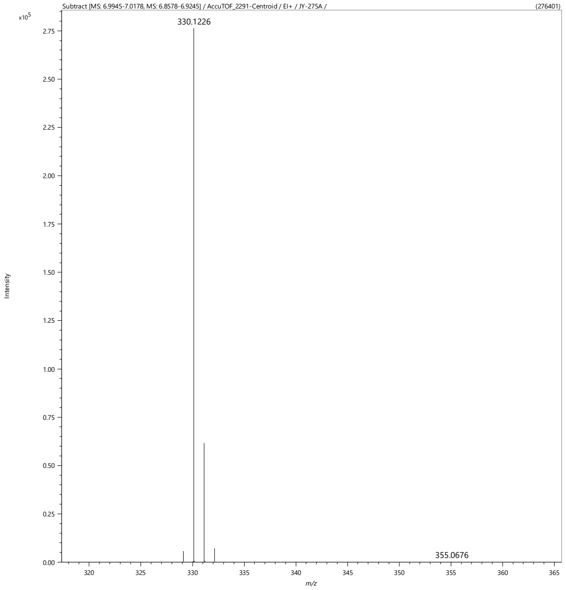

Elemental Composition

Parameters	
Tolerance:	±5.00 ppm
Electron:	Odd/Even
Charge:	+1
DBE:	-1.5 - 40.0

Elements Set 1:

Symbol	C	H	O	N	F
Min	0	0	0	0	0
Max	60	100	10	5	3

Results

Mass	Intensity	Formula	Charge	Calculated Mass	Mass Difference [mDa]	Mass Difference [ppm]	DBE
330.12261	276400.95	C20 H17 F3 O	+1	330.12260	0.01	0.04	11.0
		C14 H19 F N2 O6	+1	330.12217	0.45	1.36	6.0
		C6 H19 F3 N5 O7	+1	330.12311	-0.50	-1.50	-1.5
		C9 H18 F2 N5 O6	+1	330.12197	0.65	1.96	2.5
		C11 H20 F2 N2 O7	+1	330.12331	-0.70	-2.11	2.0
		C20 H16 N3 O2	+1	330.12370	-1.09	-3.30	14.5

Mass	Intensity	Formula	Charge	Calculated Mass	Mass Difference [mDa]	Mass Difference [ppm]	DBE
		C23 H16 F2	+1	330.12146	1.16	3.50	15.0
		C18 H15 F3 N3	+1	330.12126	1.36	4.11	11.5
		C17 H18 N2 O5	+1	330.12102	1.59	4.82	10.0
