# Supplementary material for: Diastereoselective synthesis of substituted cyclopentanones via [3 + 2] cycloaddition of sulfoxonium ylides with alkenes
Source: Chem Sci. 2026 Sep 21;17(42):20636–42. doi: 10.1039/d6sc05806j (PMC13591502; doi:10.1039/d6sc05806j)
Supplement: Supplementary file 2 [file SC-017-D6SC05806J-s001.zip › HRMS/7-MS.pdf]

Spectrum

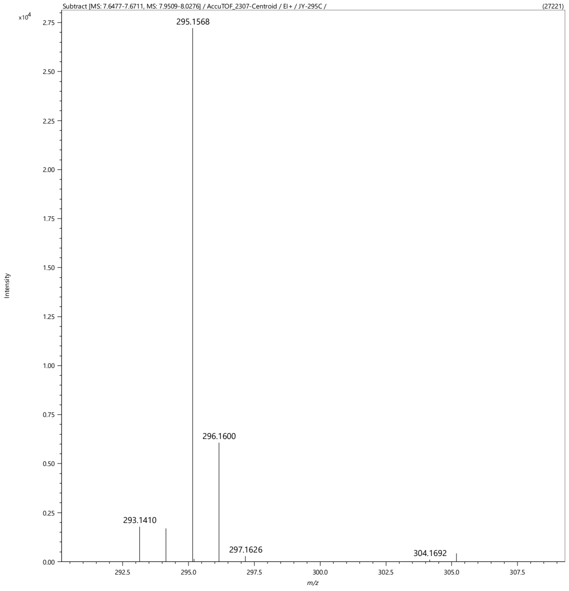


## Elemental Composition

**Parameters**

| | |
|---|---|
| Tolerance: | ±5.00 ppm |
| Electron: | Odd/Even |
| Charge: | +1 |
| DBE: | -1.5 - 40.0 |

**Elements Set 1:**

| Symbol | C | H | O | N |
|---|---|---|---|---|
| Min | 0 | 0 | 0 | 0 |
| Max | 60 | 100 | 10 | 5 |

## Results

| Mass | Intensity | Formula | Charge | Calculated Mass | Mass Difference [mDa] | Mass Difference [ppm] | DBE |
|---|---|---|---|---|---|---|---|
| 295.15678 | 27220.83 | C19 H21 N O2 | +1 | 295.15668 | 0.10 | 0.33 | 10.0 |
| | | C17 H19 N4 O | +1 | 295.15534 | 1.44 | 4.88 | 10.5 |
