# Supplementary material for: Diastereoselective synthesis of substituted cyclopentanones via [3 + 2] cycloaddition of sulfoxonium ylides with alkenes
Source: Chem Sci. 2026 Sep 21. Online ahead of print. doi: 10.1039/d6sc05806j (PMC13591502; doi:10.1039/d6sc05806j)
Supplement: Supplementary file 1 [file SC-OLF-D6SC05806J-s003.pdf]

Supplementary Information (SI) for Chemical Science.
This journal is © The Royal Society of Chemistry 2026

**Supplementary Information**

# Diastereoselective Synthesis of Substituted Cyclopentanones via [3 + 2] Cycloaddition of Sulfoxonium Ylides with Alkenes

Jihyeon Yeo, Sebastian M. Krajewski, and Jonathan A. Ellman*

Department of Chemistry, Yale University, CT 06520, United States
*Email: jonathan.ellman@yale.edu

## Table of Contents


1. General Information S3

2. Preparation of Starting Materials S3
   2.1 Additives S3
   2.2 Sulfoxonium Ylides S3
   2.3 Alkenes S4

3. Procedure for Cycloaddition Reactions and Characterization of Carbocyclic Products S6
   3.1 Products Synthesized via Conditions A and B S6
   3.2 Unsuccessful Substrates S16

4. Mechanistic Experiments S17
   4.1 Using Trans/Cis-Anetholes S17
   4.2 Using Trans/Cis-Propenylbenzenes S23
   4.3 Using Chiral Phosphoric Acid S27

5. Synthetic Elaborations of the Product S30

6. COSY and NOE Experiments for Relative Stereochemistry S32

7. X-Ray Crystallographic Data for Relative Stereochemistry S37

8. NMR Spectra for Characterization S58

9. References S90

## 1. General Information

Unless otherwise noted, reactions were set up in a nitrogen-filled glovebox using glassware oven-dried (120 °C) overnight, which was cooled during the evacuation in the antechamber. Anhydrous solvents were distilled and sparged with nitrogen prior to use. Diethyl ether was eluted through a column of activated alumina and then sparged with argon to remove the 3,5-di-*tert*-butyl-4-hydroxytoluene (BHT) inhibitor before its use when isolating compounds. The solvents were stored in a nitrogen-filled glovebox over activated 3 Å molecular sieves. Microwave vials, magnetic stir bars, and caps were purchased from Biotage® with part numbers 352016 (for 0.5–2 mL), 351521 (for 2–5 mL), and 354833 (for 10–20 mL). The reaction mixtures were heated using a preheated silicone oil bath inside the fume hood. Unless otherwise noted, all starting materials and reagents were purchased from commercial sources and used without further purification.

Unless otherwise noted, all products were purified by flash column chromatography with SiliaFlash® P60 (230–400 mesh) silica gel and/or preparative thin-layer chromatography from Analtech (1 mm $SiO_2$, 20 × 20 cm). For infrared spectra, all noticeable peaks in the range of 4000–500 $cm^{-1}$ at the resolution of 4 $cm^{-1}$ were reported using a FTIR Shimadzu IRTracer-100 spectrometer. $^{1}H$, $^{13}C$, $^{19}F$, and COSY NMR spectra along with 1D NOESY NMR spectra for select compounds were recorded on Agilent DD2 400, 500, or 600 MHz spectrometers at ambient temperature (20–25 °C). Most chemical shifts were reported in ppm relative to the $CDCl_3$ solvent (for $^{1}H$, 7.26 ppm for the $CHCl_3$ signal; for $^{13}C$, 77.16 ppm) or external $CFCl_3$ ($^{19}F$ without $^{1}H$ decoupling). Some chemical shifts were reported relative to the $CD_3CN$ solvent (for $^{1}H$, 1.94 ppm to the $CD_2HCN$ signal; for $^{13}C$, 1.32 and 118.26 ppm). Splitting patterns are indicated as follows: s, singlet; d, doublet; t, triplet; q, quartet; dd, doublet of doublets; ddd, doublet of doublets of doublets; dt, doublet of triplets; dq, doublet of quartets; td, triplet of doublets; tq, triplet of quartets; m, multiplet, with all coupling constants (*J*) reported in Hertz (Hz). High-resolution mass spectra (HRMS) were obtained using electron impact ionization (EI) method on the Waters® GCT PremierTM. Enantiomeric ratios were determined using an Agilent 1200 series HPLC equipped with Chiralpak columns (4.6 × 250 mm, particle size 5 μm) and a multiwavelength detector.

## 2. Preparation of Starting Materials

### 2.1 Additives

Zinc chloride ($ZnCl_2$, min. 97% purity, commercial, white crystalline) was stored in a $N_2$-filled glovebox and used as received. No significant change in yield of the desired product was observed when drying it in a vacuum oven under reduced pressure at 120 °C overnight prior to use (anhydrous, white powder).

### 2.2 Sulfoxonium Ylides

All sulfoxonium ylides were synthesized using literature procedures (**1a** and **1d**,[1] **1b** and **1c**,[2] **1e**,[3] **1f** and **1i**,[4] **1g**,[5] and **1h**[6]) and stored in the glovebox.

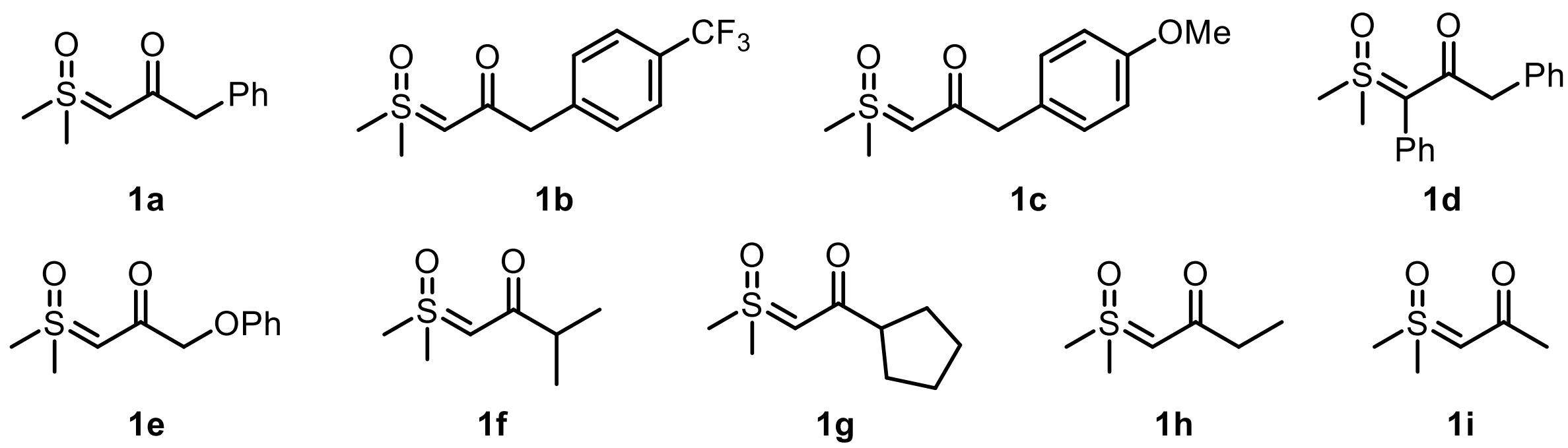


**Chart S1**. Sulfoxonium ylides used for the transformation.

### 2.3 Alkenes

All alkenes except cis-anethole (**2s**, *vide infra*) and 1-methoxy-4-(2-methylprop-1-en-1-yl)benzene (**2q**, via literature procedure[7]) were purchased from commercial sources, and most of them were used without further purification. Exceptions include: styrene (**2c**), which was purified according to the literature procedure,[8] and (*E*)-buta-1,3-dien-1-ylbenzene (**2h**), which was purified via flash silica column using pentanes. The presence of inhibitor did not affect the reaction yields. All alkenes were stored in a –20 °C freezer.

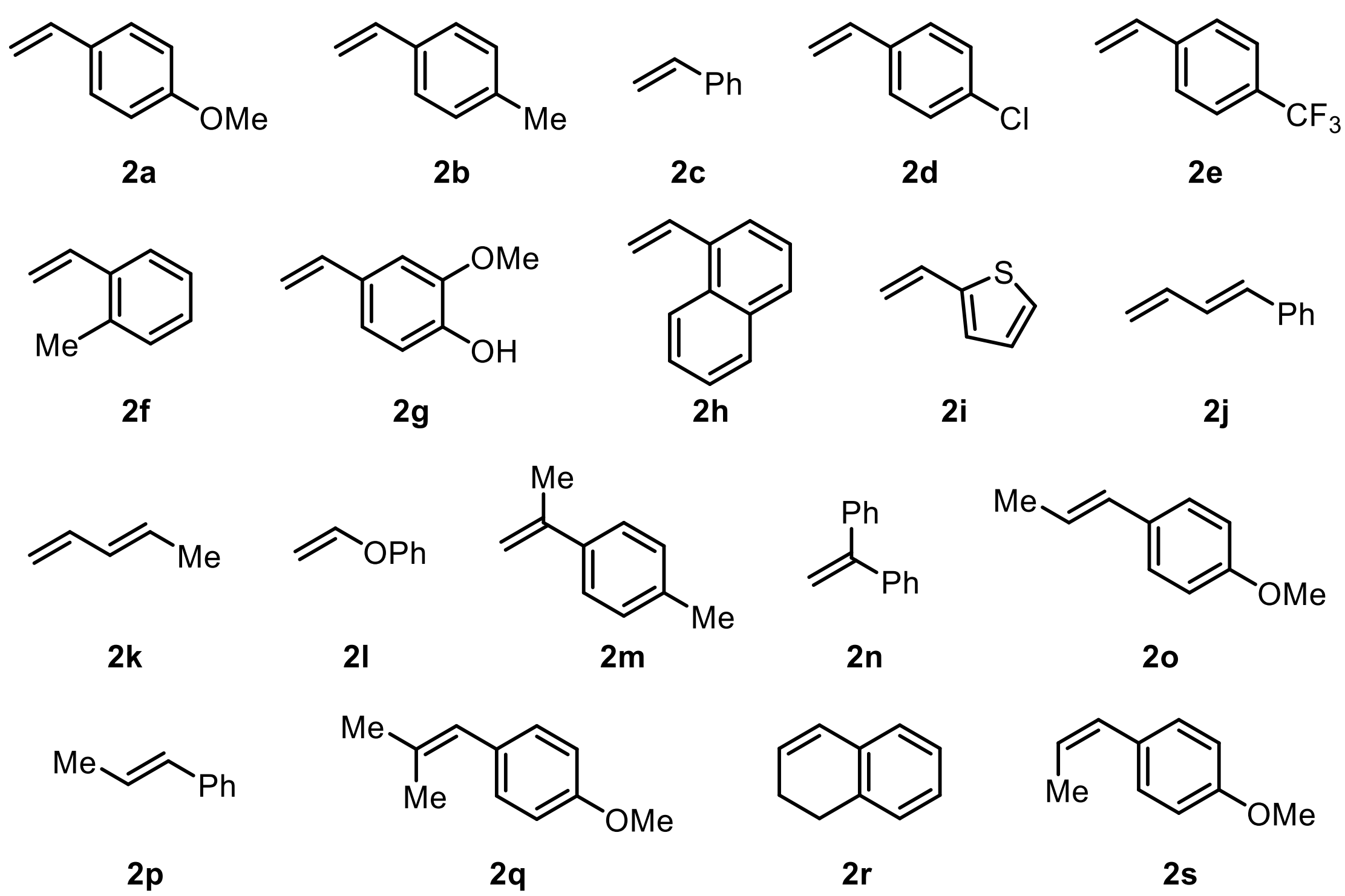


**Chart S2**. Alkenes used for the transformation.

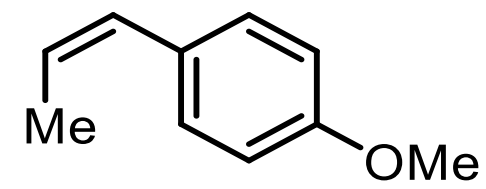


**(*Z*)-1-Methoxy-4-(prop-1-en-1-yl)benzene *or* cis-anethole (2s)**. The substrate was synthesized using a procedure adapted from the literature.[9] To an oven-dried round bottom flask were added (ethyl)triphenylphosphonium bromide (14.0 mmol, 1.40 equiv, 5.20 g) and a stir bar. The flask was evacuated and backfilled with nitrogen thrice. Anhydrous THF (64.0 mL), hexamethylphosphoramide (HMPA, 20.0 mmol, 2.00 equiv, 3.48 mL), and then potassium bis(trimethylsilyl)amide (KHMDS in THF, 1.0 M, dropwise, 13 mmol, 1.3 equiv, 13.0 mL) were sequentially added. Upon the addition of KHMDS, the cloudy white solution turned bright orange. The solution was cooled to –78 °C (acetone/dry ice in a dewar) and stirred for about 20 min. *p*-Anisaldehyde was added dropwise (10.0 mmol, 1.00 equiv, 1.22 mL), and the reaction mixture was gradually warmed to room temperature in a water bath. After 1 h, the crude mixture was concentrated under reduced pressure and then was diluted with 30 mL of DI water and 30 mL of $Et_2O$. The $Et_2O$ layer was isolated, and the aqueous layer was further extracted with $Et_2O$ (50 x 3 mL). The organic layers were combined, washed with 30 mL of brine, and dried over $MgSO_4$. Flash silica column chromatography (5% EtOAc in hexanes) afforded anethole with 92:8 ratio of (*Z*)/(*E*) isomers (1.46 g, 99%). Pure cis-anethole was obtained after iterations of isolations on C18 reverse phase column chromatography, where trans-anethole eluted out at a later retention time compared to cis-anethole. Here, the mixture was loaded with 1.0 mL of 30% $CH_3CN$ in $H_2O$ containing 0.1% TFA. It was purified with 50 g of reverse phase media and a 45-column volume gradient from 30–100% $CH_3CN/H_2O$ containing 0.1% TFA. The compound was diluted with saturated aq. $NaHCO_3$ and extracted with $Et_2O$ (30 x 4 mL). The combined organic layers washed with brine (30 mL), dried over $MgSO_4$, and concentrated, affording the title compound as a pale-yellow oil (0.165 g, 11%). **$^1$H NMR** (500 MHz, $CDCl_3$): δ 7.24 (d, *J* = 8.6 Hz, 2H), 6.88 (d, *J* = 8.8 Hz, 2H), 6.37 (d, *J* = 13.4 Hz, 1H), 5.70 (dq, *J* = 11.6, 7.2 Hz, 1H), 3.81 (s, 3H), 1.89 (dd, *J* = 7.2, 1.9 Hz, 3H). **$^{13}$C{$^1$H} NMR** (126 MHz, $CDCl_3$): δ 158.1, 130.3, 130.0, 129.2, 125.1, 113.5, 55.2, 14.6. The characterization data align with the reported literature values.[9]

## 3. Procedure for Cycloaddition Reactions and Characterization of Carbocyclic Products

### General Procedure A for the Synthesis of Cyclopentanone Derivatives (Conditions A)

In a nitrogen-filled glovebox, a 2–5 mL Biotage® microwave vial was charged with sulfoxonium ylide (0.200 mmol, 1.0 equiv), HFIP (1,1,1,3,3,3-hexafluoro-2-propanol, 800 µL, 0.25 M), and alkene (0.800 mmol, 4.0 equiv), followed by a magnetic stir bar. The vial was sealed, removed from the glovebox, and placed in an oil bath preheated to 70 °C. After stirring for 18 h, the flask was removed from the bath and allowed to cool to room temperature. The reaction mixture was transferred to a glass scintillation vial using $CH_2Cl_2$ and was concentrated. The desired product was obtained via preparative thin layer chromatography or via flash silica chromatography, which is best for cyclopentanone products that decompose on silica over time.

### General Procedure B for the Synthesis of Cyclopentanone Derivatives (Conditions B)

In a nitrogen-filled glovebox, a 2–5 mL Biotage® microwave vial was charged with $ZnCl_2$ (27.3 mg, 0.200 mmol, 1.0 equiv), sulfoxonium ylide (0.200 mmol, 1.0 equiv), TFE (2,2,2-trifluoroethanol, 800 µL, 0.25 M), and alkene (0.800 mmol, 4.0 equiv), followed by a magnetic stir bar. The vial was sealed, removed from the glovebox, and placed in an oil bath preheated to 70 °C. After stirring for 18 h, the flask was removed from the bath and allowed to cool to room temperature. The reaction mixture was transferred to a glass scintillation vial using $CH_2Cl_2$ and was concentrated. The desired product was obtained via preparative thin layer chromatography or via flash silica chromatography, which is best for cyclopentanone products that decompose on silica over time.

Unless otherwise noted, the products were obtained at >20:1 dr.

#### 3.1. Products Synthesized via Conditions A and B

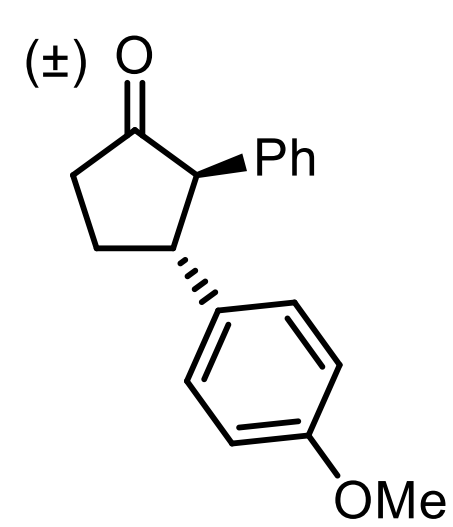


**3-(4-Methoxyphenyl)-2-phenylcyclopentan-1-one (3a).** General Procedure A was followed using 1-(dimethyl(oxo)-$\lambda^6$-sulfaneylidene)-3-phenylpropan-2-one (42.1 mg, 0.200 mmol, 1.0 equiv) as the sulfoxonium ylide input and 1-methoxy-4-vinylbenzene (0.107 g, 0.800 mmol, 4.0 equiv) as the alkene input. Preparative TLC (30% $Et_2O$ in hexanes) provided the title compound as a pale-yellow oil (38.4 mg, 72%). **$^1$H NMR** (500 MHz, $CDCl_3$): δ 7.28 (t, *J* = 7.5 Hz, 2H), 7.22 (t, *J* = 7.3 Hz, 1H), 7.11 (d, *J* = 8.7 Hz, 2H), 7.06 (d, *J* = 7.8 Hz, 2H), 6.83 (d, *J* = 8.7 Hz, 2H), 3.76 (s, 3H), 3.48 (td, *J* = 11.9, 5.3 Hz, 1H), 3.42 (d, *J* = 12.5 Hz, 1H), 2.73–2.65 (m, 1H), 2.53–2.42 (m, 2H), 2.14–2.03 (m, 1H). **$^{13}$C{$^1$H} NMR** (126 MHz, $CDCl_3$): δ 216.6, 158.4, 137.2, 133.7, 128.7, 128.5, 128.0, 127.0, 114.0, 63.0, 55.2, 49.7, 38.8, 29.6. The characterization data align with the reported literature values.[10]

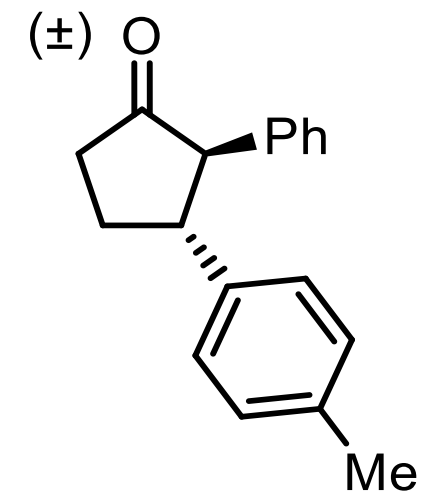

**2-Phenyl-3-(*p*-tolyl)cyclopentan-1-one (3b).** General Procedure B was followed using 1-(dimethyl(oxo)-$\lambda^6$-sulfaneylidene)-3-phenylpropan-2-one (42.1 mg, 0.200 mmol, 1.0 equiv) as the sulfoxonium ylide input and 1-methyl-4-vinylbenzene (0.0945 g, 0.800 mmol, 4.0 equiv) as the alkene input. Preparative TLC (20% EtOAc in hexanes) provided the title compound as a white crystalline solid (30.5 mg, 61%). **M.p.**: 90 °C. **IR** (neat): 3021, 2980, 2926, 2868, 1738, 1514, 1497, 1452, 1406, 1262, 1132, 1111, 1072, 1030, 918, 864, 806, 762, 731, 698, 648, 583, 554, 515 cm$^{-1}$. **$^1$H NMR** (500 MHz, $CDCl_3$): δ 7.27 (t, *J* = 7.6 Hz, 2H), 7.21 (t, *J* = 7.6 Hz, 1H), 7.17–7.00 (m, 6H), 3.56–3.43 (m, 2H), 2.76–2.65 (m, 1H), 2.54–2.44 (m, 2H), 2.31 (s, 3H), 2.15–2.03 (m, 1H). **$^{13}$C{$^1$H} NMR** (126 MHz, $CDCl_3$): δ 216.6, 138.6, 137.2, 136.4, 129.3, 128.7, 128.5, 127.0, 126.9, 62.8, 50.0, 38.8, 29.7, 21.0. **HRMS** (EI, m/z): [M]+ calc'd for $C_{18}H_{18}O$, 250.1352; found, 250.1355.

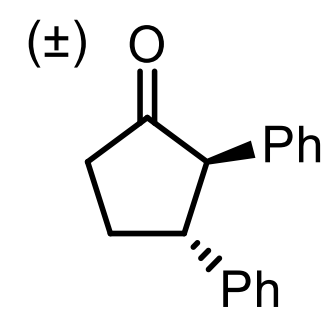

**2,3-Diphenylcyclopentan-1-one (3c)**. General Procedure B was followed using 1-(dimethyl(oxo)-$\lambda^6$-sulfaneylidene)-3-phenylpropan-2-one (42.1 mg, 0.200 mmol, 1.0 equiv) as the sulfoxonium ylide input and styrene (0.0833 g, 0.800 mmol, 4.0 equiv) as the alkene input. Preparative TLC (30% $Et_2O$ in hexanes) provided the title compound as a white crystalline solid (25.3 mg, 54%). **M.p.**: 94 °C (95 °C lit.).[11] **$^1$H NMR** (500 MHz, $CDCl_3$): δ 7.33–7.24 (m, 4H), 7.24–7.15 (m, 4H), 7.05 (d, *J* = 7.7 Hz, 2H), 3.60–3.43 (m, 2H), 2.79–2.66 (m, 1H), 2.58–2.43 (m, 2H), 2.20–2.04 (m, 1H). **$^{13}$C{$^1$H} NMR** (126 MHz, $CDCl_3$): δ 216.5, 141.6, 137.1, 128.7, 128.6, 128.5, 127.1, 127.0, 126.9, 62.8, 50.4, 38.8, 29.5. The characterization data align with the reported literature values.[12]

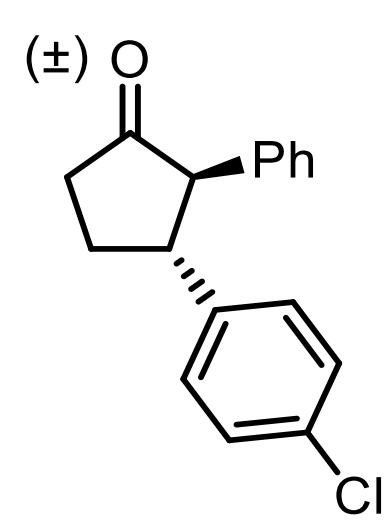

**3-(4-Chlorophenyl)-2-phenylcyclopentan-1-one (3d)**. General Procedure B was followed using 1-(dimethyl(oxo)-$\lambda^6$-sulfaneylidene)-3-phenylpropan-2-one (42.1 mg, 0.200 mmol, 1.0 equiv) as the sulfoxonium ylide input and 1-chloro-4-vinylbenzene (0.111 g, 0.800 mmol, 4.0 equiv) as the alkene input. Preparative TLC (30% $Et_2O$ in hexanes) provided the title compound as a yellow oil (24.2 mg, 45%). **IR** (neat): 3061, 3028, 2961, 2887, 1742, 1601, 1493, 1452, 1404, 1277, 1117, 1090, 1013, 909, 822, 729, 696, 646, 534, 517 cm$^{-1}$. **$^1$H NMR** (500 MHz, $CDCl_3$): δ 7.38–7.18 (m,

5H), 7.10 (d, *J* = 8.6 Hz, 2H), 7.02 (d, *J* = 8.6 Hz, 2H), 3.48 (td, *J* = 12.0, 5.4 Hz, 1H), 3.40 (d, *J* = 12.6 Hz, 1H), 2.77–2.65 (m, 1H), 2.55–2.42 (m, 2H), 2.16–2.02 (m, 1H). **$^{13}C\{^1H\}$ NMR** (126 MHz, $CDCl_3$): δ 215.9, 140.1, 136.7, 132.6, 128.8, 128.65, 128.63, 128.4, 127.2, 62.9, 49.9, 38.7, 29.3. **HRMS** (EI, m/z): [M]+ calc'd for $C_{17}H_{15}ClO$, 270.0806; found, 270.0809.

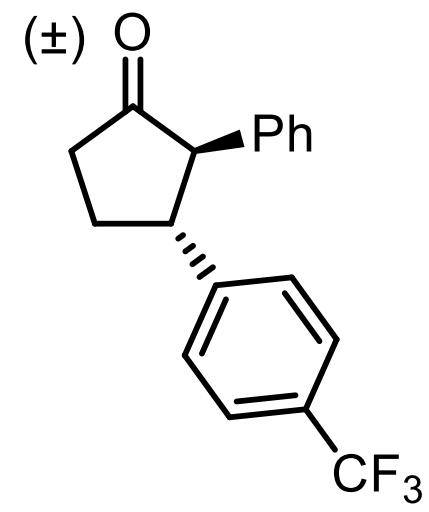


**2-Phenyl-3-(4-(trifluoromethyl)phenyl)cyclopentan-1-one (3e)**. General Procedure B was followed using 1-(dimethyl(oxo)-λ$^6$-sulfaneylidene)-3-phenylpropan-2-one (42.1 mg, 0.200 mmol, 1.0 equiv) as the sulfoxonium ylide input and 1-(trifluoromethyl)-4-vinylbenzene (0.138 g, 0.800 mmol, 4.0 equiv) as the alkene input. Flash silica column chromatography (40% $Et_2O$ in hexanes) provided the title compound as a clear oil (14.8 mg, 24%). **IR** (neat): 3032, 2963, 2918, 1736, 1618, 1497, 1422, 1402, 1323, 1155, 1103, 1067, 1015, 831, 768, 737, 698, 667, 604, 563, 517 $cm^{-1}$. **$^1H$ NMR** (500 MHz, $CDCl_3$): δ 7.53 (d, *J* = 8.3 Hz, 2H), 7.33–7.26 (m, 4H), 7.25–7.20 (m, 1H), 7.04 (d, *J* = 7.0 Hz, 2H), 3.59 (td, *J* = 12.3, 5.7 Hz, 1H), 3.46 (d, *J* = 12.6 Hz, 1H), 2.79–2.68 (m, 1H), 2.58–2.46 (m, 2H), 2.19–2.08 (m, 1H). **$^{13}C\{^1H\}$ NMR** (126 MHz, $CDCl_3$): δ 215.5, 145.7, 136.5, 129.2 (q, $2J_{CF}$ = 31.5), 128.7, 128.6, 127.4, 127.3, 125.6 (q, $3J_{CF}$ = 3.8), 124.1 (q, $1J_{CF}$ = 272), 62.7, 50.2, 38.6, 29.2. **$^{19}F$ NMR** (471 MHz, $CDCl_3$): δ -62.51 (s). **HRMS** (EI, m/z): [M]+ calc'd for, 304.1070; found, 304.1071.

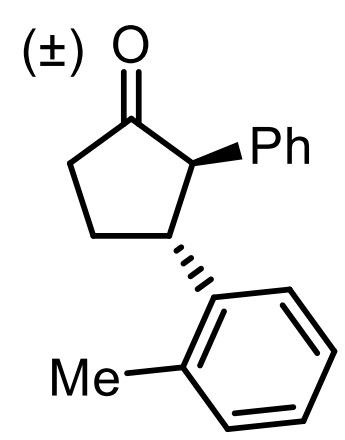


**2-Phenyl-3-(*o*-tolyl)cyclopentan-1-one (3f)**. General Procedure B was followed using 1-(dimethyl(oxo)-λ$^6$-sulfaneylidene)-3-phenylpropan-2-one (42.1 mg, 0.200 mmol, 1.0 equiv) as the sulfoxonium ylide input and 1-methyl-2-vinylbenzene (0.0945 g, 0.800 mmol, 4.0 equiv) as the alkene input. Preparative TLC (20% EtOAc in hexanes) provided the title compound as a white crystalline solid (22.7 mg, 45%). **M.p.**: 122 °C. **IR** (neat): 3063, 3017, 2955, 2932, 2095, 2872, 1736, 1603, 1495, 1452, 1395, 1281, 1140, 1119, 860, 768, 731, 696, 625, 557, 529 $cm^{-1}$. **$^1H$ NMR** (500 MHz, $CDCl_3$): δ 7.38 (d, *J* = 7.8 Hz, 1H), 7.31–7.15 (m, 4H), 7.15–7.06 (m, 2H), 7.01 (d, *J* = 6.8 Hz, 2H), 3.76 (td, *J* = 12.0, 5.9 Hz, 1H), 3.57 (d, *J* = 12.5 Hz, 1H), 2.73 (dd, *J* = 19.6, 8.0 Hz, 1H), 2.58–2.40 (m, 2H), 2.11 (s, 3H), 2.08–1.96 (m, 1H). **$^{13}C\{^1H\}$ NMR** (126 MHz, $CDCl_3$): δ 216.8, 139.7, 137.2, 136.3, 130.5, 128.6, 128.5, 127.0, 126.49, 126.47, 125.0, 62.6, 46.4, 38.9, 29.1, 19.5. **HRMS** (EI, m/z): [M]+ calc'd for, 250.1352; found, 250.1349.

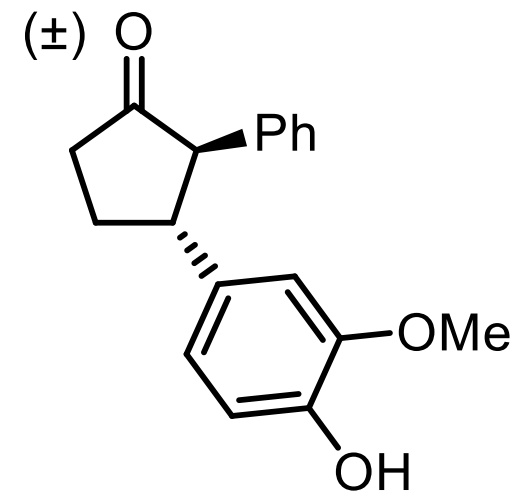

**3-(4-Hydroxy-3-methoxyphenyl)-4-methyl-2-phenylcyclopentan-1-one (3g)**. General Procedure A was followed using 1-(dimethyl(oxo)-λ$^6$-sulfaneylidene)-1,3-diphenylpropan-2-one (57.3 mg, 0.200 mmol, 1.0 equiv) as the sulfoxonium ylide input and 2-methoxy-4-vinylphenol (0.120 g, 0.800 mmol, 4.0 equiv) as the alkene input. Flash silica column chromatography (70% $Et_2O$ in hexanes) provided the title compound as a clear oil (25.7 mg, 46%). **IR** (neat): 3412, 3061, 3028, 2963, 2938, 2845, 1738, 1601, 1514, 1451, 1431, 1370, 1260, 1207, 1157, 1121, 1030, 910, 853, 804, 729, 696, 633, 552 cm$^{-1}$. **$^1$H NMR** (500 MHz, $CDCl_3$): δ 7.27 (t, *J* = 7.4 Hz, 2H), 7.21 (t, *J* = 7.3 Hz, 1H), 7.04 (d, *J* = 7.2 Hz, 2H), 6.82 (d, *J* = 8.2 Hz, 1H), 6.71 (d, *J* = 10.3 Hz, 1H), 6.56 (s, 1H), 5.50 (s, 1H), 3.75 (s, 3H), 3.44 (td, *J* = 11.9, 5.3 Hz, 1H), 3.37 (d, *J* = 12.5 Hz, 1H), 2.74–2.65 (m, 1H), 2.53–2.42 (m, 2H), 2.15–2.04 (m, 1H). **$^{13}$C{$^1$H} NMR** (126 MHz, $CDCl_3$): δ 216.7, 146.4, 144.4, 137.3, 133.5, 128.8, 128.6, 127.0, 119.4, 114.4, 109.8, 63.2, 55.8, 50.3, 38.8, 29.3. **HRMS** (EI, m/z): [M]+ calc'd for $C_{18}H_{18}O_3$, 282.1251; found, 282.1254.

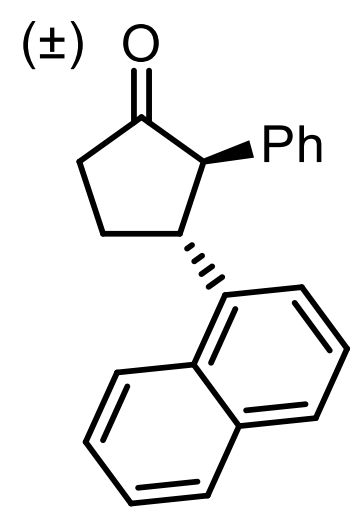

**3-(Naphthalen-1-yl)-2-phenylcyclopentan-1-one (3h)**. General Procedure B was followed using 1-(dimethyl(oxo)-λ$^6$-sulfaneylidene)-3-phenylpropan-2-one (42.1 mg, 0.200 mmol, 1.0 equiv) as the sulfoxonium ylide input and 1-vinylnapthalene (0.123 g, 0.800 mmol, 4.0 equiv) as the alkene input. Preparative TLC (40% $Et_2O$ in hexanes) provided the title compound as a white crystalline solid (34.3 mg, 60%). **M.p.**: 110 °C. **IR** (neat): 3038, 2949, 2916, 2878, 1728, 1597, 1499, 1398, 1279, 1254, 1223, 1171, 1134, 1105, 1076, 1017, 858, 802, 779, 758, 731, 698, 625, 569, 534 cm$^{-1}$. **$^1$H NMR** (400 MHz, $CDCl_3$): δ 8.09–8.02 (m, 1H), 7.89–7.82 (m, 1H), 7.73 (d, *J* = 8.1 Hz, 1H), 7.52–7.40 (m, 4H), 7.22 (t, *J* = 7.2 Hz, 2H), 7.15 (t, *J* = 8.2 Hz, 3H), 4.43 (td, *J* = 11.8, 5.9 Hz, 1H), 3.89 (d, *J* = 12.2 Hz, 1H), 2.80–2.70 (m, 2H), 2.66–2.56 (m, 1H), 2.09–1.98 (m, 1H). **$^{13}$C{$^1$H} NMR** (126 MHz, $CDCl_3$): δ 216.2, 137.5, 136.6, 134.0, 131.9, 129.0, 128.6, 128.5, 127.4, 127.0, 126.0, 125.6, 122.8, 122.4, 60.8, 44.8, 38.6, 30.2. **HRMS** (EI, m/z): [M]+ calc'd for $C_{21}H_{18}O$, 286.1352; found, 286.1352.

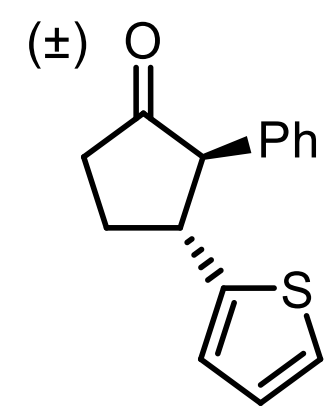

**2-Phenyl-3-(thiophen-2-yl)cyclopentan-1-one (3i)**. General Procedure B was followed using 1-(dimethyl(oxo)-λ$^6$-sulfaneylidene)-3-phenylpropan-2-one (42.1 mg, 0.200 mmol, 1.0 equiv) as the sulfoxonium ylide input and 2-vinylthiophene (0.0881 g, 0.800 mmol, 4.0 equiv) as the alkene input. Preparative TLC (70% $CH_2Cl_2$ in hexanes) provided the title compound as a pale-yellow crystalline solid (29.5 mg, 61%). **M.p.**: 110 °C. **IR** (neat): 3107, 3059, 3026, 2963, 2934, 2893, 2866, 1738, 1601, 1497, 1451, 1391, 1285, 1219, 1130, 1103, 1072, 1030, 984, 847, 829, 762, 698, 648, 557, 527 cm$^{-1}$. **$^1$H NMR** (400 MHz, $CDCl_3$): δ 7.34–7.27 (m, 2H), 7.27–7.21 (m, 1H), 7.12 (dd, *J* = 5.1, 1.2 Hz, 1H), 7.11–7.06 (m, 2H), 6.87 (dd, *J* = 5.1, 3.5 Hz, 1H), 6.73 (dt, *J* = 3.4, 1.0 Hz, 1H), 3.78 (td, *J* = 11.9, 5.9 Hz, 1H), 3.39 (d, *J* = 12.1 Hz, 1H), 2.72–2.57 (m, 2H), 2.54–2.42 (m, 1H), 2.22–2.10 (m, 1H). **$^{13}$C{$^1$H} NMR** (126 MHz, $CDCl_3$): δ 215.6, 145.7, 136.8, 128.8, 128.6, 127.3, 126.7, 124.0, 123.5, 64.1, 45.7, 38.7, 30.2. **HRMS** (EI, m/z): [M]+ calc'd for $C_{15}H_{14}OS$, 242.0760; found, 242.0758.

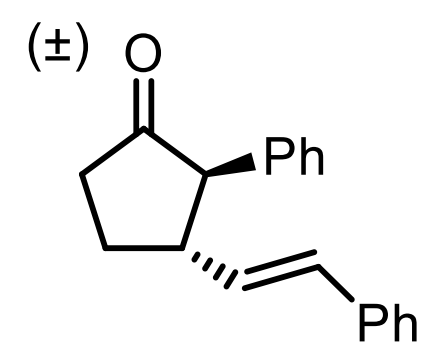

**2-Phenyl-3-((*E*)-styryl)cyclopentan-1-one (3j)**. General Procedure A was followed using 1-(dimethyl(oxo)-λ$^6$-sulfaneylidene)-3-phenylpropan-2-one (42.1 mg, 0.200 mmol, 1.0 equiv) as the sulfoxonium ylide input and (*E*)-buta-1,3-dien-1-ylbenzene (0.104 g, 0.800 mmol, 4.0 equiv) as the alkene input. Preparative TLC (30% $Et_2O$ in hexanes) provided the title compound as a clear oil (42.3 mg, 81%). **IR** (neat): 3082, 3059, 3026, 2961, 2930, 2882, 1738, 1599, 1578, 1493, 1449, 1404, 1275, 1263, 1144, 1128, 1070, 1028, 962, 908, 746, 729, 692, 559, 536 cm$^{-1}$. **$^1$H NMR** (400 MHz, $CDCl_3$): δ 7.38–7.18 (m, 8H), 7.15 (d, *J* = 8.5 Hz, 2H), 6.38 (d, *J* = 16.0 Hz, 1H), 6.22 (dd, *J* = 15.8, 6.8 Hz, 1H), 3.23–3.05 (m, 2H), 2.67–2.56 (m, 1H), 2.48–2.32 (m, 2H), 2.00–1.83 (m, 1H). **$^{13}$C{$^1$H} NMR** (126 MHz, $CDCl_3$): δ 216.6, 137.01, 136.98, 130.9, 130.8, 128.8, 128.7, 128.5, 127.4, 127.1, 126.2, 62.1, 48.2, 38.3, 27.8. **HRMS** (EI, m/z): [M]+ calc'd for $C_{19}H_{18}O$, 262.1352; found, 262.1350.

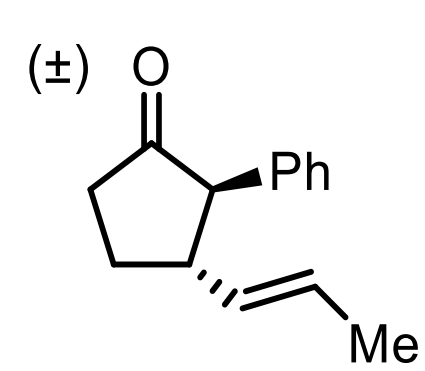

**2-Phenyl-3-((*E*)-prop-1-en-1-yl)cyclopentan-1-one (3k)**. General Procedure B was followed using 1-(dimethyl(oxo)-λ$^6$-sulfaneylidene)-3-phenylpropan-2-one (42.1 mg, 0.200 mmol, 1.0 equiv) as the sulfoxonium ylide input and (*E*)-penta-1,3-diene (0.0545 g, 0.800 mmol, 4.0 equiv) as the alkene input. Preparative TLC (20% $Et_2O$ in hexanes) provided the title compound as a clear oil (18.0 mg, 45%). **IR** (neat): 3030, 2961, 2934, 2878, 1742, 1603, 1497, 1451, 1404, 1275, 1152, 1090, 963, 756, 698, 552 cm$^{-1}$. **$^1$H NMR** (400 MHz, $CD_3CN$): δ 7.35–7.23 (m, 3H), 7.12 (d, *J* = 6.9

Hz, 2H), 5.56–5.33 (m, 2H), 3.11 (d, *J* = 12.1 Hz, 1H), 3.00–2.87 (m, 1H), 2.47–2.40 (m, 1H), 2.38–2.29 (m, 1H), 2.23–2.16 (m, 1H), 1.82–1.70 (m, 1H), 1.58 (d, *J* = 5.5 Hz, 3H). **$^{13}C\{^1H\}$ NMR** (126 MHz, $CD_3CN$): δ 216.8, 138.4, 132.8, 129.1, 128.3, 126.7, 125.8, 61.8, 47.8, 37.7, 27.8, 17.0. **HRMS** (EI, m/z): [M]+ calc'd for $C_{14}H_{16}O$, 200.1196; found, 200.1194.

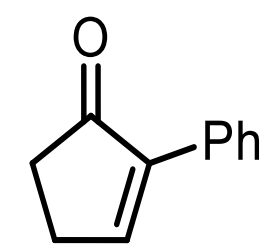

**2-Phenylcyclopent-2-en-1-one (3l).** General Procedure A was followed using 1-(dimethyl(oxo)-λ$^6$-sulfaneylidene)-3-phenylpropan-2-one (42.1 mg, 0.200 mmol, 1.0 equiv) as the sulfoxonium ylide input and (vinyloxy)benzene (0.0961 g, 0.800 mmol, 4.0 equiv) as the alkene input. Preparative TLC (30% $Et_2O$ in hexanes) provided the title compound as a white crystalline solid (9.9 mg, 31%). **M.p.**: 69 °C (68 °C lit.).[13] **$^1H$ NMR** (500 MHz, $CDCl_3$): δ 7.82 (t, *J* = 2.9 Hz, 1H), 7.68 (d, *J* = 7.1 Hz, 2H), 7.38 (t, *J* = 7.3 Hz, 2H), 7.33 (t, *J* = 7.3 Hz, 1H), 2.73–2.70 (m, 2H), 2.62–2.59 (m, 2H). **$^{13}C\{^1H\}$ NMR** (126 MHz, $CDCl_3$): δ 207.6, 158.9, 143.5, 131.7, 128.4, 128.3, 127.0, 35.8, 26.2. The characterization data align with the reported literature values.[13]

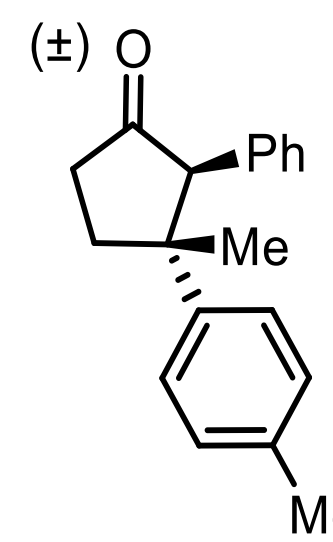

**3-Methyl-2-phenyl-3-(*p*-tolyl)cyclopentan-1-one (3m).** General Procedure B was followed using 1-(dimethyl(oxo)-λ$^6$-sulfaneylidene)-1,3-diphenylpropan-2-one (57.3 mg, 0.200 mmol, 1.0 equiv) as the sulfoxonium ylide input and 1-methyl-4-(prop-1-en-2-yl)benzene (0.106 g, 0.800 mmol, 4.0 equiv) as the alkene input. Preparative TLC (20% $Et_2O$ in hexanes) provided the title compound as a white crystalline solid (31.7 mg, 60%, 90:10 dr). COSY and 1D selective NOE confirmed the relative configuration of the major diastereomer (see Section 6). **M.p.**: 107 °C. **IR** (neat): 3061, 3021, 2955, 2913, 2876, 1722, 1499, 1454, 1414, 1294, 1128, 1074, 818, 772, 741, 698, 596, 579, 523 $cm^{-1}$. **$^1H$ NMR** (400 MHz, $CDCl_3$): δ 7.26–7.10 (m, 7H), 6.88–6.79 (m, 2H), 3.76 (s, 1H), 2.69–2.53 (m, 3H), 2.34 (s, 3H), 2.17–2.10 (m, 1H), 1.20 (s, 3H). **$^{13}C\{^1H\}$ NMR** (126 MHz, $CDCl_3$): δ 217.0, 143.4, 135.9, 135.0, 130.4, 129.0, 127.9, 127.0, 126.0, 68.0, 47.7, 36.1, 34.9, 21.5, 20.9. **HRMS** (EI, m/z): [M]+ calc'd for $C_{19}H_{20}O$, 264.1509; found, 264.1509.

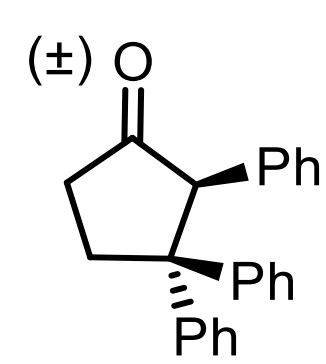

**2,3,3-Triphenylcyclopentan-1-one (3n)**. General Procedure B was followed using 1-(dimethyl(oxo)-λ$^6$-sulfaneylidene)-3-phenylpropan-2-one (42.1 mg, 0.200 mmol, 1.0 equiv) as the sulfoxonium ylide input and ethene-1,1-diyldibenzene (0.144 g, 0.800 mmol, 4.0 equiv) as the alkene input. Preparative TLC (10% $Et_2O$ in hexanes) provided the title compound as a pale-yellow crystalline solid (37.4 mg, 60%). **M.p.**: 174 °C. **IR** (neat): 3065, 3026, 2951, 2918, 2853,

1732, 1597, 1578, 1489, 1441, 1398, 1242, 1138, 1082, 1030, 984, 907, 837, 774, 762, 696, 656, 592, 509 $cm^{-1}$. **$^1$H NMR** (500 MHz, $CDCl_3$): δ 7.38–7.27 (m, 4H), 7.23 (t, $J$ = 6.9 Hz, 1H), 7.20–6.99 (m, 6H), 6.93–6.84 (m, 2H), 6.77–6.71 (m, 2H), 4.46 (s, 1H), 3.23–3.10 (m, 1H), 2.70–2.60 (m, 1H), 2.58–2.48 (m, 2H). **$^{13}$C{$^1$H} NMR** (126 MHz, $CDCl_3$): δ 217.9, 148.3, 143.6, 136.4, 130.3, 128.9, 128.4, 127.8, 127.7, 127.3, 126.8, 126.5, 126.3, 64.2, 57.5, 36.6, 34.1. **HRMS** (EI, m/z): [M]+ calc'd for $C_{23}H_{20}O$, 312.1509; found, 312.1510.

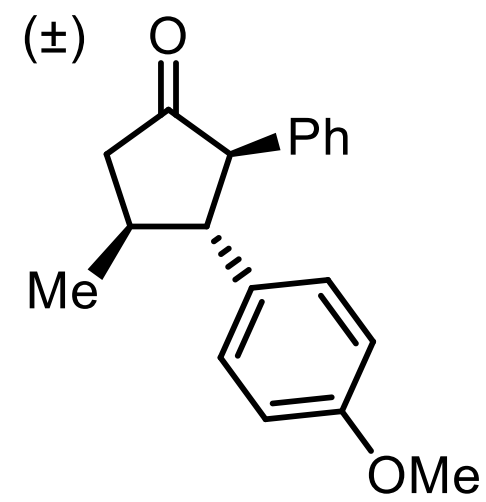


**3-(4-Methoxyphenyl)-4-methyl-2-phenylcyclopentan-1-one (3o).** General Procedure A was followed using 1-(dimethyl(oxo)-λ$^6$-sulfaneylidene)-3-phenylpropan-2-one (42.1 mg, 0.200 mmol, 1.0 equiv) as the sulfoxonium ylide input and (*E*)-1-methoxy-4-(prop-1-en-1-yl)benzene (trans-anethole, 0.119 g, 0.800 mmol, 4.0 equiv) as the alkene input. Preparative TLC (20% $Et_2O$ in hexanes) provided the title compound as a white crystalline solid (44.0 mg, 79%). COSY and 1D selective NOE confirmed the relative configuration (see Section 6). **M.p.**: 80 °C. **IR** (neat): 2957, 2926, 2837, 1732, 1609, 1584, 1512, 1454, 1402, 1371, 1306, 1287, 1244, 1177, 1144, 1107, 1024, 829, 7733, 739, 696, 650, 571, 540, 523 $cm^{-1}$. **$^1$H NMR** (500 MHz, $CDCl_3$): δ 7.24 (t, $J$ = 7.0 Hz, 2H), 7.18 (t, $J$ = 6.7 Hz, 1H), 7.09 (d, $J$ = 8.7 Hz, 2H), 7.00 (d, $J$ = 8.8 Hz, 2H), 6.83 (d, $J$ = 6.6 Hz, 2H), 3.76 (s, 3H), 3.53 (d, $J$ = 12.5 Hz, 1H), 2.93 (t, $J$ = 11.7 Hz, 1H), 2.84 (dd, $J$ = 18.6, 7.3 Hz, 1H), 2.40 (m, 1H), 2.15 (dd, $J$ = 18.5, 13.9 Hz, 1H), 1.10 (d, $J$ = 6.4 Hz, 3H). **$^{13}$C{$^1$H} NMR** (126 MHz, $CDCl_3$): δ 215.9, 158.5, 137.2, 132.0, 128.6, 128.5, 128.4, 126.9, 114.0, 64.5, 58.3, 55.2, 47.2, 37.0, 17.9. **HRMS** (EI, m/z): [M]+ calc'd for $C_{19}H_{20}O_2$, 280.1458; found, 280.1455.

For a 1 mmol scale reaction, General Procedure A was followed at a larger scale using 1-(dimethyl(oxo)-λ$^6$-sulfaneylidene)-3-phenylpropan-2-one (0.210 g, 1.00 mmol, 1.0 equiv), HFIP (4.00 mL, 0.25 M), and (*E*)-1-methoxy-4-(prop-1-en-1-yl)benzene (trans-anethole, 0.593 g, 4.00 mmol, 4.0 equiv) in a 10–20 mL Biotage® microwave vial. The desired product was initially isolated via flash column chromatography (30% $Et_2O$ in hexanes), and its presence was tracked using UV light and $KMNO_4$ stain on TLC plates. Impure fractions from the column chromatography were repurified by preparative TLC (10% $Et_2O$ in hexanes). The desired compound was obtained as a white crystalline solid (214.1 mg, 76%). The characterization data align with the results reported above.

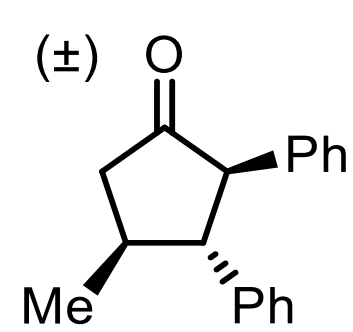


**4-Methyl-2,3-diphenylcyclopentan-1-one (3p)**. General Procedure B was followed using 1-(dimethyl(oxo)-λ$^6$-sulfaneylidene)-3-phenylpropan-2-one (42.1 mg, 0.200 mmol, 1.0 equiv) as the sulfoxonium ylide input and (*E*)-prop-1-en-1-ylbenzene (0.0945 g, 0.800 mmol, 4.0 equiv) as the alkene input. Preparative TLC (30% $Et_2O$ in hexanes) provided the title compound as a white crystalline solid (24.8 mg, 50%). **M.p.**: 99 °C. **IR** (neat): 3024, 2969, 2932, 2878, 1732, 1601,

1495, 1451, 1406, 1275, 1157, 1140, 1071, 1030, 756, 741, 696, 625, 577, 507 cm$^{-1}$. **$^{1}$H NMR** (400 MHz, $CDCl_3$): δ 7.32–7.13 (m, 8H), 7.00 (d, *J* = 6.9 Hz, 2H), 3.58 (d, *J* = 12.5 Hz, 1H), 2.99 (t, *J* = 10.8 Hz, 1H), 2.85 (dd, *J* = 18.5, 7.3 Hz, 1H), 2.53–2.38 (m, 1H), 2.17 (dd, *J* = 18.5, 11.8 Hz, 1H), 1.10 (d, *J* = 6.5 Hz, 3H). **$^{13}$C{$^{1}$H} NMR** (126 MHz, $CDCl_3$): δ 215.7, 140.1, 137.1, 128.60, 128.56, 128.47, 127.6, 126.98, 126.95 64.4, 59.0, 47.2, 36.9, 17.9. **HRMS** (EI, m/z): [M]+ calc'd for $C_{18}H_{18}O$, 250.1352; found, 250.1353.

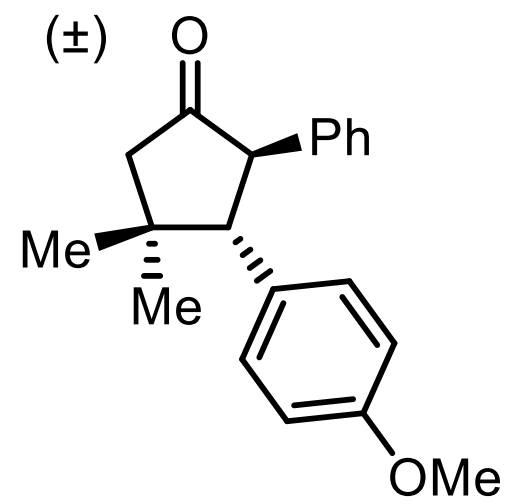


**3-(4-Methoxyphenyl)-4,4-dimethyl-2-phenylcyclopentan-1-one (3q)**. General Procedure B was followed using 1-(dimethyl(oxo)-λ$^{6}$-sulfaneylidene)-3-phenylpropan-2-one (42.1 mg, 0.200 mmol, 1.0 equiv) as the sulfoxonium ylide input and 1-methoxy-4-(2-methylprop-1-en-1-yl)benzene (0.130 g, 0.800 mmol, 4.0 equiv) as the alkene input. Preparative TLC (30% $Et_2O$ in hexanes) provided the title compound as a white crystalline solid (18.4 mg, 31%). **M.p.**: 97 °C. **IR** (neat): 3030, 2959, 2930, 2835, 1738, 1612, 1512, 1454, 1290, 1250, 1177, 1107, 1028, 831, 795, 750, 702, 581, 552, 527 cm$^{-1}$. **$^{1}$H NMR** (500 MHz, $CDCl_3$): δ 7.25–7.20 (m, 2H), 7.18–7.09 (m, 5H), 6.84 (d, *J* = 8.4 Hz, 2H), 3.92 (d, *J* = 13.2 Hz, 1H), 3.77 (s, 3H), 3.37 (d, *J* = 13.2 Hz, 1H), 2.56–2.40 (m, 2H), 1.21 (s, 3H), 0.90 (s, 3H). **$^{13}$C{$^{1}$H} NMR** (126 MHz, $CDCl_3$): δ 216.3, 158.5, 137.5, 129.7, 128.6, 128.5, 128.4, 126.9, 113.5, 59.5, 57.8, 55.1, 54.9, 38.0, 27.9, 22.8. **HRMS** (EI, m/z): [M]+ calc'd for, 294.1614; found, 294.1616.

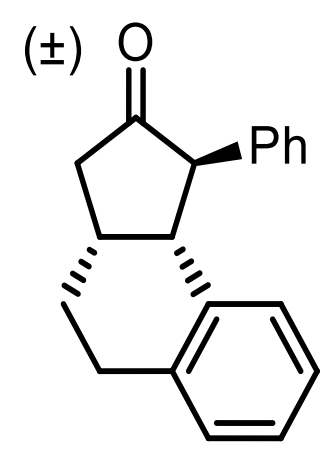


**1-Phenyl-1,3,3a,4,5,9b-hexahydro-2*H*-cyclopenta[*a*]naphthalen-2-one (3r)**. General Procedure B was followed using 1-(dimethyl(oxo)-λ$^{6}$-sulfaneylidene)-3-phenylpropan-2-one (42.1 mg, 0.200 mmol, 1.0 equiv) as the sulfoxonium ylide input and 1,2-dihydronaphthalene (0.104 g, 0.800 mmol, 4.0 equiv) as the alkene input. Preparative TLC (20% $Et_2O$ in hexanes) provided the title compound as a pale-yellow crystalline solid (21.6 mg, 41%). **M.p.**: 147 °C. **IR** (neat): 3019, 2918, 2897, 2897, 2857, 1730, 1599, 1487, 1451, 1439, 1402, 1240, 1105, 1026, 764, 739, 702, 623, 550, 513 cm$^{-1}$. **$^{1}$H NMR** (400 MHz, $CDCl_3$): δ 7.39–7.26 (m, 3H), 7.16–7.04 (m, 4H), 6.89 (t, *J* = 7.3 Hz, 1H), 6.40 (d, *J* = 7.7 Hz, 1H), 3.62 (dd, *J* = 11.5, 6.3 Hz, 1H), 3.31 (d, *J* = 11.6 Hz, 1H), 2.99–2.87 (m, 2H), 2.81 (dd, *J* = 18.6, 8.2 Hz, 1H), 2.74–2.64 (m, 1H), 2.45 (d, *J* = 18.6 Hz, 1H), 2.00–1.89 (m, 1H), 1.77–1.63 (m, 1H). **$^{13}$C{$^{1}$H} NMR** (126 MHz, $CDCl_3$): δ 217.7, 138.8, 136.7, 135.4, 129.4, 129.3, 129.1, 128.8, 127.1, 126.5, 125.6, 61.6, 47.4, 46.3, 33.2, 29.3, 26.3. **HRMS** (EI, m/z): [M]+ calc'd for $C_{19}H_{18}O$, 262.1352; found, 262.1355.

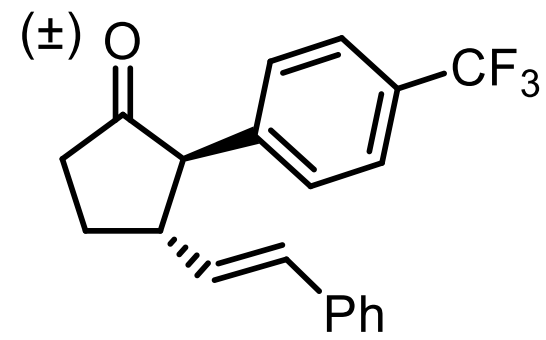


**3-((*E*)-Styryl)-2-(4-(trifluoromethyl)phenyl)cyclopentan-1-one (3s)**. General Procedure A was followed using 1-(dimethyl(oxo)-λ$^6$-sulfaneylidene)-3-(4-(trifluoromethyl)phenyl)propan-2-one (55.7 mg, 0.200 mmol, 1.0 equiv) as the sulfoxonium ylide input and (*E*)-buta-1,3-dien-1-ylbenzene (0.104 g, 0.800 mmol, 4.0 equiv) as the alkene input. Preparative TLC (30% $Et_2O$ in hexanes) provided the title compound as a clear oil (51.0 mg, 77%). **IR** (neat): 3028, 2970, 2884, 1742, 1618, 1493, 1418, 1323, 1163, 1119, 1067, 1018, 964, 880, 822, 754, 694, 606, 509 cm$^{-1}$. **$^1$H NMR** (500 MHz, $CDCl_3$): δ 7.60 (d, *J* = 8.2 Hz, 2H), 7.36–7.20 (m, 7H), 6.38 (d, *J* = 15.9 Hz, 1H), 6.20 (dd, *J* = 15.9, 7.3 Hz, 1H), 3.27 (d, *J* = 12.2 Hz, 1H), 3.19–3.07 (m, 1H), 2.71–2.59 (m, 1H), 2.50–2.35 (m, 2H), 2.02–1.87 (m, 1H). **$^{13}$C{$^1$H} NMR** (126 MHz, $CDCl_3$): δ 215.4, 140.9, 136.7, 131.4, 130.1, 129.3 (q, $2J_{CF}$ = 32.8 Hz), 129.2, 128.6, 127.6, 126.2, 125.6 (q, $3J_{CF}$ = 3.8 Hz), 124.2 (q, $1J_{CF}$ = 272 Hz), 61.8, 48.2, 38.1, 27.9. **$^{19}$F NMR** (471 MHz, $CDCl_3$): δ -62.51 (s). **HRMS** (EI, m/z): [M]+ calc'd for $C_{20}H_{17}F_3O$, 330.1226; found, 330.1226.

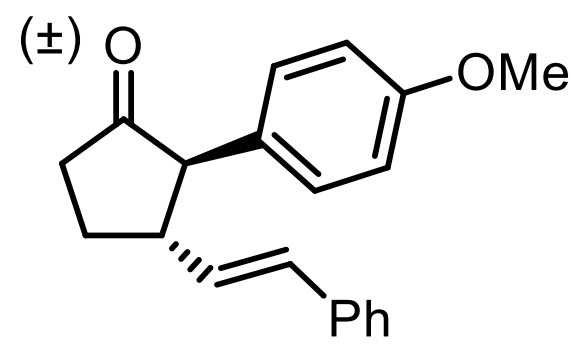


**2-(4-Methoxyphenyl)-3-((*E*)-styryl)cyclopentan-1-one (3t)**. General Procedure A was followed using 1-(dimethyl(oxo)-λ$^6$-sulfaneylidene)-3-(4-methoxyphenyl)propan-2-one (48.1 mg, 0.200 mmol, 1.0 equiv) as the sulfoxonium ylide input and (*E*)-buta-1,3-dien-1-ylbenzene (0.104 g, 0.800 mmol, 4.0 equiv) as the alkene input. Preparative TLC (30% $Et_2O$ in hexanes) provided the title compound as a yellow oil (30.3 mg, 52%). **IR** (neat): 3059, 3024, 2957, 2932, 2835, 1740, 1613, 1512, 1449, 1246, 1179, 1146, 1084, 1030, 964, 910, 868, 818, 750, 694, 540 cm$^{-1}$. **$^1$H NMR** (500 MHz, $CDCl_3$): δ 7.33–7.26 (m, 4H), 7.21 (t, *J* = 8.2 Hz, 1H), 7.06 (d, *J* = 8.7 Hz, 2H), 6.88 (d, *J* = 8.8 Hz, 2H), 6.38 (d, *J* = 15.8 Hz, 1H), 6.21 (dd, *J* = 15.9, 7.1 Hz, 1H), 3.78 (s, 3H), 3.13 (d, *J* = 12.1 Hz, 1H), 3.11–3.00 (m, 1H), 2.65–2.57 (m, 1H), 2.45–2.33 (m, 2H), 1.94–1.86 (m, 1H). **$^{13}$C{$^1$H} NMR** (126 MHz, $CDCl_3$): δ 217.0, 158.6, 137.0, 130.9, 130.8, 129.7, 129.0, 128.5, 127.4, 126.2, 114.2, 61.4, 55.2, 48.3, 38.1, 27.7. **HRMS** (EI, m/z): [M]+ calc'd for $C_{20}H_{20}O_2$, 292.1458; found, 292.1463.

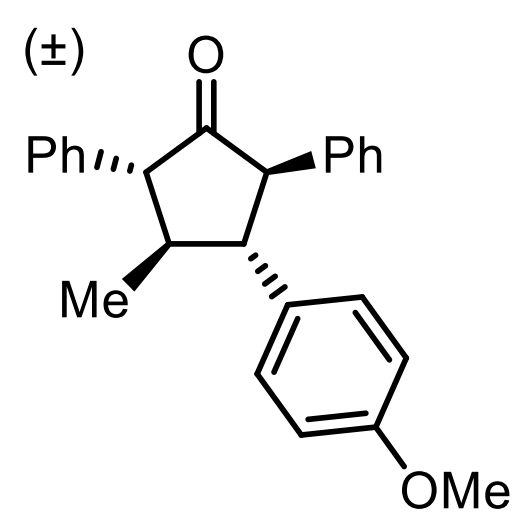


**3-(4-Methoxyphenyl)-4-methyl-2,5-diphenylcyclopentan-1-one (3u).** General Procedure A was followed using 1-(dimethyl(oxo)-λ$^6$-sulfaneylidene)-1,3-diphenylpropan-2-one (57.3 mg, 0.200 mmol, 1.0 equiv) as the sulfoxonium ylide input and (*E*)-1-methoxy-4-(prop-1-en-1-yl)benzene (trans-anethole, 0.119 g, 0.800 mmol, 4.0 equiv) as the alkene input. Preparative TLC

(40% $Et_2O$ in hexanes) provided the title compound as a yellow crystalline solid (32.3 mg, 50%). COSY and 1D selective NOE confirmed the relative configuration (see Section 6). **M.p.**: 168 °C. **IR** (neat): 3032, 2957, 2860, 1734, 1613, 1512, 1452, 1242, 1177, 1134, 1028, 827, 747, 700, 584, 523 $cm^{-1}$. **$^1$H NMR** (500 MHz, $CDCl_3$): δ 7.40 (t, *J* = 7.5 Hz, 2H), 7.34–7.19 (m, 6H), 7.15 (d, *J* = 8.2 Hz, 2H), 7.07 (d, *J* = 7.5 Hz, 2H), 6.86 (d, *J* = 8.2 Hz, 2H), 3.78 (s, 3H), 3.66 (d, *J* = 12.1 Hz, 1H), 3.23 (d, *J* = 12.0 Hz, 1H), 3.05 (t, *J* = 11.5 Hz, 1H), 2.51 (tq, *J* = 12.3, 6.5 Hz, 1H), 1.08 (d, *J* = 6.3 Hz, 3H). **$^{13}$C{$^1$H} NMR** (126 MHz, $CDCl_3$): δ 214.3, 158.6, 137.5, 137.4, 131.8, 128.8, 128.7, 128.6, 128.53, 128.50, 127.2, 127.0, 114.1, 64.7, 64.1, 56.2, 55.2, 44.9, 16.5. **HRMS** (EI, m/z): [M]+ calc'd for $C_{25}H_{24}O_2$, 356.1771; found, 356.1768.

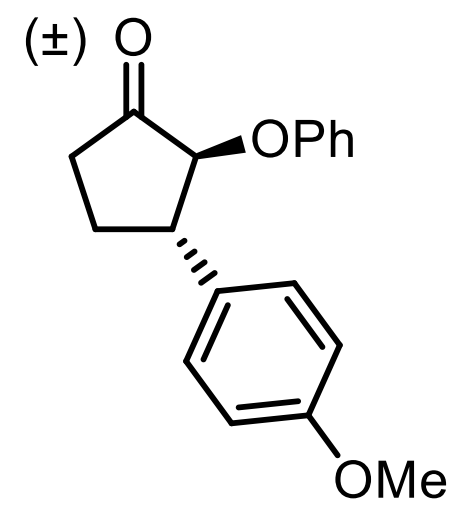


**3-(4-Methoxyphenyl)-2-phenoxycyclopentan-1-one (3v)**. General Procedure A was followed using 1-(dimethyl(oxo)-λ$^6$-sulfaneylidene)-3-phenoxypropan-2-one (45.3 mg, 0.200 mmol, 1.0 equiv) as the sulfoxonium ylide input and 1-methoxy-4-vinylbenzene (0.107 g, 0.800 mmol, 4.0 equiv) as the alkene input. Preparative TLC (40% $Et_2O$ in hexanes) provided the title compound as a clear oil (18.7 mg, 33%). **IR** (neat): 3061, 3003, 2959, 2907, 2837, 1751, 1587, 1514, 1491, 1456, 1242, 1179, 1146, 1094, 1030, 828, 752, 691, 575, 505 $cm^{-1}$. **$^1$H NMR** (500 MHz, $CDCl_3$): 7.25 (d, *J* = 8.6 Hz, 2H), 7.20 (t, *J* = 8.1 Hz, 2H), 6.94 (t, *J* = 7.4 Hz, 1H), 6.91–6.82 (m, 4H), 4.64 (d, *J* = 11.2 Hz, 1H), 3.78 (s, 3H), 3.45 (td, *J* = 11.9, 6.5 Hz, 1H), 2.66–2.55 (m, 1H), 2.51–2.38 (m, 2H), 2.15–2.05 (m, 1H). **$^{13}$C{$^1$H} NMR** (126 MHz, $CDCl_3$): δ 213.6, 158.7, 158.6, 132.4, 129.3, 128.0, 121.9, 116.3, 114.1, 86.5, 55.3, 46.6, 35.1, 24.5. **HRMS** (EI, m/z): [M]+ calc'd for $C_{18}H_{18}O_3$, 282.1251; found, 282.1254.

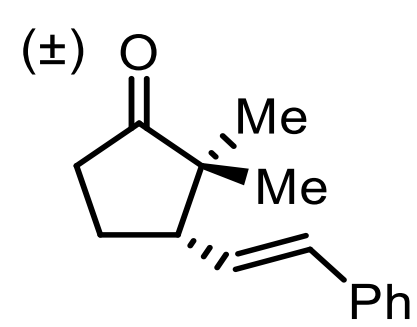


**(*E*)-2,2-Dimethyl-3-styrylcyclopentan-1-one (3w)**. General Procedure A was followed using 1-(dimethyl(oxo)-λ$^6$-sulfaneylidene)-3-methylbutan-2-one (32.4 mg, 0.200 mmol, 1.0 equiv) as the sulfoxonium ylide input and (*E*)-buta-1,3-dien-1-ylbenzene (0.104 g, 0.800 mmol, 4.0 equiv) as the alkene input. Preparative TLC (70% $CH_2Cl_2$ in hexanes) provided the title compound as a clear oil (18.5 mg, 43%). **IR** (neat): 3059, 3026, 2963, 2926, 2870, 1734, 1599, 1495, 1460, 1406, 1379, 1362, 1287, 1252, 1198, 1090, 1057, 968, 797, 748, 694, 571, 525 $cm^{-1}$. **$^1$H NMR** (500 MHz, $CDCl_3$): δ 7.38 (d, *J* = 8.1 Hz, 2H), 7.32 (t, *J* = 7.6 Hz, 2H), 7.23 (t, *J* = 7.3 Hz, 1H), 6.48 (d, *J* = 15.8 Hz, 1H), 6.19 (dd, *J* = 15.8, 8.2 Hz, 1H), 2.58 (dt, *J* = 11.2, 7.2 Hz, 1H), 2.47 (dd, *J* = 19.1, 8.7 Hz, 1H), 2.31–2.19 (m, 1H), 2.17–2.08 (m, 1H), 1.94–1.82 (m, 1H), 1.08 (s, 3H), 0.90 (s, 3H). **$^{13}$C{$^1$H} NMR** (126 MHz, $CDCl_3$): δ 222.7, 137.2, 131.8, 128.8, 128.6, 127.4, 126.2, 51.2, 49.0, 36.2, 25.0, 22.4, 18.5. **HRMS** (EI, m/z): [M]+ calc'd for $C_{15}H_{18}O$, 214.1352; found, 214.1353.

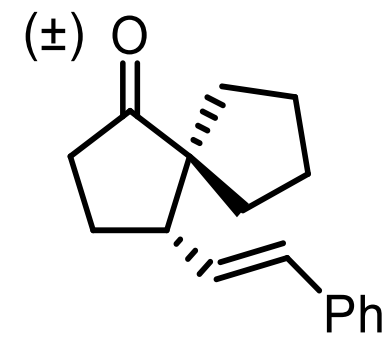

**(*E*)-4-Styrylspiro[4.4]nonan-1-one (3x).** General Procedure A was followed using 1-cyclopentyl-2-(dimethyl(oxo)-$\lambda^6$-sulfaneylidene)ethan-1-one (37.7 mg, 0.200 mmol, 1.0 equiv) as the sulfoxonium ylide input and (*E*)-buta-1,3-dien-1-ylbenzene (0.104 g, 0.800 mmol, 4.0 equiv) as the alkene input. Preparative TLC (70% $CH_2Cl_2$ in hexanes) provided the title compound as a pale-yellow oil (19.3 mg, 40%). **IR** (neat): 3026, 2951, 2864, 1730, 1599, 1493, 1449, 1406, 1221, 1138, 1071, 964, 748, 692, 521 $cm^{-1}$. **$^1$H NMR** (400 MHz, $CDCl_3$): δ 7.40–7.26 (m, 4H), 7.22 (t, *J* = 7.2 Hz, 1H), 6.47 (d, *J* = 15.8 Hz, 1H), 6.20 (dd, *J* = 15.8, 8.1 Hz, 1H), 2.73–2.62 (m, 1H), 2.44 (ddd, *J* = 19.0, 8.9, 3.3 Hz, 1H), 2.32–2.19 (m, 1H), 2.18–2.06 (m, 1H), 2.01–1.90 (m, 1H), 1.90–1.78 (m, 1H), 1.75–1.46 (m, 7H). **$^{13}$C{$^1$H} NMR** (126 MHz, $CDCl_3$): δ 222.5, 137.2, 131.6, 129.1, 128.6, 127.4, 126.1, 60.2, 50.5, 35.8, 34.3, 30.9, 26.34, 26.29, 25.8. **HRMS** (EI, m/z): [M]+ calc'd for $C_{17}H_{20}O$, 240.1509; found, 240.1512.

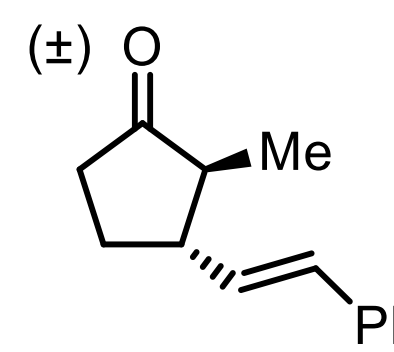

**2-Methyl-3-((*E*)-styryl)cyclopentan-1-one (3y)**. General Procedure A was followed using 1-(dimethyl(oxo)-$\lambda^6$-sulfaneylidene)butan-2-one (29.6 mg, 0.200 mmol, 1.0 equiv) as the sulfoxonium ylide input and (*E*)-buta-1,3-dien-1-ylbenzene (0.104 g, 0.800 mmol, 4.0 equiv) as the alkene input. Flash silica column chromatography (10% EtOAc in hexanes + 1% triethylamine) provided the title compound as a clear oil (12.4 mg, 31%, 95:5 dr). **IR** (neat): 3026, 2963, 2930, 2872, 1736, 1493, 1451, 1406, 1277, 1159, 1055, 964, 822, 750, 692, 523 $cm^{-1}$. **$^1$H NMR** (400 MHz, $CD_3CN$): δ 7.44 (d, *J* = 7.2 Hz, 2H), 7.35 (t, *J* = 7.7 Hz, 2H), 7.26 (t, *J* = 7.3 Hz, 1H), 6.55 (d, *J* = 15.9 Hz, 1H), 6.31 (dd, *J* = 15.8, 8.1 Hz, 1H), 2.51–2.43 (m, 1H), 2.40–2.33 (m, 1H), 2.21–2.12 (m, 2H), 2.05–1.97 (m, 1H), 1.82–1.72 (m, 1H), 1.04 (d, *J* = 7.0 Hz, 3H). **$^{13}$C{$^1$H} NMR** (126 MHz, $CD_3CN$): δ 219.1, 137.5, 132.7, 130.1, 128.6, 127.2, 126.0, 49.6, 48.6, 36.6, 27.4, 11.4. **HRMS** (EI, m/z): [M]+ calc'd for $C_{14}H_{16}O$, 200.1196; found, 200.1193.

### 3.2 Unsuccessful Substrates

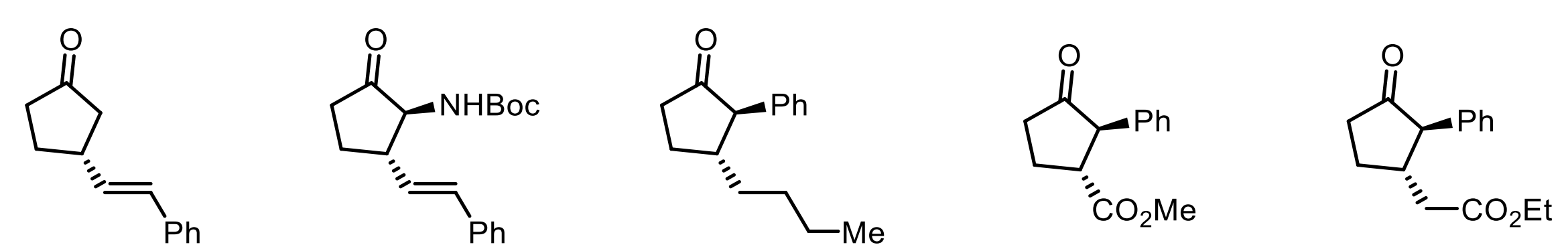

Conditions A: 0% | Conditions A: 16% | Conditions A: <15% | Conditions A/B: 0% | Conditions A/B: 16%

# 4. Mechanistic Experiments

## 4.1 Using Trans/Cis-Anetholes

**Figure S1.** Diastereomeric ratios obtained with trans- and cis-anethole inputs.

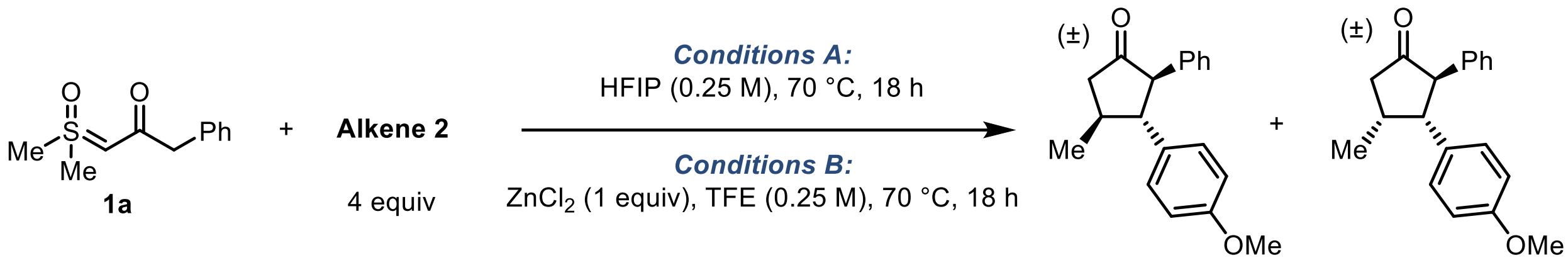


| | Alkene | | 3o | 4 |
|---|---|---|---|---|
| 2o | 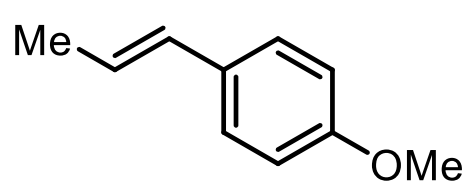 | ***Conditions A*** | 79% | Not Detected |
| | | ***Conditions B*** | 62% | Not Detected |
| 2s | 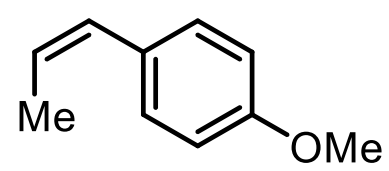 | ***Conditions A*** | 25% | 16% |
| | | ***Conditions B*** | 47% | 29% |

<u>Synthesis of Cyclopentanone with Trans-Anethole: Conditions A</u>

See Section 3.1 for compound **3o**. A single diastereomer was obtained as a white solid (79%).

<u>Synthesis of Cyclopentanone with Trans-Anethole: Conditions B</u>

General Procedure B was followed using 1-(dimethyl(oxo)-$\lambda^6$-sulfaneylidene)-3-phenylpropan-2-one (21.0 mg, 0.100 mmol, 1.0 equiv) as the sulfoxonium ylide input and (*E*)-1-methoxy-4-(prop-1-en-1-yl)benzene (trans-anethole, 59.3 mg, 0.400 mmol, 4.0 equiv) as the alkene input. Preparative TLC (20% $Et_2O$ in hexanes) provided a single diastereomer as a white solid (17.4 mg, 62%).

<u>Synthesis of Cyclopentanone with Cis-Anethole: Conditions A</u>

General Procedure A was followed using 1-(dimethyl(oxo)-$\lambda^6$-sulfaneylidene)-3-phenylpropan-2-one (21.0 mg, 0.100 mmol, 1.0 equiv) as the sulfoxonium ylide input and (*Z*)-1-methoxy-4-(prop-1-en-1-yl)benzene (cis-anethole, 59.3 mg, 0.400 mmol, 4.0 equiv) as the alkene input. Preparative TLC (20% $Et_2O$ in hexanes) provided the diastereomeric mixture of desired compound as a clear oil (11.4 mg, 41%). The diastereomeric ratio was analyzed via $^1H$ NMR integration, which showed 62:38 of **3o**:**4** (Figure S2).

**Figure S2**. $^1$H and $^{13}$C NMR of diastereomeric mixture of products from cis-anethole in conditions A with a comparison to the corresponding spectra for the diastereomerically pure product obtained from trans-anethole.

A 62:38 of **3o:4** obtained from cis-anethole

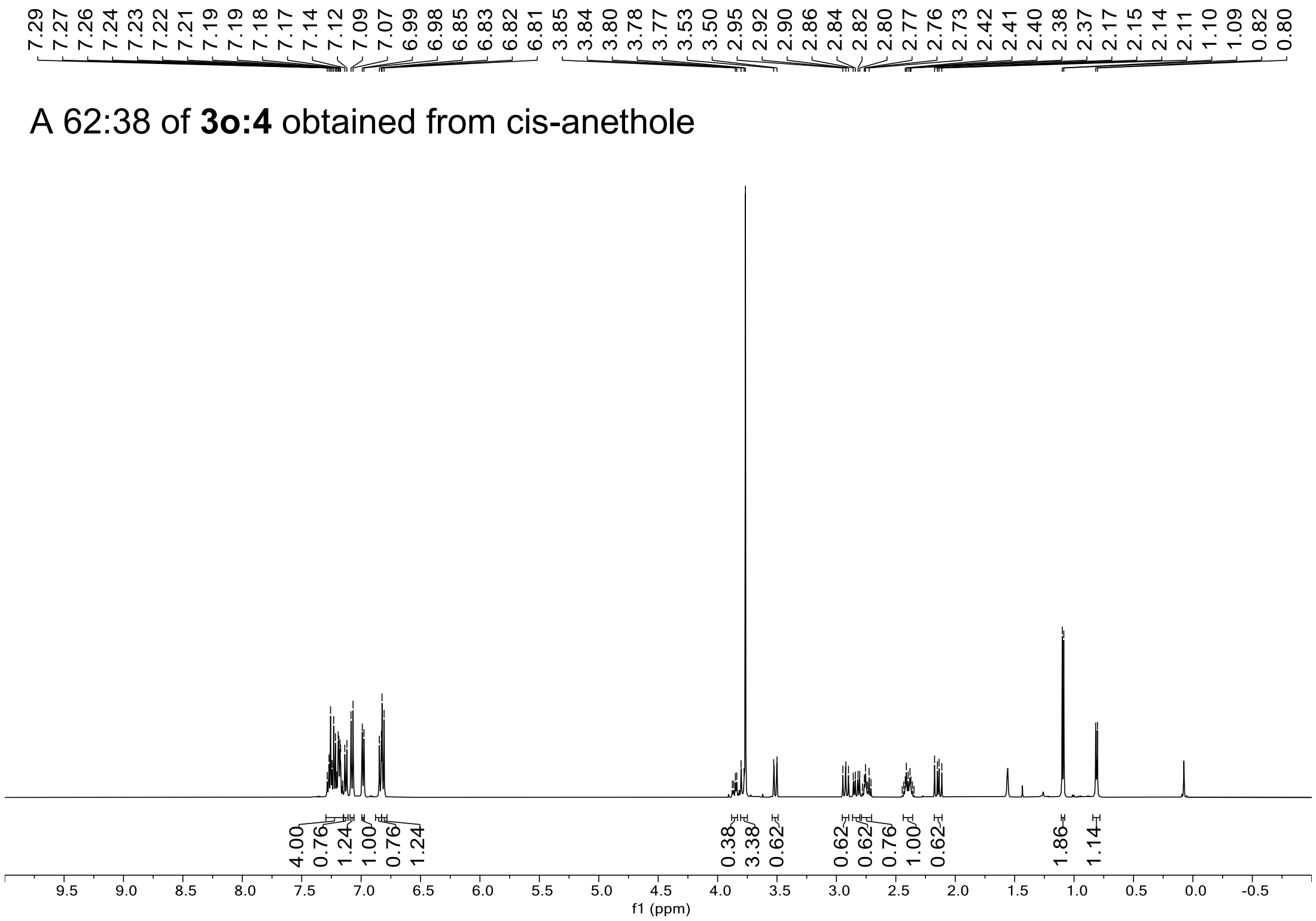

Diastereomerically pure **3o** obtained from trans-anethole

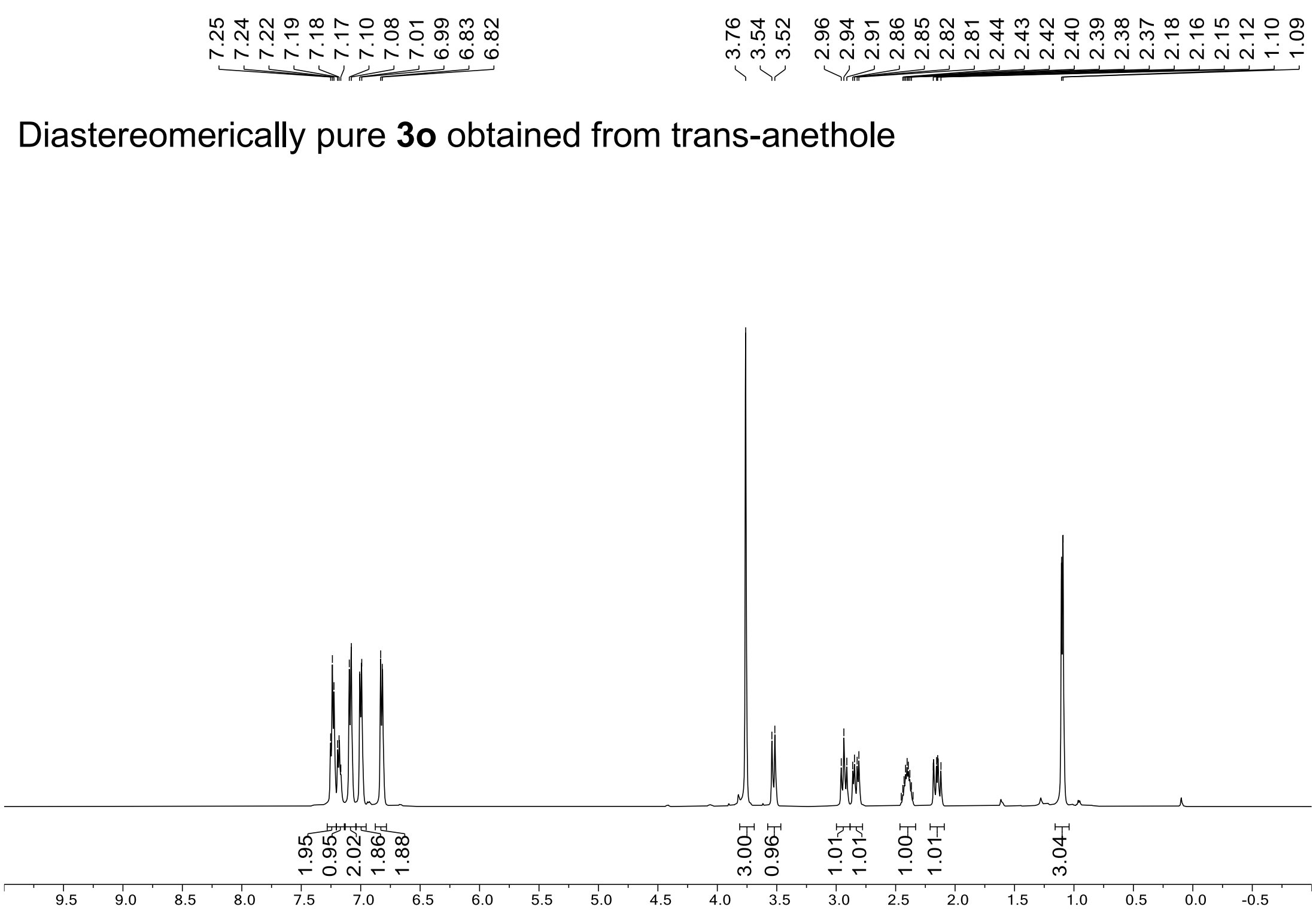

A 62:38 of **3o:4** obtained from cis-anethole

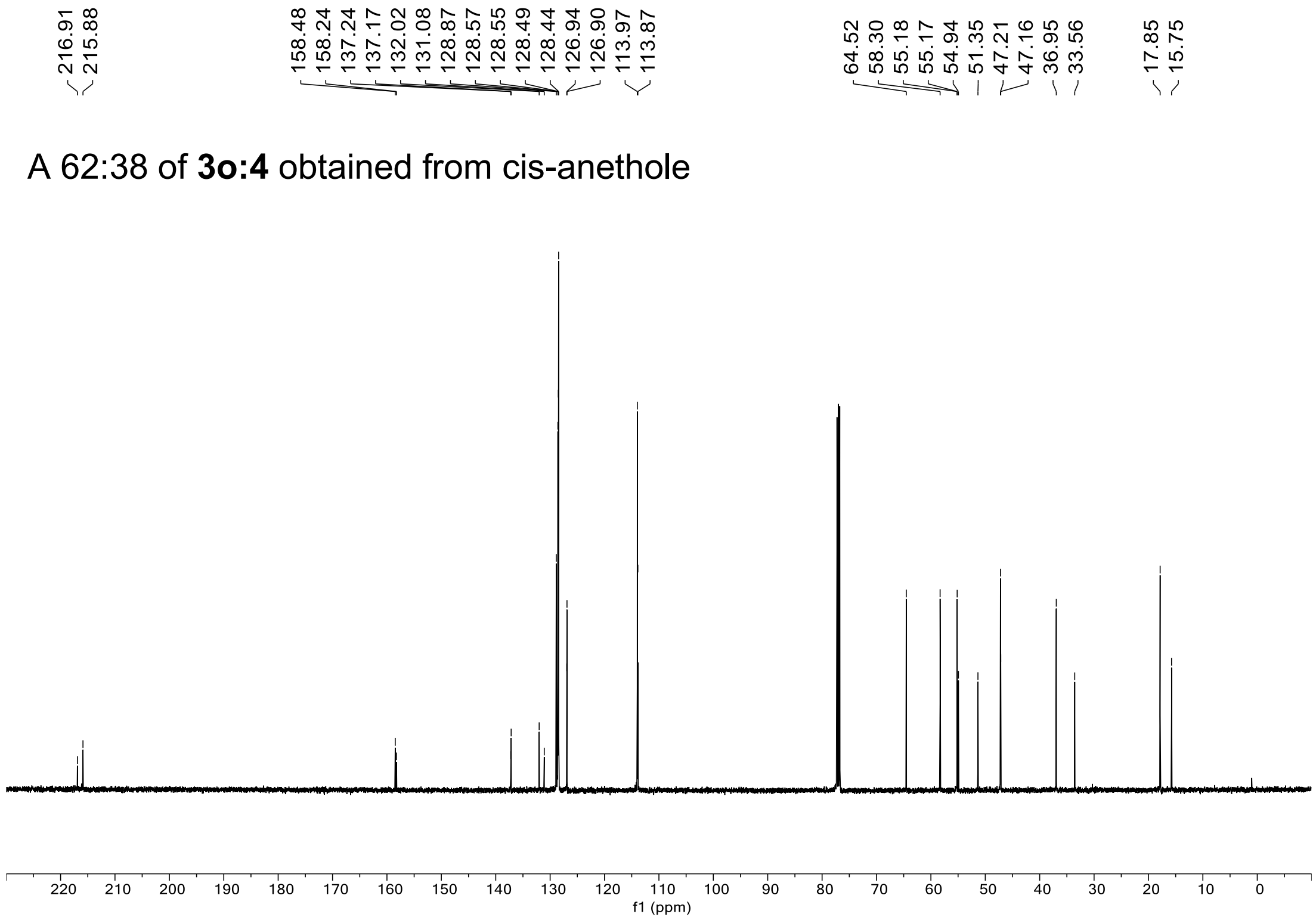


Diastereomerically pure **3o** obtained from trans-anethole

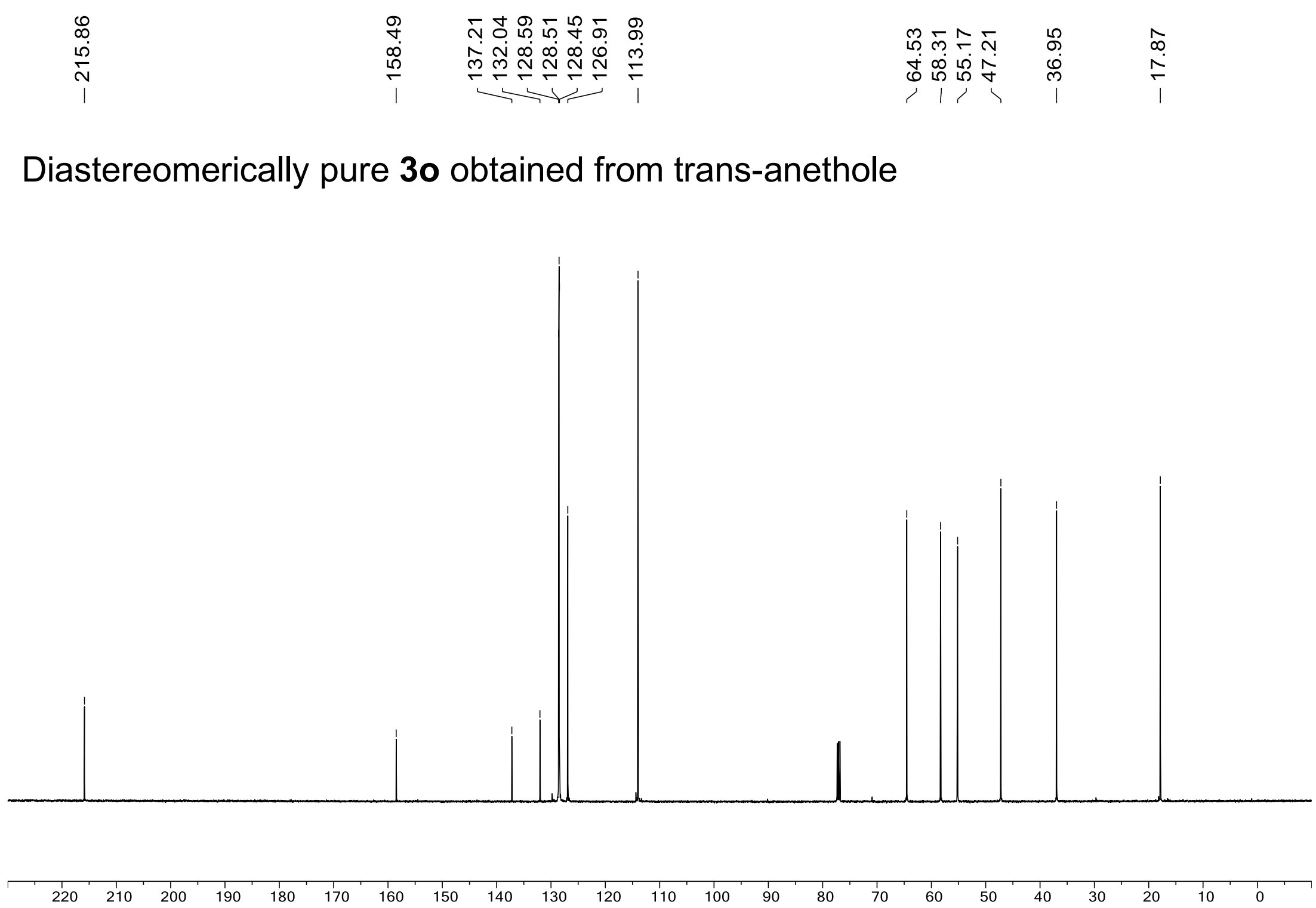

Analytical data for the 62:38 diastereomer mixture: **$^1$H NMR** (500 MHz, $CDCl_3$): δ 7.30–7.15 (m, 4H), 7.13 (d, *J* = 8.8 Hz, 0.76H), 7.08 (d, *J* = 8.7 Hz, 1.24H), 6.98 (d, *J* = 7.0 Hz, 1H), 6.84 (d, *J* = 8.7 Hz, 0.76H), 6.82 (d, *J* = 8.7 Hz, 1.24H), 3.86 (dd, *J* = 12.7, 5.7 Hz, 0.38H), 3.81–3.75 (m, 3.38H), 3.51 (d, *J* = 13.9 Hz, 0.62H), 2.96–2.88 (m, 0.62H), 2.83 (dd, *J* = 19.3, 8.1 Hz, 0.62H), 2.79–2.70 (m, 0.76H), 2.44–2.36 (m, 1H), 2.14 (dd, *J* = 18.6, 11.9 Hz, 0.62H), 1.09 (d, *J* = 6.5 Hz, 1.86H), 0.81 (d, *J* = 7.6 Hz, 1.14H). **$^{13}$C{$^1$H} NMR** (126 MHz, $CDCl_3$): δ 216.9, 215.9, 158.5, 158.2, 137.24, 137.17, 132.0, 131.1, 128.9, 128.57, 128.55, 128.49, 128.4, 126.94, 126.90, 114.0, 113.9, 64.5, 58.3, 55.18, 55.17, 54.9, 51.4, 47.21, 47.16, 37.0, 33.6, 17.9, 15.8. **HRMS** (EI, m/z): [M]+ calc'd for $C_{19}H_{20}O_2$, 280.1458; found, 280.1461.

<u>Synthesis of Cyclopentanone with Cis-Anethole: Conditions B</u>

General Procedure B was followed using 1-(dimethyl(oxo)-$\lambda^6$-sulfaneylidene)-3-phenylpropan-2-one (21.0 mg, 0.100 mmol, 1.0 equiv) as the sulfoxonium ylide input and (*Z*)-1-methoxy-4-(prop-1-en-1-yl)benzene (cis-anethole, 59.3 mg, 0.400 mmol, 4.0 equiv) as the alkene input. Preparative TLC (20% $Et_2O$ in hexanes) provided the diastereomeric mixture of desired compound as a clear oil (21.1 mg, 75%). The diastereomeric ratio was analyzed via $^1$H NMR integration, which showed 62:38 of **3o**:**4** (Figure S3).

**Figure S3**. $^1$H and $^{13}$C NMR of diastereomeric mixture of products from cis-anethole in conditions B.

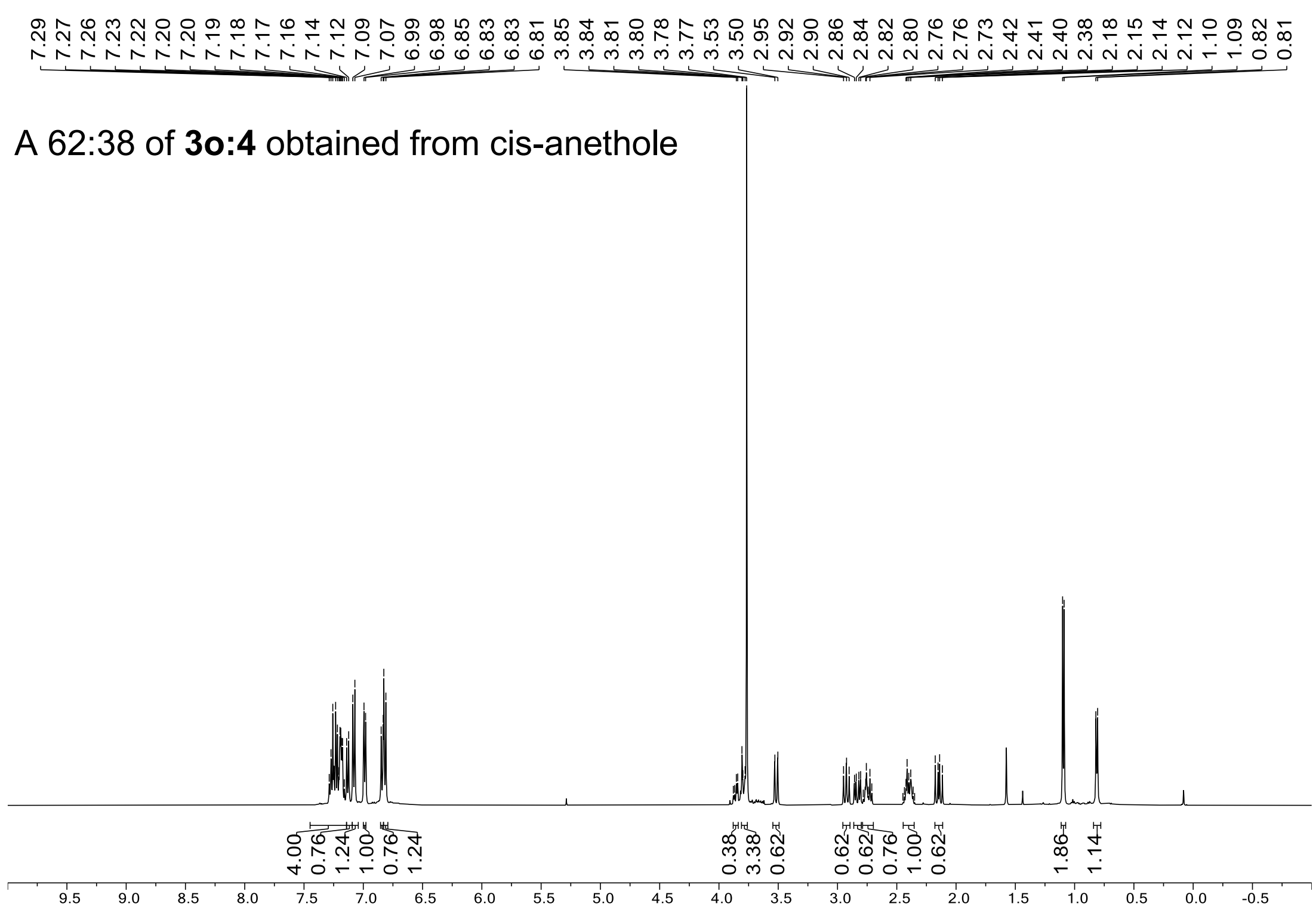

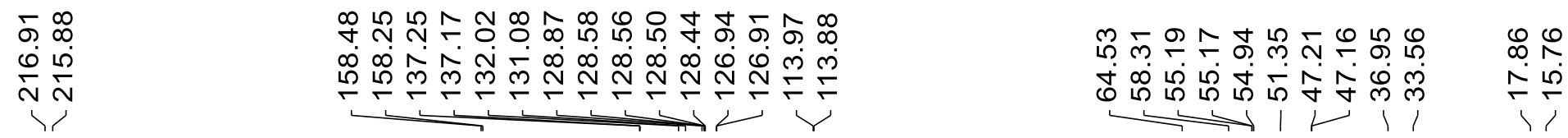


A 62:38 of **3o:4** obtained from cis-anethole

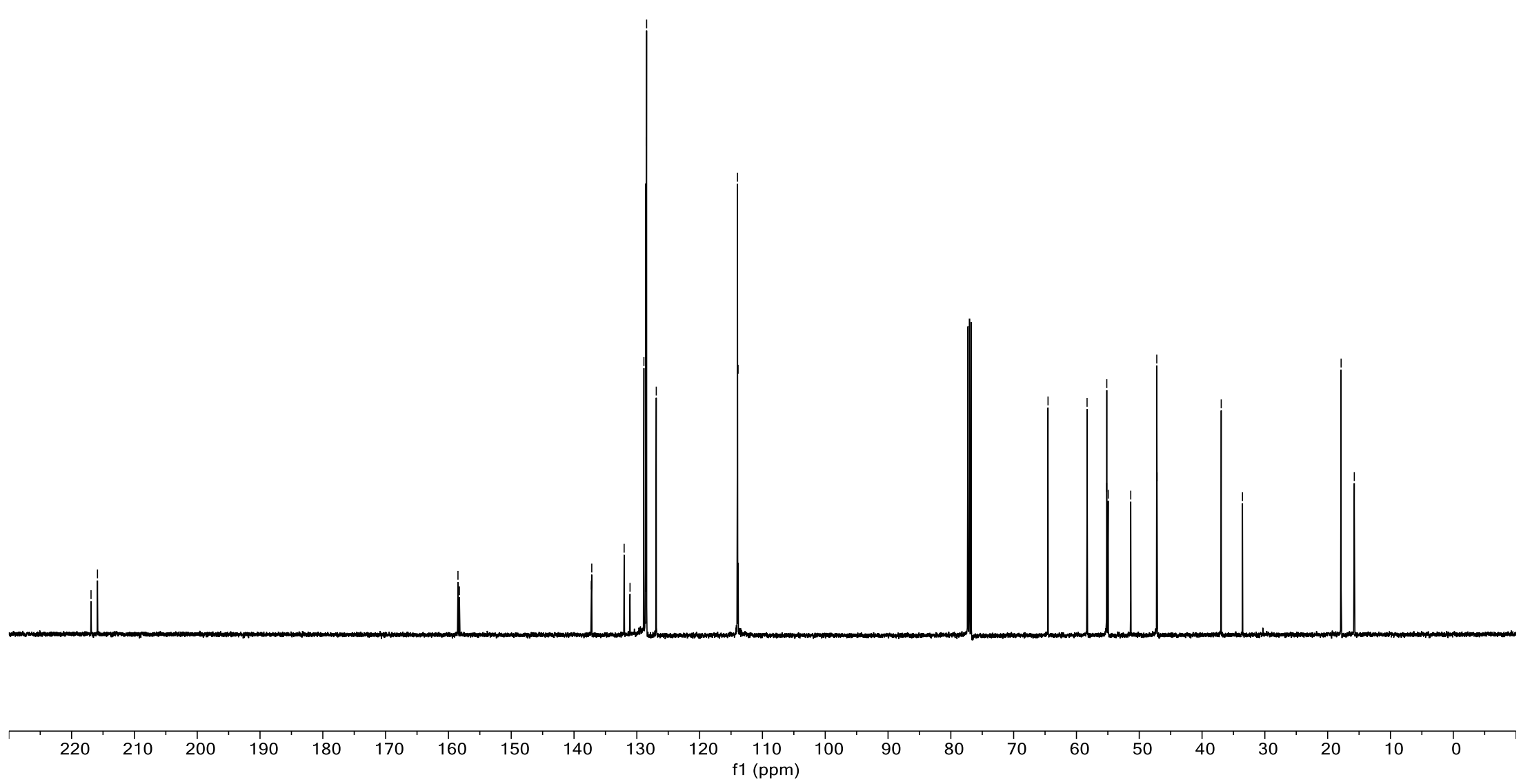


Alkene Isomerization Control

Each trans- and cis-anethole was subjected to the reaction conditions in the absence of sulfoxonium ylides to exclude the possibility of independent alkene isomerization as a side reaction pathway (Figure S4). The recovery rate of alkene was determined via crude $^1$H NMR analysis with trimethyl(phenyl)silane as standard. As indicated in Figure S4, extensive decomposition of both trans and cis- anethole occurred in the presence of $ZnCl_2$.

**Figure S4.** Recovery of anethole under conditions A and B.

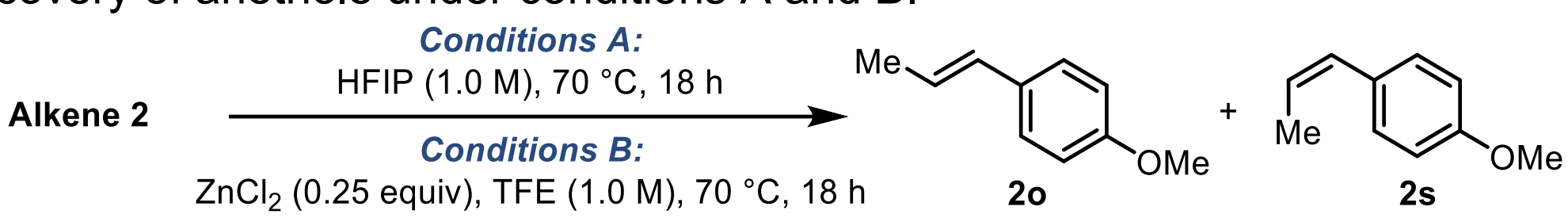


| Starting Alkene | | | 2o | 2s |
|---|---|---|---|---|
| 2o | 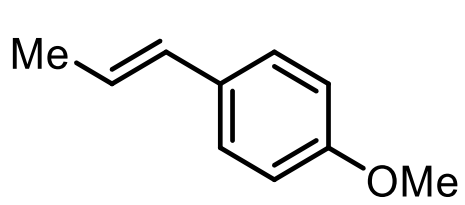 | ***Conditions A*** | quantitative | <2% |
| | | ***Conditions B*** | <2% | <2% |
| 2s | 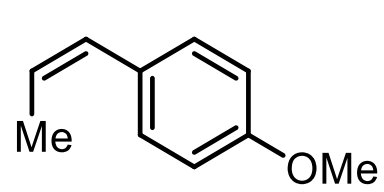 | ***Conditions A*** | <2% | 92% |
| | | ***Conditions B*** | <2% | <2% |

- Trans-Anethole Trial: Conditions A

In a nitrogen-filled glovebox, a 0.5–2.0 mL Biotage® microwave vial was charged with HFIP (100 μL, 1.0 M) and (*E*)-1-methoxy-4-(prop-1-en-1-yl)benzene (trans-anethole, 14.8 mg, 0.100 mmol, 1.0 equiv) followed by a magnetic stir bar. The vial was sealed, removed from the glovebox, and

placed in an oil bath preheated to 70 °C. After stirring for 18 h, the flask was removed from the bath and was allowed to cool to room temperature. The reaction mixture was transferred to a glass scintillation vial using $CH_2Cl_2$ and was concentrated.

A quantitative amount of (*E*)-1-methoxy-4-(prop-1-en-1-yl)benzene was recovered.

- Cis-Anethole Trial: Conditions A

The same procedure as above was repeated with (*Z*)-1-methoxy-4-(prop-1-en-1-yl)benzene instead of (*E*)-1-methoxy-4-(prop-1-en-1-yl)benzene.

92% of (*E*)-1-methoxy-4-(prop-1-en-1-yl)benzene was recovered with <2% of (*Z*)-isomer observed.

- Trans-Anethole Trial: Conditions B

In a nitrogen-filled glovebox, a 0.5–2.0 mL Biotage® microwave vial was charged with $ZnCl_2$ (3.4 mg, 0.025 mmol, 0.25 equiv), TFE (100 µL, 1.0 M), and (*E*)-1-methoxy-4-(prop-1-en-1-yl)benzene (trans-anethole, 14.8 mg, 0.100 mmol, 1.0 equiv) followed by a magnetic stir bar. The vial was sealed, removed from the glovebox, and placed in an oil bath preheated to 70 °C. After stirring for 18 h, the flask was removed from the bath and was allowed to cool to room temperature. The reaction mixture was transferred to a glass scintillation vial using $CH_2Cl_2$ and was concentrated.

None of the alkene was recovered.

- Cis-Anethole Trial: Conditions B

The same procedure as above was repeated with (*Z*)-1-methoxy-4-(prop-1-en-1-yl)benzene instead of (*E*)-1-methoxy-4-(prop-1-en-1-yl)benzene.

Likewise, none of the alkene was recovered. However, a less electron-rich alkene in the scope did not fully decompose by the end of the reaction time, providing the information on potential alkene isomerization under conditions B (*vide infra*).

### 4.2 Using Trans/Cis-Propenylbenzenes

We have also explored the reactivity of trans/cis-propenylbenzenes. Conditions A provided low yields for this substrate, and therefore it was not explored further.

**Figure S5.** Diastereomeric results with trans- and cis-propenylbenzene inputs.

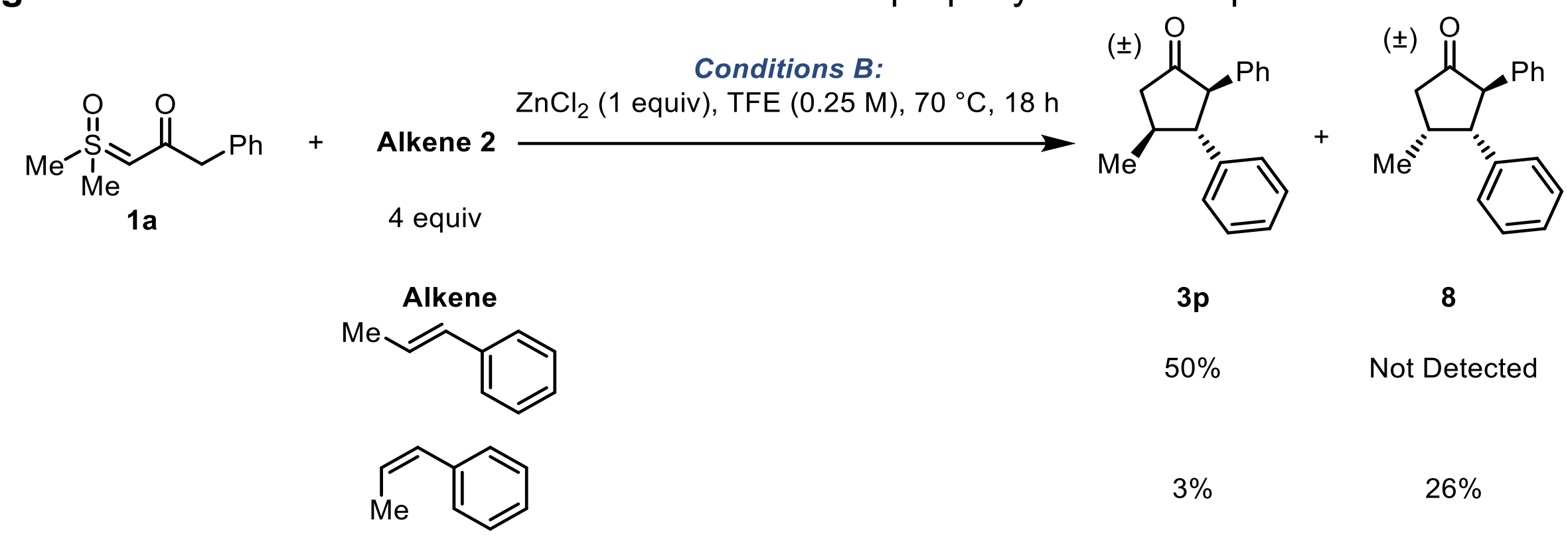


Synthesis of Cyclopentanone with Trans-Propenylbenzene: Conditions B

See Section 3.2 for compound **3p**. A single diastereomer was obtained as a white solid (50%).

Synthesis of Cyclopentanone with Cis-Propenylbenzene: Conditions B

General Procedure B was followed using 1-(dimethyl(oxo)-λ$^6$-sulfaneylidene)-3-phenylpropan-2-one (42.1 mg, 0.200 mmol, 1.0 equiv) as the sulfoxonium ylide input and (*Z*)-prop-1-en-1-ylbenzene (0.0945 g, 0.800 mmol, 4.0 equiv) as the alkene input. Preparative TLC (30% $Et_2O$ in hexanes) provided the diastereomeric mixture of desired compound as a yellow oil (14.6 mg, 29%). The diastereomeric ratio was analyzed via $^1$H NMR integration, which showed 9:91 of **3p**:**8**.

A higher degree of stereoretention was demonstrated in this case. The cation intermediate (see Scheme 2 in the manuscript) derived from this alkene is likely less stable than that from more electron-rich anethole, resulting in a shorter lifetime and less isomerization.

**Figure S6.** $^{1}$H and $^{13}$C NMR of diastereomeric mixture of products from cis-propenylbenzene in conditions B with a comparison to the corresponding spectra for the diastereomerically pure product obtained from trans-propenylbenzene.

A 9:91 of **3p:8** obtained from cis-propenylbenzene

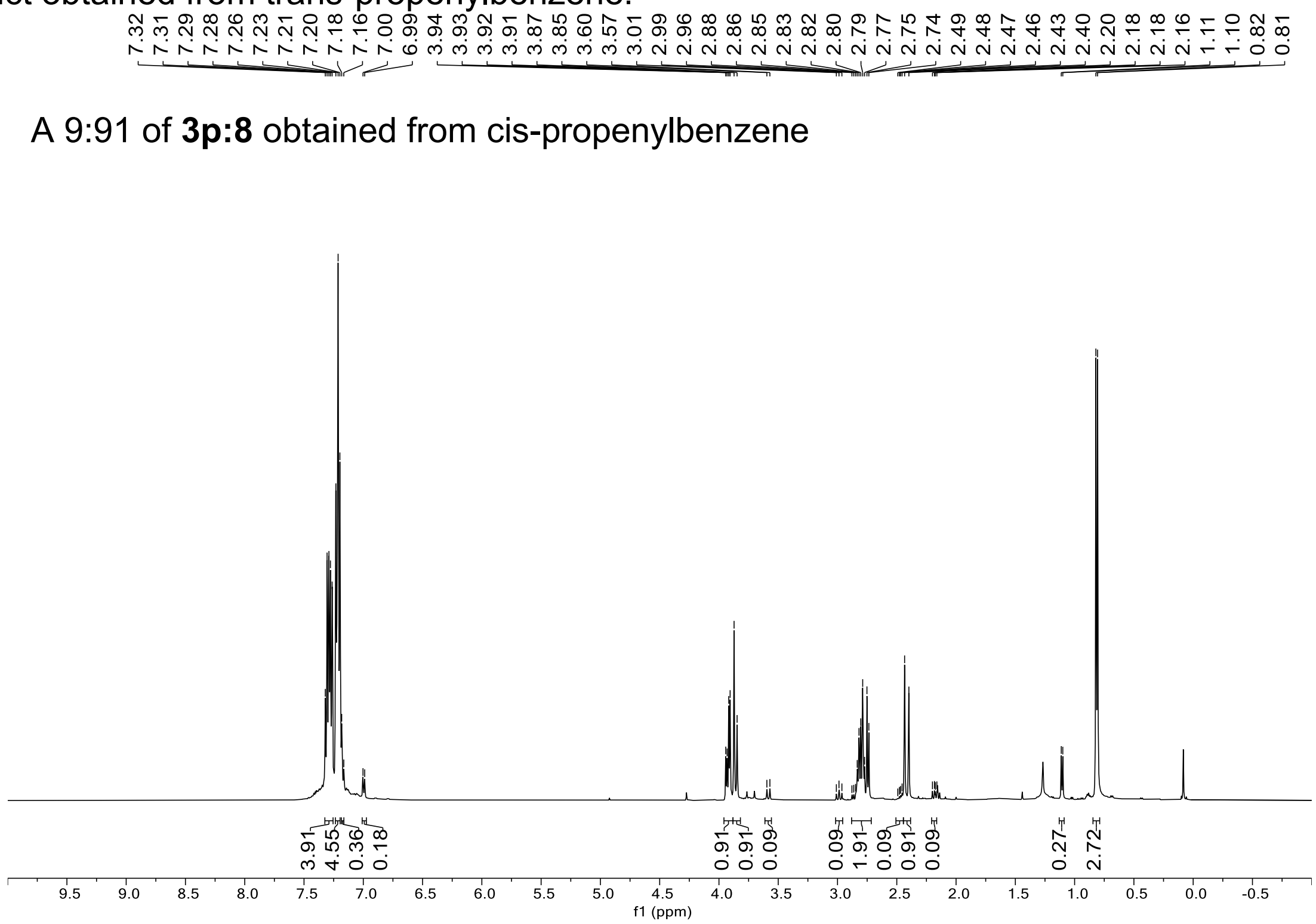


Diastereomerically pure **3p** obtained from trans-propenylbenzene

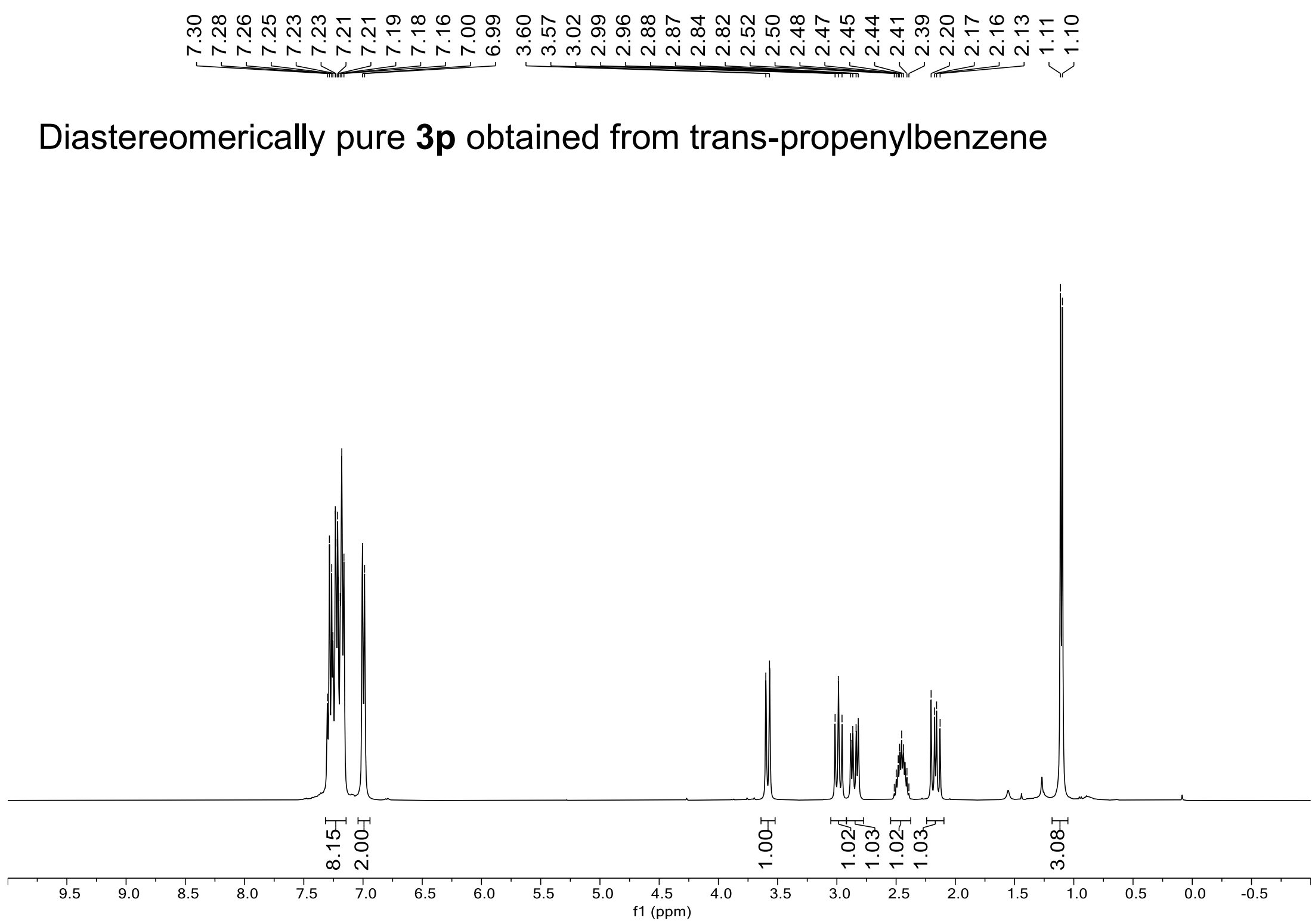

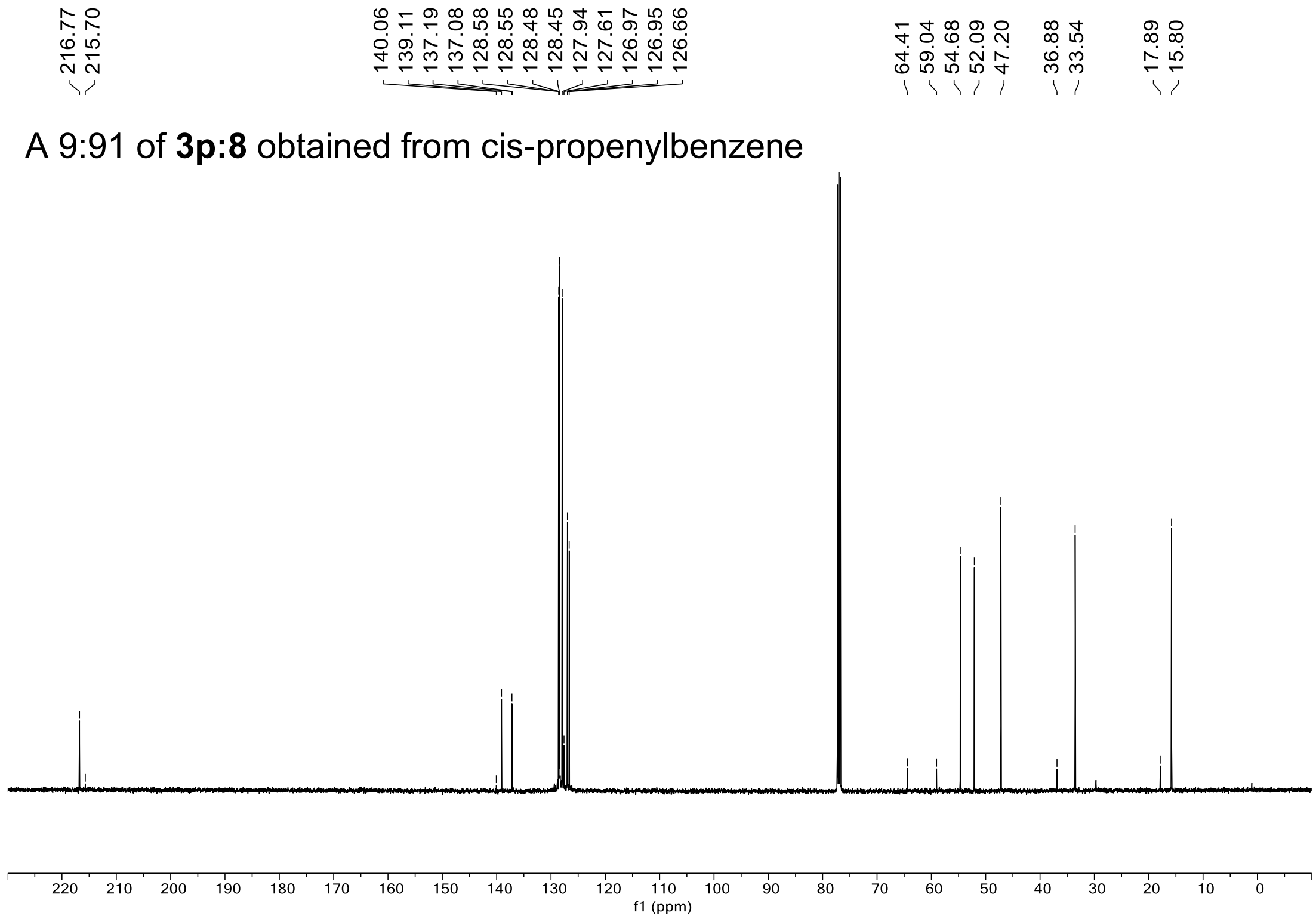


A 9:91 of **3p:8** obtained from cis-propenylbenzene

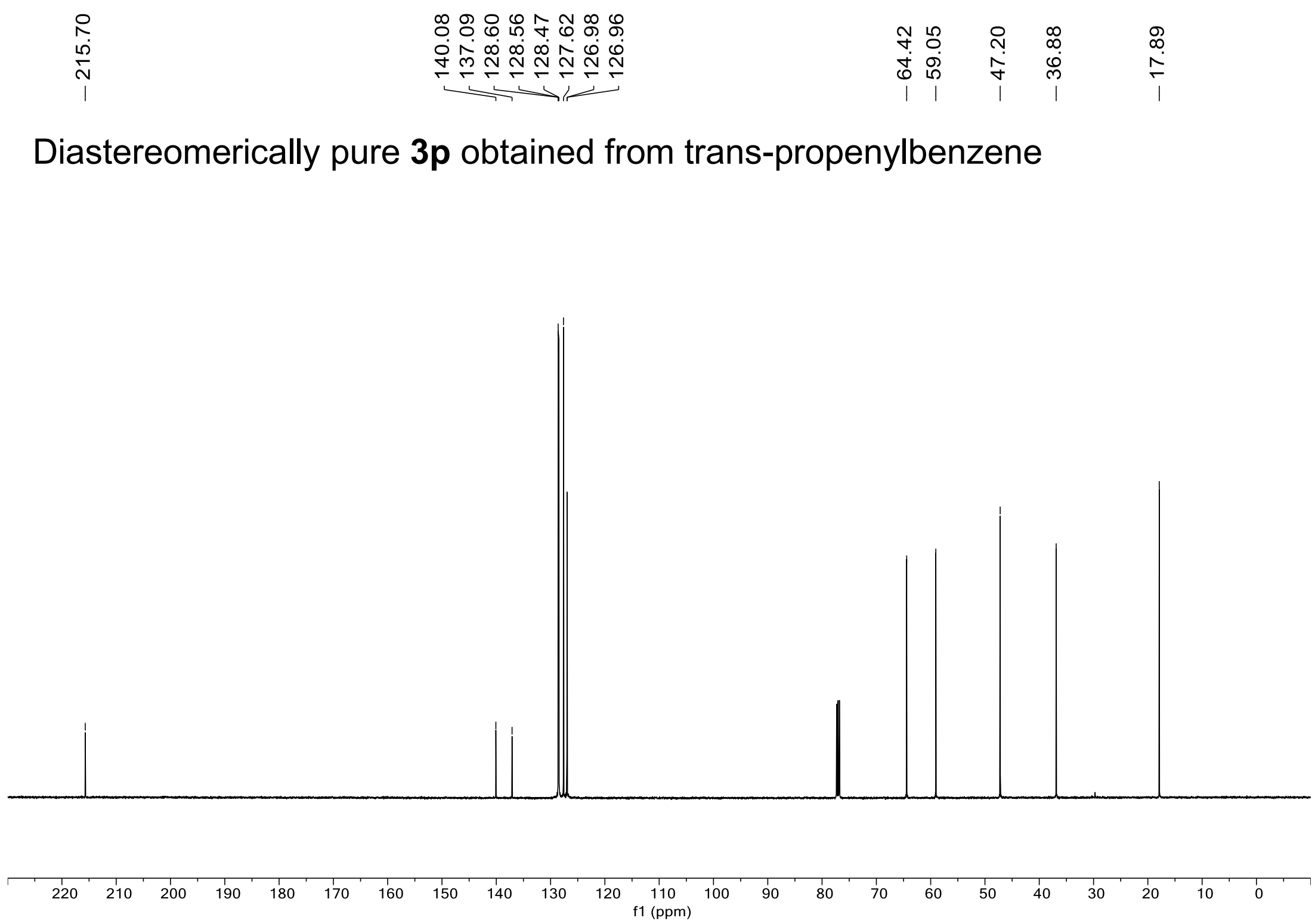


Diastereomerically pure **3p** obtained from trans-propenylbenzene

Analytical data for NMR spectra of 9:91 diastereomer mixture: **$^{1}$H NMR** (500 MHz, $CDCl_3$): δ 7.32–7.25 (m, 3.91H), 7.24–7.20 (m, 4.55H), 7.19–7.14 (m, 0.36H), 7.00 (d, *J* = 7.1 Hz, 0.18H), 3.92 (dd, *J* = 12.6, 5.9 Hz, 0.91H), 3.86 (d, *J* = 12.7 Hz, 0.91H), 3.58 (d, *J* = 12.7 Hz, 0.09H), 3.03–2.95 (m, 0.09H), 2.88–2.72 (m, 1.91H), 2.49–2.45 (m, 0.09H), 2.42 (d, *J* = 18.0 Hz, 0.91H), 2.18 (dd, *J* = 10.9, 7.5 Hz, 0.09H), 1.11 (d, *J* = 6.4 Hz, 0.27H), 0.81 (d, *J* = 7.1 Hz, 2.72H). **$^{13}C\{^{1}H\}$ NMR** (126 MHz, $CDCl_3$): 216.8, 215.7, 140.1, 139.1, 137.2, 137.1, 128.58, 128.55, 128.48, 128.45, 127.9, 127.6, 127.0, 126.9, 126.7, 64.4, 59.0, 54.7, 52.1, 47.2, 36.9, 33.5, 17.9, 15.8.

<u>Alkene Isomerization Control</u>

**Figure S7**. Recovery of propenylbenzene under conditions A and B.

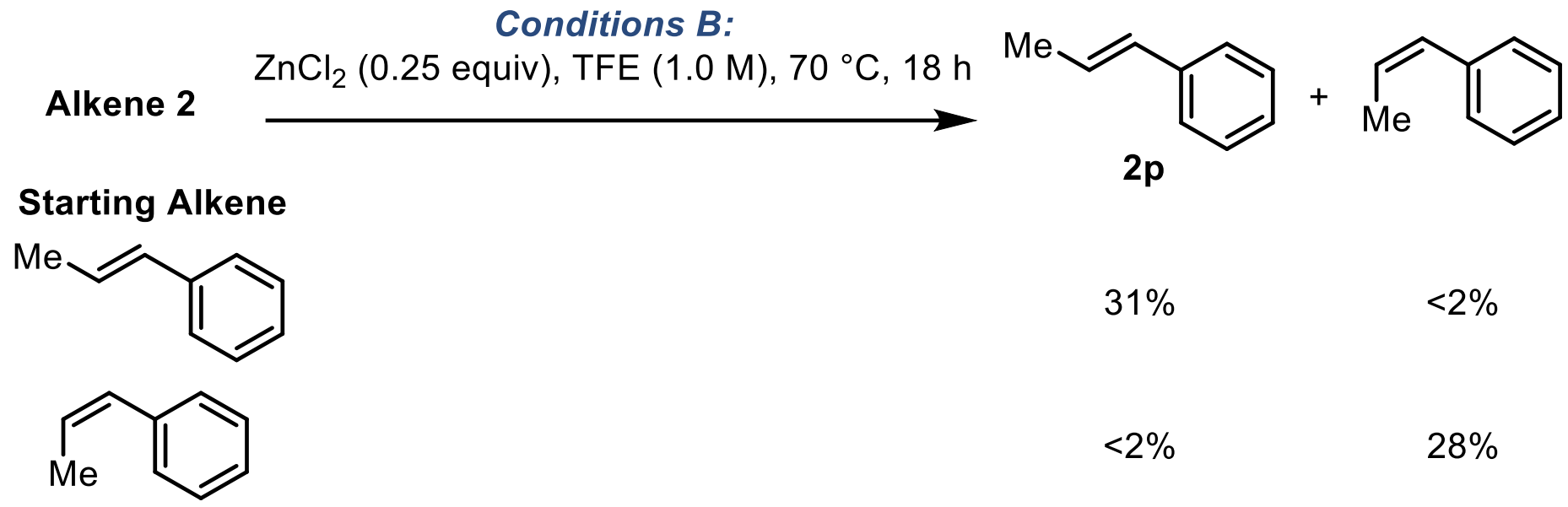


- <u>Trans-Propenylbenzene Trial: Conditions B</u>

In a nitrogen-filled glovebox, a 0.5–2.0 mL Biotage® microwave vial was charged with $ZnCl_2$ (3.4 mg, 0.025 mmol, 0.25 equiv), TFE (100 µL, 1.0 M), and (*E*)-prop-1-en-1-ylbenzene (11.8 mg, 0.100 mmol, 1.0 equiv) followed by a magnetic stir bar. The vial was sealed, removed from the glovebox, and placed in an oil bath preheated to 70 °C. After stirring for 18 h, the flask was removed from the bath and allowed to cool to room temperature. The reaction mixture was transferred to a glass scintillation vial using $CH_2Cl_2$ and was concentrated.

31% of (*E*)-prop-1-en-1-ylbenzene was recovered with <2% of (*Z*)-isomer observed.

- <u>Cis-Propenylbenzene Trial: Conditions B</u>

The same procedure as above was repeated with (*Z*)-prop-1-en-1-ylbenzene instead of (*E*)-prop-1-en-1-ylbenzene.

28% of (*E*)-prop-1-en-1-ylbenzene was recovered with <2% of (*E*)-isomer observed.

## 4.3 Use of a Chiral Phosphoric Acid Catalyst

While exploring different conditions, we noticed that using 20 mol % of diphenyl phosphate delivers the desired product at a good to moderate yield (see Table 1 and 2 of the manuscript). Since the achiral phosphoric acid is an effective additive, we envisioned that chiral phosphoric acid catalysts could induce enantioselectivity if these Brønsted acids actively participate in the cycloaddition step. We chose the commercially available (*S*)-TRIP as the chiral phosphoric acid input. However, minimal to no enantiomeric induction was observed.

**Figure S8**. (*S*)-TRIP catalyst as an acid source in the reaction conditions.

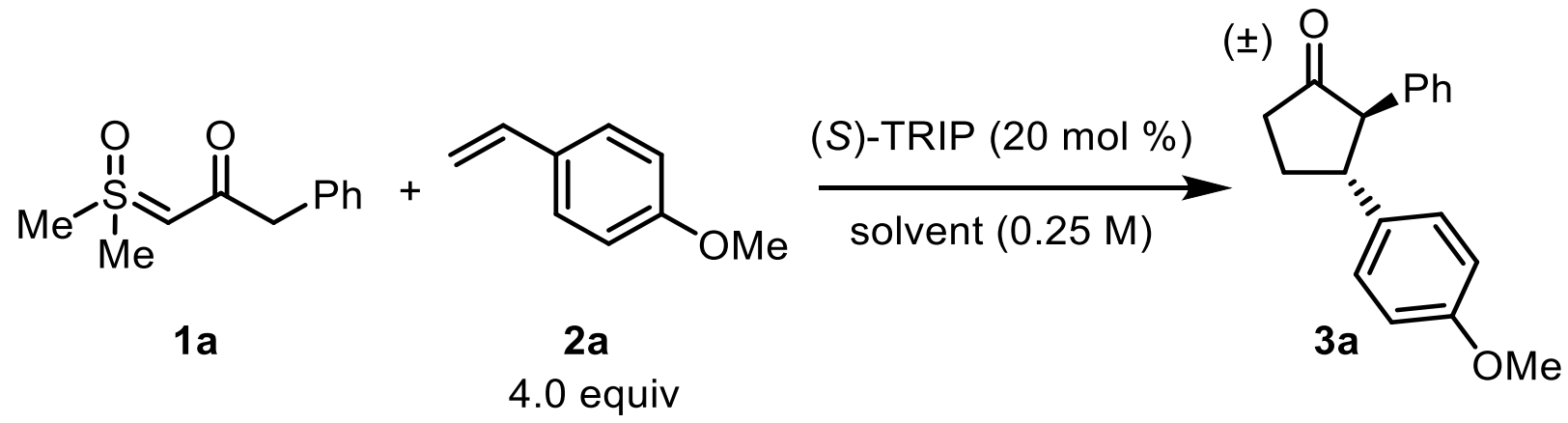


| *entry* | *solvent* | *T* | *time* | ***yield*** | ***er*** |
|---|---|---|---|---|---|
| **1** | TFE | 70 °C | 18 h | 56% | 52:48 |
| **2** | DCE | rt | 48 h | 13% | 50:50 |

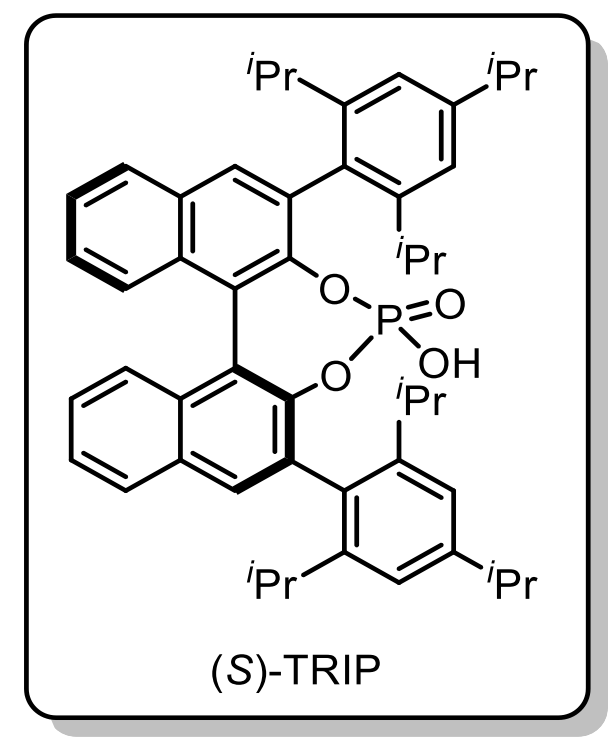


Entry 1:

In a nitrogen-filled glovebox, a 0.5–2.0 mL Biotage® microwave vial was charged with 1-(dimethyl(oxo)-λ$^6$-sulfaneylidene)-3-phenylpropan-2-one (21.0 mg, 0.200 mmol, 1.0 equiv). In a separate vial, (*S*)-TRIP (15.1 mg, 0.0200 mmol, 0.20 equiv) was weighed and dissolved in TFE (400 µL, 0.25 M). The solution was transferred to the microwave vial with the sulfoxonium ylide using a glass pipette. 1-Methoxy-4-vinylbenzene (53.7 mg, 0.400 mmol, 4.0 equiv) and a magnetic stir bar were added last. The vial was sealed, removed from the glovebox, and placed in an oil bath preheated to 70 °C. After stirring for 18 h, the flask was removed from the bath and allowed to cool to room temperature. The reaction mixture was transferred to a glass scintillation vial using $CH_2Cl_2$ and was concentrated. Preparative TLC (30% $Et_2O$ in hexanes) provided **3a** as a white solid (15.0 mg, 56%, 52:48 er)

Entry 2: Because TFE as the solvent and a high reaction temperature led to ineffective enantiomeric induction (see Entry 1), we also tried a room temperature trial in DCE.

In a nitrogen-filled glovebox, a 10–20 mL Biotage® microwave vial was charged with 1-(dimethyl(oxo)-λ$^6$-sulfaneylidene)-3-phenylpropan-2-one (63.1 mg, 0.300 mmol, 1.0 equiv). In a separate vial, (*S*)-TRIP (45.2 mg, 0.0600 mmol, 0.20 equiv) was weighed and dissolved in DCE (1.20 mL, 0.25 M). The solution was transferred to the microwave vial with the sulfoxonium ylide using a glass pipette. 1-Methoxy-4-vinylbenzene (0.161 g, 1.200 mmol, 4.0 equiv) and a magnetic stir bar were added last. The vial was sealed, removed from the glovebox, and placed in a room temperature water bath. After stirring for 48 h, the reaction mixture was transferred to a glass scintillation vial using $CH_2Cl_2$ and was concentrated. Preparative TLC (30% $Et_2O$ in hexanes) provided **3a** as a white solid (10.6 mg, 13%, 50:50 er).

**Figure S9**. Enantiomeric ratio analysis of **3a** via HPLC.

Racemic **3a** (Chiralpak AD-H, 90:10 hexanes:isopropanol, 1 mL/min, 210 nm)

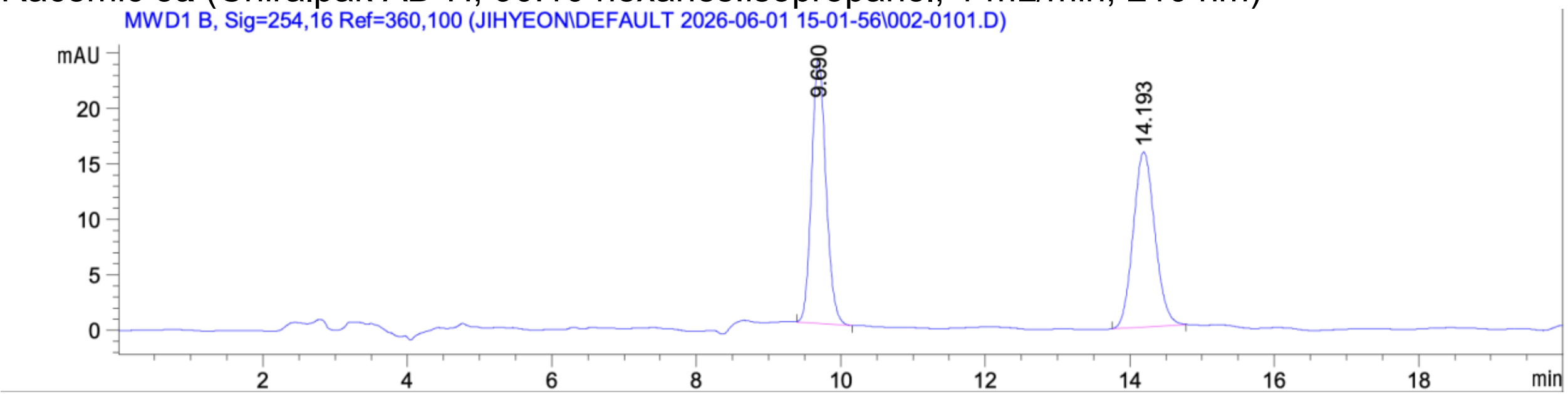


Signal 3: MWD1 C, Sig=210,8 Ref=360,100

| Peak # | RetTime [min] | Type | Width [min] | Area [mAU*s] | Height [mAU] | Area % |
|---|---|---|---|---|---|---|
| 1 | 6.272 | VV | 0.1385 | 9880.34766 | 1102.96936 | 49.4714 |
| 2 | 9.201 | VV | 0.2086 | 1.00915e4 | 742.48090 | 50.5286 |
| Totals : | | | | 1.99719e4 | 1845.45026 | |

Entry 1 (Chiralpak AD-H, 90:10 hexanes:isopropanol, 1 mL/min, 210 nm)

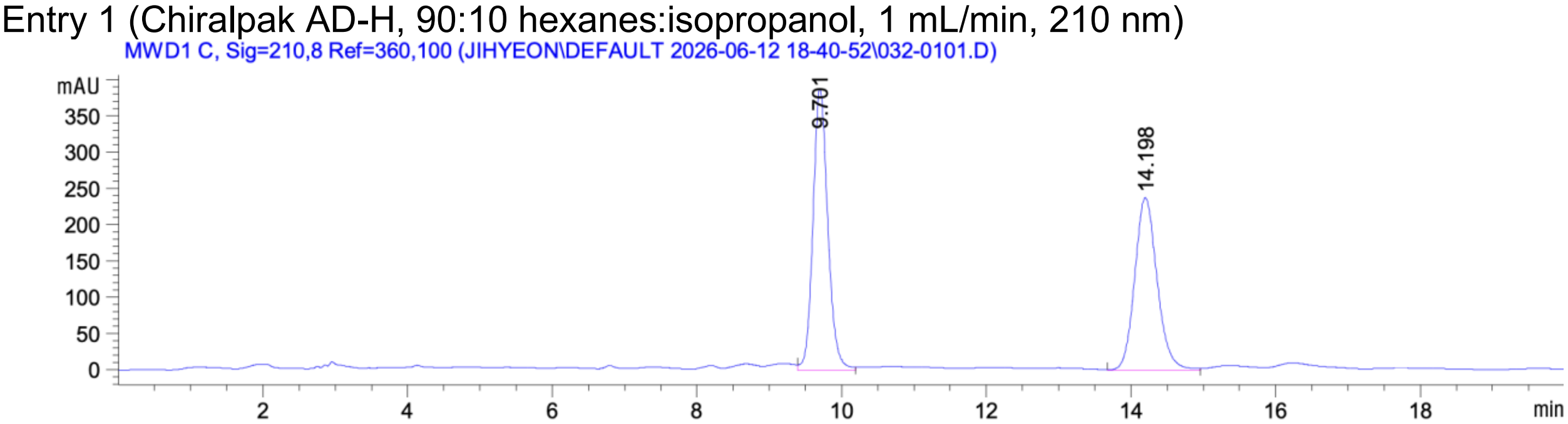


Signal 3: MWD1 C, Sig=210,8 Ref=360,100

| Peak # | RetTime [min] | Type | Width [min] | Area [mAU*s] | Height [mAU] | Area % |
|---|---|---|---|---|---|---|
| 1 | 9.701 | VV | 0.2207 | 5550.28125 | 388.39896 | 51.9083 |
| 2 | 14.198 | VV | 0.3319 | 5142.18945 | 238.19400 | 48.0917 |
| Totals : | | | | 1.06925e4 | 626.59296 | |

Entry 2 (Chiralpak AD-H, 90:10 hexanes:isopropanol, 1 mL/min, 210 nm)

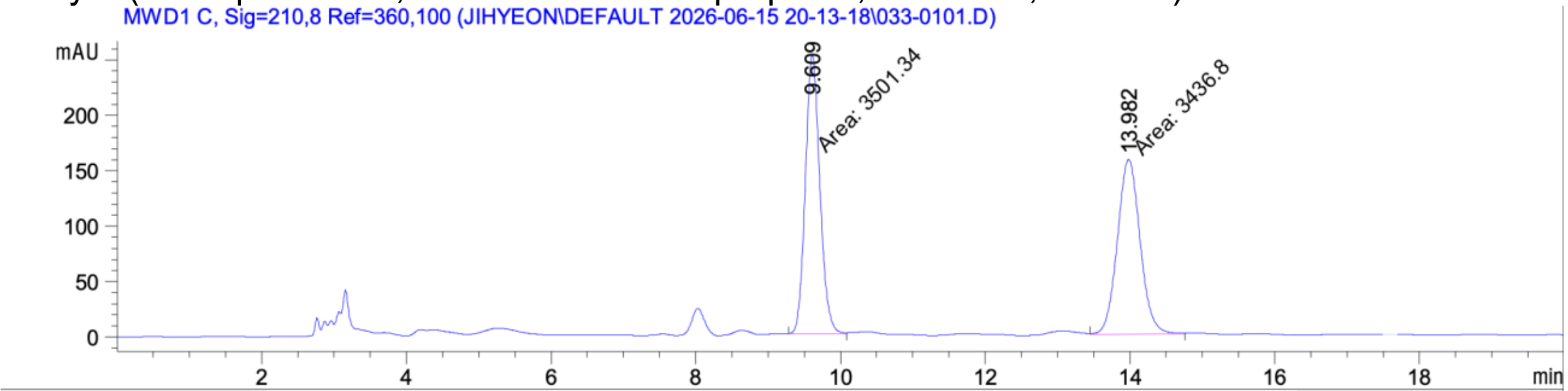


Signal 3: MWD1 C, Sig=210,8 Ref=360,100

| Peak # | RetTime [min] | Type | Width [min] | Area [mAU*s] | Height [mAU] | Area % |
|---|---|---|---|---|---|---|
| 1 | 9.609 | MM | 0.2320 | 3501.34497 | 251.48180 | 50.4651 |
| 2 | 13.982 | MM | 0.3634 | 3436.80103 | 157.60321 | 49.5349 |
| Totals : | | | | 6938.14600 | 409.08501 | |

## 5. Synthetic Elaborations of the Product

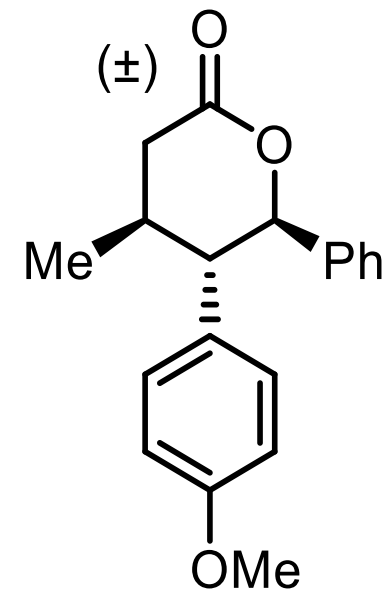


**5-(4-Methoxyphenyl)-4-methyl-6-phenyltetrahydro-2H-pyran-2-one (5)**. The reaction was performed using a procedure adapted from the literature.[14] To an oven-dried 2–5 mL Biotage® microwave vial were added the cyclopentanone **3o** (0.150 mmol, 1.0 equiv, 42.1 mg) and a magnetic stir bar. Once the vial was capped, it was evacuated and backfilled with nitrogen thrice. Anhydrous $CH_2Cl_2$ (3.0 mL) was syringed into the vial. The reaction mixture was cooled to 0 °C in an ice bath, after which *m*CPBA (0.300 mmol, 2.0 equiv, 74.0 mg, 70–75% purity) and $NaHCO_3$ (0.300 mmol, 2.0 equiv, 25.2 mg) were added quickly under positive nitrogen pressure. After 8 h at 0 °C under a nitrogen atmosphere, the reaction mixture was diluted with $H_2O$ (5 mL). 10 mL of $H_2O$ and 20 mL of $CH_2Cl_2$ were added for layer separation. The aqueous layer was further extracted with $CH_2Cl_2$ (10 x 3 mL). The organic layers were combined, dried over $Na_2SO_4$, and concentrated. The desired product was obtained via preparative TLC (40% EtOAc in hexanes) as a white crystalline solid (27.6 mg, 62%). **M.p.**: 132 °C. **IR** (neat): 3009, 2959, 2928, 2872, 2835, 1730, 1611, 1512, 1456, 1364, 1273, 1252, 1229, 1169, 1099, 1020, 827, 766, 706, 573, 556, 534 $cm^{-1}$. **$^1$H NMR** (500 MHz, $CDCl_3$): δ 7.16 (m, 3H), 7.04–6.90 (m, 2H), 6.83 (d, *J* = 8.6 Hz, 2H), 6.72 (d, *J* = 8.6 Hz, 2H), 5.28 (d, *J* = 10.8 Hz, 1H), 3.72 (s, 3H), 3.03–2.91 (m, 1H), 2.59 (t, J = 10.5 Hz, 1H), 2.52–2.36 (m, 2H), 0.85 (d, *J* = 6.2 Hz, 3H). **$^{13}$C{$^1$H} NMR** (126 MHz, $CDCl_3$): δ 170.7, 158.6, 138.3, 130.0, 129.3, 128.1, 128.0, 126.9, 114.0, 87.1, 55.1, 54.6, 38.3, 32.7, 19.9. **HRMS** (EI, m/z): [M]+ calc'd for $C_{19}H_{20}O_3$, 296.1407; found, 296.1404.

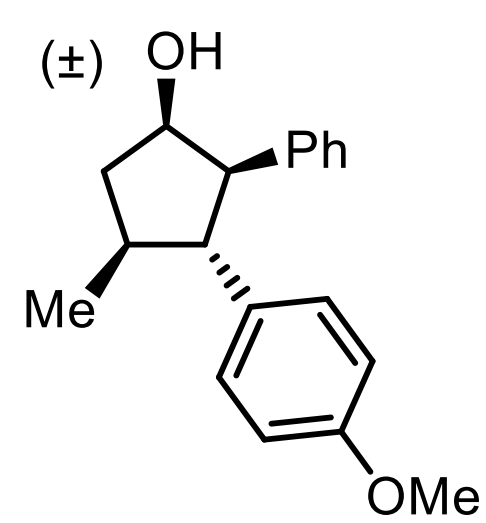


**3-(4-Methoxyphenyl)-4-methyl-2-phenylcyclopentan-1-ol (6)**. The reaction was performed using a procedure adapted from the literature.[15] To an oven-dried 2–5 mL Biotage® microwave vial were added the cyclopentanone **3o** (0.150 mmol, 1.0 equiv, 42.1 mg) and a magnetic stir bar. Once the vial was capped, it was evacuated and backfilled with nitrogen thrice. Anhydrous THF (1.4 mL) was syringed into the vial. The reaction mixture was cooled to –78 °C in an acetone/dry ice dewar, and L-selectride (1 M in THF, 3.0 equiv, 0.45 mL) was added via syringe. After stirring for 30 min, the vial was allowed to gradually warm to room temperature and then placed in an ambient temperature water bath overnight. After stirring for 16.5 h at room temperature, the reaction was quenched via slow dropwise addition of MeOH (3 mL, vigorous bubbling at first occurred), which was followed by addition of $H_2O$ (1 mL). The crude mixture was concentrated and then diluted with 5 mL of $H_2O$ and 5 mL of $Et_2O$. The organic layer was collected, and the

aqueous layer was further extracted with $Et_2O$ (5 x 2 mL). The organic layers were combined, dried over $Na_2SO_4$, and concentrated. The desired product was obtained via preparative TLC (3:1:6 of $CH_2Cl_2$:acetone:hexanes) as a white crystalline solid (30.9 mg, 73%). The relative stereochemistry was determined by X-ray crystallographic data (see Section 7). **M.p.**: 79 °C. **IR** (neat): 3532, 3428, 3032, 2947, 2903, 2832, 1613, 1584, 1512, 1451, 1302, 1244, 1175, 1123, 1030, 1013, 880, 820, 802, 752, 702, 619, 544, 505 $cm^{-1}$. **$^1$H NMR** (500 MHz, $CDCl_3$): δ 7.29–7.19 (m, 4H), 7.18–7.07 (m, 3H), 6.77 (d, *J* = 7.0 Hz, 2H), 4.40–4.32 (m, 1H), 3.72 (s, 3H), 3.35 (dd, *J* = 12.4, 5.2 Hz, 1H), 3.10 (t, *J* = 11.4 Hz, 1H), 2.62–2.53 (m, 1H), 2.09–1.97 (m, 1H), 1.57–1.48 (m, 1H), 1.27 (s, 1H), 1.08 (d, *J* = 6.6 Hz, 3H). **$^{13}$C{$^1$H} NMR** (126 MHz, $CDCl_3$): δ 157.9, 138.1, 134.2, 129.2, 128.6, 128.4, 126.7, 113.7, 74.7, 59.0, 55.1, 53.4, 41.8, 41.5, 19.4. **HRMS** (EI, m/z): [M]+ calc'd for $C_{19}H_{22}O_2$, 282.1614; found, 282.1618.

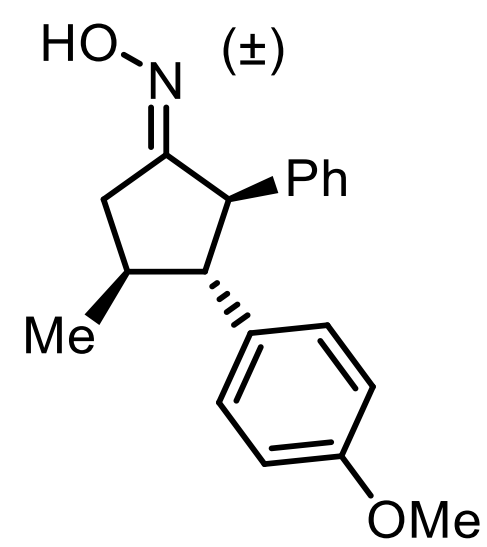


**(*E*)-3-(4-Methoxyphenyl)-4-methyl-2-phenylcyclopentan-1-one oxime (7)**. The reaction was performed using a procedure adapted from the literature.[16] To an oven-dried 2–5 mL Biotage® microwave vial were added the cyclopentanone **3o** (0.200 mmol, 1.0 equiv, 56.1 mg) and a magnetic stir bar. Once the vial was capped, it was evacuated and backfilled with nitrogen thrice. Anhydrous MeOH (1.0 mL) was syringed into the vial. Hydroxylamine hydrochloride ($NH_2OH$•HCl, 0.600 mmol, 3.0 equiv, 41.7 mg) and anhydrous NaOAc (2.00 mmol, 10 equiv, 0.164 g, freshly dried in a vacuum oven at 120 °C) were added quickly under positive nitrogen pressure. After stirring in a 70 °C pre-heated oil bath for 1 h, the reaction flask was allowed to cool to room temperature. The crude mixture was filtered through a medium-fritted funnel and was rinsed using $CH_2Cl_2$. The filtrate was collected and concentrated under reduced pressure. The desired product was obtained via flash silica chromatography (30% EtOAc in hexanes) as a white solid (48.0 mg, 81%). The (*Z*)-isomer was also collected as a white crystalline solid but was not characterized or used for further elaboration (lower Rf, 10.2 mg, 17%). **M.p.**: 157 °C. **IR** (neat): 3256, 3034, 2953, 2901, 2862, 1614, 1514, 1449, 1250, 1177, 1030, 963, 835, 804, 758, 698, 635, 544, 527 $cm^{-1}$. **$^1$H NMR** (500 MHz, $CDCl_3$): δ 7.67 (br s, 1H), 7.21 (t, *J* = 7.5 Hz, 2H), 7.15 (t, *J* = 7.1 Hz, 1H), 7.03 (d, *J* = 7.6 Hz, 2H), 6.99 (d, *J* = 8.7 Hz, 2H), 6.77 (d, *J* = 8.6 Hz, 2H), 3.82–3.71 (m, 4H), 3.12 (dd, *J* = 12.5, 12.3 Hz, 1H), 2.68 (t, *J* = 11.2 Hz, 1H), 2.29–2.17 (m, 2H), 1.01 (d, *J* = 5.9 Hz, 3H). **$^{13}$C{$^1$H} NMR** (126 MHz, $CDCl_3$): δ 166.9, 158.3, 139.7, 132.0, 128.6, 128.5, 128.3, 126.7, 113.8, 60.8, 57.9, 55.1, 38.5, 35.6, 17.8. **HRMS** (EI, m/z): [M]+ calc'd for $C_{19}H_{21}NO_2$, 295.1567; found, 295.1568.

## 6. COSY and NOE Experiments for Relative Stereochemistry

### 3-Methyl-2-phenyl-3-(*p*-tolyl)cyclopentan-1-one (3m).

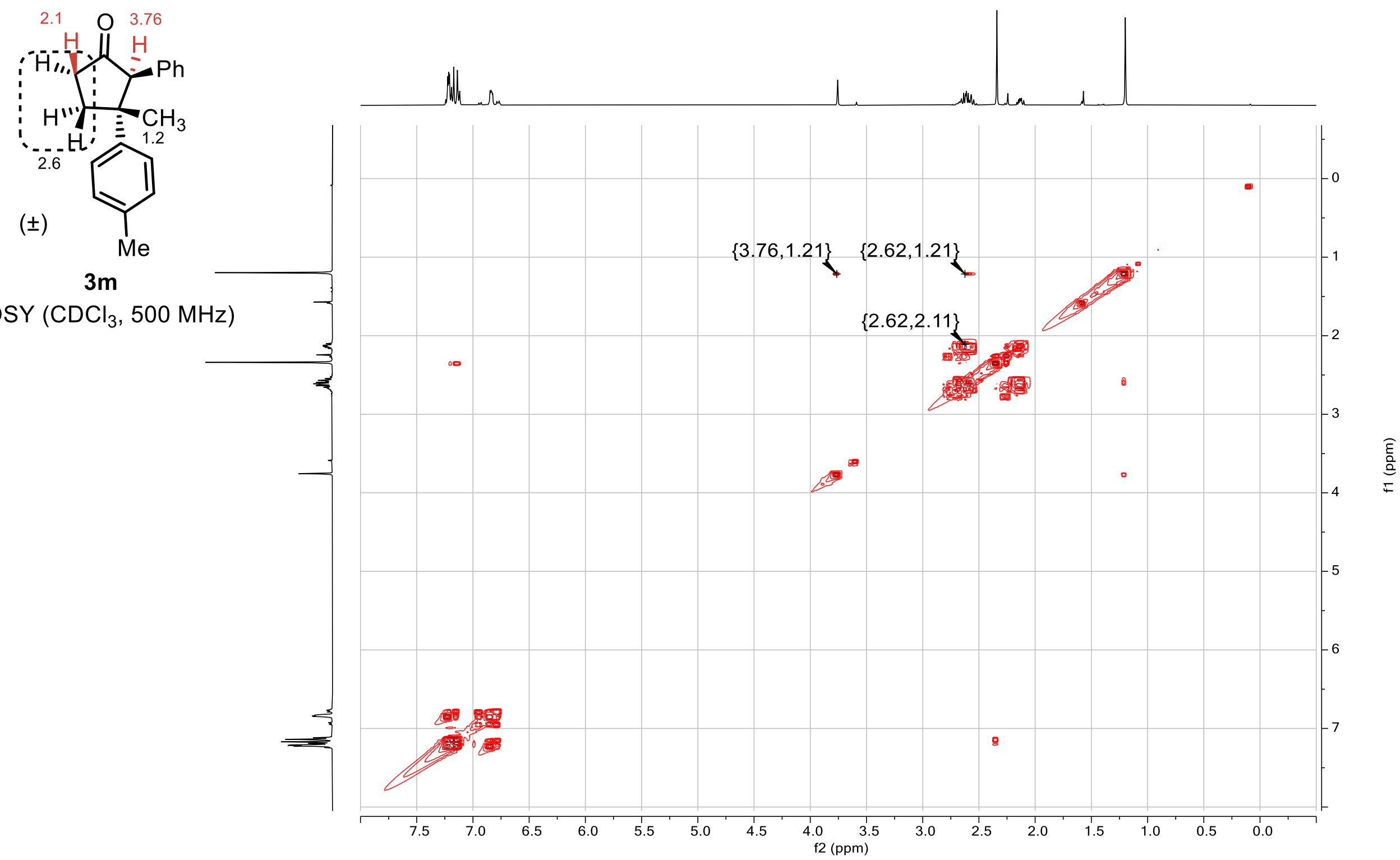


**3m**
COSY ($CDCl_3$, 500 MHz)

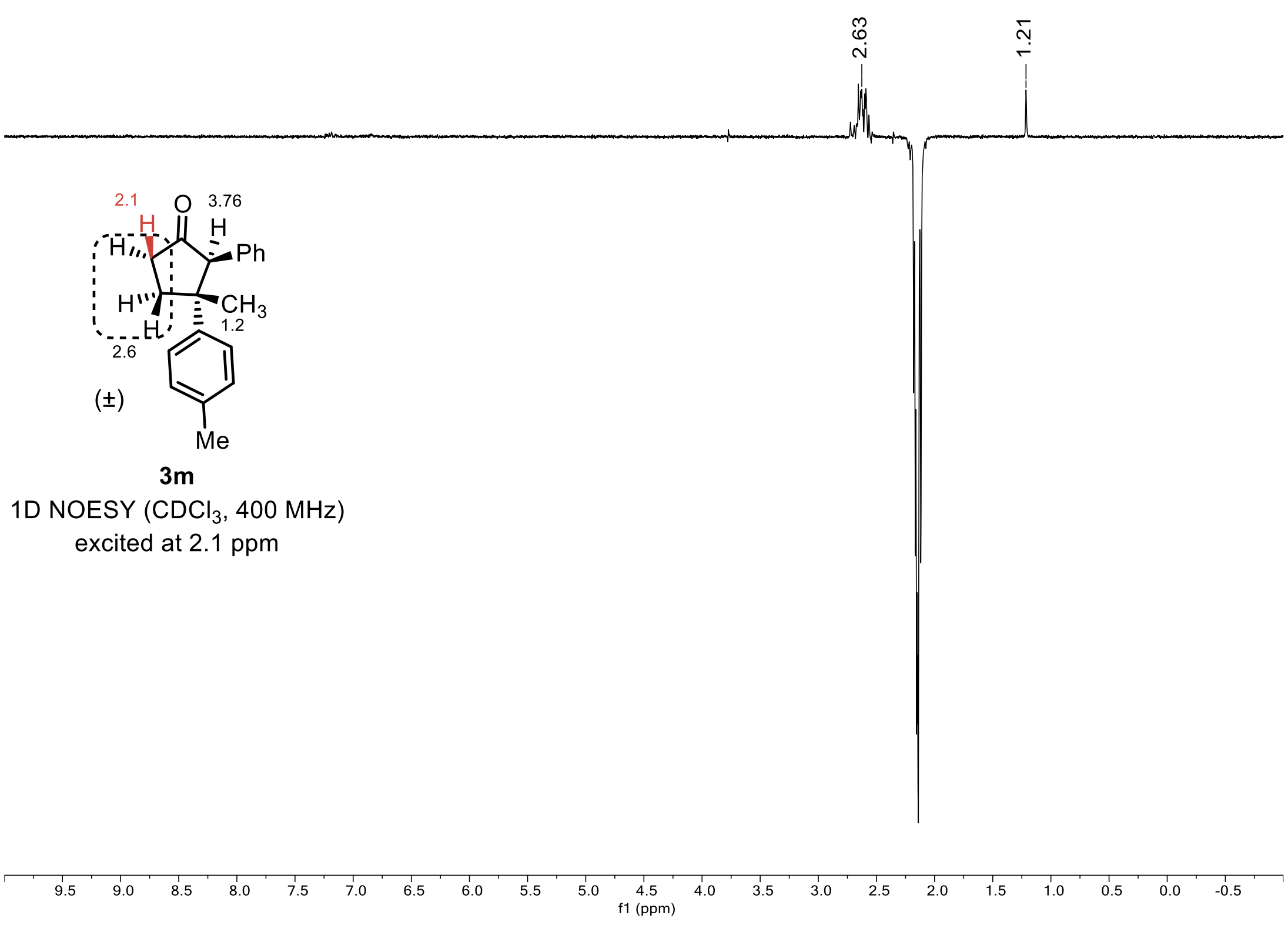


**3m**
1D NOESY ($CDCl_3$, 400 MHz)
excited at 2.1 ppm

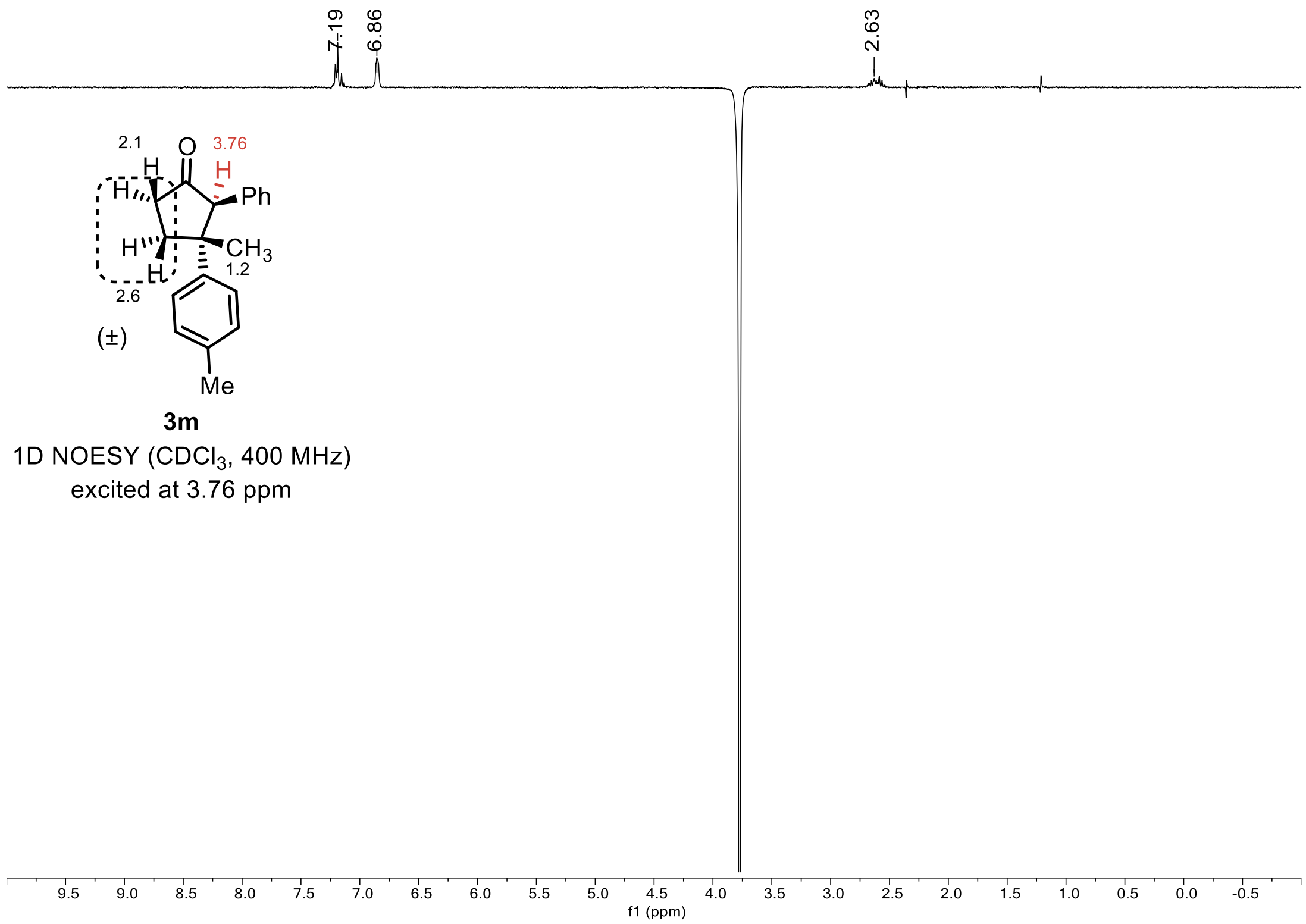


**3m**
1D NOESY ($CDCl_3$, 400 MHz)
excited at 3.76 ppm

## 3-(4-Methoxyphenyl)-4-methyl-2-phenylcyclopentan-1-one (3o).

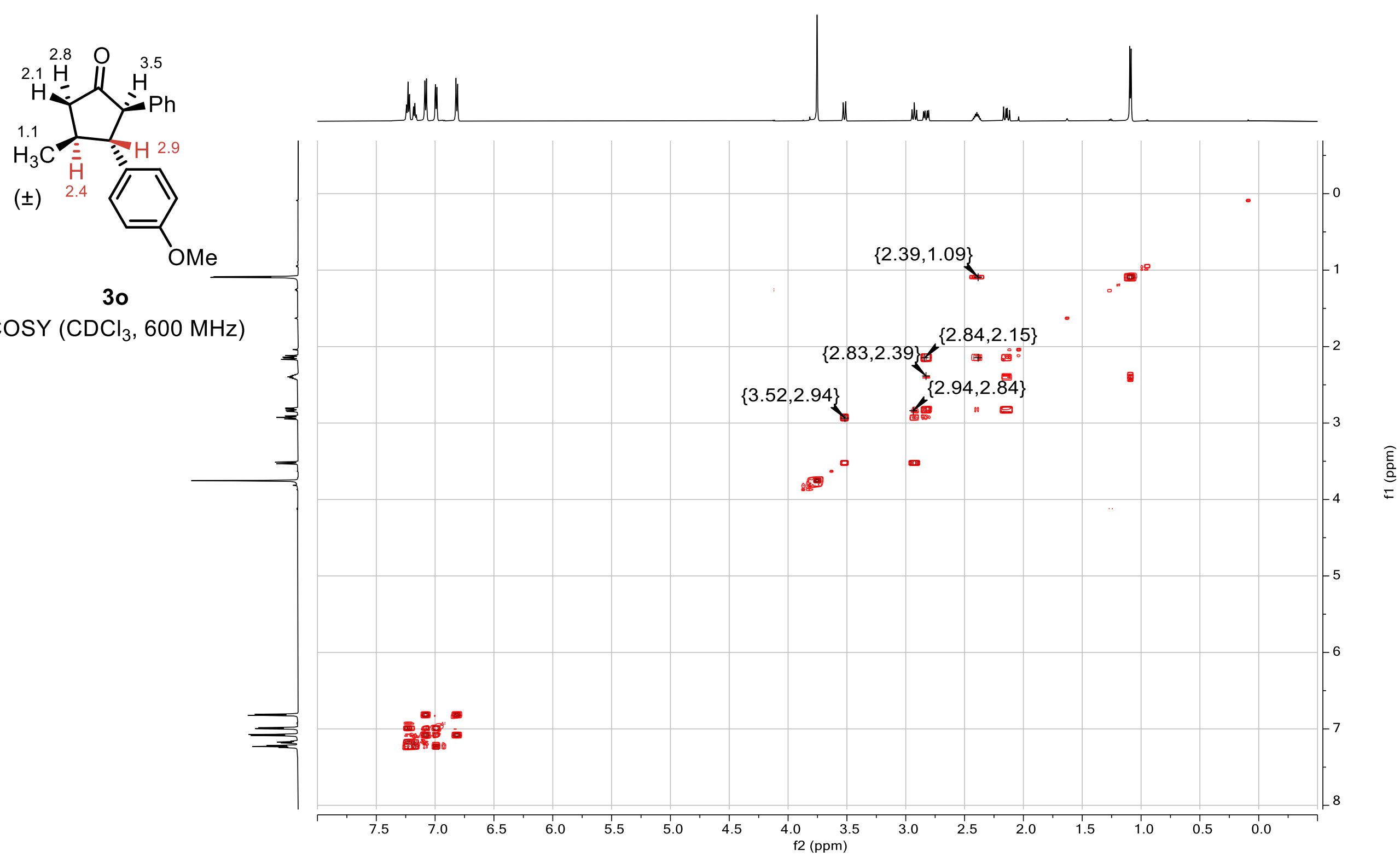


**3o**
COSY ($CDCl_3$, 600 MHz)

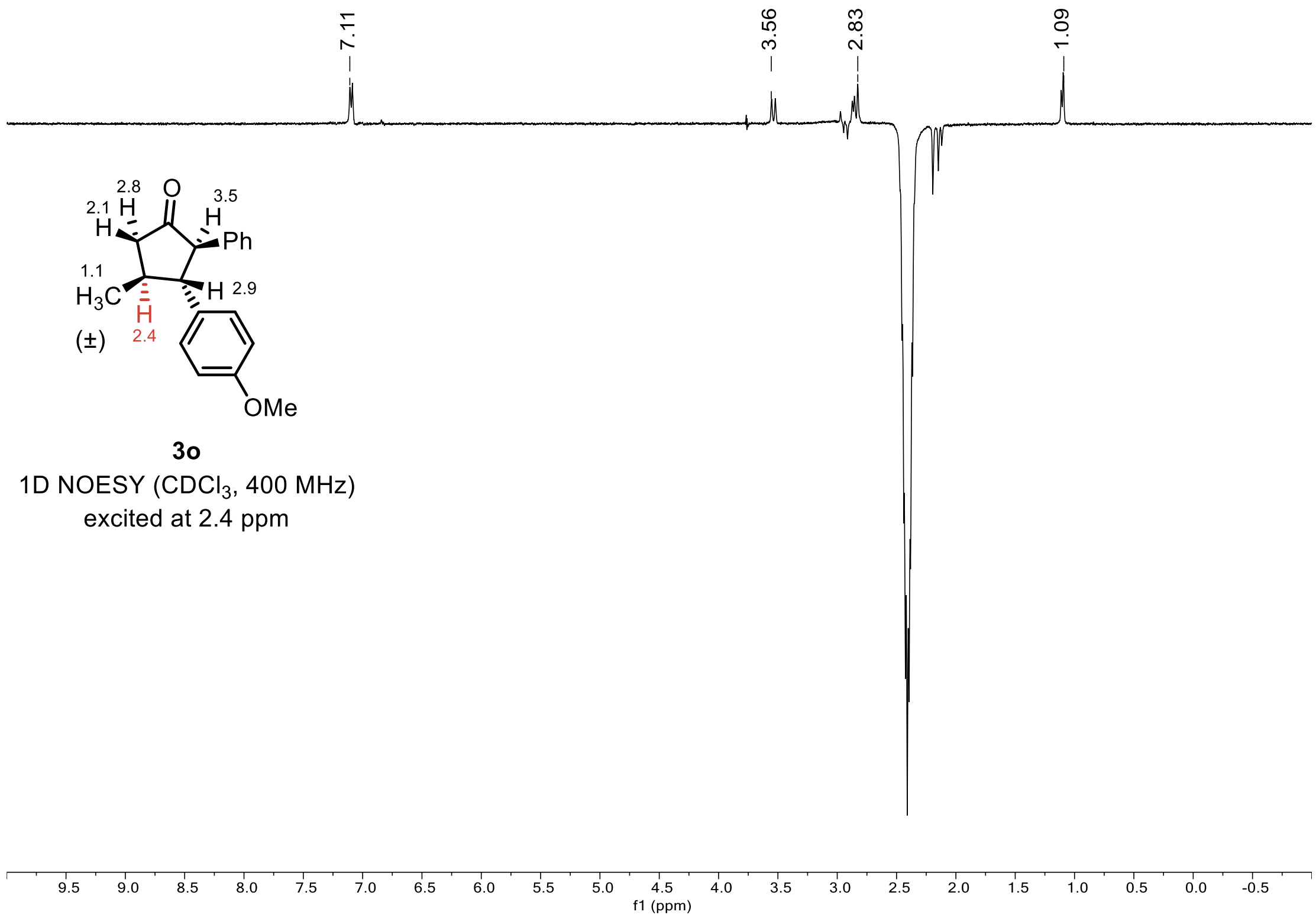


**3o**
1D NOESY ($CDCl_3$, 400 MHz)
excited at 2.4 ppm

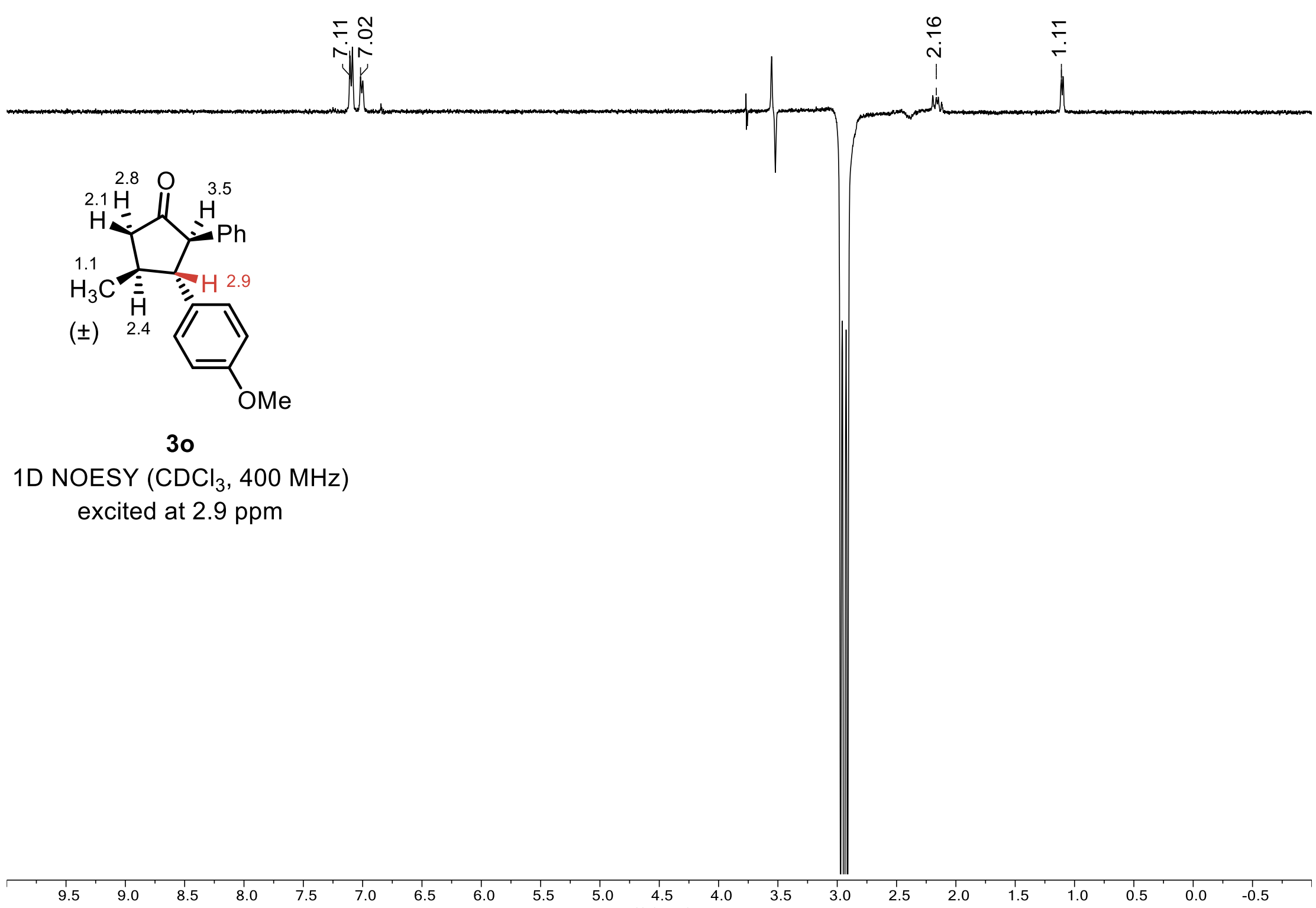


**3o**
1D NOESY ($CDCl_3$, 400 MHz)
excited at 2.9 ppm

**3-(4-Methoxyphenyl)-4-methyl-2,5-diphenylcyclopentan-1-one (3u).**

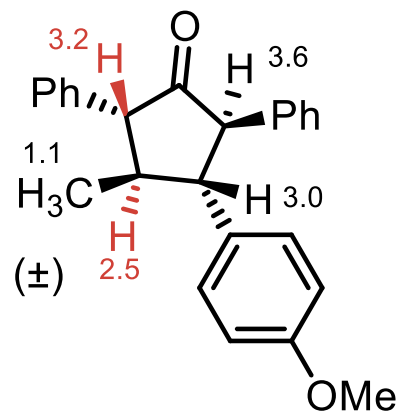


**3u**

COSY ($CDCl_3$, 400 MHz)

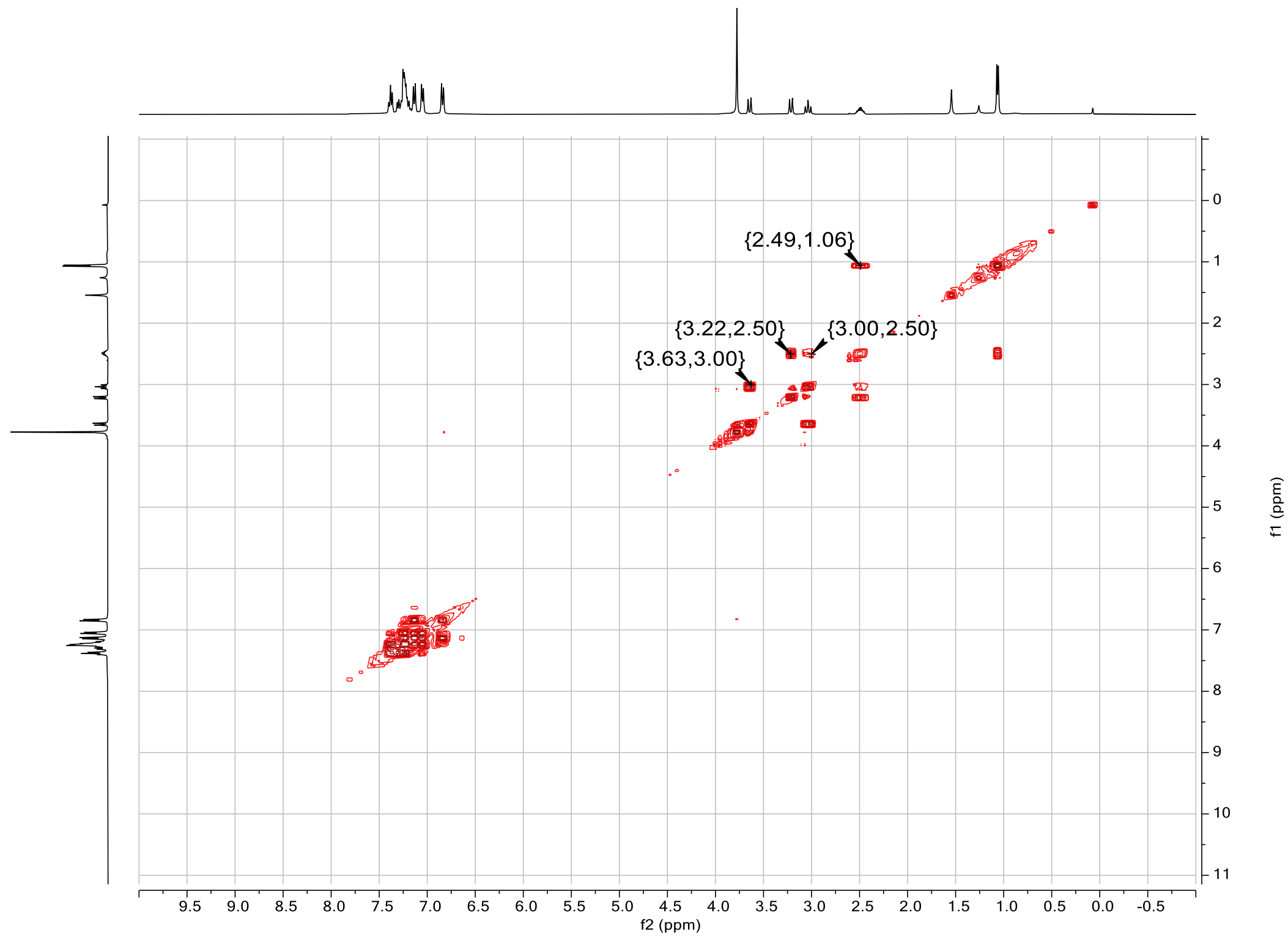


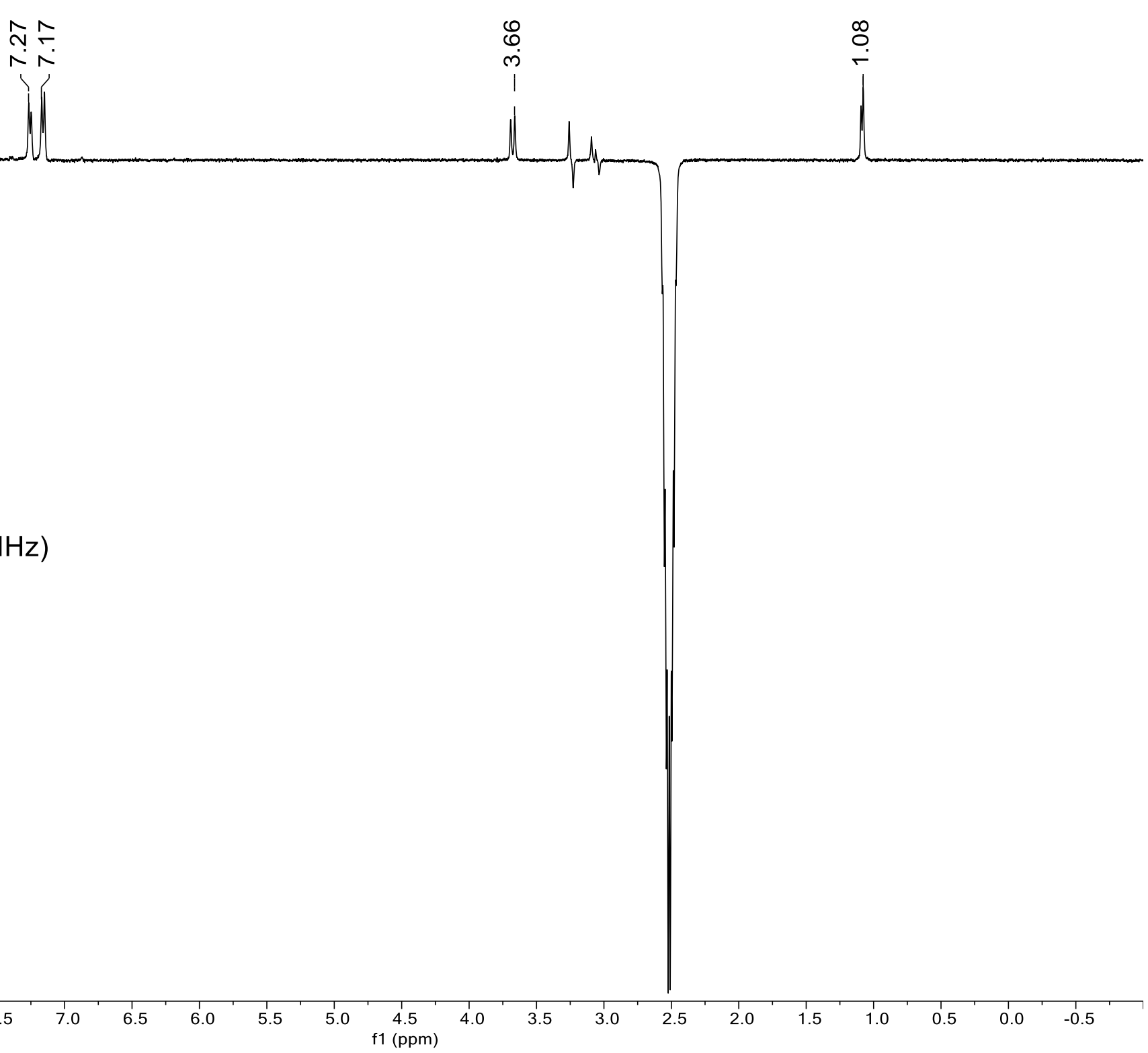


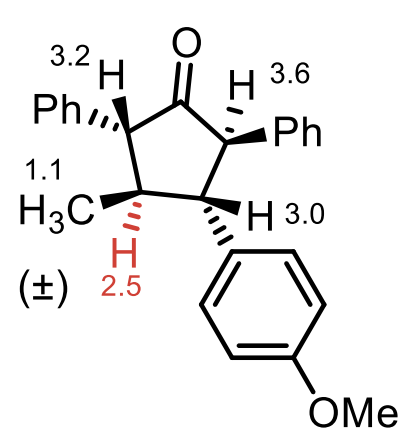


**3u**

1D NOESY ($CDCl_3$, 400 MHz)

excited at 2.5 ppm

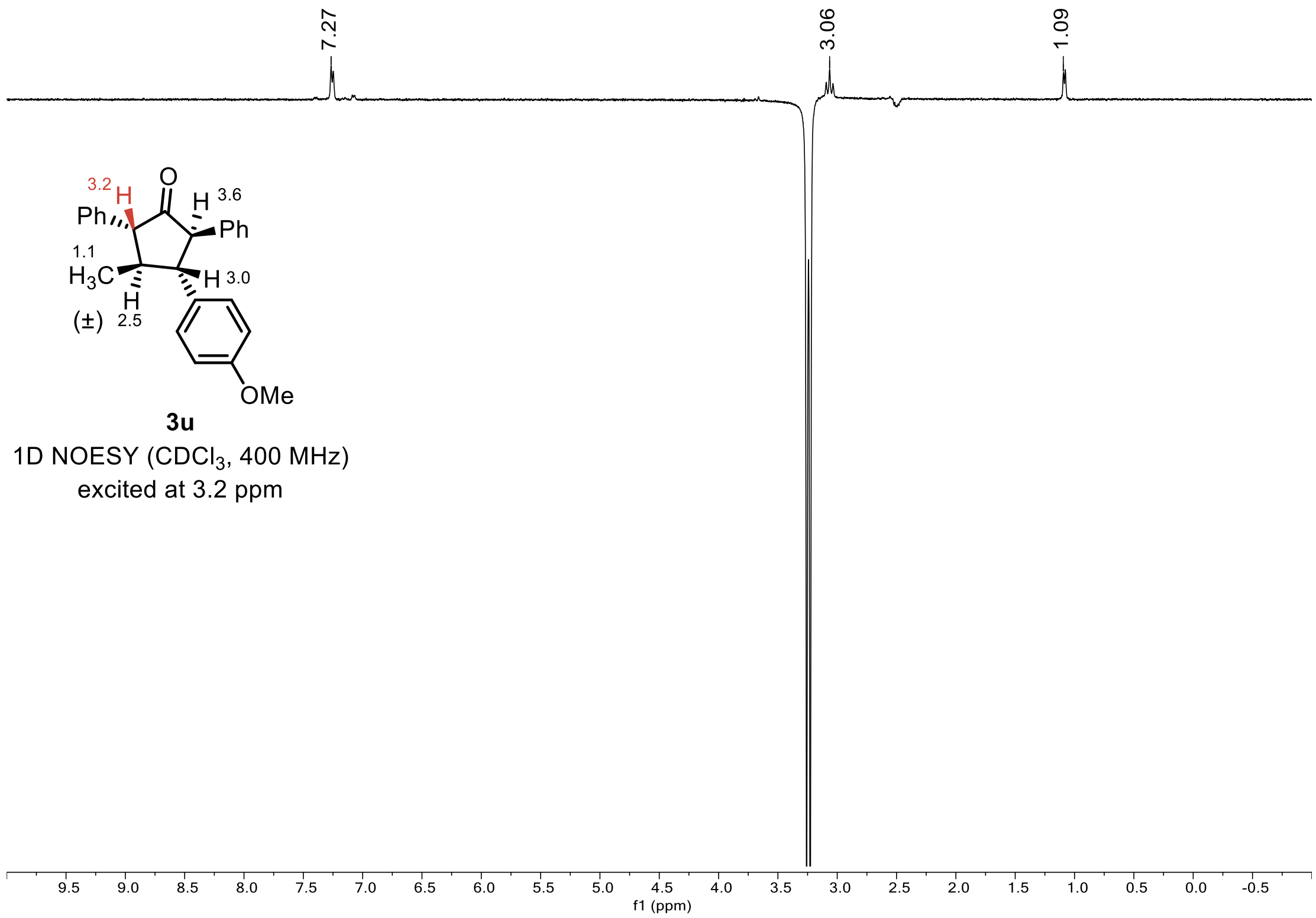


**3u**

1D NOESY ($CDCl_3$, 400 MHz)

excited at 3.2 ppm

## 7. X-Ray Crystallographic Data for Relative Stereochemistry

**3-(4-Methoxyphenyl)-4-methyl-2-phenylcyclopentan-1-ol (6)**.

### Crystal Growth

Single crystals of **6** were achieved via dissolving 6.0 mg of the pure compound in 10 drops of *n*-pentane at 55 °C and gradually allowing the solution to cool to room temperature overnight.

### Experimental

Low-temperature diffraction data (ω-scans) were collected on a Rigaku MicroMax-007HF diffractometer coupled to a Dectris Pilatus3R detector with Mo Kα (λ = 0.71073 Å) for the structure of **6**. The diffraction images were processed and scaled using Rigaku Oxford Diffraction software (CrysAlisPro; Rigaku OD: The Woodlands, TX, 2015). The structure was solved with SHELXT and was refined against $F^2$ on all data by full-matrix least squares with SHELXL (Sheldrick, G. M. Acta Cryst. 2008, A64, 112–122). All non-hydrogen atoms were refined anisotropically. Hydrogen atoms were included in the model at geometrically calculated positions and refined using a riding model, except for the O-H hydrogen which was refined semi-freely. The isotropic displacement parameters of all hydrogen atoms were fixed to 1.2 times the U value of the atoms to which they are linked (1.5 times for methyl groups).

The compound crystallizes as thin plates that are highly prone to twinning. Analysis of the reflections allowed for the indexing of two twin components related by a nearly 180-degree rotation. Many reflections were partially overlapping, resulting in high values of R(int) and R1.

The full numbering scheme of compound **6** can be found in the full details of the X-ray structure determination (CIF), which is included as Supporting Information. CCDC number 2566079 (007c-26114) contains the supplementary crystallographic data for this paper. These data can be obtained free of charge from The Cambridge Crystallographic Data Center via www.ccdc.cam.ac.uk/data_request/cif.

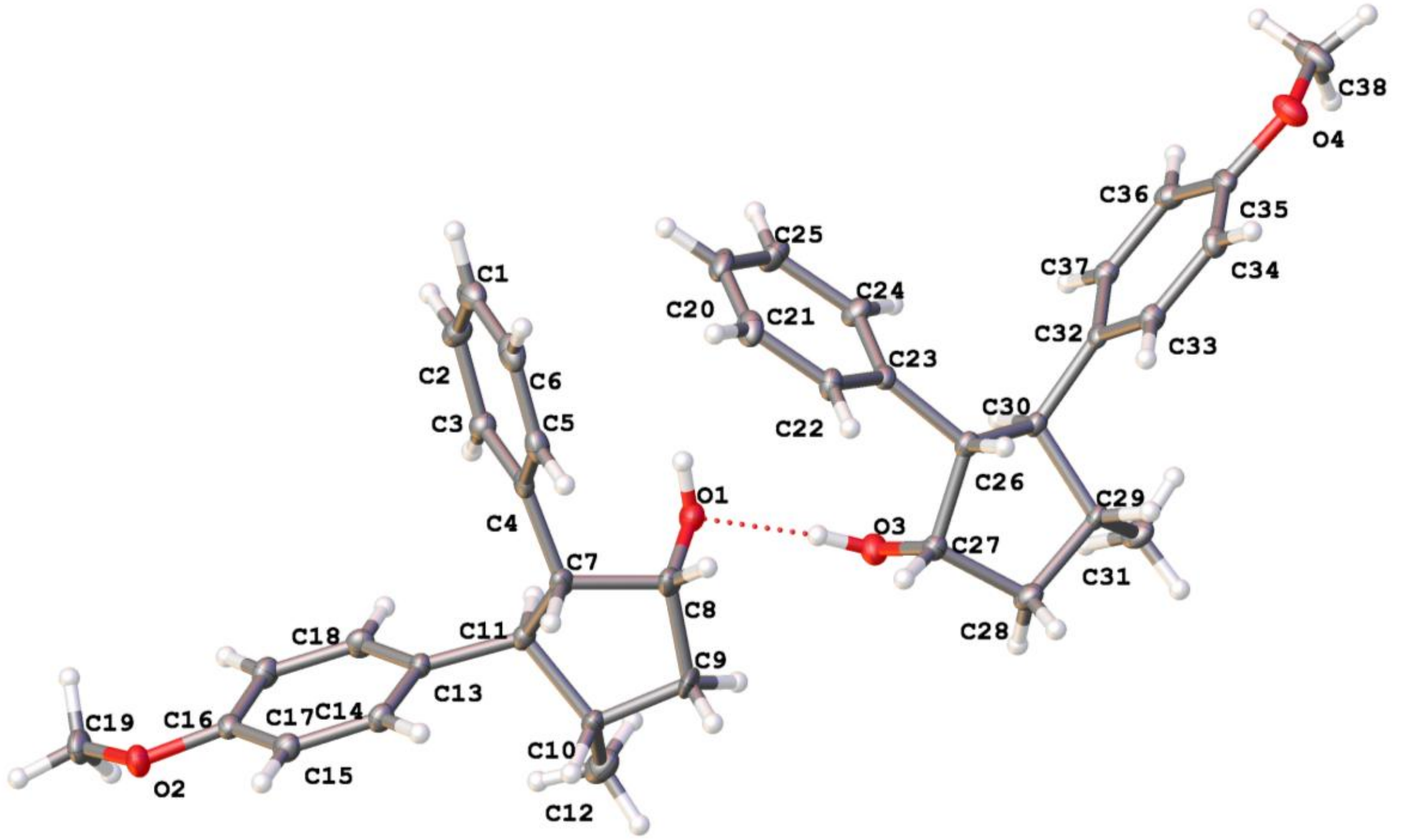


**Figure S9**. The complete numbering scheme of **6** with 50% thermal ellipsoid probability levels. The hydrogen atoms are shown as circles for clarity.

**Table S1**. Crystal data and structure refinement for **6**.

| | | |
|---|---|---|
| Identification code | 007c-26114 | |
| Empirical formula | C19 H22 O2 | |
| Formula weight | 282.36 | |
| Temperature | 99.9(2) K | |
| Wavelength | 0.71073 Å | |
| Crystal system | Monoclinic | |
| Space group | I 1 2/a 1 | |
| Unit cell dimensions | a = 21.6853(10) Å | $\alpha$= 90°. |
| | b = 5.7916(3) Å | $\beta$= 99.704(4)°. |
| | c = 49.897(2) Å | $\gamma$ = 90°. |
| Volume | 6177.1(5) Å$^3$ | |
| Z | 16 | |
| Density (calculated) | 1.214 Mg/m$^3$ | |
| Absorption coefficient | 0.077 mm$^{-1}$ | |
| F(000) | 2432 | |
| Crystal size | 0.19 x 0.09 x 0.01 mm$^3$ | |
| Crystal color and habit | colourless plate | |
| Diffractometer | XtaLAB AFC12 (RCD3): Kappa single | |
| Theta range for data collection | 2.728 to 30.924°. | |
| Index ranges | -30<=h<=29, -8<=k<=7, -70<=l<=67 | |
| Reflections collected | 93611 | |
| Independent reflections | 8763 [R(int) = 0.4465] | |
| Observed reflections (I > 2sigma(I)) | 5713 | |
| Completeness to theta = 25.242° | 99.7 % | |
| Absorption correction | Semi-empirical from equivalents | |
| Max. and min. transmission | 1.00000 and 0.54425 | |
| Solution method | SHELXT 2018/2 (Sheldrick, 2018) | |
| Refinement method | SHELXL 2019/3 (Sheldrick, 2015) | |
| Data / restraints / parameters | 8763 / 2 / 389 | |
| Goodness-of-fit on F$^2$ | 1.152 | |
| Final R indices [I>2sigma(I)] | R1 = 0.1237, wR2 = 0.2305 | |
| R indices (all data) | R1 = 0.1835, wR2 = 0.2539 | |
| Largest diff. peak and hole | 0.241 and -0.391 e.Å$^{-3}$ | |

**Table S2**. Atomic coordinates (x $10^4$) and equivalent isotropic displacement parameters ($Å^2$x $10^3$) for **6**. U(eq) is defined as one third of the trace of the orthogonalized $U^{ij}$ tensor.

| | x | y | z | U(eq) |
|---|---|---|---|---|
| O(1) | 2530(1) | 5449(4) | 3682(1) | 21(1) |
| O(2) | -932(1) | 7308(4) | 2615(1) | 22(1) |
| C(1) | 2913(1) | 9384(6) | 2851(1) | 23(1) |
| C(2) | 2599(1) | 7302(5) | 2852(1) | 20(1) |
| C(3) | 2200(1) | 6895(5) | 3039(1) | 18(1) |
| C(4) | 2115(1) | 8577(5) | 3230(1) | 17(1) |
| C(5) | 2429(1) | 10669(6) | 3224(1) | 21(1) |
| C(6) | 2826(1) | 11076(6) | 3038(1) | 23(1) |
| C(7) | 1741(1) | 8142(5) | 3454(1) | 18(1) |
| C(8) | 2158(1) | 7438(6) | 3723(1) | 22(1) |
| C(9) | 1688(2) | 6714(8) | 3902(1) | 32(1) |
| C(10) | 1075(1) | 6059(6) | 3710(1) | 20(1) |
| C(11) | 1244(1) | 6235(5) | 3421(1) | 16(1) |
| C(12) | 811(2) | 3742(6) | 3776(1) | 28(1) |
| C(13) | 685(1) | 6563(5) | 3198(1) | 14(1) |
| C(14) | 312(1) | 8538(5) | 3178(1) | 17(1) |
| C(15) | -218(1) | 8753(5) | 2981(1) | 18(1) |
| C(16) | -392(1) | 6964(5) | 2798(1) | 16(1) |
| C(17) | -25(1) | 5000(5) | 2808(1) | 19(1) |
| C(18) | 507(1) | 4829(5) | 3007(1) | 18(1) |
| C(19) | -1148(2) | 5438(6) | 2437(1) | 25(1) |
| O(3) | 3031(1) | 3147(4) | 4175(1) | 21(1) |
| O(4) | 6994(1) | 2688(4) | 4642(1) | 26(1) |
| C(20) | 3998(1) | 6545(6) | 3428(1) | 23(1) |
| C(21) | 3833(2) | 8276(6) | 3593(1) | 23(1) |
| C(22) | 3857(1) | 7857(5) | 3871(1) | 18(1) |
| C(23) | 4048(1) | 5724(5) | 3984(1) | 14(1) |
| C(24) | 4225(1) | 4022(5) | 3818(1) | 20(1) |
| C(25) | 4196(2) | 4432(6) | 3538(1) | 23(1) |
| C(26) | 4017(1) | 5308(5) | 4282(1) | 14(1) |
| C(27) | 3339(1) | 4970(5) | 4335(1) | 17(1) |

| | | | | |
|---|---|---|---|---|
| C(28) | 3439(1) | 4211(5) | 4634(1) | 19(1) |
| C(29) | 4094(1) | 3078(5) | 4694(1) | 15(1) |
| C(30) | 4341(1) | 3163(5) | 4419(1) | 14(1) |
| C(31) | 4104(2) | 645(6) | 4808(1) | 22(1) |
| C(32) | 5048(1) | 3075(5) | 4457(1) | 13(1) |
| C(33) | 5416(1) | 4880(5) | 4580(1) | 16(1) |
| C(34) | 6061(1) | 4706(5) | 4637(1) | 18(1) |
| C(35) | 6356(1) | 2705(5) | 4570(1) | 17(1) |
| C(36) | 6002(1) | 901(5) | 4441(1) | 18(1) |
| C(37) | 5351(1) | 1115(5) | 4384(1) | 15(1) |
| C(38) | 7304(2) | 563(6) | 4619(1) | 30(1) |

**Table S3**. Bond lengths [Å] and angles [°] for **6**.

| | |
|---|---|
| O(1)-C(8) | 1.440(4) |
| O(1)-H(1) | 0.846(19) |
| O(2)-C(16) | 1.373(3) |
| O(2)-C(19) | 1.428(4) |
| C(1)-H(1A) | 0.9500 |
| C(1)-C(2) | 1.385(5) |
| C(1)-C(6) | 1.388(5) |
| C(2)-H(2) | 0.9500 |
| C(2)-C(3) | 1.395(4) |
| C(3)-H(3) | 0.9500 |
| C(3)-C(4) | 1.395(4) |
| C(4)-C(5) | 1.392(4) |
| C(4)-C(7) | 1.510(4) |
| C(5)-H(5) | 0.9500 |
| C(5)-C(6) | 1.391(4) |
| C(6)-H(6) | 0.9500 |
| C(7)-H(7) | 1.0000 |
| C(7)-C(8) | 1.542(4) |
| C(7)-C(11) | 1.532(4) |
| C(8)-H(8) | 1.0000 |
| C(8)-C(9) | 1.525(4) |
| C(9)-H(9A) | 0.9900 |
| C(9)-H(9B) | 0.9900 |
| C(9)-C(10) | 1.549(4) |
| C(10)-H(10) | 1.0000 |
| C(10)-C(11) | 1.551(4) |
| C(10)-C(12) | 1.516(5) |
| C(11)-H(11) | 1.0000 |
| C(11)-C(13) | 1.513(4) |
| C(12)-H(12A) | 0.9800 |
| C(12)-H(12B) | 0.9800 |
| C(12)-H(12C) | 0.9800 |
| C(13)-C(14) | 1.395(4) |
| C(13)-C(18) | 1.392(4) |

| | |
|---|---|
| C(14)-H(14) | 0.9500 |
| C(14)-C(15) | 1.387(4) |
| C(15)-H(15) | 0.9500 |
| C(15)-C(16) | 1.392(4) |
| C(16)-C(17) | 1.385(4) |
| C(17)-H(17) | 0.9500 |
| C(17)-C(18) | 1.393(4) |
| C(18)-H(18) | 0.9500 |
| C(19)-H(19A) | 0.9800 |
| C(19)-H(19B) | 0.9800 |
| C(19)-H(19C) | 0.9800 |
| O(3)-H(3A) | 0.853(19) |
| O(3)-C(27) | 1.423(4) |
| O(4)-C(35) | 1.369(4) |
| O(4)-C(38) | 1.417(4) |
| C(20)-H(20) | 0.9500 |
| C(20)-C(21) | 1.384(5) |
| C(20)-C(25) | 1.379(5) |
| C(21)-H(21) | 0.9500 |
| C(21)-C(22) | 1.398(4) |
| C(22)-H(22) | 0.9500 |
| C(22)-C(23) | 1.393(4) |
| C(23)-C(24) | 1.383(4) |
| C(23)-C(26) | 1.518(3) |
| C(24)-H(24) | 0.9500 |
| C(24)-C(25) | 1.411(4) |
| C(25)-H(25) | 0.9500 |
| C(26)-H(26) | 1.0000 |
| C(26)-C(27) | 1.550(4) |
| C(26)-C(30) | 1.529(4) |
| C(27)-H(27) | 1.0000 |
| C(27)-C(28) | 1.534(4) |
| C(28)-H(28A) | 0.9900 |
| C(28)-H(28B) | 0.9900 |
| C(28)-C(29) | 1.548(4) |
| C(29)-H(29) | 1.0000 |

| | |
|---|---|
| C(29)-C(30) | 1.557(3) |
| C(29)-C(31) | 1.518(4) |
| C(30)-H(30) | 1.0000 |
| C(30)-C(32) | 1.515(4) |
| C(31)-H(31A) | 0.9800 |
| C(31)-H(31B) | 0.9800 |
| C(31)-H(31C) | 0.9800 |
| C(32)-C(33) | 1.394(4) |
| C(32)-C(37) | 1.390(4) |
| C(33)-H(33) | 0.9500 |
| C(33)-C(34) | 1.385(4) |
| C(34)-H(34) | 0.9500 |
| C(34)-C(35) | 1.391(4) |
| C(35)-C(36) | 1.388(4) |
| C(36)-H(36) | 0.9500 |
| C(36)-C(37) | 1.398(4) |
| C(37)-H(37) | 0.9500 |
| C(38)-H(38A) | 0.9800 |
| C(38)-H(38B) | 0.9800 |
| C(38)-H(38C) | 0.9800 |

| | |
|---|---|
| C(8)-O(1)-H(1) | 106(3) |
| C(16)-O(2)-C(19) | 117.2(2) |
| C(2)-C(1)-H(1A) | 120.4 |
| C(2)-C(1)-C(6) | 119.2(3) |
| C(6)-C(1)-H(1A) | 120.4 |
| C(1)-C(2)-H(2) | 119.6 |
| C(1)-C(2)-C(3) | 120.8(3) |
| C(3)-C(2)-H(2) | 119.6 |
| C(2)-C(3)-H(3) | 119.8 |
| C(2)-C(3)-C(4) | 120.5(3) |
| C(4)-C(3)-H(3) | 119.8 |
| C(3)-C(4)-C(7) | 122.6(3) |
| C(5)-C(4)-C(3) | 118.2(3) |
| C(5)-C(4)-C(7) | 119.0(3) |
| C(4)-C(5)-H(5) | 119.3 |

| | |
|---|---|
| C(6)-C(5)-C(4) | 121.4(3) |
| C(6)-C(5)-H(5) | 119.3 |
| C(1)-C(6)-C(5) | 120.0(3) |
| C(1)-C(6)-H(6) | 120.0 |
| C(5)-C(6)-H(6) | 120.0 |
| C(4)-C(7)-H(7) | 107.5 |
| C(4)-C(7)-C(8) | 112.3(2) |
| C(4)-C(7)-C(11) | 119.7(2) |
| C(8)-C(7)-H(7) | 107.5 |
| C(11)-C(7)-H(7) | 107.5 |
| C(11)-C(7)-C(8) | 101.7(2) |
| O(1)-C(8)-C(7) | 110.4(2) |
| O(1)-C(8)-H(8) | 111.6 |
| O(1)-C(8)-C(9) | 108.0(3) |
| C(7)-C(8)-H(8) | 111.6 |
| C(9)-C(8)-C(7) | 103.3(2) |
| C(9)-C(8)-H(8) | 111.6 |
| C(8)-C(9)-H(9A) | 110.3 |
| C(8)-C(9)-H(9B) | 110.3 |
| C(8)-C(9)-C(10) | 107.1(2) |
| H(9A)-C(9)-H(9B) | 108.6 |
| C(10)-C(9)-H(9A) | 110.3 |
| C(10)-C(9)-H(9B) | 110.3 |
| C(9)-C(10)-H(10) | 108.1 |
| C(9)-C(10)-C(11) | 104.3(2) |
| C(11)-C(10)-H(10) | 108.1 |
| C(12)-C(10)-C(9) | 113.5(3) |
| C(12)-C(10)-H(10) | 108.1 |
| C(12)-C(10)-C(11) | 114.6(3) |
| C(7)-C(11)-C(10) | 102.7(2) |
| C(7)-C(11)-H(11) | 107.6 |
| C(10)-C(11)-H(11) | 107.6 |
| C(13)-C(11)-C(7) | 116.8(2) |
| C(13)-C(11)-C(10) | 114.0(2) |
| C(13)-C(11)-H(11) | 107.6 |
| C(10)-C(12)-H(12A) | 109.5 |

| | |
|---|---|
| C(10)-C(12)-H(12B) | 109.5 |
| C(10)-C(12)-H(12C) | 109.5 |
| H(12A)-C(12)-H(12B) | 109.5 |
| H(12A)-C(12)-H(12C) | 109.5 |
| H(12B)-C(12)-H(12C) | 109.5 |
| C(14)-C(13)-C(11) | 122.4(2) |
| C(18)-C(13)-C(11) | 120.5(3) |
| C(18)-C(13)-C(14) | 117.0(3) |
| C(13)-C(14)-H(14) | 119.2 |
| C(15)-C(14)-C(13) | 121.6(3) |
| C(15)-C(14)-H(14) | 119.2 |
| C(14)-C(15)-H(15) | 120.0 |
| C(14)-C(15)-C(16) | 120.1(3) |
| C(16)-C(15)-H(15) | 120.0 |
| O(2)-C(16)-C(15) | 115.9(3) |
| O(2)-C(16)-C(17) | 124.4(3) |
| C(17)-C(16)-C(15) | 119.7(3) |
| C(16)-C(17)-H(17) | 120.4 |
| C(16)-C(17)-C(18) | 119.1(3) |
| C(18)-C(17)-H(17) | 120.4 |
| C(13)-C(18)-C(17) | 122.5(3) |
| C(13)-C(18)-H(18) | 118.7 |
| C(17)-C(18)-H(18) | 118.7 |
| O(2)-C(19)-H(19A) | 109.5 |
| O(2)-C(19)-H(19B) | 109.5 |
| O(2)-C(19)-H(19C) | 109.5 |
| H(19A)-C(19)-H(19B) | 109.5 |
| H(19A)-C(19)-H(19C) | 109.5 |
| H(19B)-C(19)-H(19C) | 109.5 |
| C(27)-O(3)-H(3A) | 108(3) |
| C(35)-O(4)-C(38) | 117.3(3) |
| C(21)-C(20)-H(20) | 120.0 |
| C(25)-C(20)-H(20) | 120.0 |
| C(25)-C(20)-C(21) | 119.9(3) |
| C(20)-C(21)-H(21) | 120.1 |
| C(20)-C(21)-C(22) | 119.7(3) |

| | |
|---|---|
| C(22)-C(21)-H(21) | 120.1 |
| C(21)-C(22)-H(22) | 119.5 |
| C(23)-C(22)-C(21) | 121.0(3) |
| C(23)-C(22)-H(22) | 119.5 |
| C(22)-C(23)-C(26) | 118.7(2) |
| C(24)-C(23)-C(22) | 118.8(2) |
| C(24)-C(23)-C(26) | 122.4(3) |
| C(23)-C(24)-H(24) | 119.9 |
| C(23)-C(24)-C(25) | 120.3(3) |
| C(25)-C(24)-H(24) | 119.9 |
| C(20)-C(25)-C(24) | 120.2(3) |
| C(20)-C(25)-H(25) | 119.9 |
| C(24)-C(25)-H(25) | 119.9 |
| C(23)-C(26)-H(26) | 108.0 |
| C(23)-C(26)-C(27) | 112.8(2) |
| C(23)-C(26)-C(30) | 118.2(2) |
| C(27)-C(26)-H(26) | 108.0 |
| C(30)-C(26)-H(26) | 108.0 |
| C(30)-C(26)-C(27) | 101.4(2) |
| O(3)-C(27)-C(26) | 111.5(2) |
| O(3)-C(27)-H(27) | 111.4 |
| O(3)-C(27)-C(28) | 108.1(2) |
| C(26)-C(27)-H(27) | 111.4 |
| C(28)-C(27)-C(26) | 102.8(2) |
| C(28)-C(27)-H(27) | 111.4 |
| C(27)-C(28)-H(28A) | 110.4 |
| C(27)-C(28)-H(28B) | 110.4 |
| C(27)-C(28)-C(29) | 106.4(2) |
| H(28A)-C(28)-H(28B) | 108.6 |
| C(29)-C(28)-H(28A) | 110.4 |
| C(29)-C(28)-H(28B) | 110.4 |
| C(28)-C(29)-H(29) | 108.3 |
| C(28)-C(29)-C(30) | 104.9(2) |
| C(30)-C(29)-H(29) | 108.3 |
| C(31)-C(29)-C(28) | 114.8(2) |
| C(31)-C(29)-H(29) | 108.3 |

| | |
|---|---|
| C(31)-C(29)-C(30) | 112.0(2) |
| C(26)-C(30)-C(29) | 102.7(2) |
| C(26)-C(30)-H(30) | 108.0 |
| C(29)-C(30)-H(30) | 108.0 |
| C(32)-C(30)-C(26) | 117.5(2) |
| C(32)-C(30)-C(29) | 112.3(2) |
| C(32)-C(30)-H(30) | 108.0 |
| C(29)-C(31)-H(31A) | 109.5 |
| C(29)-C(31)-H(31B) | 109.5 |
| C(29)-C(31)-H(31C) | 109.5 |
| H(31A)-C(31)-H(31B) | 109.5 |
| H(31A)-C(31)-H(31C) | 109.5 |
| H(31B)-C(31)-H(31C) | 109.5 |
| C(33)-C(32)-C(30) | 121.4(3) |
| C(37)-C(32)-C(30) | 120.5(3) |
| C(37)-C(32)-C(33) | 117.9(3) |
| C(32)-C(33)-H(33) | 119.4 |
| C(34)-C(33)-C(32) | 121.3(3) |
| C(34)-C(33)-H(33) | 119.4 |
| C(33)-C(34)-H(34) | 120.0 |
| C(33)-C(34)-C(35) | 120.0(3) |
| C(35)-C(34)-H(34) | 120.0 |
| O(4)-C(35)-C(34) | 115.3(3) |
| O(4)-C(35)-C(36) | 124.7(3) |
| C(36)-C(35)-C(34) | 119.9(3) |
| C(35)-C(36)-H(36) | 120.4 |
| C(35)-C(36)-C(37) | 119.2(3) |
| C(37)-C(36)-H(36) | 120.4 |
| C(32)-C(37)-C(36) | 121.6(3) |
| C(32)-C(37)-H(37) | 119.2 |
| C(36)-C(37)-H(37) | 119.2 |
| O(4)-C(38)-H(38A) | 109.5 |
| O(4)-C(38)-H(38B) | 109.5 |
| O(4)-C(38)-H(38C) | 109.5 |
| H(38A)-C(38)-H(38B) | 109.5 |
| H(38A)-C(38)-H(38C) | 109.5 |

| | |
|---|---|
| H(38B)-C(38)-H(38C) | 109.5 |

**Table S4**. Anisotropic displacement parameters ($Å^2$x $10^3$) for **6**. The anisotropic displacement factor exponent takes the form: $-2p^2[h^2a^{*2}U^{11} + ... + 2hka^*b^*U^{12}]$

| | $U^{11}$ | $U^{22}$ | $U^{33}$ | $U^{23}$ | $U^{13}$ | $U^{12}$ |
|---|---|---|---|---|---|---|
| O(1) | 19(1) | 30(1) | 15(1) | 3(1) | 1(1) | 1(1) |
| O(2) | 17(1) | 30(1) | 16(1) | -2(1) | -2(1) | 1(1) |
| C(1) | 19(1) | 27(2) | 22(1) | 10(1) | 3(1) | 2(1) |
| C(2) | 24(2) | 22(2) | 16(1) | 1(1) | 6(1) | 3(1) |
| C(3) | 18(1) | 18(1) | 16(1) | 1(1) | 2(1) | 0(1) |
| C(4) | 14(1) | 22(2) | 13(1) | 1(1) | -3(1) | 4(1) |
| C(5) | 19(1) | 21(2) | 20(1) | -1(1) | -4(1) | 2(1) |
| C(6) | 21(2) | 21(2) | 25(2) | 8(1) | -4(1) | -2(1) |
| C(7) | 17(1) | 23(2) | 13(1) | -5(1) | 1(1) | 3(1) |
| C(8) | 18(1) | 34(2) | 12(1) | -5(1) | -1(1) | 3(1) |
| C(9) | 23(2) | 67(3) | 7(1) | -2(1) | 0(1) | 2(2) |
| C(10) | 16(1) | 33(2) | 11(1) | -1(1) | 2(1) | 4(1) |
| C(11) | 17(1) | 19(2) | 11(1) | 0(1) | 2(1) | 4(1) |
| C(12) | 35(2) | 30(2) | 21(2) | 5(1) | 9(1) | 5(2) |
| C(13) | 14(1) | 18(1) | 11(1) | 0(1) | 1(1) | 1(1) |
| C(14) | 20(1) | 17(1) | 13(1) | -2(1) | 2(1) | 0(1) |
| C(15) | 20(1) | 17(1) | 16(1) | 1(1) | 3(1) | 3(1) |
| C(16) | 14(1) | 21(2) | 13(1) | 2(1) | 2(1) | -2(1) |
| C(17) | 22(1) | 19(2) | 15(1) | -3(1) | 2(1) | -2(1) |
| C(18) | 20(1) | 17(2) | 18(1) | -2(1) | 4(1) | 5(1) |
| C(19) | 20(2) | 37(2) | 17(1) | -3(1) | -4(1) | -5(1) |
| O(3) | 18(1) | 26(1) | 18(1) | 2(1) | 0(1) | -3(1) |
| O(4) | 15(1) | 29(1) | 34(1) | 5(1) | 3(1) | 1(1) |
| C(20) | 24(2) | 33(2) | 13(1) | 6(1) | 1(1) | -6(1) |
| C(21) | 24(2) | 24(2) | 20(1) | 9(1) | -1(1) | -5(1) |
| C(22) | 20(1) | 19(2) | 13(1) | 0(1) | -1(1) | -1(1) |
| C(23) | 12(1) | 17(1) | 14(1) | 2(1) | 1(1) | -2(1) |
| C(24) | 24(2) | 21(2) | 15(1) | 3(1) | 6(1) | 0(1) |
| C(25) | 30(2) | 28(2) | 13(1) | -2(1) | 6(1) | -2(1) |
| C(26) | 17(1) | 14(1) | 12(1) | -1(1) | 2(1) | 1(1) |
| C(27) | 16(1) | 22(2) | 13(1) | 0(1) | 2(1) | 1(1) |

| | | | | | | |
|---|---|---|---|---|---|---|
| C(28) | 22(1) | 22(2) | 15(1) | 3(1) | 7(1) | 2(1) |
| C(29) | 18(1) | 21(2) | 8(1) | 1(1) | 2(1) | 0(1) |
| C(30) | 16(1) | 16(1) | 10(1) | 0(1) | 2(1) | 1(1) |
| C(31) | 26(2) | 22(2) | 19(1) | 7(1) | 8(1) | 2(1) |
| C(32) | 15(1) | 15(1) | 9(1) | 3(1) | 1(1) | -1(1) |
| C(33) | 19(1) | 16(1) | 14(1) | -1(1) | 3(1) | -1(1) |
| C(34) | 18(1) | 18(2) | 15(1) | 1(1) | -1(1) | -5(1) |
| C(35) | 15(1) | 22(2) | 14(1) | 6(1) | 3(1) | 0(1) |
| C(36) | 20(1) | 20(2) | 17(1) | 2(1) | 7(1) | 1(1) |
| C(37) | 18(1) | 15(1) | 12(1) | 1(1) | 4(1) | -1(1) |
| C(38) | 16(2) | 29(2) | 46(2) | 13(2) | 6(1) | 5(1) |

**Table S5**. Hydrogen coordinates (x $10^4$) and isotropic displacement parameters ($Å^2$x $10^3$) for **6**.

| | x | y | z | U(eq) |
|---|---|---|---|---|
| H(1A) | 3184 | 9650 | 2723 | 27 |
| H(2) | 2656 | 6137 | 2724 | 25 |
| H(3) | 1985 | 5464 | 3037 | 21 |
| H(5) | 2371 | 11842 | 3351 | 25 |
| H(6) | 3037 | 12513 | 3038 | 28 |
| H(7) | 1529 | 9622 | 3488 | 21 |
| H(8) | 2425 | 8751 | 3805 | 27 |
| H(9A) | 1611 | 8001 | 4023 | 39 |
| H(9B) | 1848 | 5375 | 4017 | 39 |
| H(1) | 2796(17) | 5910(80) | 3588(8) | 49 |
| H(3A) | 2890(20) | 3670(80) | 4016(5) | 49 |
| H(10) | 755 | 7270 | 3727 | 24 |
| H(11) | 1454 | 4759 | 3384 | 19 |
| H(12A) | 462 | 3323 | 3632 | 42 |
| H(12B) | 661 | 3842 | 3950 | 42 |
| H(12C) | 1138 | 2563 | 3788 | 42 |
| H(14) | 424 | 9767 | 3303 | 20 |
| H(15) | -462 | 10121 | 2972 | 21 |
| H(17) | -134 | 3785 | 2681 | 23 |
| H(18) | 757 | 3478 | 3013 | 22 |
| H(19A) | -842 | 5121 | 2318 | 38 |
| H(19B) | -1551 | 5849 | 2326 | 38 |
| H(19C) | -1201 | 4059 | 2545 | 38 |
| H(20) | 3975 | 6811 | 3238 | 28 |
| H(21) | 3705 | 9742 | 3519 | 28 |
| H(22) | 3741 | 9045 | 3984 | 22 |
| H(24) | 4367 | 2571 | 3893 | 24 |
| H(25) | 4312 | 3249 | 3424 | 28 |
| H(26) | 4197 | 6692 | 4387 | 17 |
| H(27) | 3095 | 6440 | 4307 | 20 |
| H(28A) | 3112 | 3090 | 4665 | 23 |

| | | | | |
|---|---|---|---|---|
| H(28B) | 3419 | 5559 | 4755 | 23 |
| H(29) | 4371 | 4068 | 4828 | 19 |
| H(30) | 4173 | 1785 | 4309 | 17 |
| H(31A) | 3807 | -321 | 4686 | 33 |
| H(31B) | 3984 | 684 | 4988 | 33 |
| H(31C) | 4526 | 0 | 4821 | 33 |
| H(33) | 5219 | 6256 | 4626 | 20 |
| H(34) | 6303 | 5955 | 4722 | 21 |
| H(36) | 6201 | -462 | 4392 | 22 |
| H(37) | 5110 | -110 | 4293 | 18 |
| H(38A) | 7267 | 140 | 4427 | 45 |
| H(38B) | 7114 | -643 | 4716 | 45 |
| H(38C) | 7748 | 722 | 4698 | 45 |

**Table 6**. Torsion angles [°] for **6**.

| | |
|---|---|
| O(1)-C(8)-C(9)-C(10) | -94.3(3) |
| O(2)-C(16)-C(17)-C(18) | -178.6(3) |
| C(1)-C(2)-C(3)-C(4) | -0.6(4) |
| C(2)-C(1)-C(6)-C(5) | 0.2(5) |
| C(2)-C(3)-C(4)-C(5) | 1.1(4) |
| C(2)-C(3)-C(4)-C(7) | -173.7(3) |
| C(3)-C(4)-C(5)-C(6) | -1.0(4) |
| C(3)-C(4)-C(7)-C(8) | 95.8(3) |
| C(3)-C(4)-C(7)-C(11) | -23.3(4) |
| C(4)-C(5)-C(6)-C(1) | 0.3(5) |
| C(4)-C(7)-C(8)-O(1) | -55.8(3) |
| C(4)-C(7)-C(8)-C(9) | -171.1(3) |
| C(4)-C(7)-C(11)-C(10) | 169.4(2) |
| C(4)-C(7)-C(11)-C(13) | -65.1(3) |
| C(5)-C(4)-C(7)-C(8) | -79.0(3) |
| C(5)-C(4)-C(7)-C(11) | 161.9(3) |
| C(6)-C(1)-C(2)-C(3) | -0.1(5) |
| C(7)-C(4)-C(5)-C(6) | 174.0(3) |
| C(7)-C(8)-C(9)-C(10) | 22.7(4) |
| C(7)-C(11)-C(13)-C(14) | -54.6(3) |
| C(7)-C(11)-C(13)-C(18) | 128.1(3) |
| C(8)-C(7)-C(11)-C(10) | 45.0(3) |
| C(8)-C(7)-C(11)-C(13) | 170.6(2) |
| C(8)-C(9)-C(10)-C(11) | 4.9(4) |
| C(8)-C(9)-C(10)-C(12) | 130.4(3) |
| C(9)-C(10)-C(11)-C(7) | -30.9(3) |
| C(9)-C(10)-C(11)-C(13) | -158.1(3) |
| C(10)-C(11)-C(13)-C(14) | 65.0(4) |
| C(10)-C(11)-C(13)-C(18) | -112.3(3) |
| C(11)-C(7)-C(8)-O(1) | 73.4(3) |
| C(11)-C(7)-C(8)-C(9) | -41.9(3) |
| C(11)-C(13)-C(14)-C(15) | -176.4(3) |
| C(11)-C(13)-C(18)-C(17) | 176.2(3) |
| C(12)-C(10)-C(11)-C(7) | -155.5(3) |

| | |
|---|---|
| C(12)-C(10)-C(11)-C(13) | 77.2(3) |
| C(13)-C(14)-C(15)-C(16) | 0.5(4) |
| C(14)-C(13)-C(18)-C(17) | -1.3(4) |
| C(14)-C(15)-C(16)-O(2) | 178.3(2) |
| C(14)-C(15)-C(16)-C(17) | -1.8(4) |
| C(15)-C(16)-C(17)-C(18) | 1.5(4) |
| C(16)-C(17)-C(18)-C(13) | 0.1(4) |
| C(18)-C(13)-C(14)-C(15) | 1.1(4) |
| C(19)-O(2)-C(16)-C(15) | -175.5(2) |
| C(19)-O(2)-C(16)-C(17) | 4.6(4) |
| O(3)-C(27)-C(28)-C(29) | 92.3(3) |
| O(4)-C(35)-C(36)-C(37) | 178.7(3) |
| C(20)-C(21)-C(22)-C(23) | 0.4(4) |
| C(21)-C(20)-C(25)-C(24) | 0.5(5) |
| C(21)-C(22)-C(23)-C(24) | 1.0(4) |
| C(21)-C(22)-C(23)-C(26) | -175.4(3) |
| C(22)-C(23)-C(24)-C(25) | -1.6(4) |
| C(22)-C(23)-C(26)-C(27) | 73.4(3) |
| C(22)-C(23)-C(26)-C(30) | -168.6(2) |
| C(23)-C(24)-C(25)-C(20) | 0.9(5) |
| C(23)-C(26)-C(27)-O(3) | 55.8(3) |
| C(23)-C(26)-C(27)-C(28) | 171.4(2) |
| C(23)-C(26)-C(30)-C(29) | -168.9(2) |
| C(23)-C(26)-C(30)-C(32) | 67.3(3) |
| C(24)-C(23)-C(26)-C(27) | -102.8(3) |
| C(24)-C(23)-C(26)-C(30) | 15.2(4) |
| C(25)-C(20)-C(21)-C(22) | -1.2(5) |
| C(26)-C(23)-C(24)-C(25) | 174.6(3) |
| C(26)-C(27)-C(28)-C(29) | -25.7(3) |
| C(26)-C(30)-C(32)-C(33) | 51.8(3) |
| C(26)-C(30)-C(32)-C(37) | -132.3(3) |
| C(27)-C(26)-C(30)-C(29) | -45.1(3) |
| C(27)-C(26)-C(30)-C(32) | -168.8(2) |
| C(27)-C(28)-C(29)-C(30) | -1.8(3) |
| C(27)-C(28)-C(29)-C(31) | -125.2(3) |
| C(28)-C(29)-C(30)-C(26) | 29.1(3) |

| | |
|---|---|
| C(28)-C(29)-C(30)-C(32) | 156.3(2) |
| C(29)-C(30)-C(32)-C(33) | -66.9(3) |
| C(29)-C(30)-C(32)-C(37) | 109.0(3) |
| C(30)-C(26)-C(27)-O(3) | -71.7(3) |
| C(30)-C(26)-C(27)-C(28) | 43.9(3) |
| C(30)-C(32)-C(33)-C(34) | 174.0(2) |
| C(30)-C(32)-C(37)-C(36) | -173.6(2) |
| C(31)-C(29)-C(30)-C(26) | 154.3(2) |
| C(31)-C(29)-C(30)-C(32) | -78.6(3) |
| C(32)-C(33)-C(34)-C(35) | 0.2(4) |
| C(33)-C(32)-C(37)-C(36) | 2.4(4) |
| C(33)-C(34)-C(35)-O(4) | -178.4(2) |
| C(33)-C(34)-C(35)-C(36) | 1.3(4) |
| C(34)-C(35)-C(36)-C(37) | -0.9(4) |
| C(35)-C(36)-C(37)-C(32) | -1.0(4) |
| C(37)-C(32)-C(33)-C(34) | -2.0(4) |
| C(38)-O(4)-C(35)-C(34) | 169.9(3) |
| C(38)-O(4)-C(35)-C(36) | -9.8(4) |

**Table S7**. Hydrogen bonds for **6** [Å and °].

| D-H...A | d(D-H) | d(H...A) | d(D...A) | <(DHA) |
|---|---|---|---|---|
| O(3)-H(3A)...O(1) | 0.853(19) | 2.00(2) | 2.845(3) | 169(5) |

## 8. NMR Spectra for Characterization

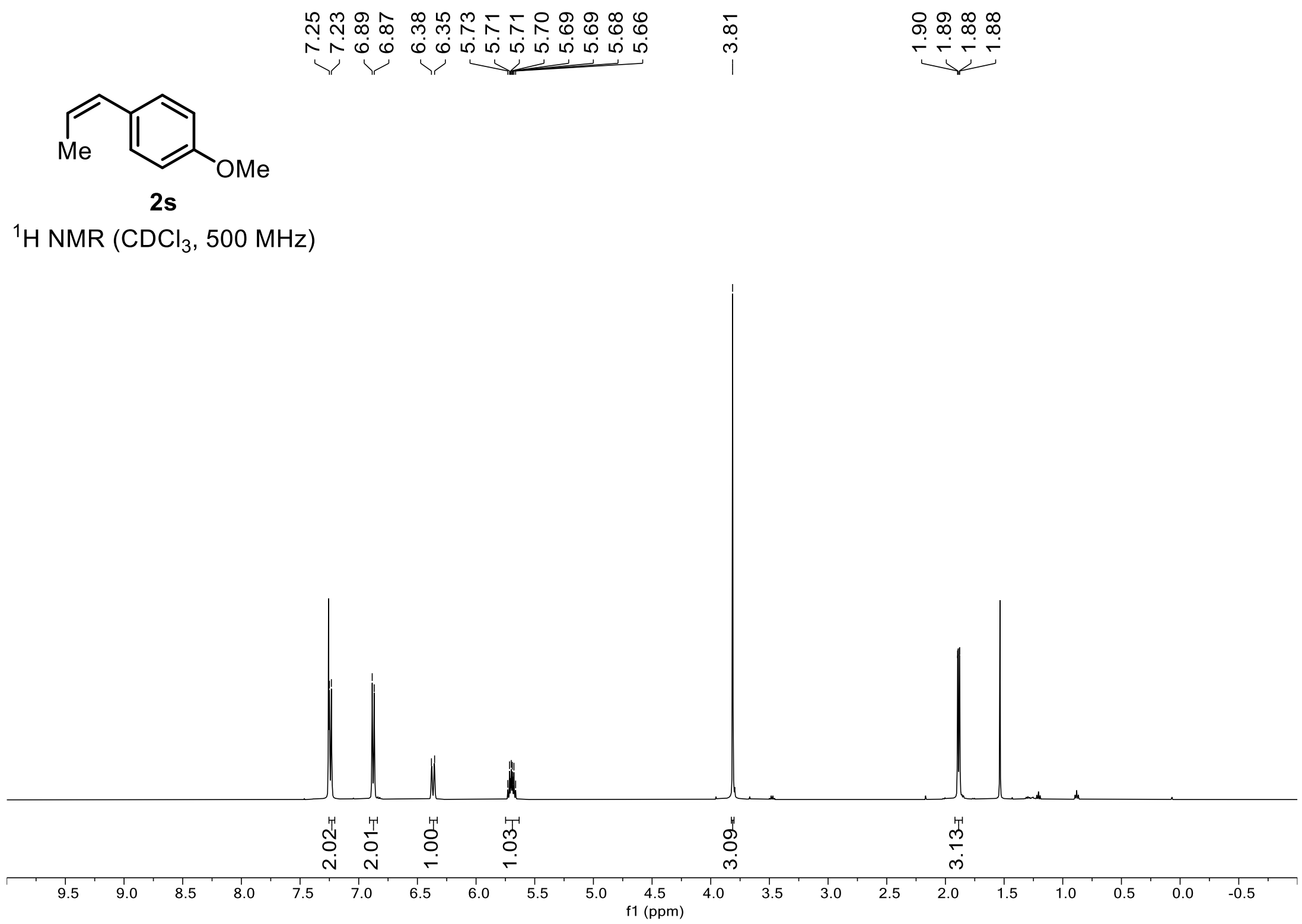


**2s**

$^1$H NMR ($CDCl_3$, 500 MHz)

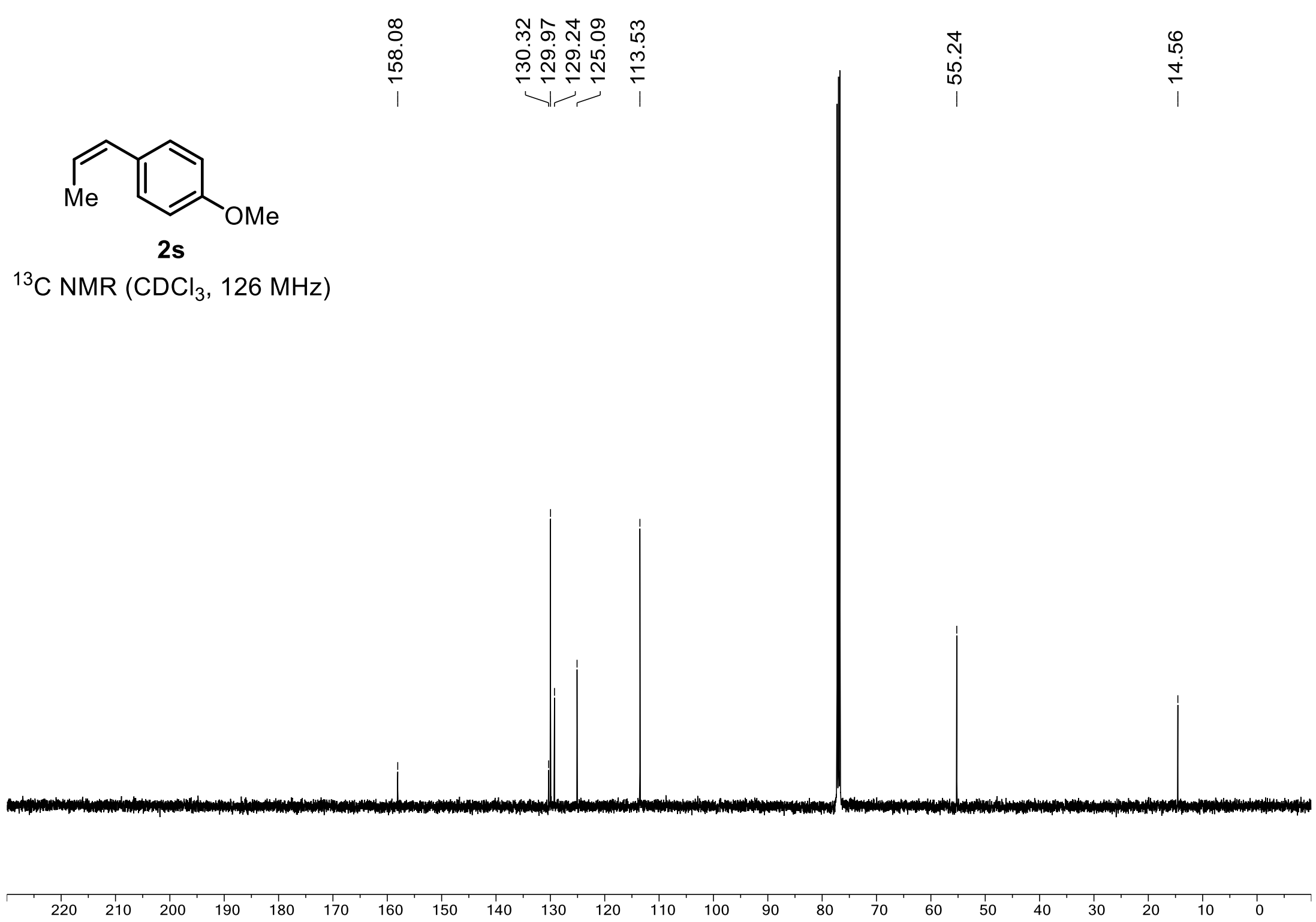


**2s**

$^{13}$C NMR ($CDCl_3$, 126 MHz)

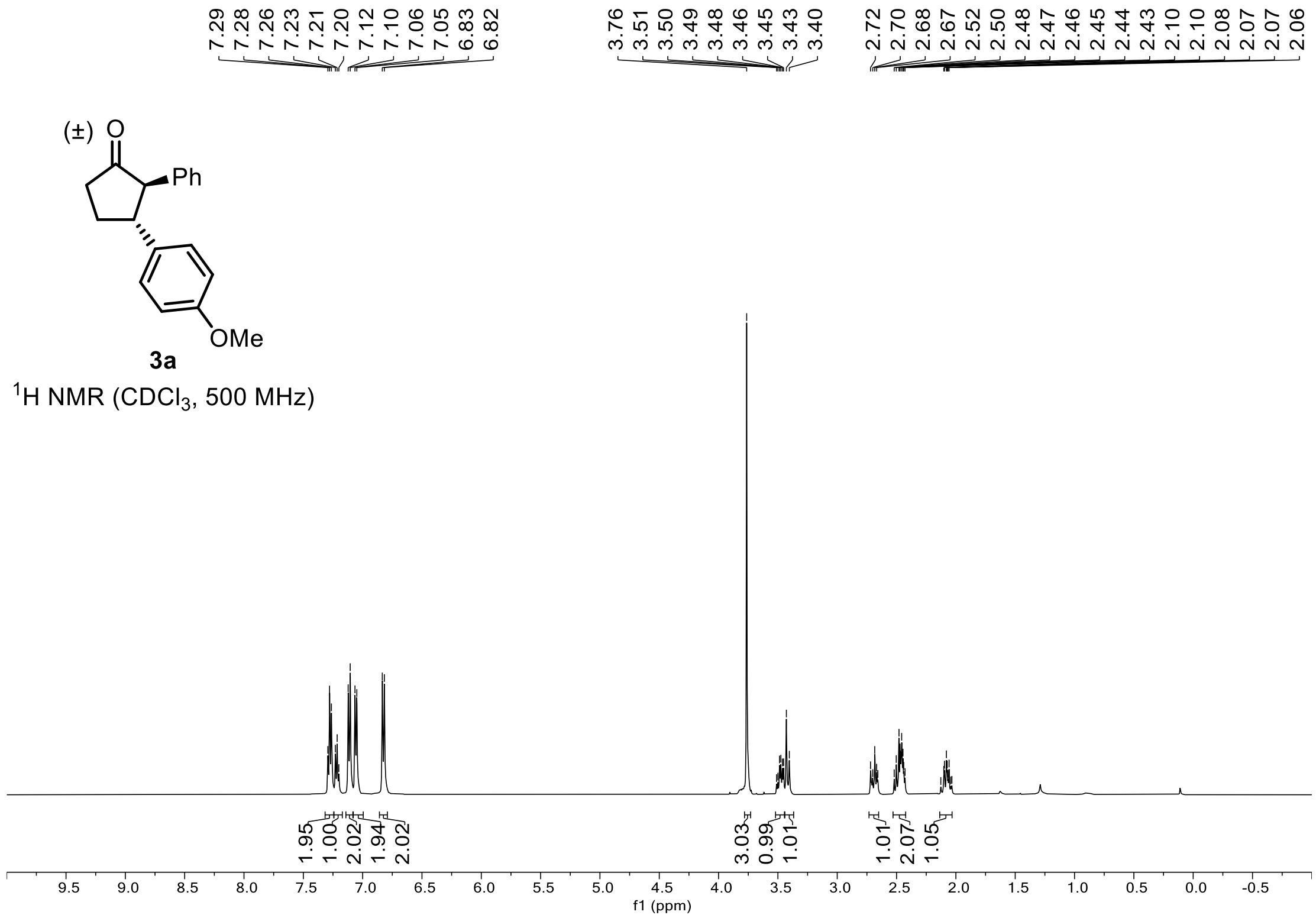
7.29
7.28
7.26
7.23
7.21
7.20
7.12
7.10
7.06
7.05
6.83
6.82
3.76
3.51
3.50
3.49
3.48
3.46
3.45
3.43
3.40
2.72
2.70
2.68
2.67
2.52
2.50
2.48
2.47
2.46
2.45
2.44
2.43
2.10
2.10
2.08
2.07
2.07
2.06
(±)
O
Ph
OMe
3a
$^1$H NMR ($CDCl_3$, 500 MHz)
1.95
1.00
2.02
1.94
2.02
3.03
0.99
1.01
1.01
2.07
1.05
9.5
9.0
8.5
8.0
7.5
7.0
6.5
6.0
5.5
5.0
4.5
4.0
3.5
3.0
2.5
2.0
1.5
1.0
0.5
0.0
-0.5
f1 (ppm)

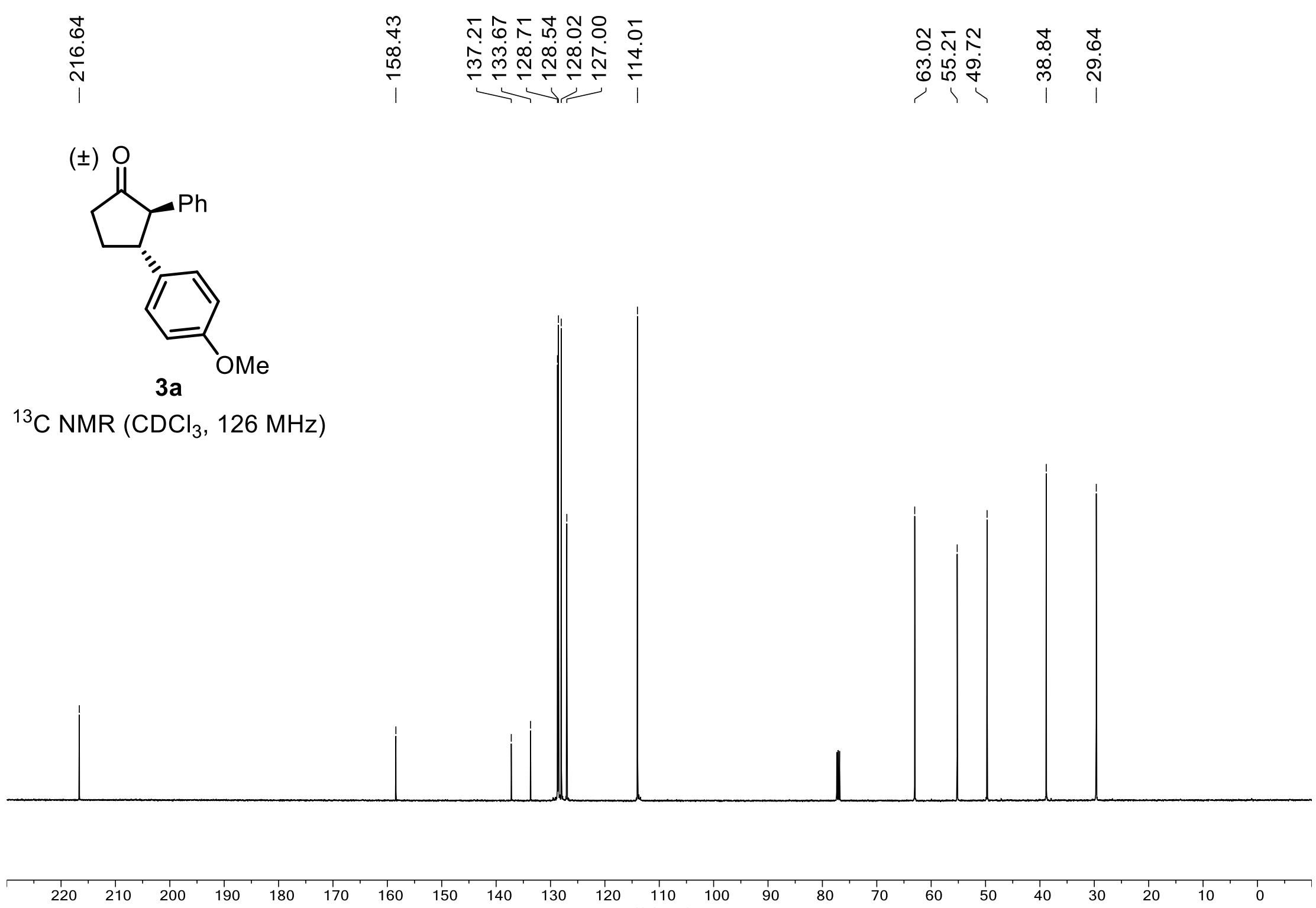
216.64
158.43
137.21
133.67
128.71
128.54
128.02
127.00
114.01
63.02
55.21
49.72
38.84
29.64
(±)
O
Ph
OMe
3a
$^{13}$C NMR ($CDCl_3$, 126 MHz)
220
210
200
190
180
170
160
150
140
130
120
110
100
90
80
70
60
50
40
30
20
10
0
f1 (ppm)

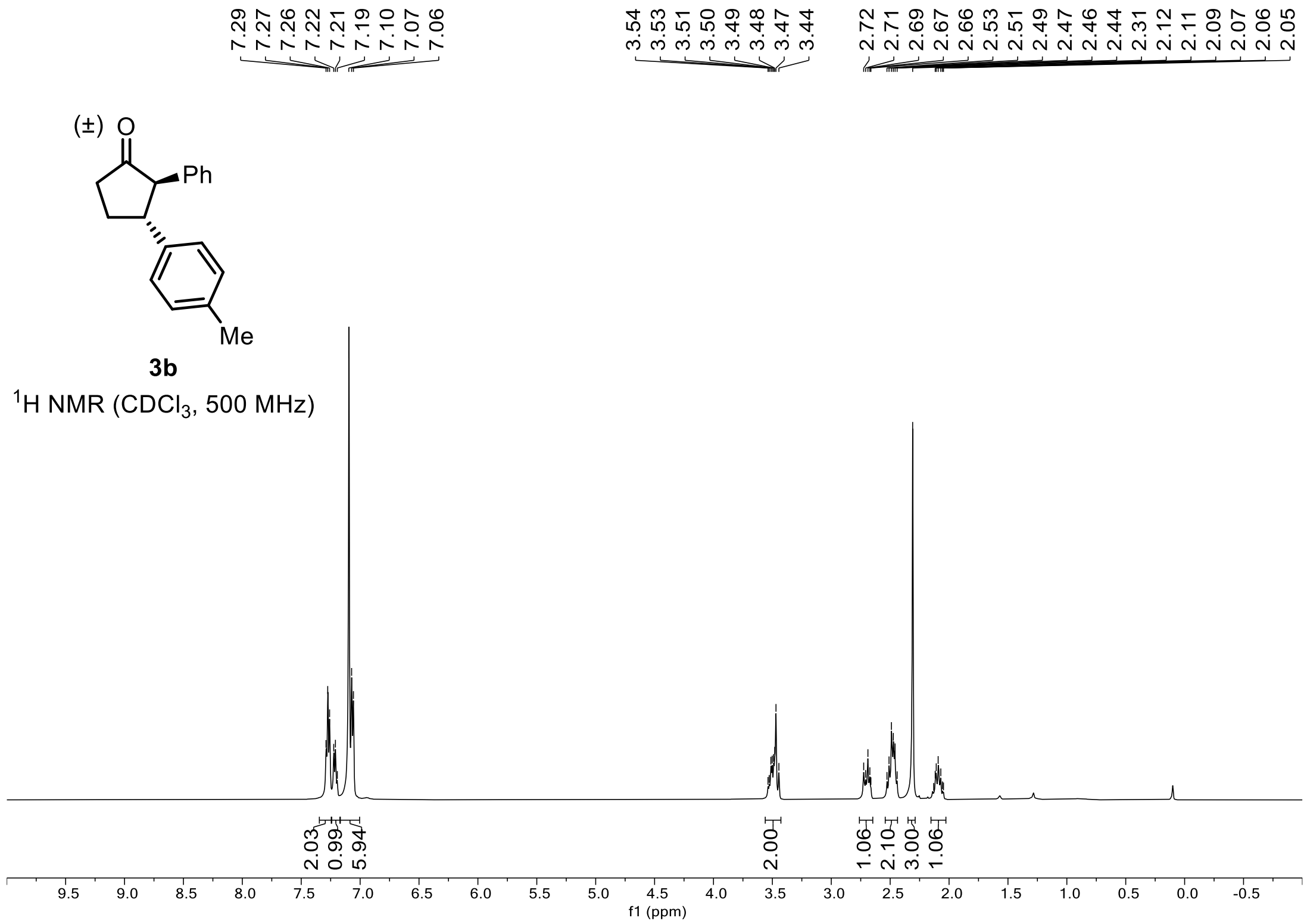


**3b**

$^{1}H$ NMR ($CDCl_3$, 500 MHz)

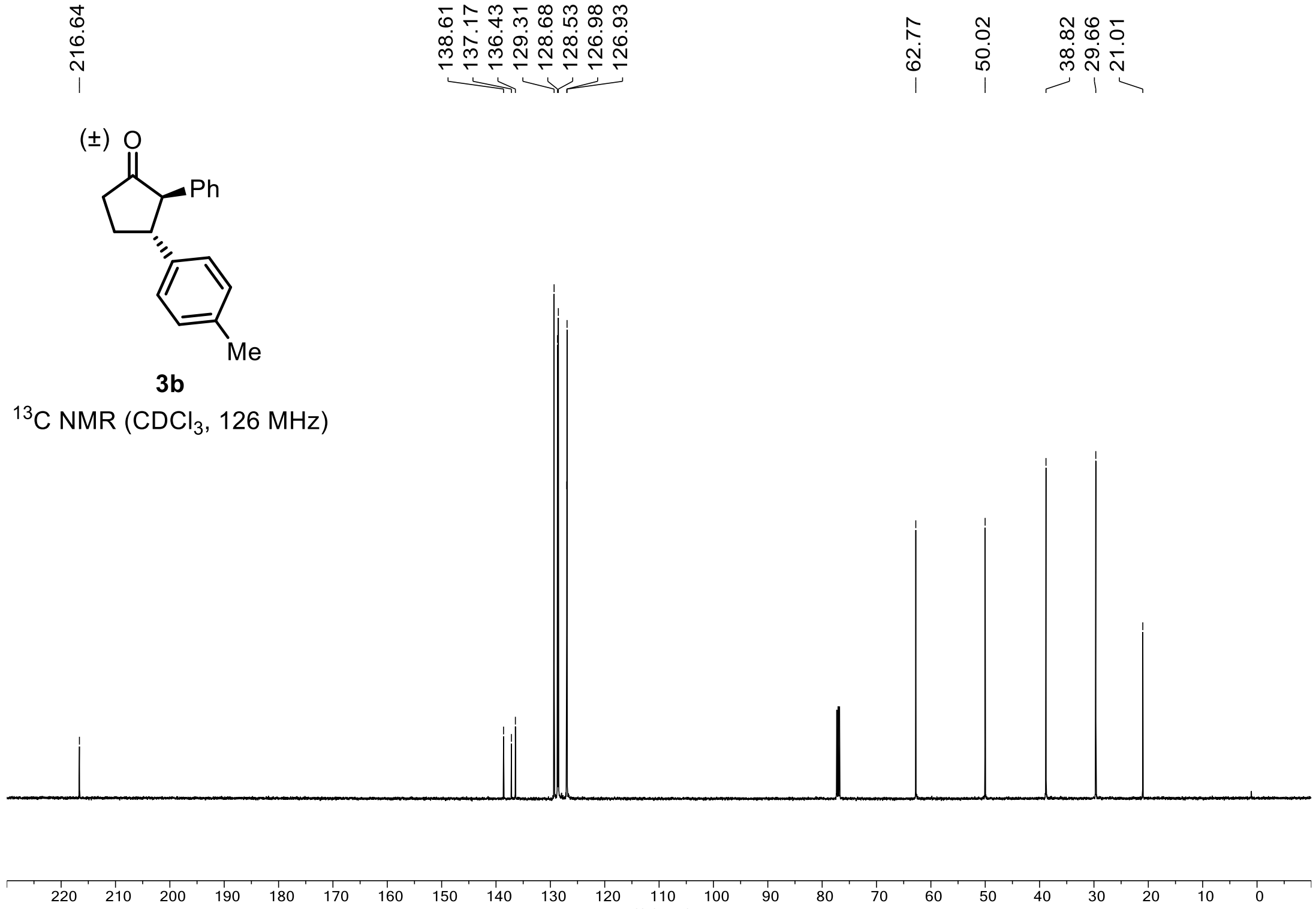


**3b**

$^{13}C$ NMR ($CDCl_3$, 126 MHz)

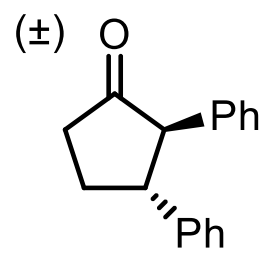

**3c**

$^{1}$H NMR ($CDCl_3$, 500 MHz)

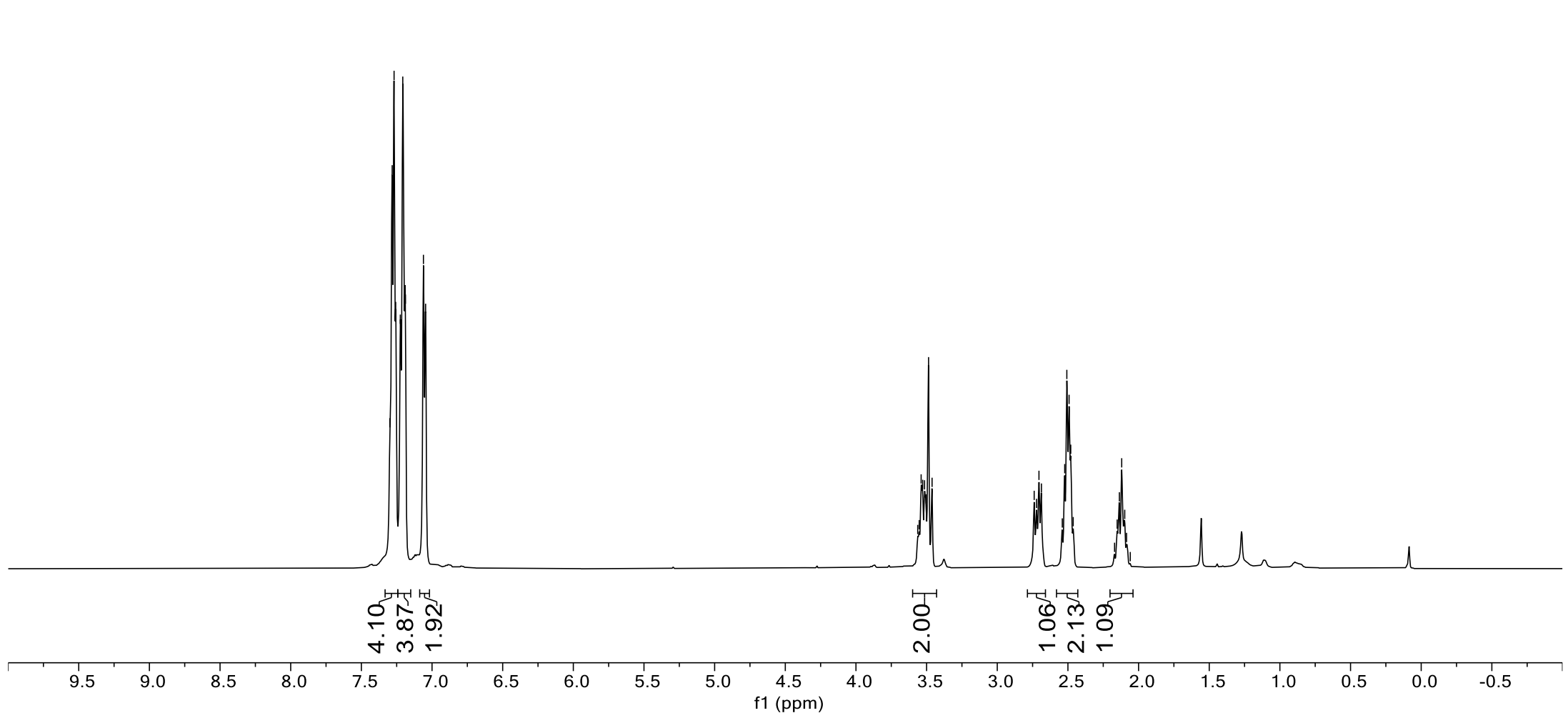


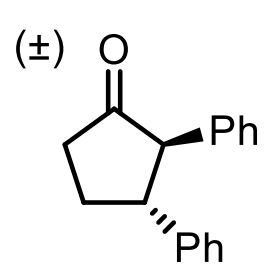

**3c**

$^{13}$C NMR ($CDCl_3$, 126 MHz)

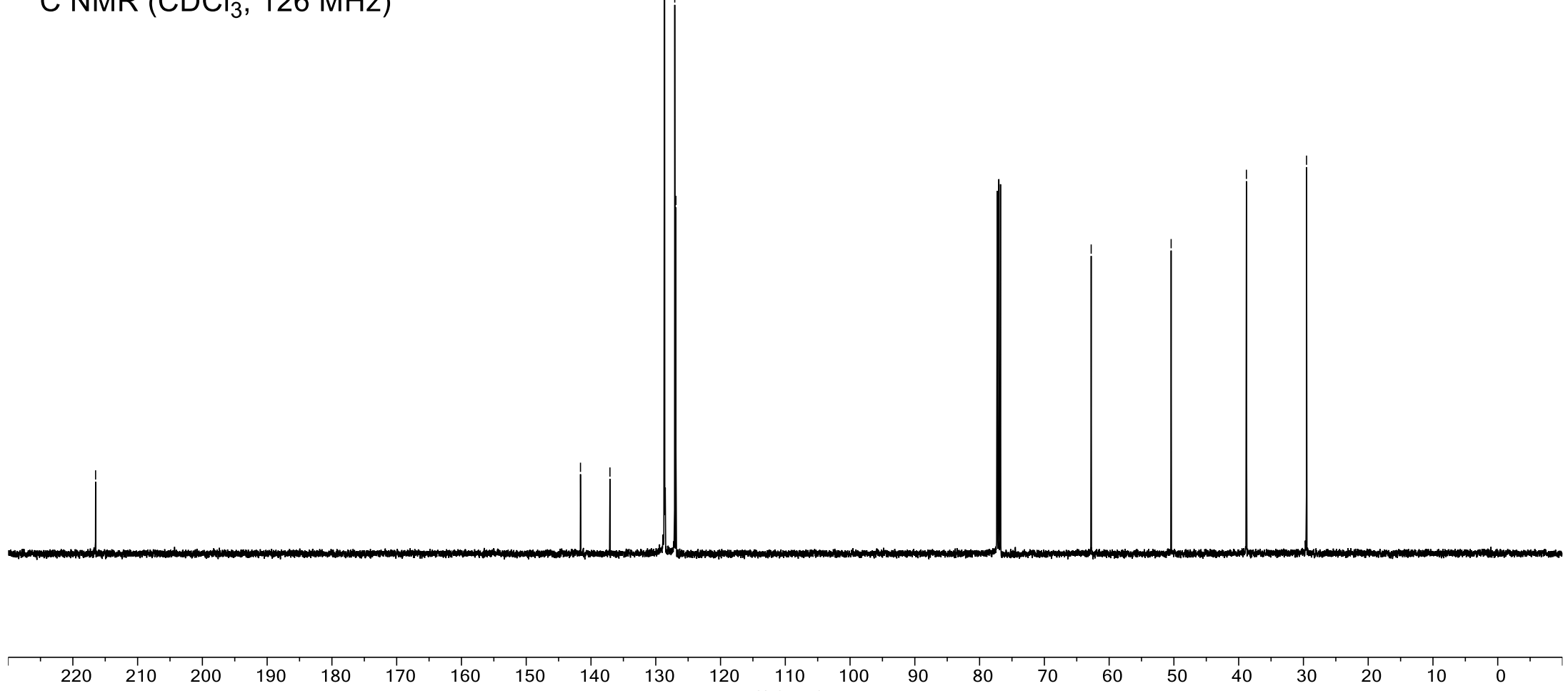

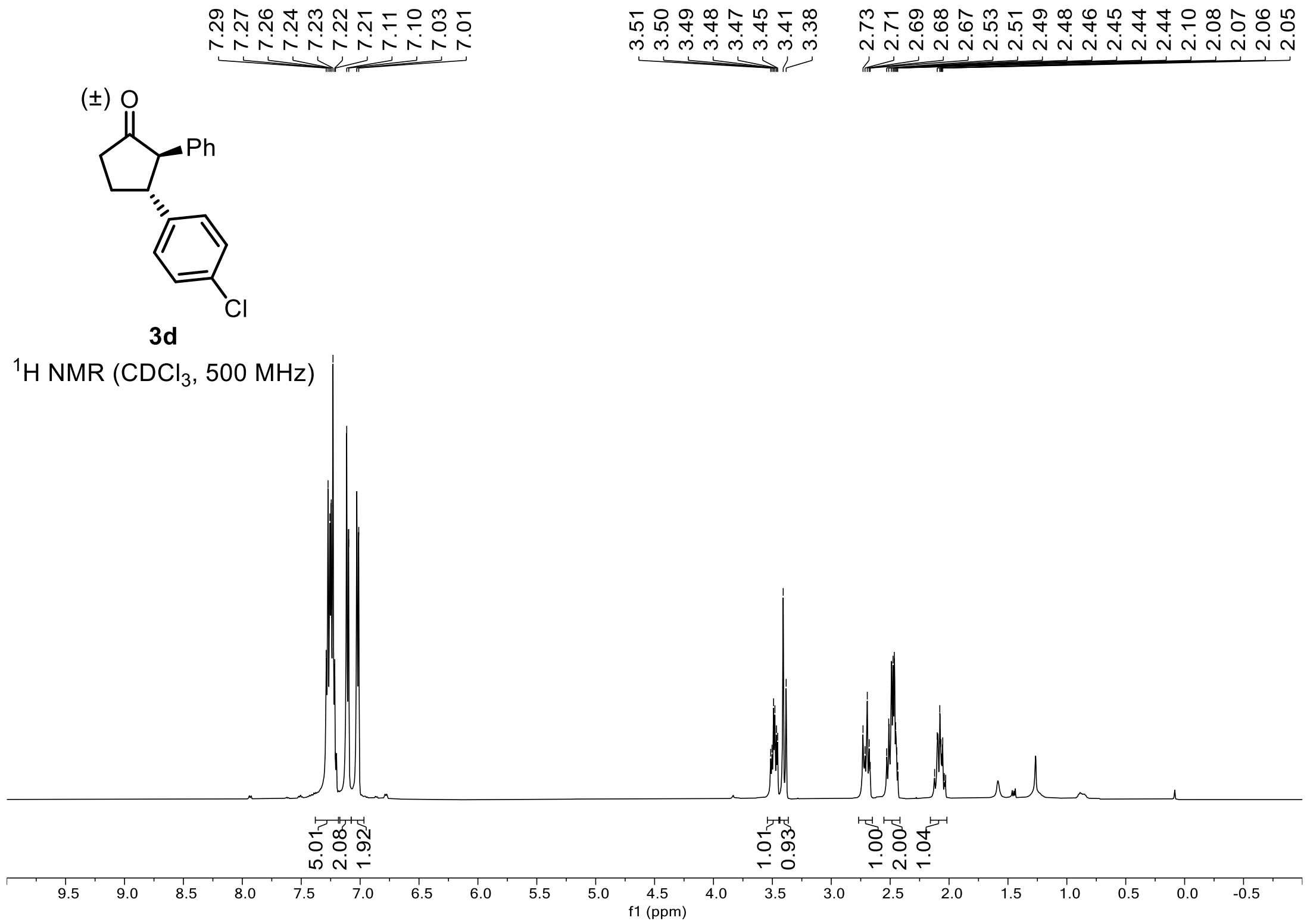


**3d**

$^{1}H$ NMR ($CDCl_3$, 500 MHz)

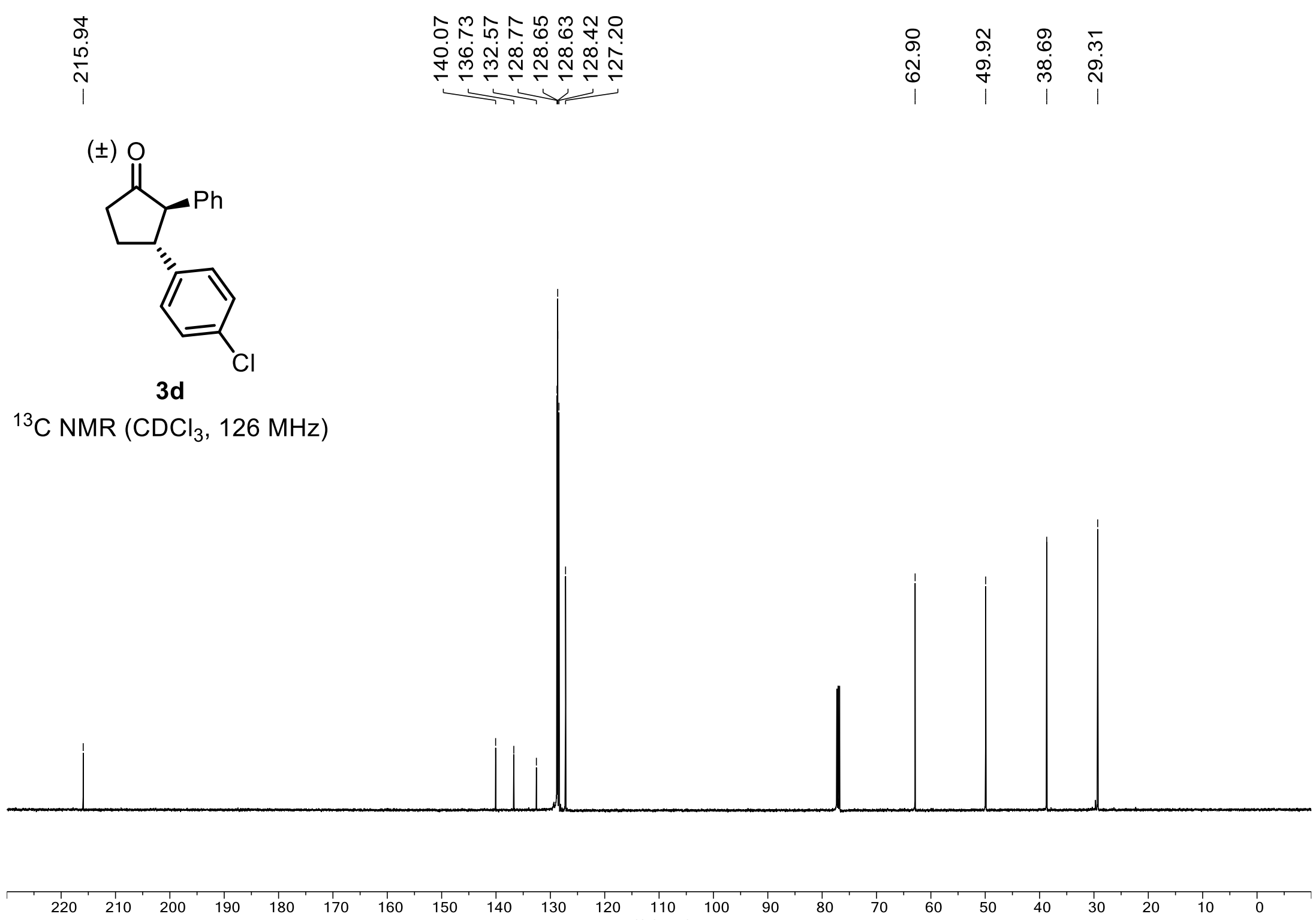


**3d**

$^{13}C$ NMR ($CDCl_3$, 126 MHz)

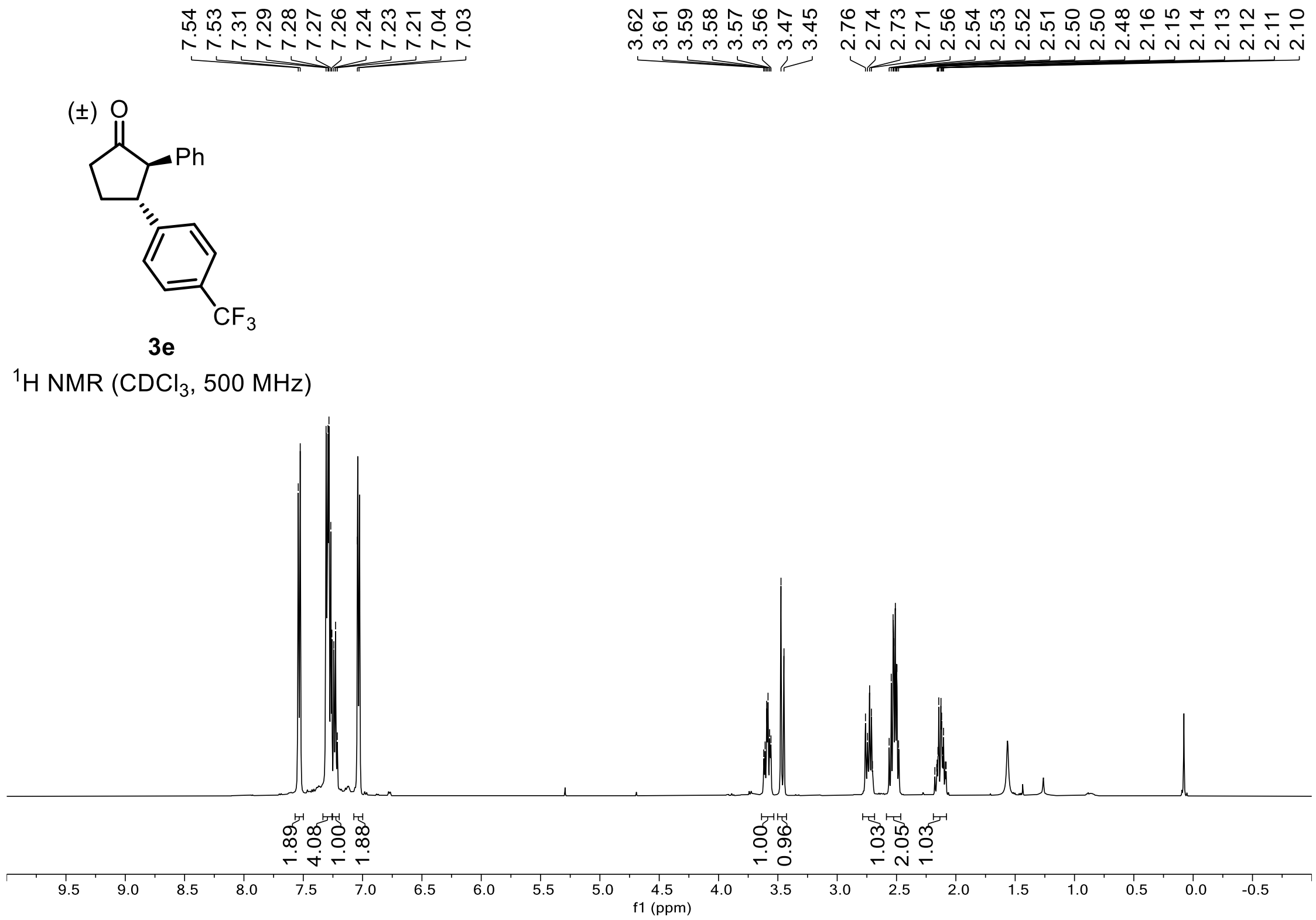
7.54
7.53
7.31
7.29
7.28
7.27
7.26
7.24
7.23
7.21
7.04
7.03
3.62
3.61
3.59
3.58
3.57
3.56
3.47
3.45
2.76
2.74
2.73
2.71
2.56
2.54
2.53
2.52
2.51
2.50
2.50
2.48
2.16
2.15
2.14
2.13
2.12
2.11
2.10
(±)
O
Ph
$CF_3$
3e
$^{1}$H NMR ($CDCl_3$, 500 MHz)
1.89
4.08
1.00
1.88
1.00
0.96
1.03
2.05
1.03
f1 (ppm)

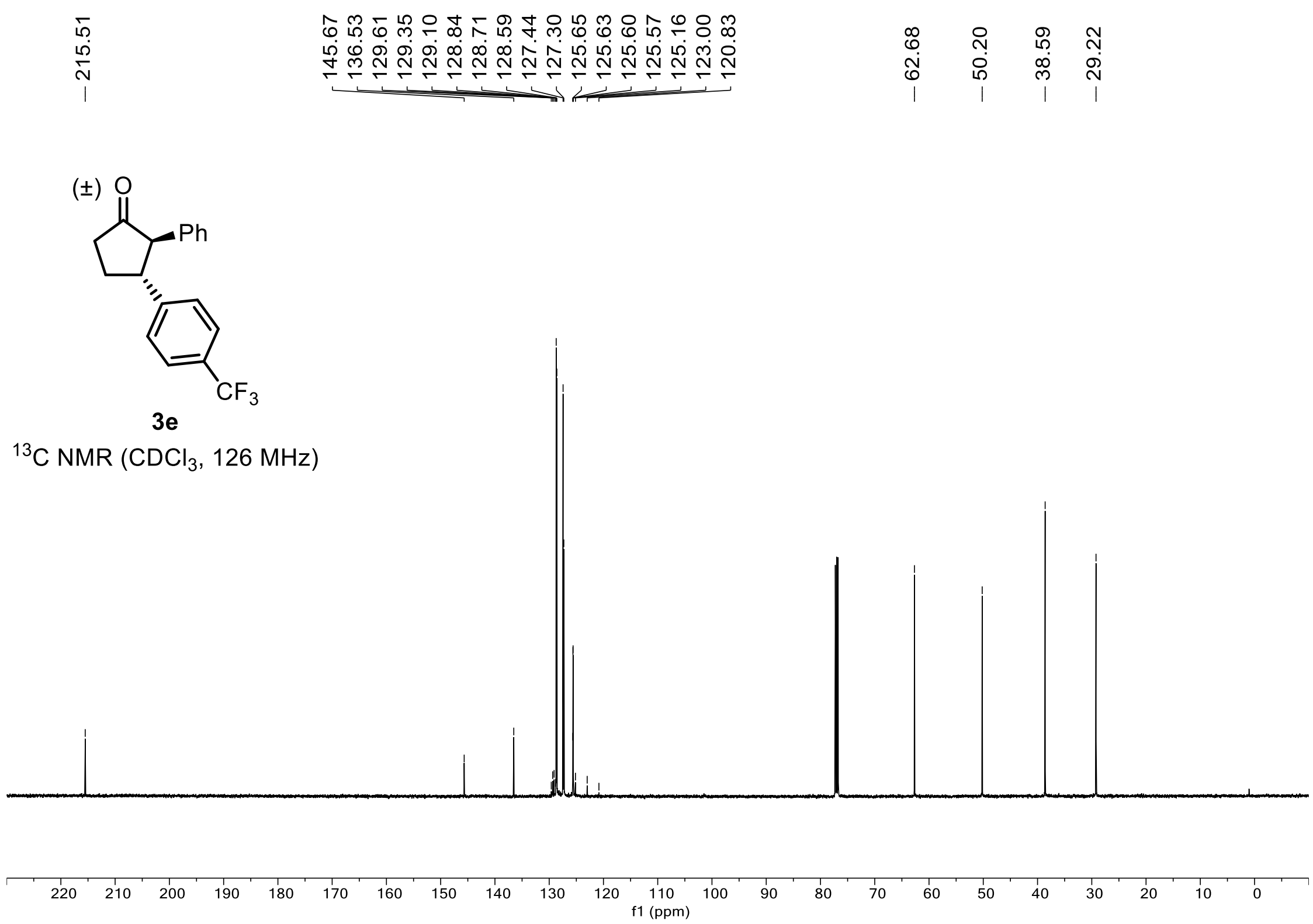
215.51
145.67
136.53
129.61
129.35
129.10
128.84
128.71
128.59
127.44
127.30
125.65
125.63
125.60
125.57
125.16
123.00
120.83
62.68
50.20
38.59
29.22
(±)
O
Ph
$CF_3$
3e
$^{13}$C NMR ($CDCl_3$, 126 MHz)
f1 (ppm)

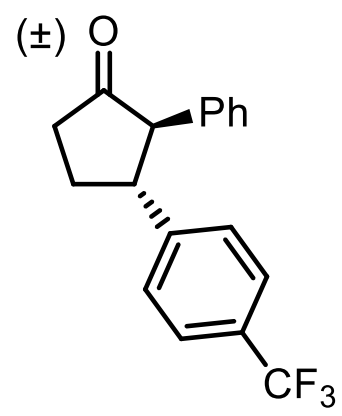

**3e**

$^{19}F$ NMR ($CDCl_3$, 471 MHz)

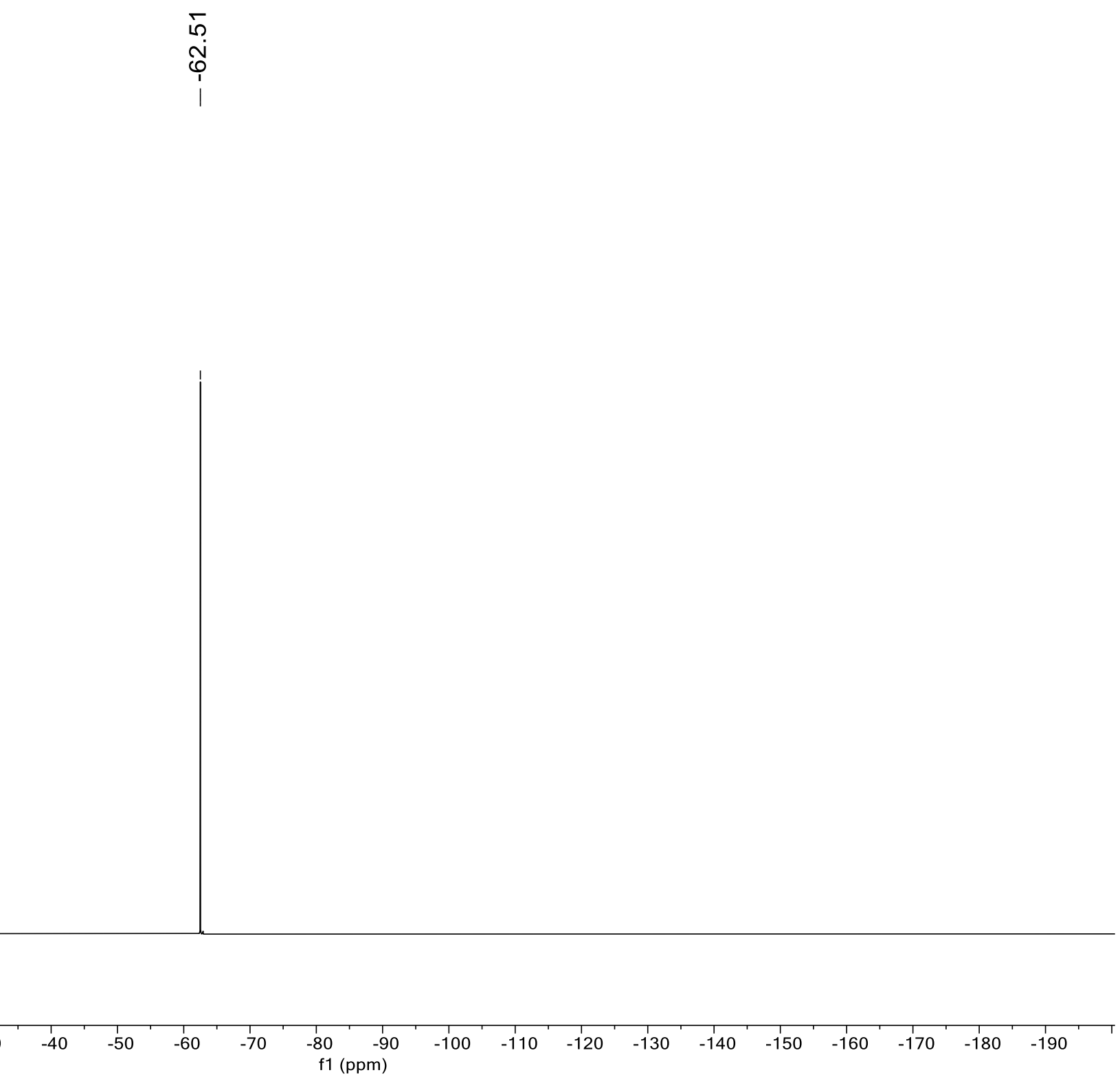

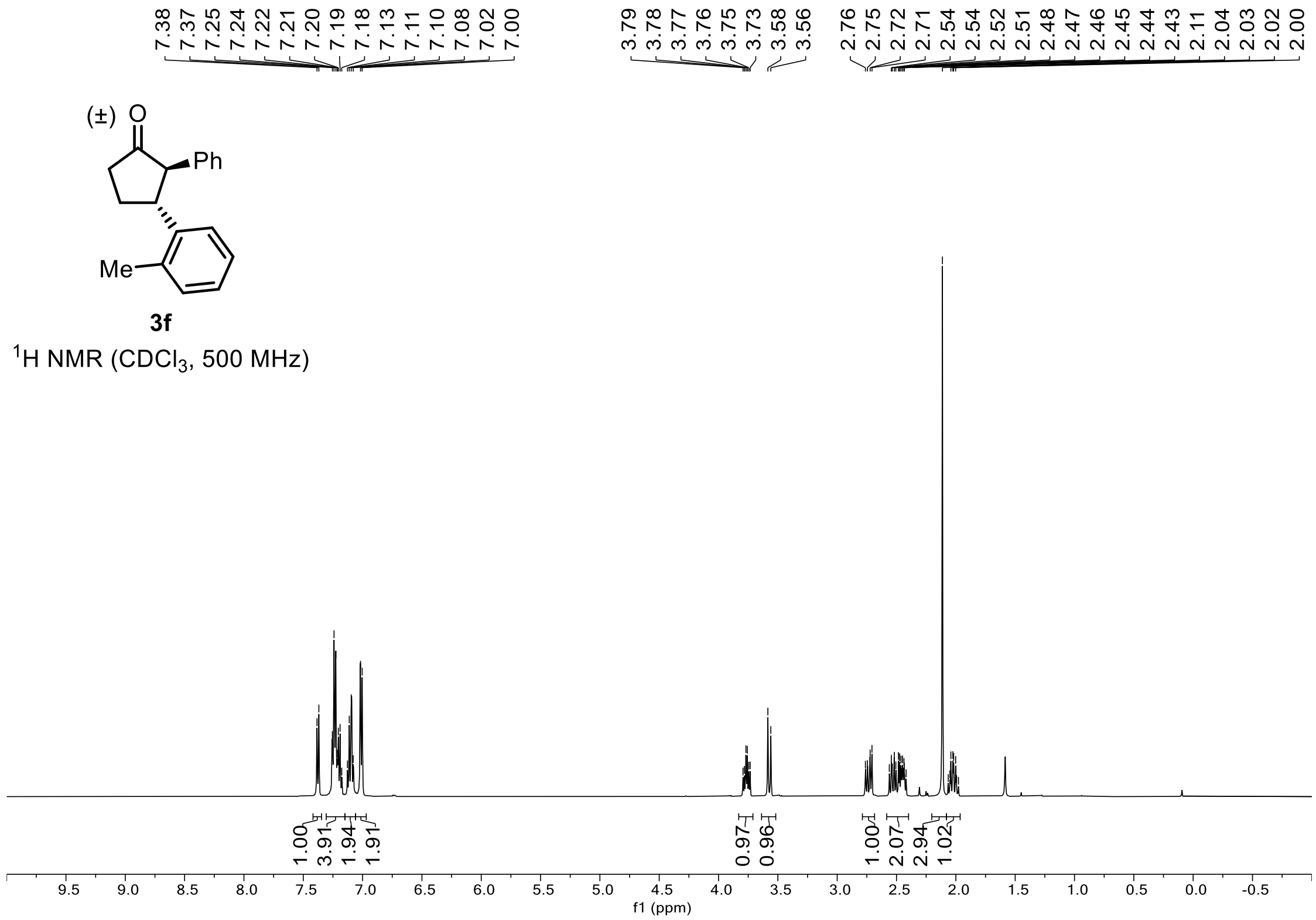
7.38 7.37 7.25 7.24 7.22 7.21 7.20 7.19 7.18 7.13 7.11 7.10 7.08 7.02 7.00
3.79 3.78 3.77 3.76 3.75 3.73 3.58 3.56
2.76 2.75 2.72 2.71 2.54 2.54 2.52 2.51 2.48 2.47 2.46 2.45 2.44 2.43 2.11 2.04 2.03 2.02 2.00
(±)
O
Ph
Me
3f
$^{1}$H NMR (CDCl$_3$, 500 MHz)
1.00 3.91 1.94 1.91
0.97 0.96
1.00 2.07 2.94 1.02
9.5 9.0 8.5 8.0 7.5 7.0 6.5 6.0 5.5 5.0 4.5 4.0 3.5 3.0 2.5 2.0 1.5 1.0 0.5 0.0 -0.5
f1 (ppm)

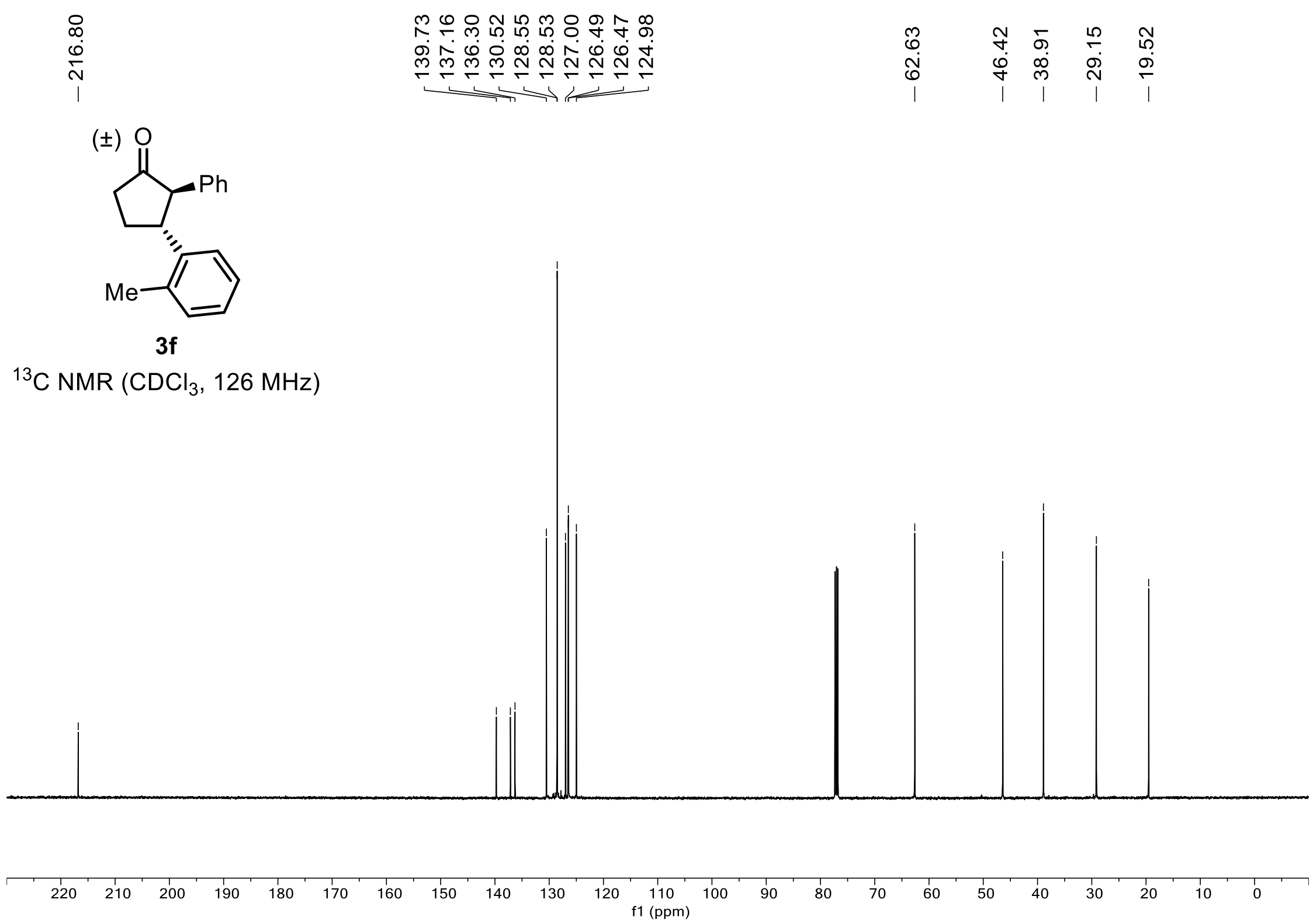
216.80
139.73 137.16 136.30 130.52 128.55 128.53 127.00 126.49 126.47 124.98
62.63
46.42
38.91
29.15
19.52
(±)
O
Ph
Me
3f
$^{13}$C NMR (CDCl$_3$, 126 MHz)
220 210 200 190 180 170 160 150 140 130 120 110 100 90 80 70 60 50 40 30 20 10 0
f1 (ppm)

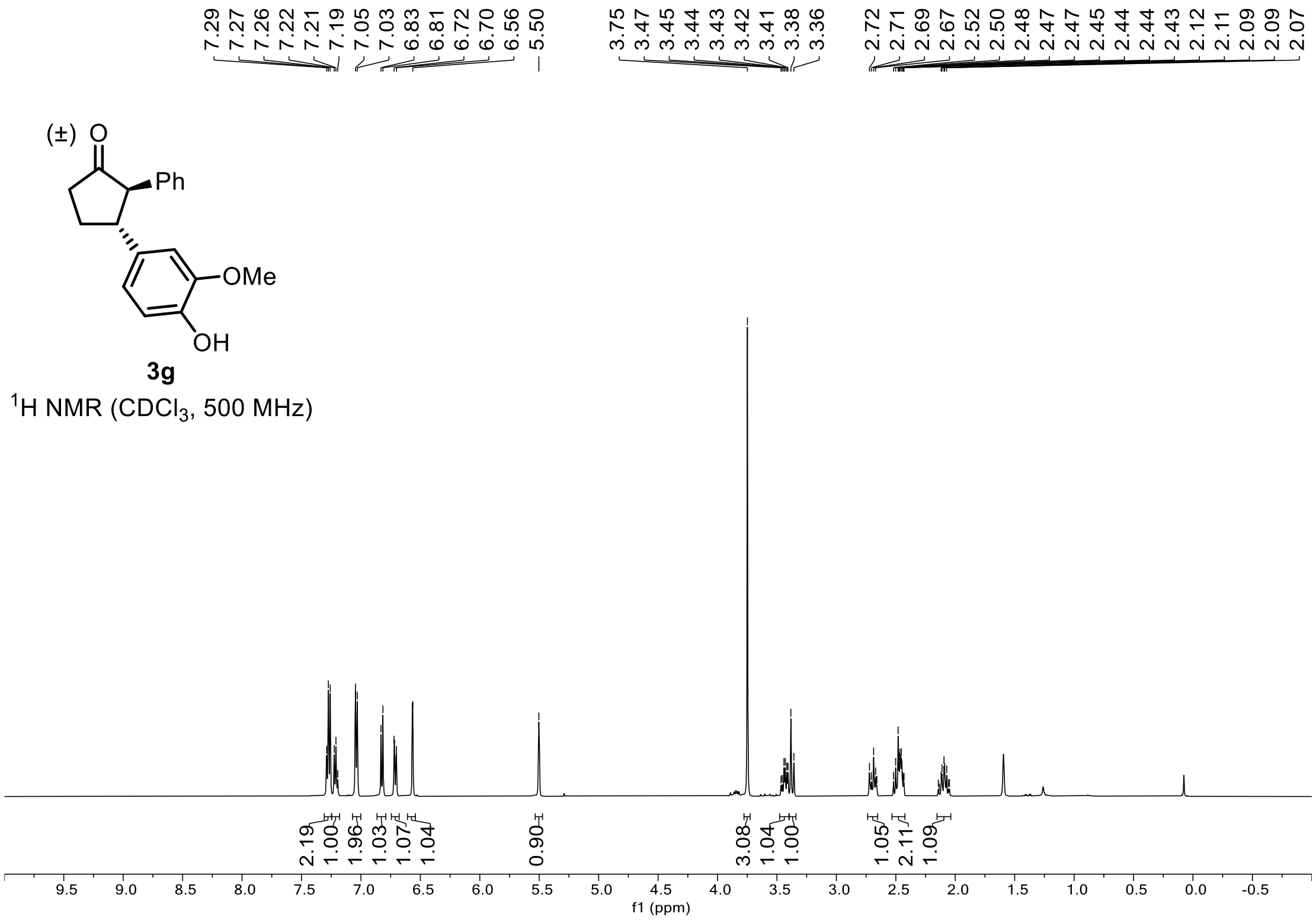
7.29 7.27 7.26 7.22 7.21 7.19 7.05 7.03 6.83 6.81 6.72 6.70 6.56 5.50
3.75 3.47 3.45 3.44 3.43 3.42 3.41 3.38 3.36
2.72 2.71 2.69 2.67 2.52 2.50 2.48 2.47 2.47 2.45 2.44 2.44 2.43 2.12 2.11 2.09 2.07
(±)
O
Ph
OMe
OH
3g
$^{1}$H NMR (CDCl$_3$, 500 MHz)
2.19 1.00 1.96 1.03 1.07 1.04 0.90 3.08 1.04 1.00 1.05 2.11 1.09
f1 (ppm)

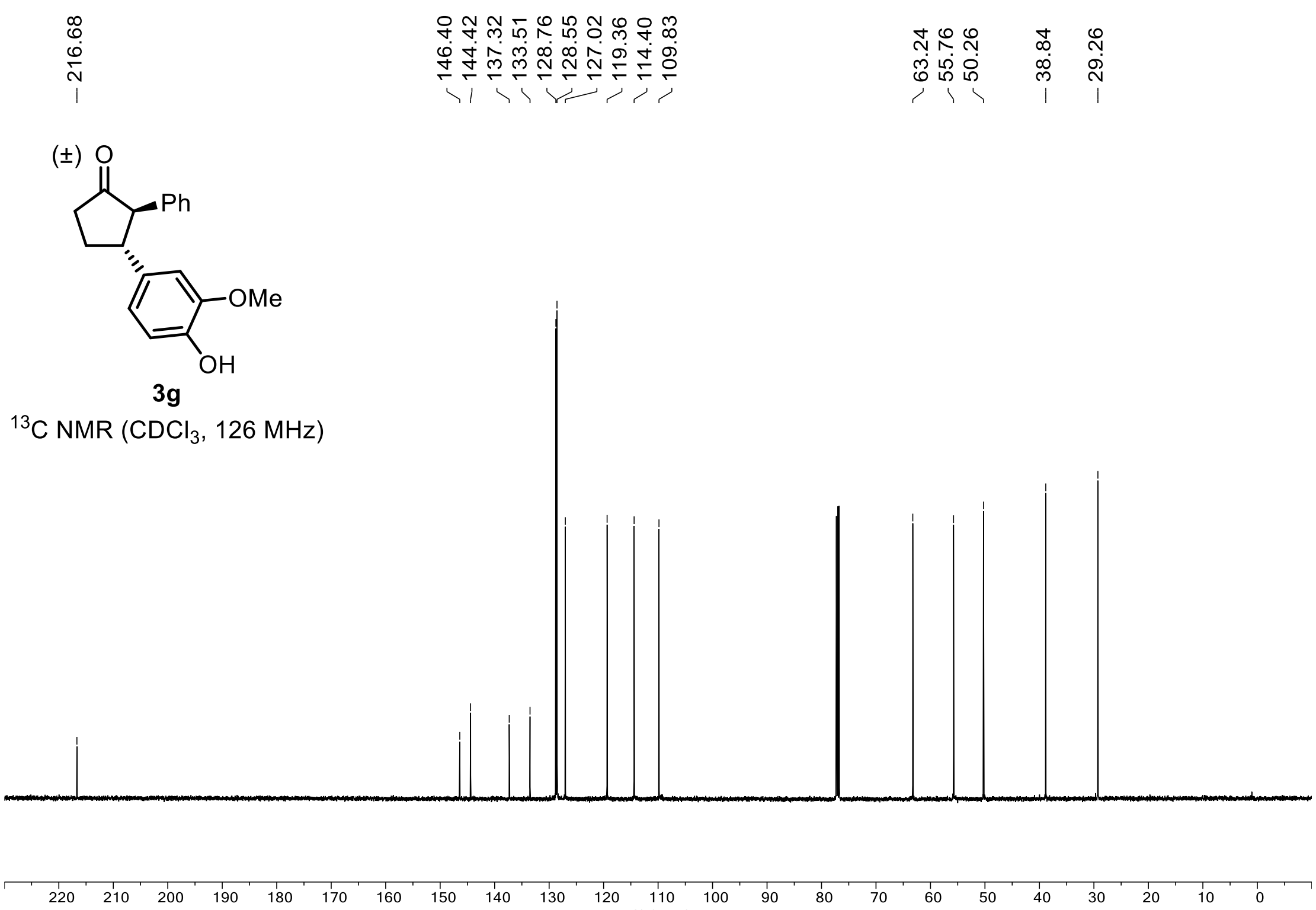
216.68
146.40 144.42 137.32 133.51 128.76 128.55 127.02 119.36 114.40 109.83
63.24 55.76 50.26 38.84 29.26
(±)
O
Ph
OMe
OH
3g
$^{13}$C NMR (CDCl$_3$, 126 MHz)
f1 (ppm)

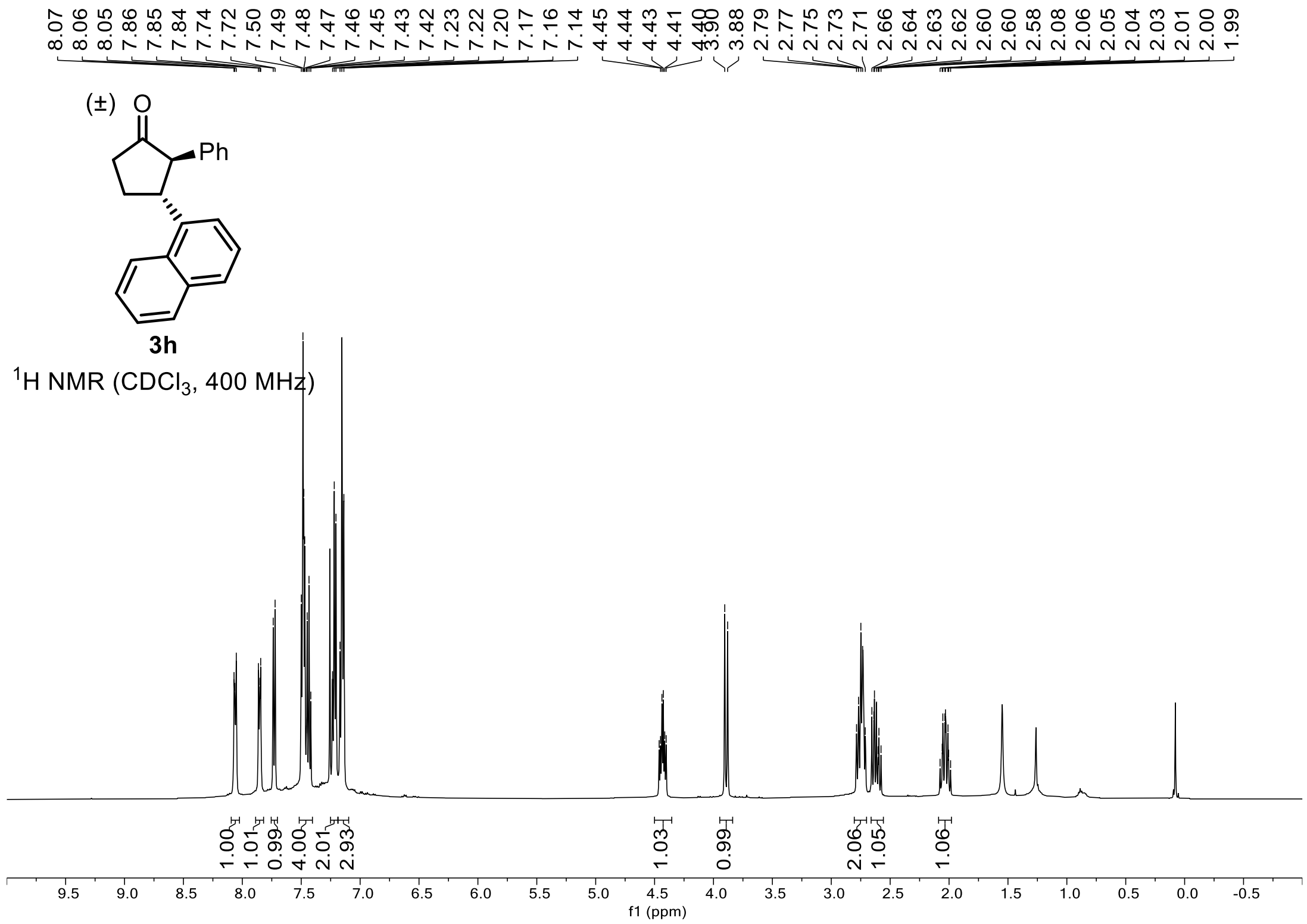
8.07 8.06 8.05 7.86 7.85 7.84 7.74 7.72 7.50 7.49 7.48 7.47 7.46 7.45 7.43 7.42 7.23 7.22 7.20 7.17 7.16 7.14 4.45 4.44 4.43 4.41 4.40 3.90 3.88 2.79 2.77 2.75 2.73 2.71 2.66 2.64 2.63 2.62 2.60 2.60 2.58 2.08 2.06 2.05 2.04 2.03 2.01 2.00 1.99
(±)
O
Ph
3h
$^1$H NMR (CDCl$_3$, 400 MHz)
1.00 1.01 0.99 4.00 2.01 2.93 1.03 0.99 2.06 1.05 1.06
9.5 9.0 8.5 8.0 7.5 7.0 6.5 6.0 5.5 5.0 4.5 4.0 3.5 3.0 2.5 2.0 1.5 1.0 0.5 0.0 -0.5
f1 (ppm)

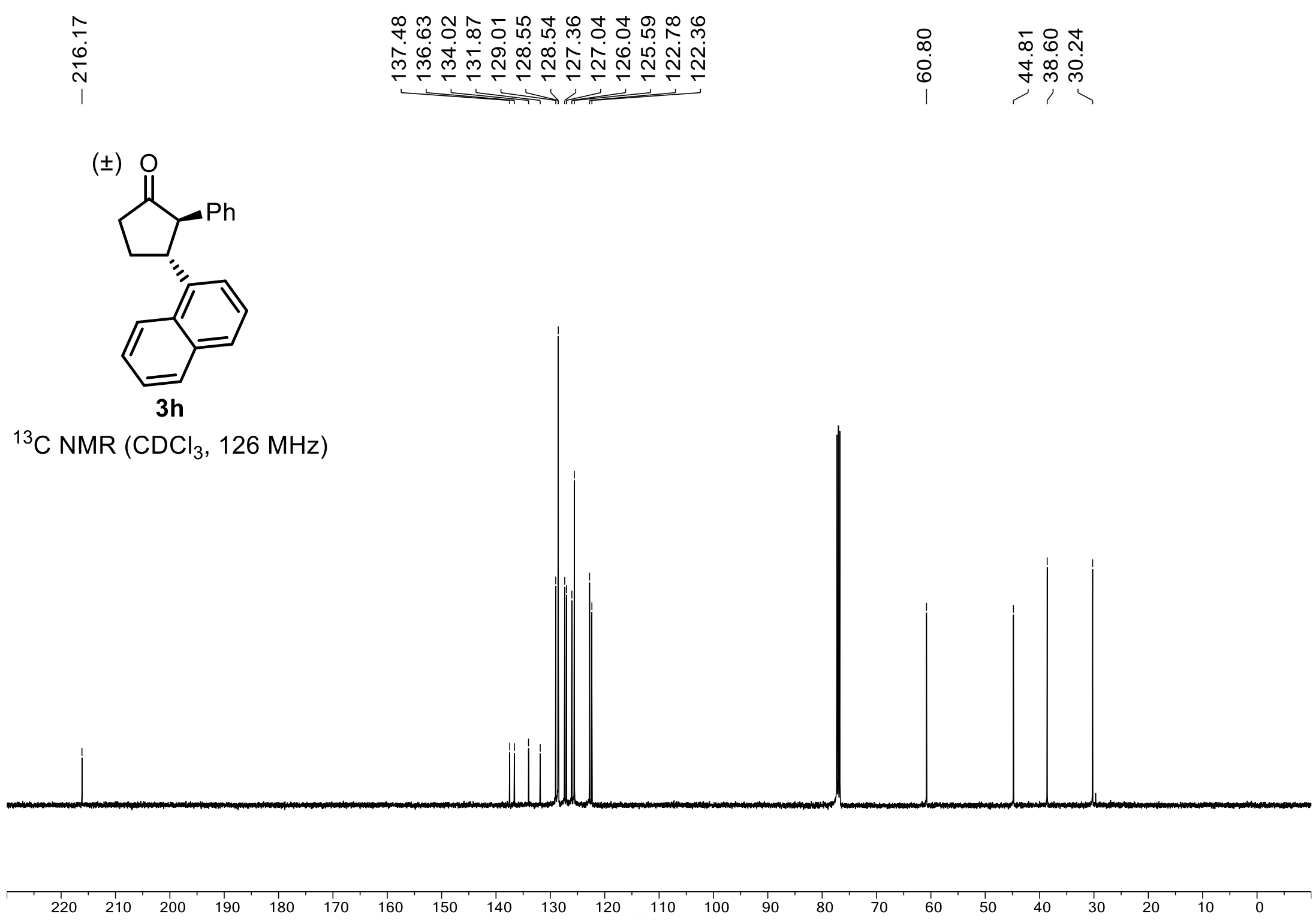
216.17
137.48 136.63 134.02 131.87 129.01 128.55 128.54 127.36 127.04 126.04 125.59 122.78 122.36
60.80
44.81 38.60 30.24
(±)
O
Ph
3h
$^{13}$C NMR (CDCl$_3$, 126 MHz)
220 210 200 190 180 170 160 150 140 130 120 110 100 90 80 70 60 50 40 30 20 10 0
f1 (ppm)

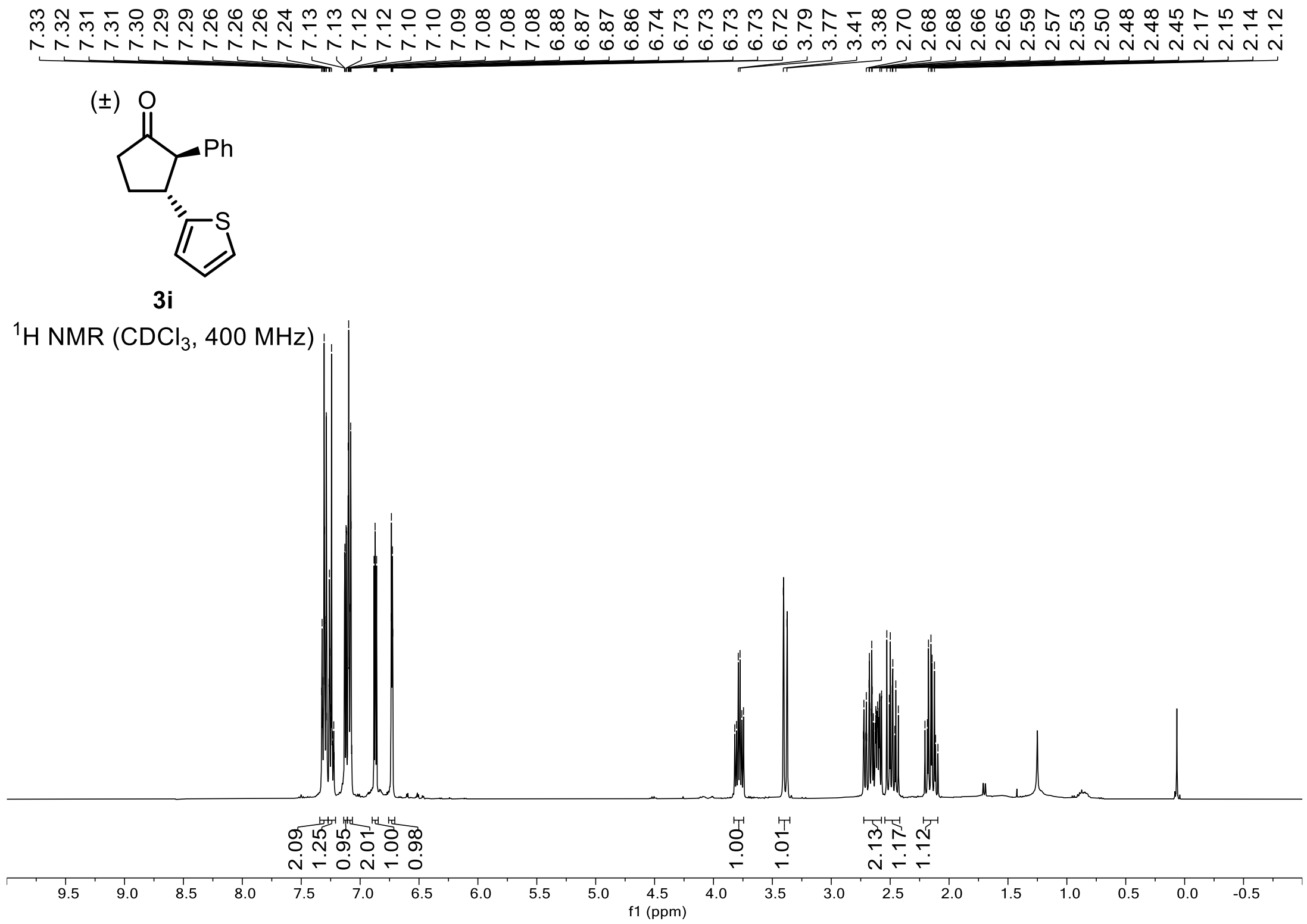
(±)
Ph
S
3i
$^1$H NMR (CDCl$_3$, 400 MHz)
f1 (ppm)

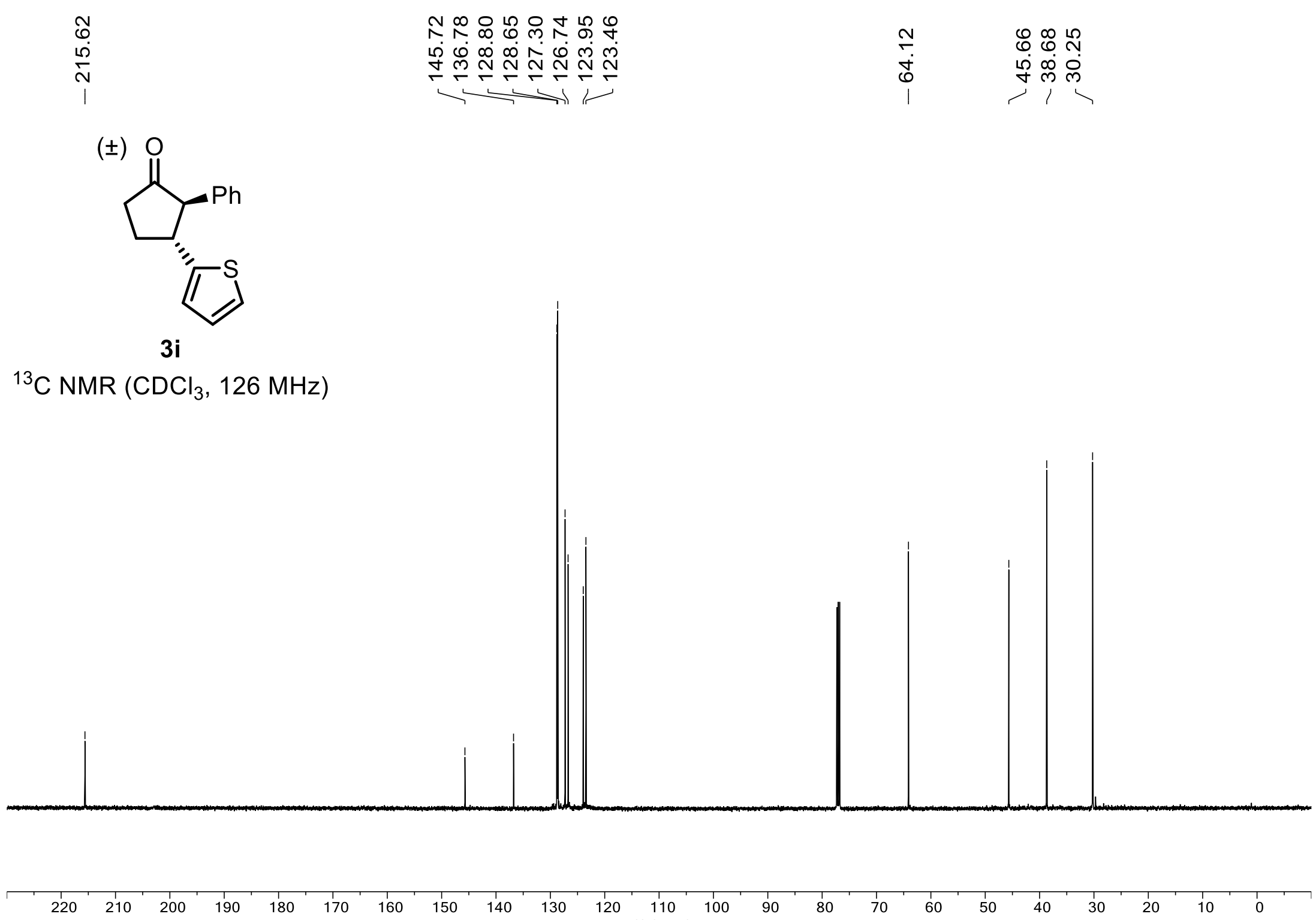
215.62
145.72
136.78
128.80
128.65
127.30
126.74
123.95
123.46
64.12
45.66
38.68
30.25
(±)
Ph
S
3i
$^{13}$C NMR (CDCl$_3$, 126 MHz)
f1 (ppm)

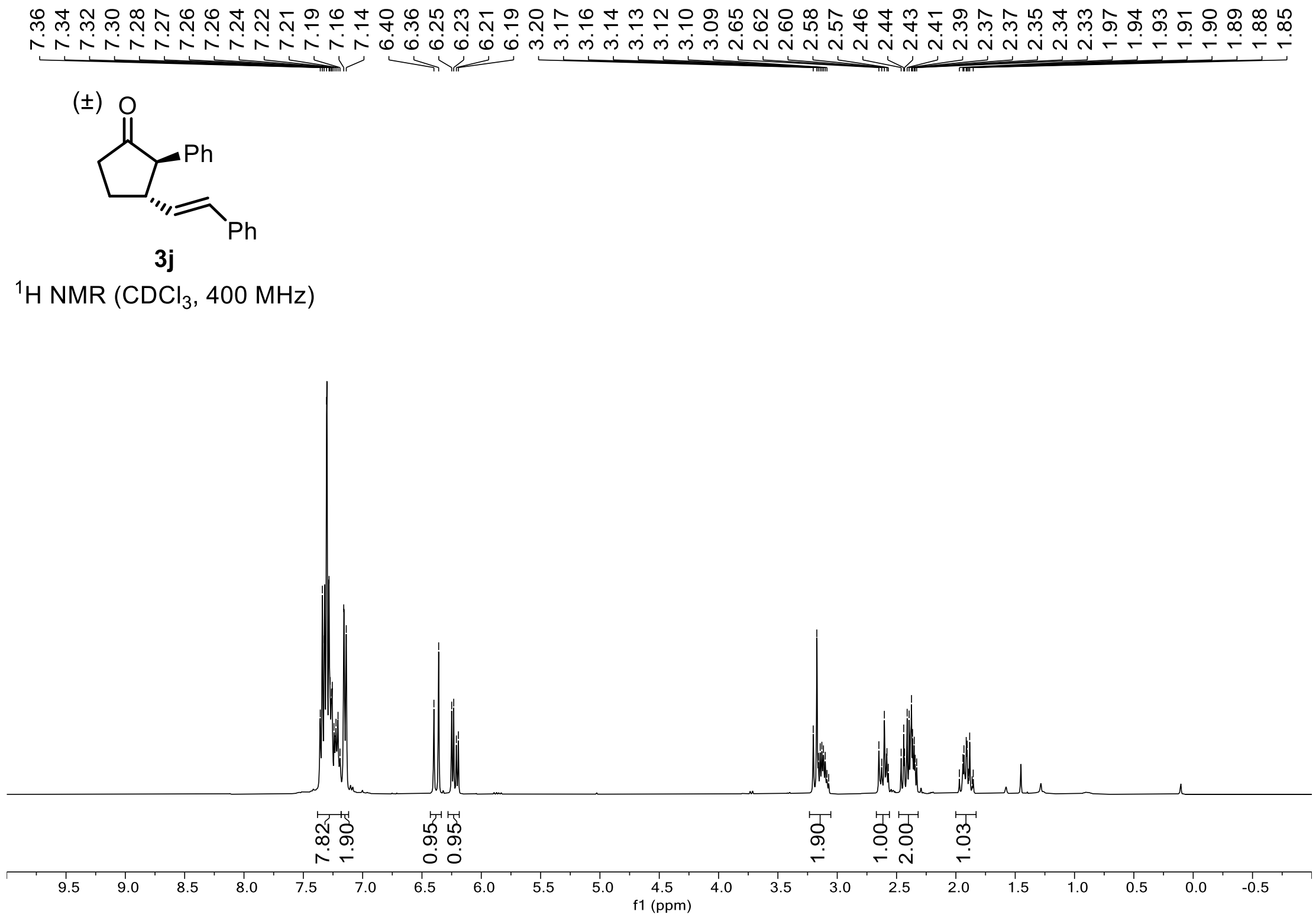
7.36 7.34 7.32 7.30 7.28 7.27 7.26 7.26 7.24 7.22 7.21 7.19 7.16 7.14 6.40 6.36 6.25 6.23 6.21 6.19 3.20 3.17 3.16 3.14 3.13 3.12 3.10 3.09 2.65 2.62 2.60 2.58 2.57 2.46 2.44 2.43 2.41 2.39 2.37 2.37 2.35 2.34 2.33 1.97 1.94 1.93 1.91 1.90 1.89 1.88 1.85
(±)
O
Ph
Ph
3j
$^{1}$H NMR ($CDCl_3$, 400 MHz)
7.82 1.90 0.95 0.95 1.90 1.00 2.00 1.03
9.5 9.0 8.5 8.0 7.5 7.0 6.5 6.0 5.5 5.0 4.5 4.0 3.5 3.0 2.5 2.0 1.5 1.0 0.5 0.0 -0.5
f1 (ppm)

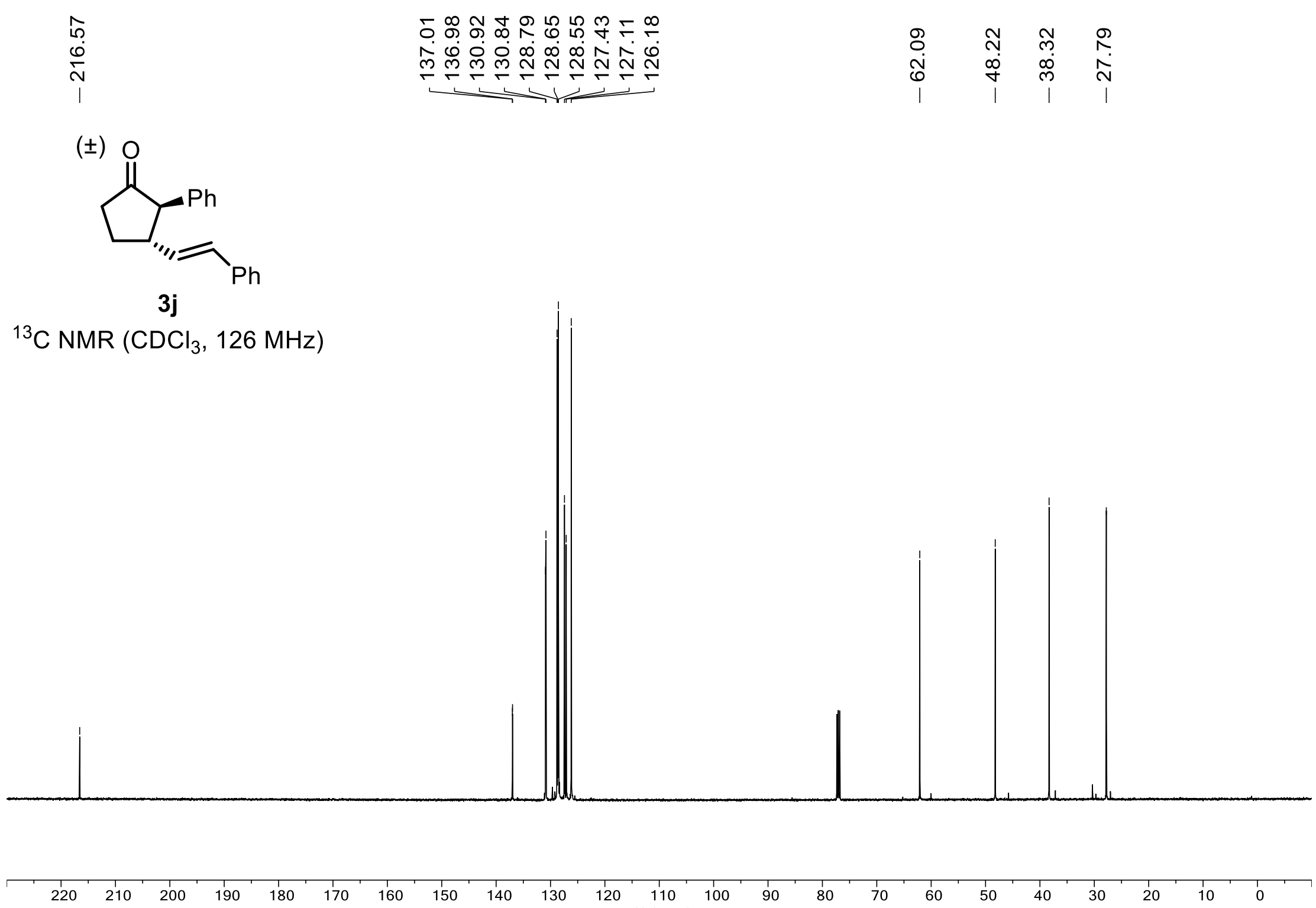
216.57
137.01 136.98 130.92 130.84 128.79 128.65 128.55 127.43 127.11 126.18
62.09
48.22
38.32
27.79
(±)
O
Ph
Ph
3j
$^{13}$C NMR ($CDCl_3$, 126 MHz)
220 210 200 190 180 170 160 150 140 130 120 110 100 90 80 70 60 50 40 30 20 10 0
f1 (ppm)

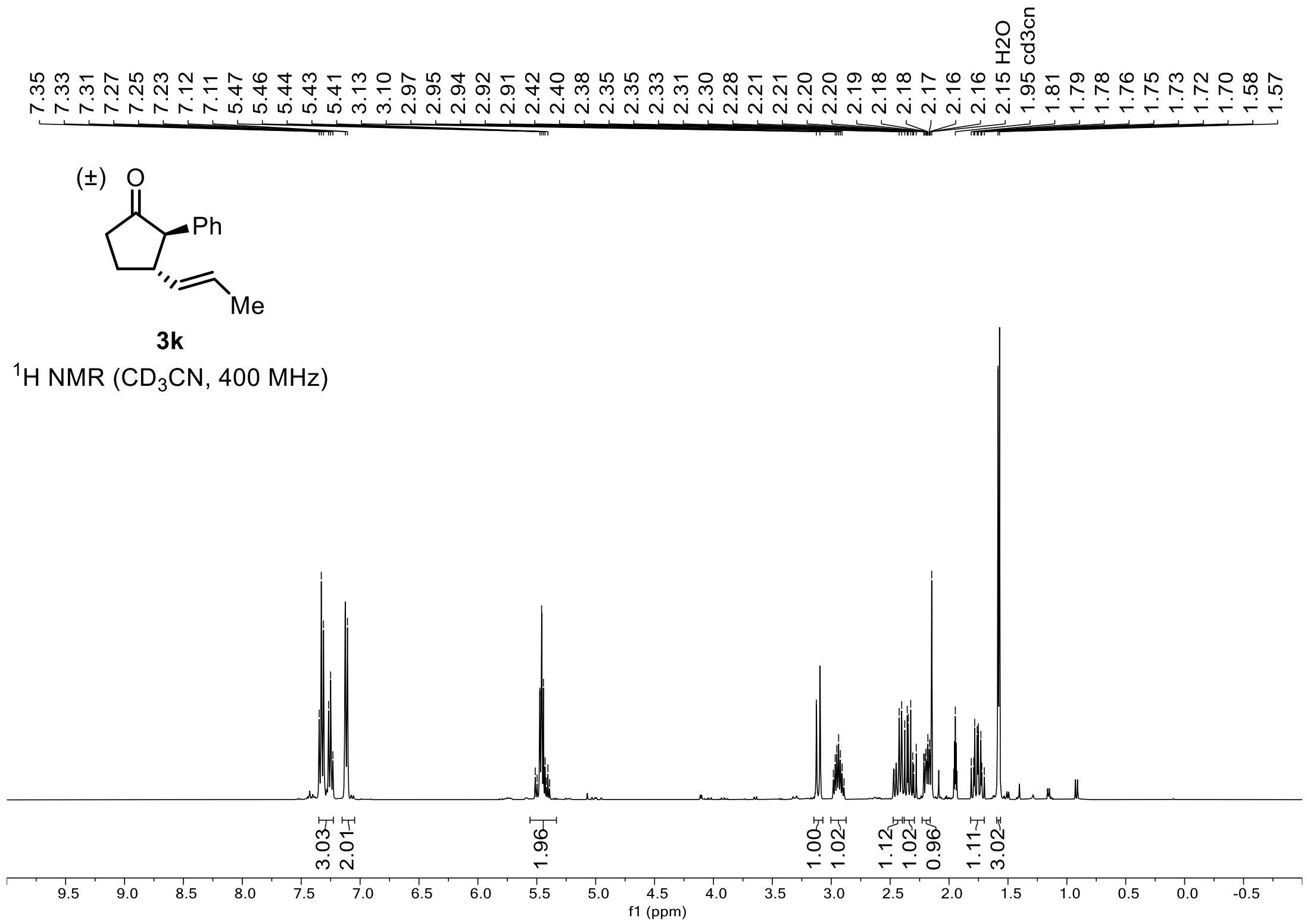
(±)
O
Ph
Me
3k
$^1$H NMR ($CD_3CN$, 400 MHz)
7.35 7.33 7.31 7.27 7.25 7.23 7.12 7.11 5.47 5.46 5.44 5.43 5.41 3.13 3.10 2.97 2.95 2.94 2.92 2.91 2.42 2.40 2.38 2.35 2.35 2.33 2.31 2.30 2.28 2.21 2.21 2.20 2.20 2.19 2.18 2.18 2.17 2.16 2.16 2.15 H2O 1.95 cd3cn 1.81 1.79 1.78 1.76 1.75 1.73 1.72 1.70 1.58 1.57
3.03 2.01 1.96 1.00 1.02 1.12 1.02 0.96 1.11 3.02
f1 (ppm)

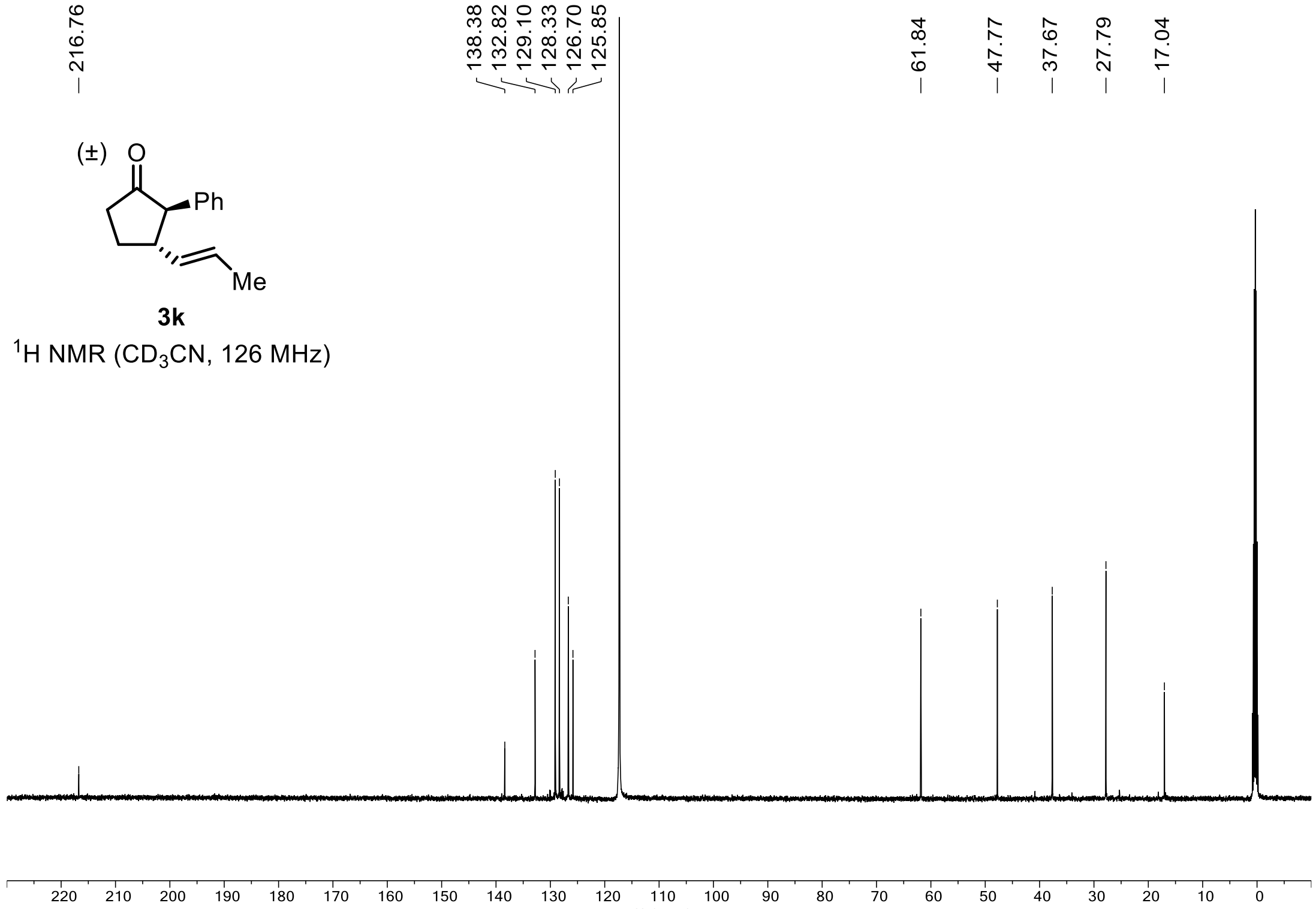
216.76
138.38 132.82 129.10 128.33 126.70 125.85
61.84 47.77 37.67 27.79 17.04
(±)
O
Ph
Me
3k
$^1$H NMR ($CD_3CN$, 126 MHz)
f1 (ppm)

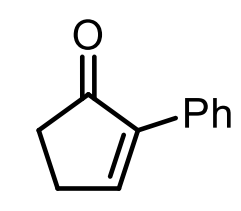

**3l**

$^{1}H$ NMR ($CDCl_3$, 500 MHz)

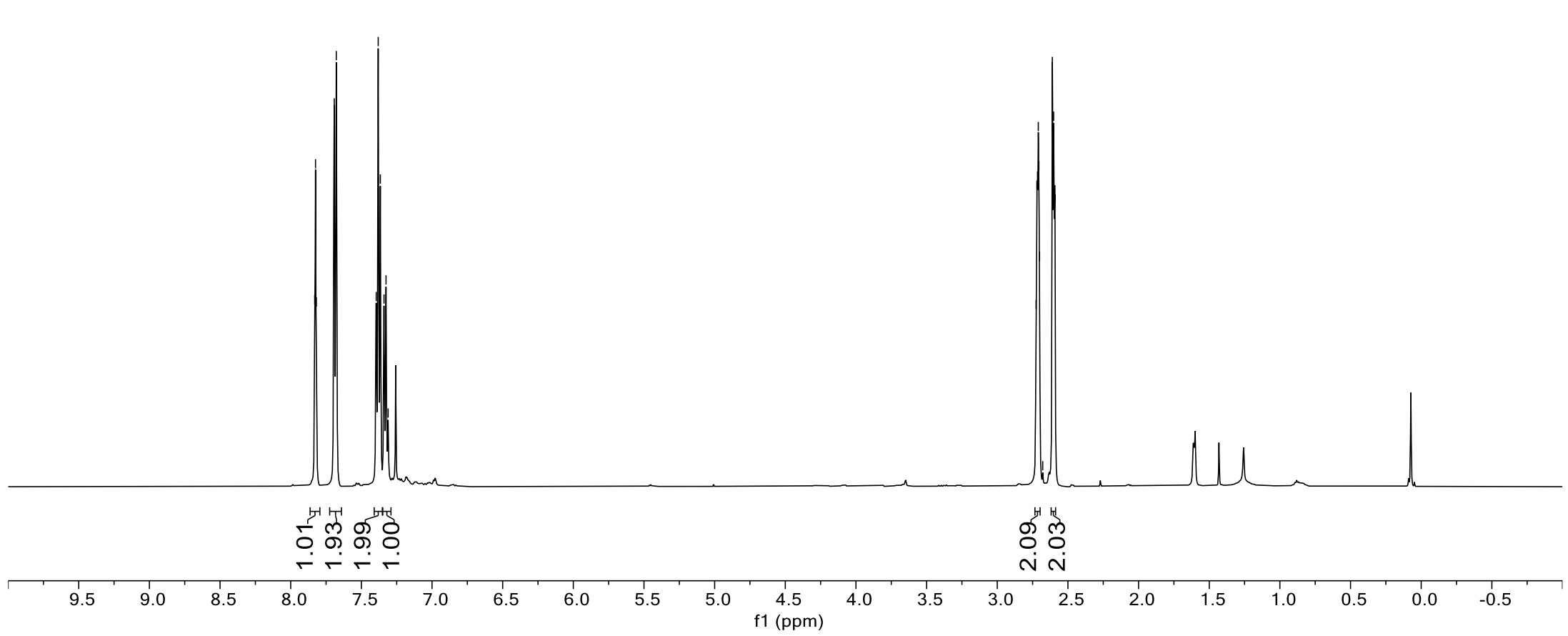


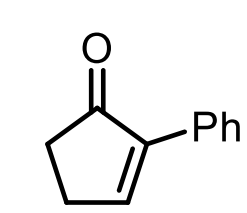

**3l**

$^{13}C$ NMR ($CDCl_3$, 126 MHz)

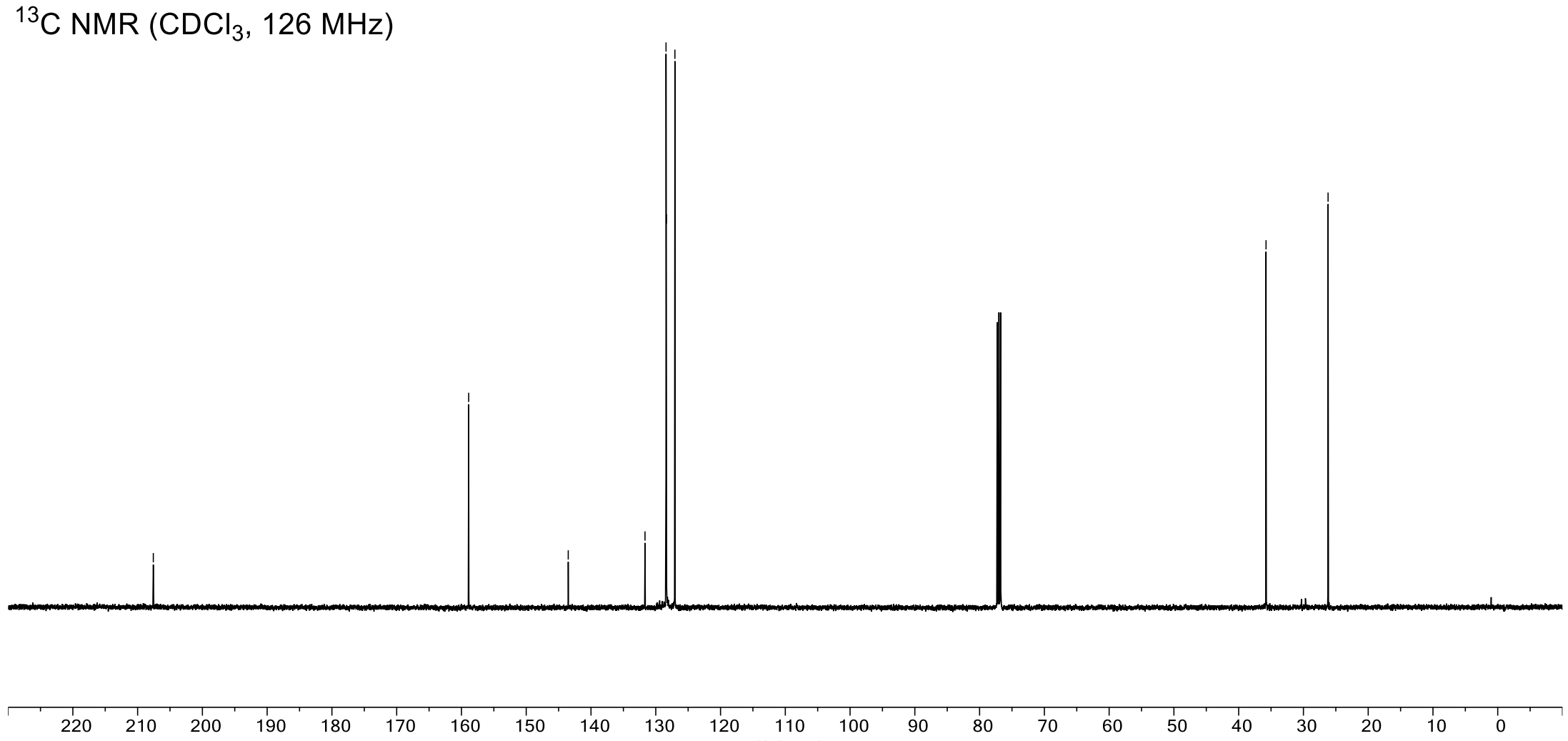

**3m** (90:10 dr)
$^1$H NMR ($CDCl_3$, 400 MHz)

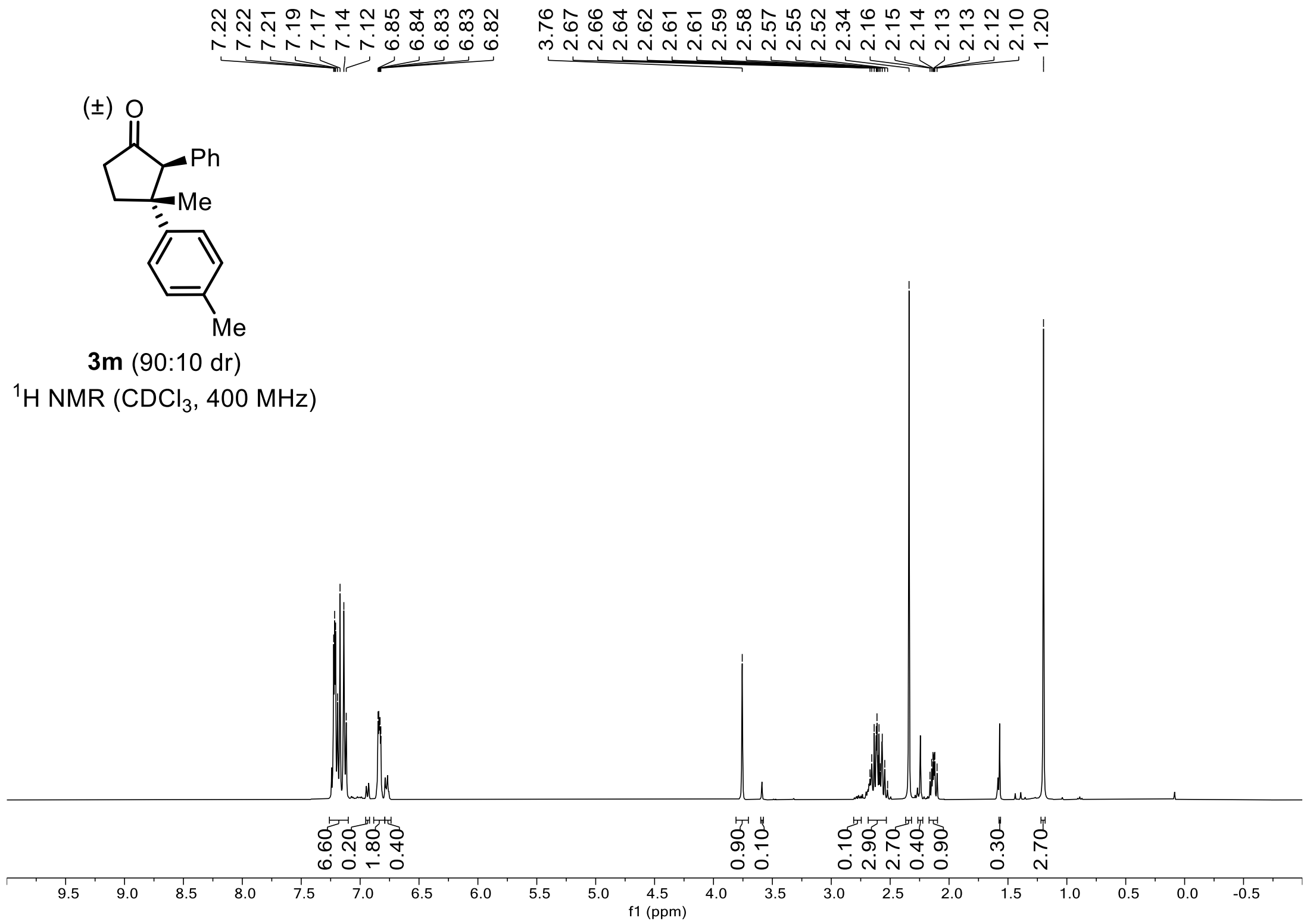


**3m** (90:10 dr)
$^{13}$C NMR ($CDCl_3$, 126 MHz)

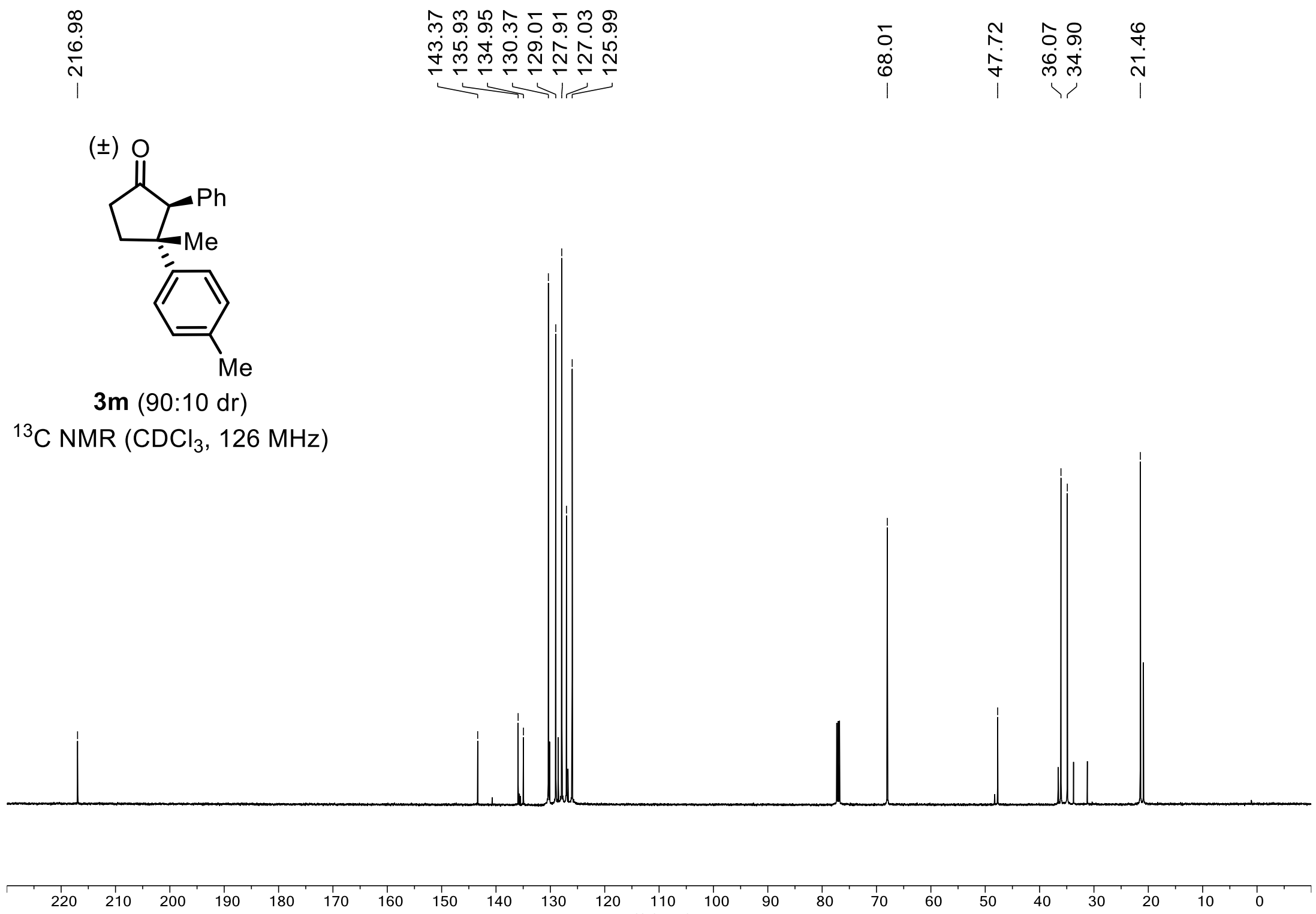

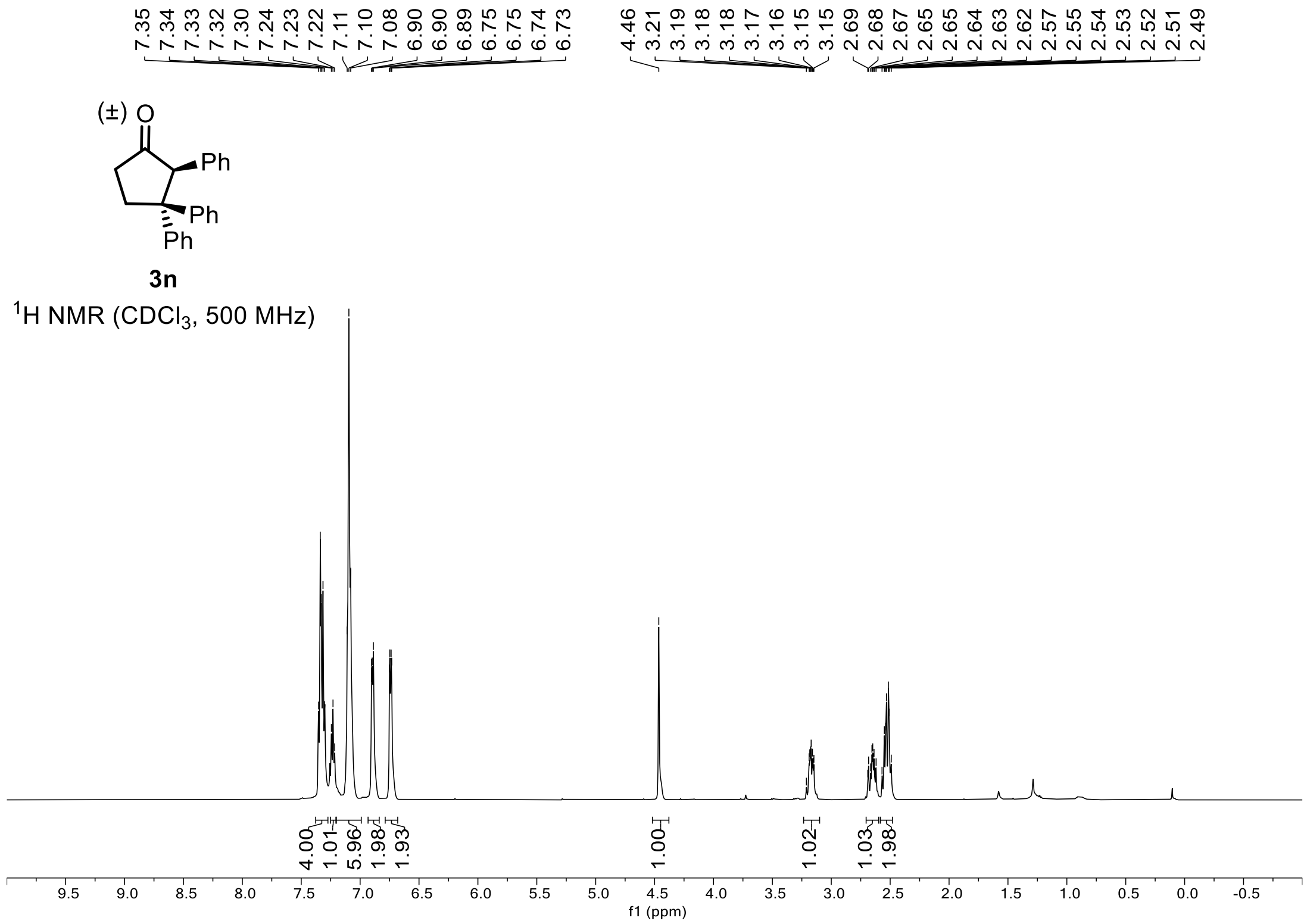

7.35 7.34 7.33 7.32 7.30 7.24 7.23 7.22 7.11 7.10 7.08 6.90 6.90 6.89 6.75 6.74 6.73
4.46 3.21 3.19 3.18 3.18 3.17 3.16 3.15 3.15 2.69 2.68 2.67 2.65 2.65 2.64 2.63 2.62 2.57 2.55 2.54 2.53 2.52 2.51 2.49
(±) O
Ph
Ph
Ph
3n
$^{1}$H NMR (CDCl$_3$, 500 MHz)
4.00 1.01 5.96 1.98 1.93 1.00 1.02 1.03 1.98
9.5 9.0 8.5 8.0 7.5 7.0 6.5 6.0 5.5 5.0 4.5 4.0 3.5 3.0 2.5 2.0 1.5 1.0 0.5 0.0 -0.5
f1 (ppm)


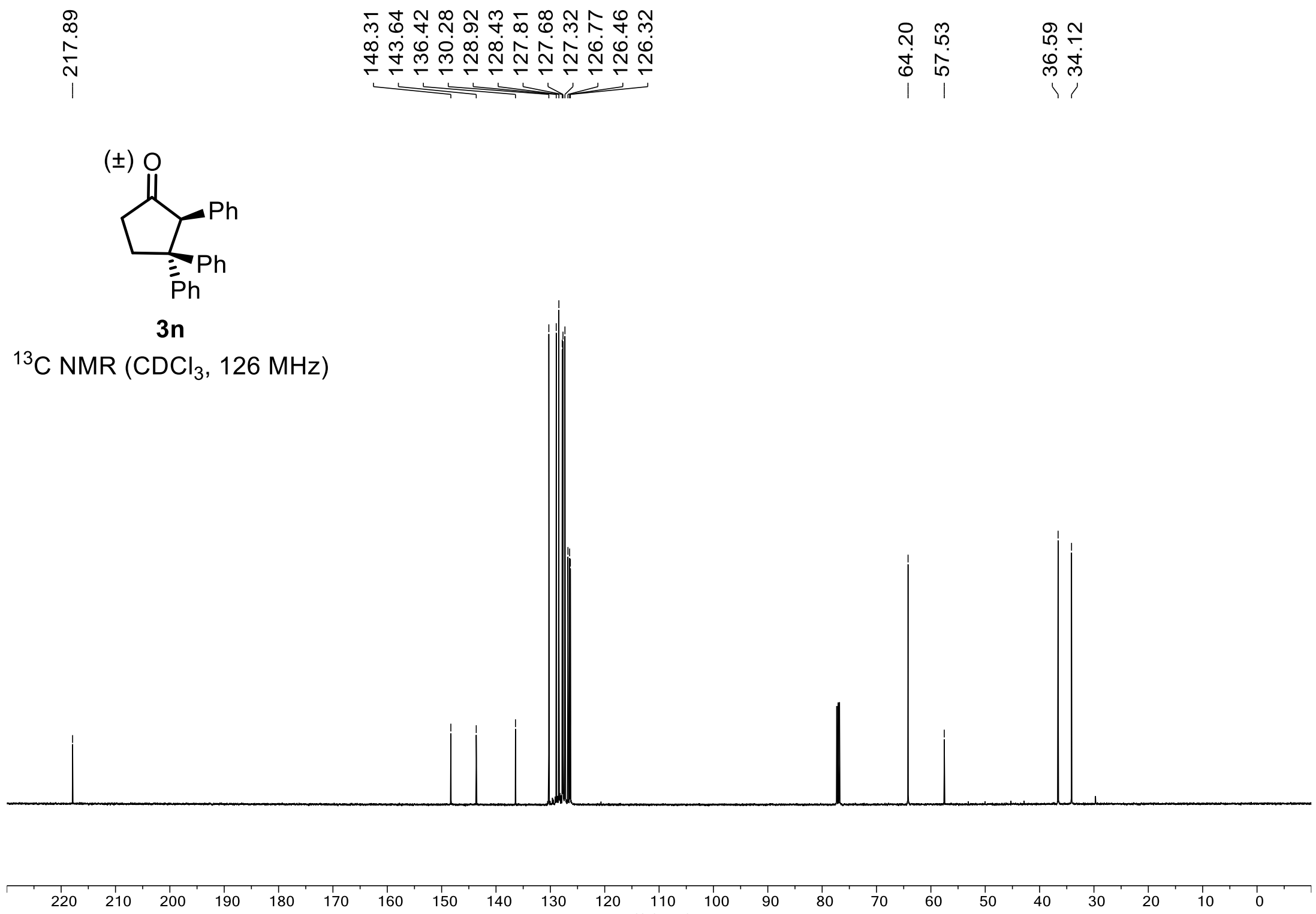

217.89
148.31 143.64 136.42 130.28 128.92 128.43 127.81 127.68 127.32 126.77 126.46 126.32
64.20 57.53
36.59 34.12
(±) O
Ph
Ph
Ph
3n
$^{13}$C NMR (CDCl$_3$, 126 MHz)
220 210 200 190 180 170 160 150 140 130 120 110 100 90 80 70 60 50 40 30 20 10 0
f1 (ppm)

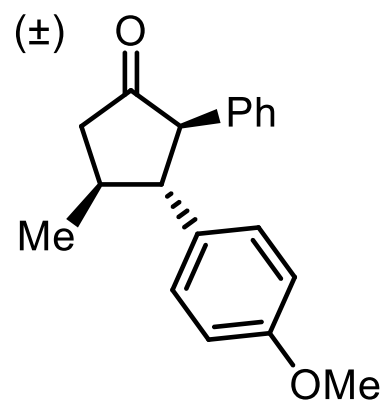


**3o**

$^{1}$H NMR ($CDCl_3$, 500 MHz)

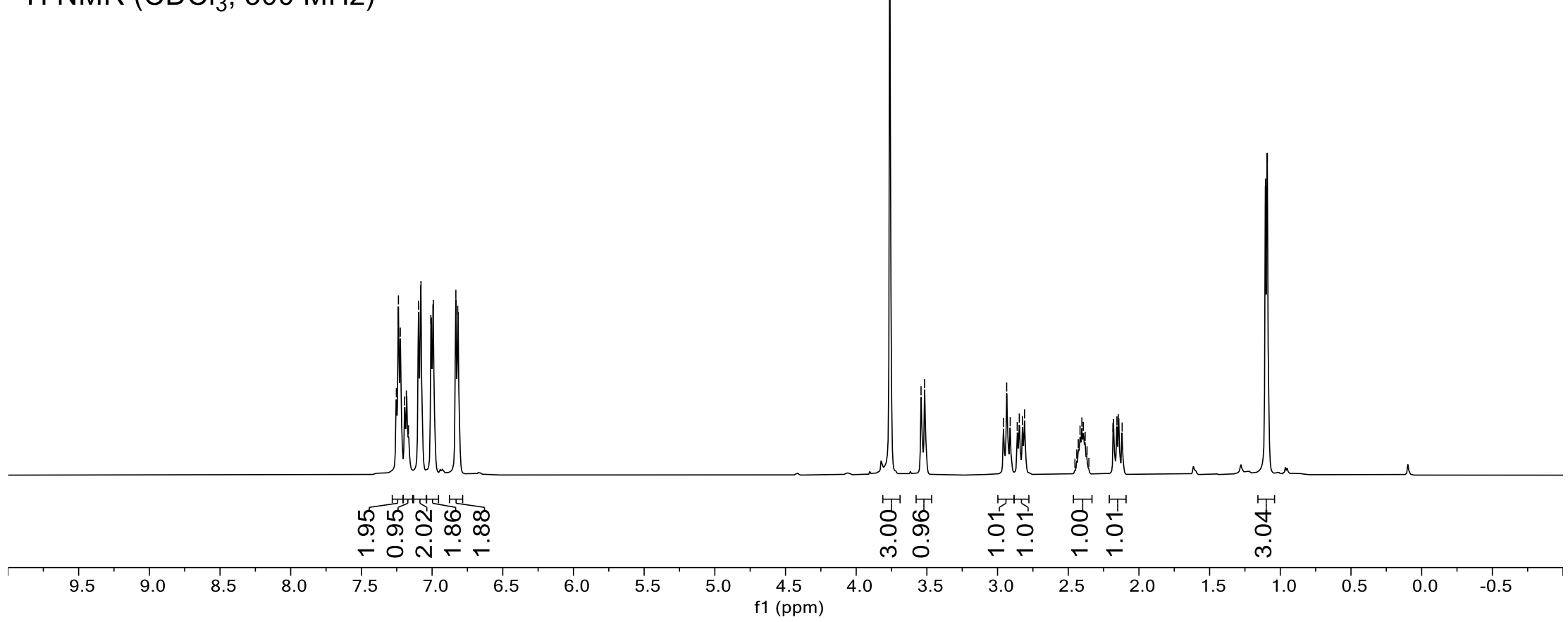


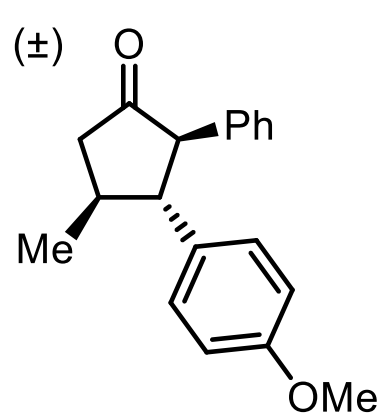


**3o**

$^{13}$C NMR ($CDCl_3$, 126 MHz)

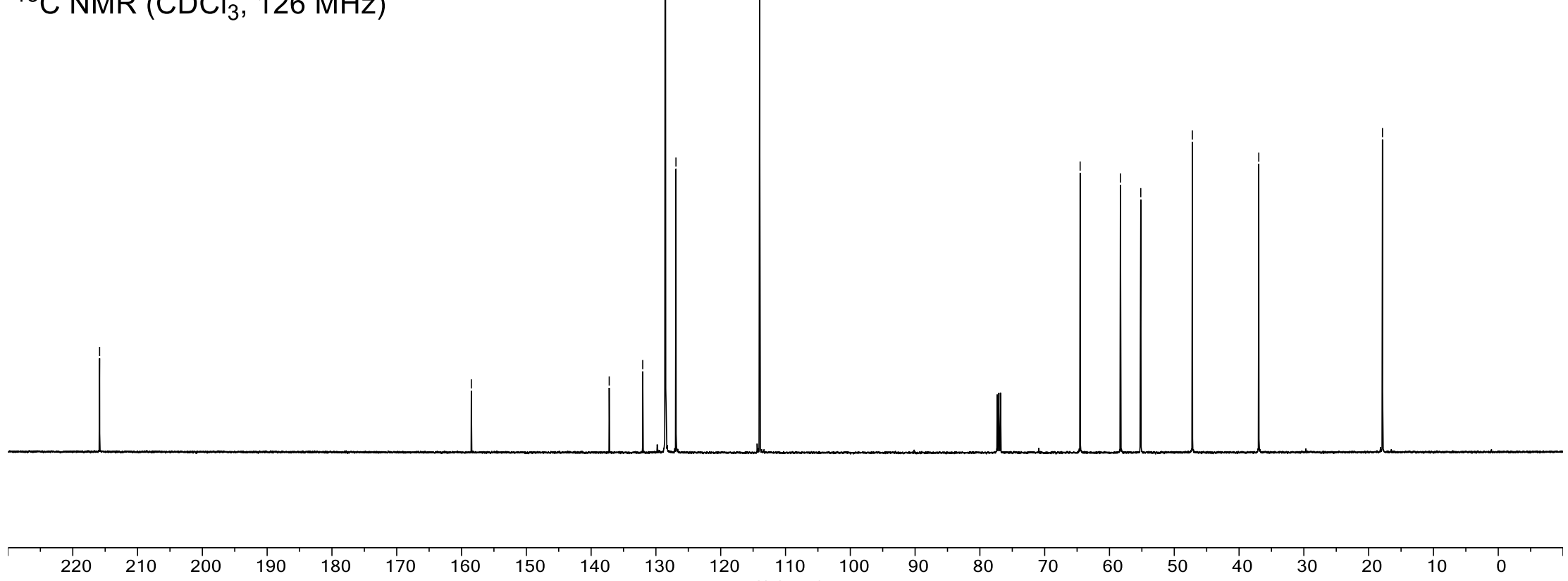

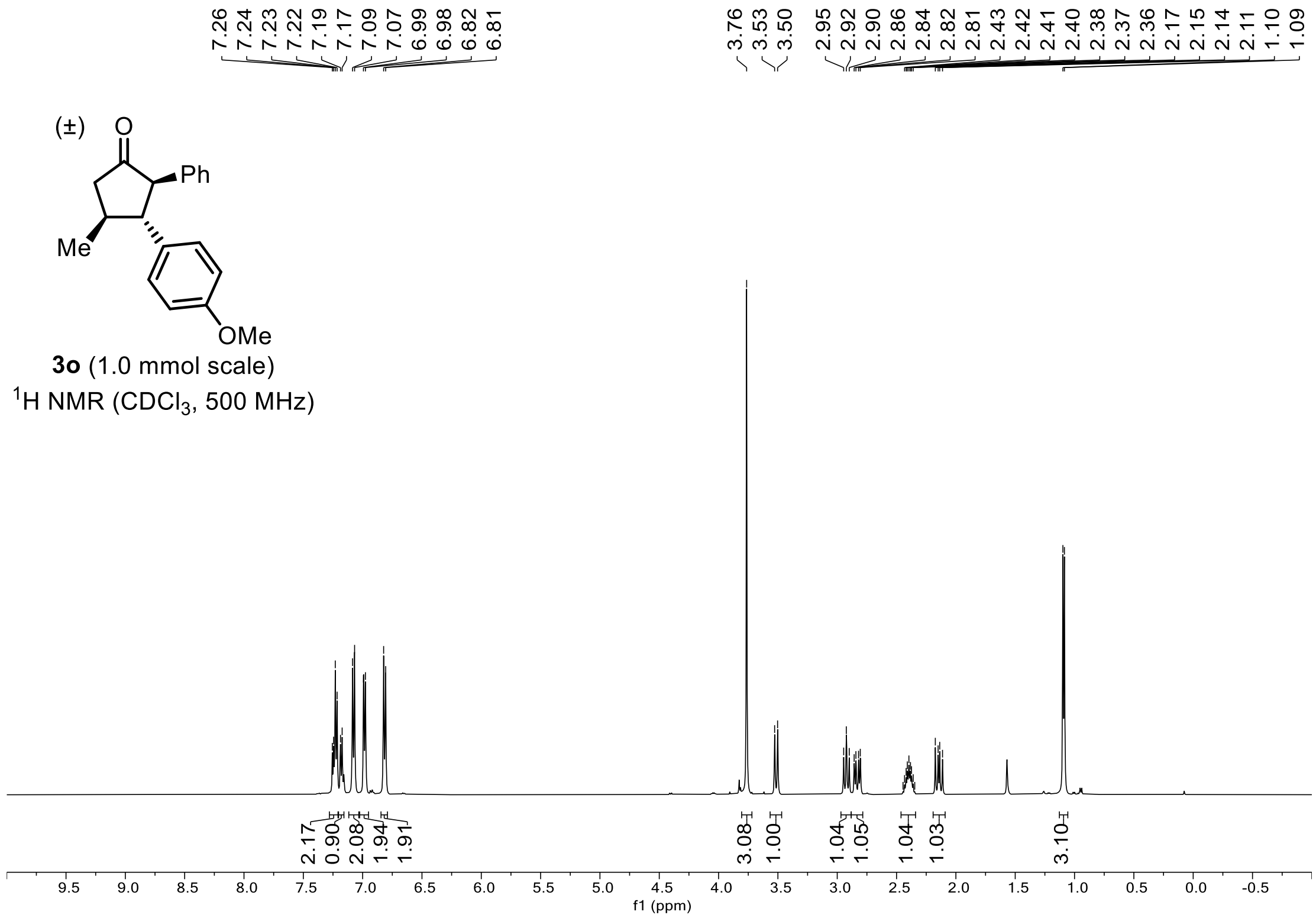


**3o** (1.0 mmol scale)

$^{1}H$ NMR ($CDCl_3$, 500 MHz)

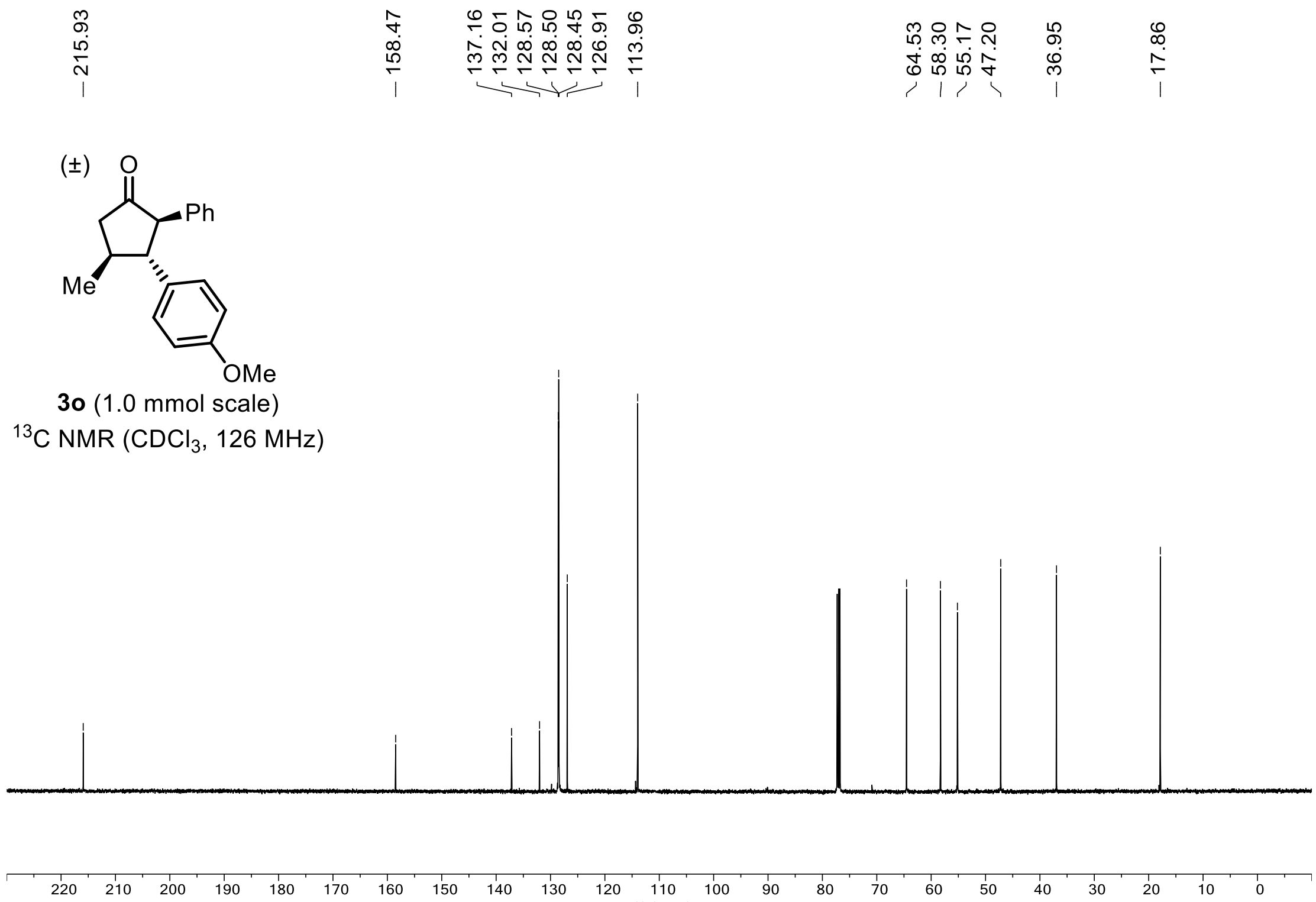


**3o** (1.0 mmol scale)

$^{13}C$ NMR ($CDCl_3$, 126 MHz)

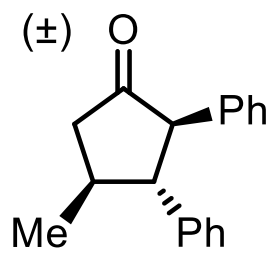


**3p**

$^{1}$H NMR ($CDCl_3$, 400 MHz)

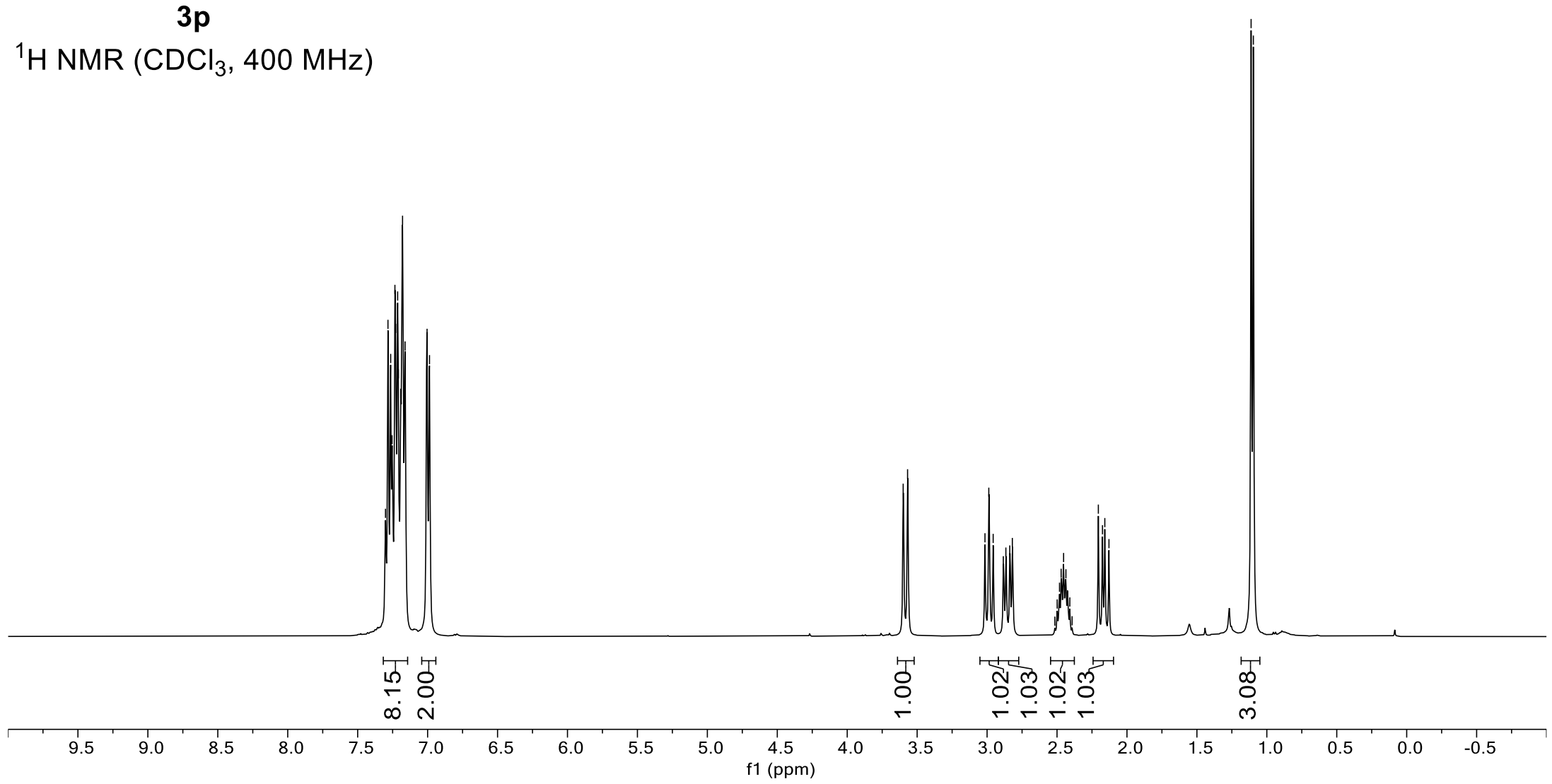


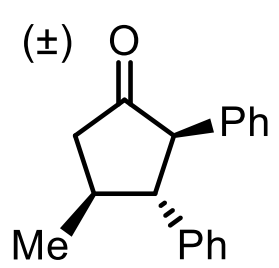


**3p**

$^{13}$C NMR ($CDCl_3$, 126 MHz)

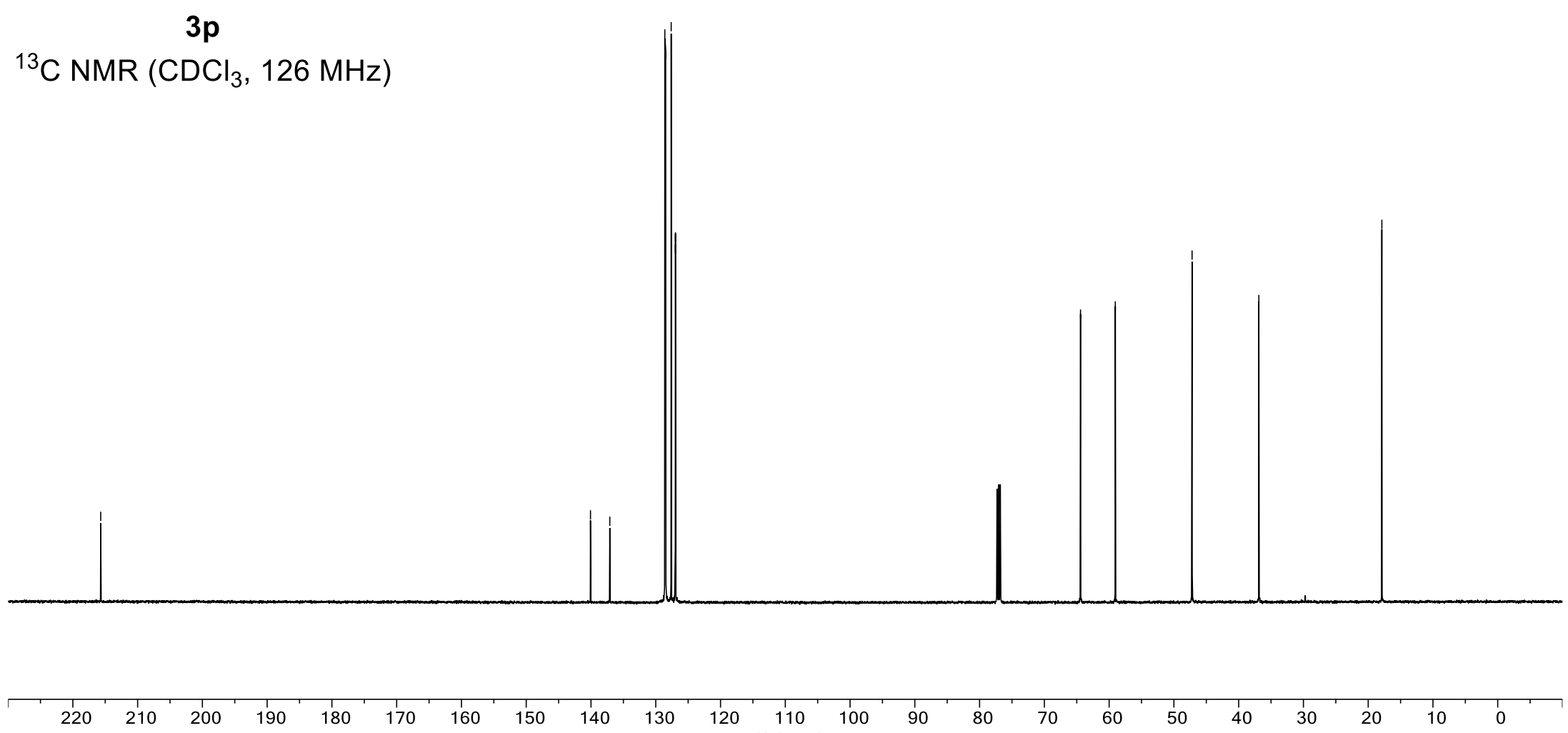

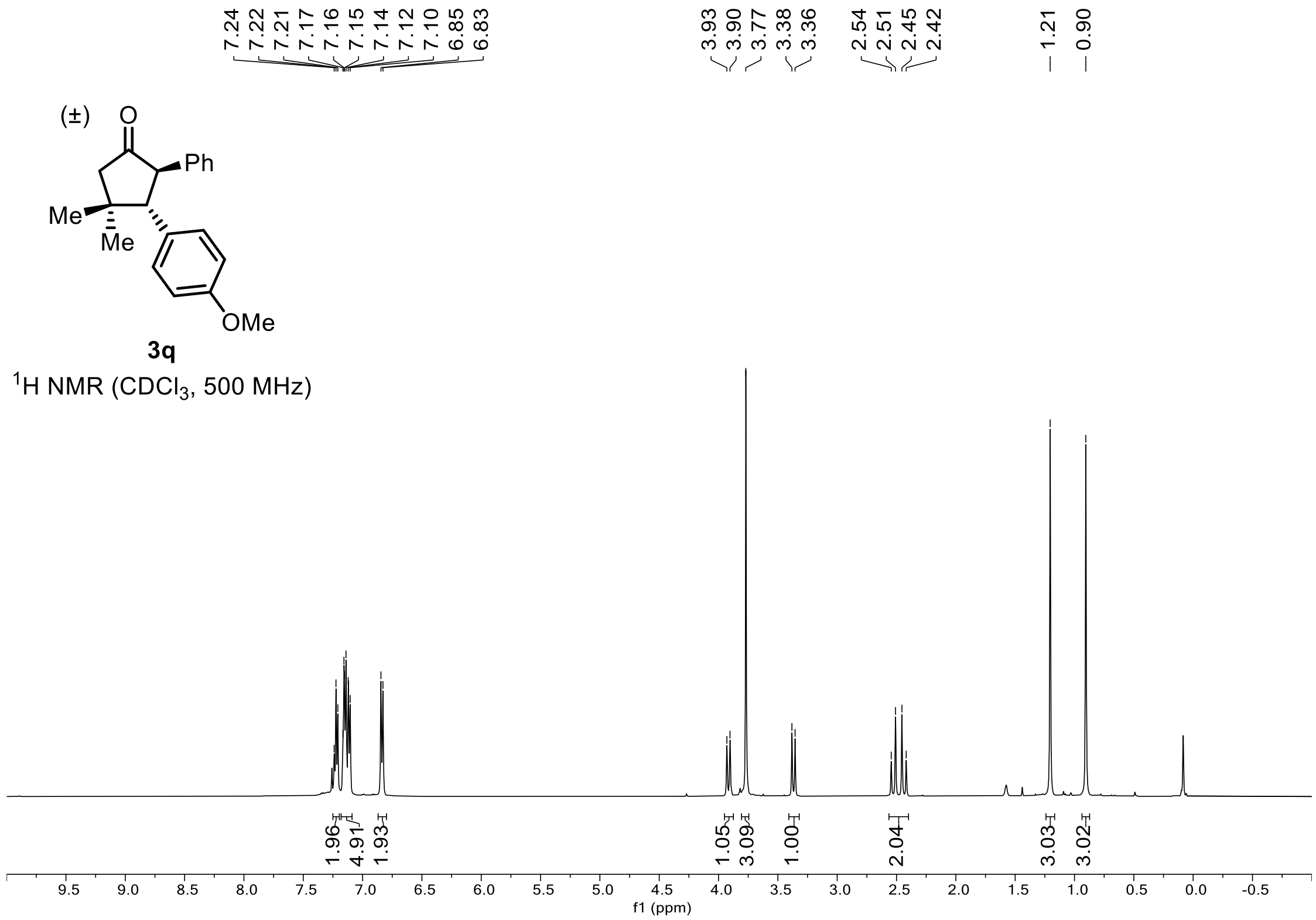
7.24
7.22
7.21
7.17
7.16
7.15
7.14
7.12
7.10
6.85
6.83
3.93
3.90
3.77
3.38
3.36
2.54
2.51
2.45
2.42
1.21
0.90
(±)
O
Ph
Me
Me
OMe
3q
1H NMR (CDCl3, 500 MHz)
1.96
4.91
1.93
1.05
3.09
1.00
2.04
3.03
3.02
f1 (ppm)

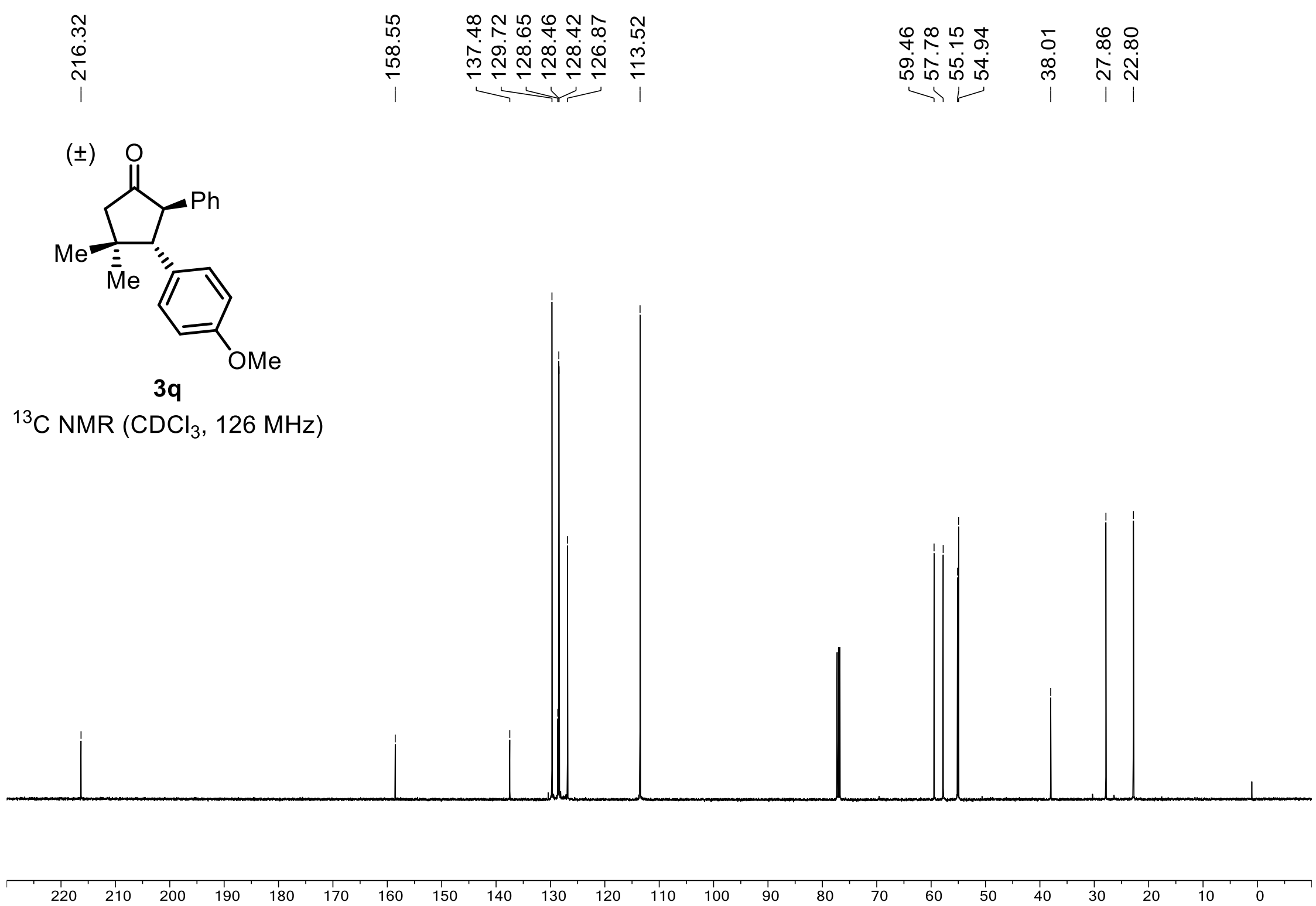
216.32
158.55
137.48
129.72
128.65
128.46
128.42
126.87
113.52
59.46
57.78
55.15
54.94
38.01
27.86
22.80
(±)
O
Ph
Me
Me
OMe
3q
13C NMR (CDCl3, 126 MHz)
f1 (ppm)

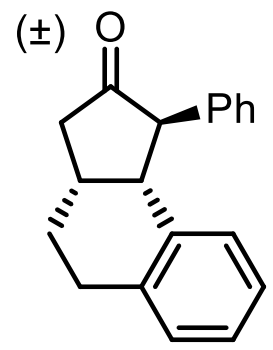

**3r**

$^{1}$H NMR ($CDCl_3$, 400 MHz)

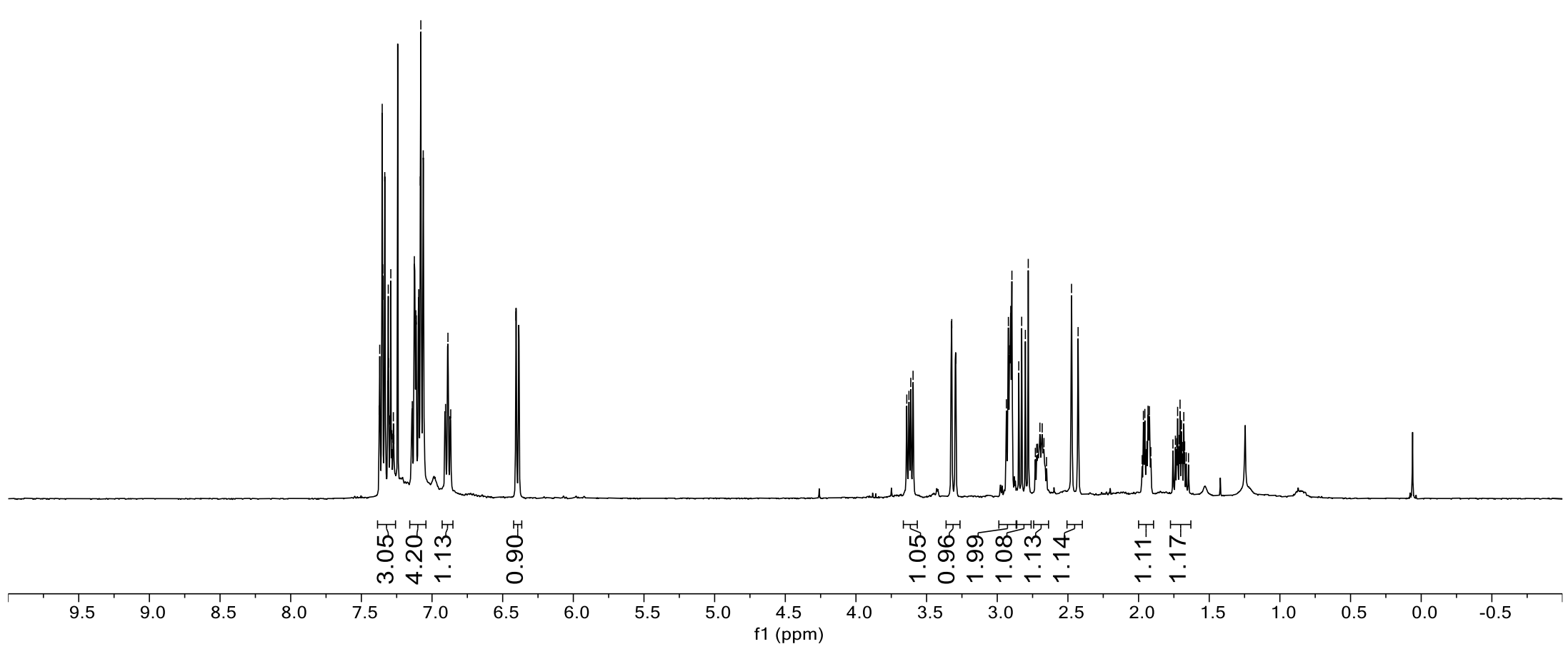


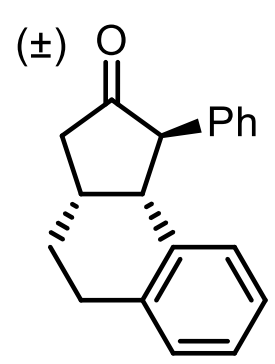

**3r**

$^{13}$C NMR ($CDCl_3$, 126 MHz)

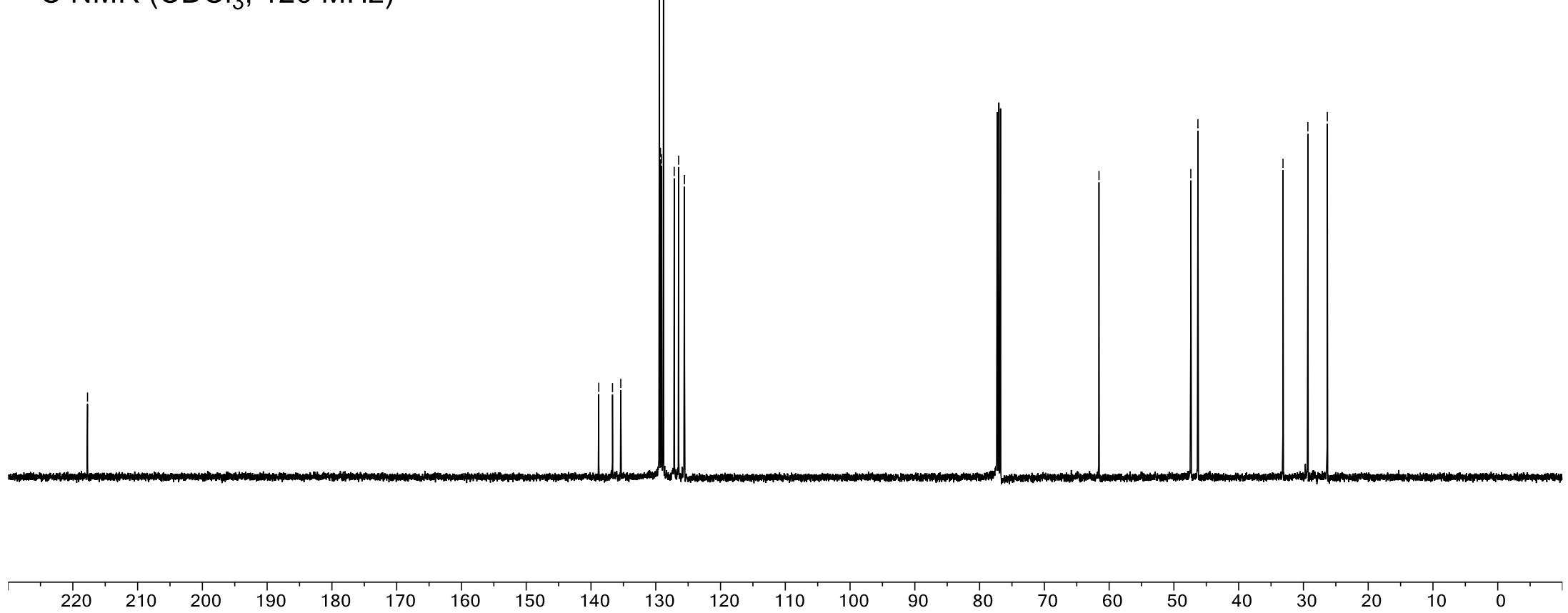

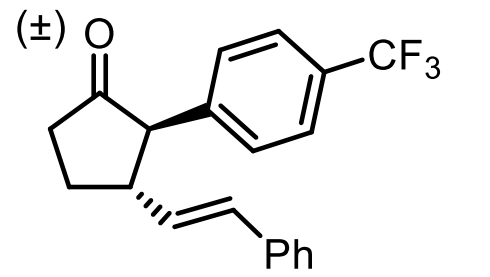


**3s**

$^{1}$H NMR ($CDCl_3$, 500 MHz)

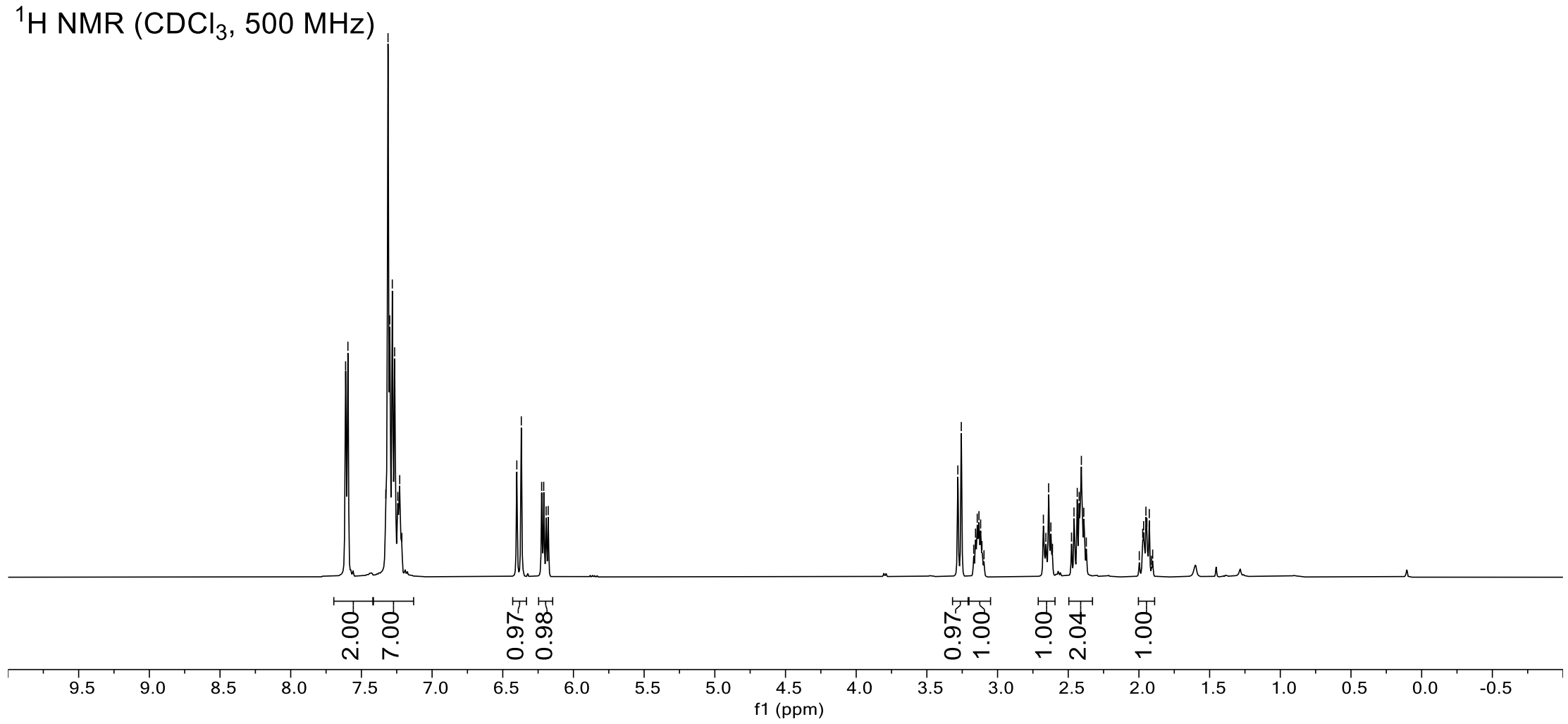


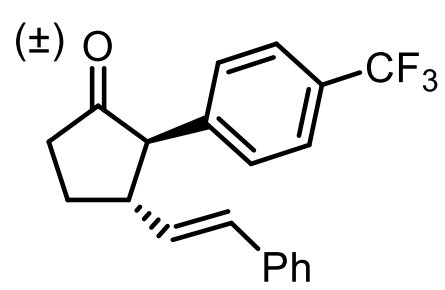


**3s**

$^{13}$C NMR ($CDCl_3$, 126 MHz)

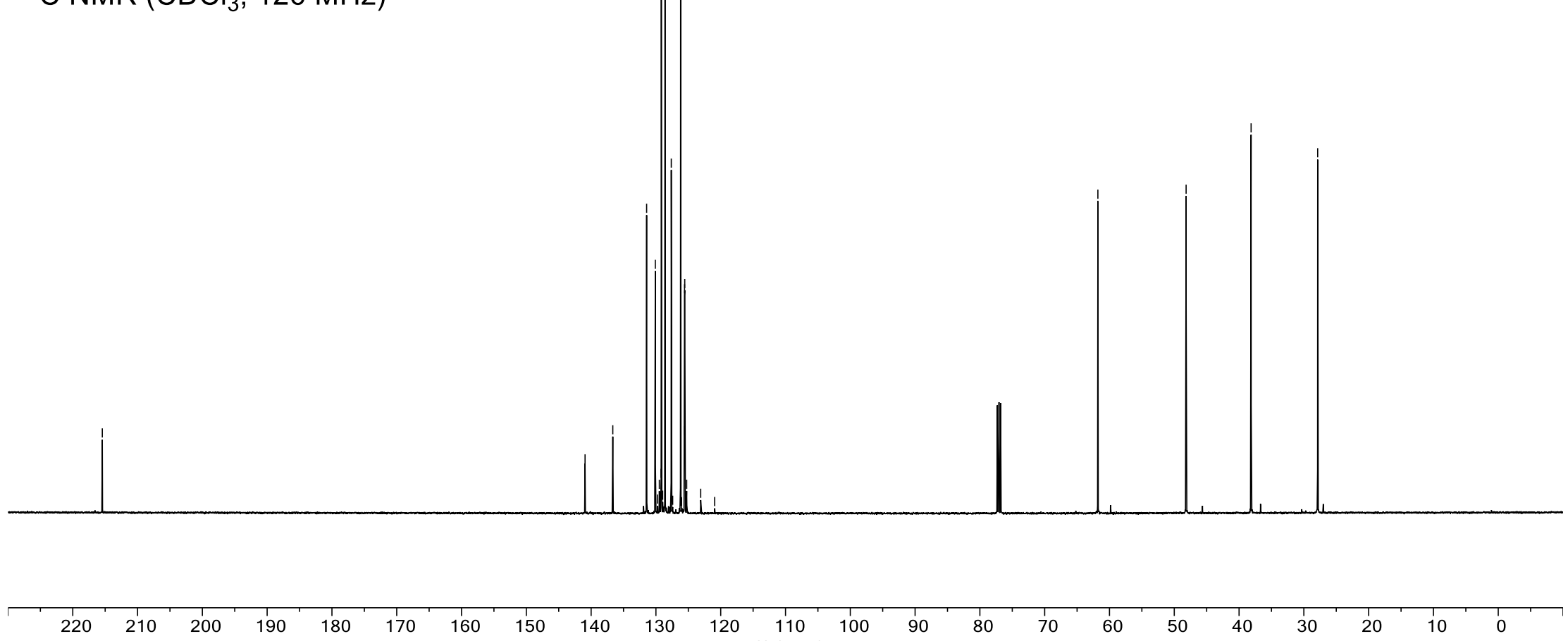

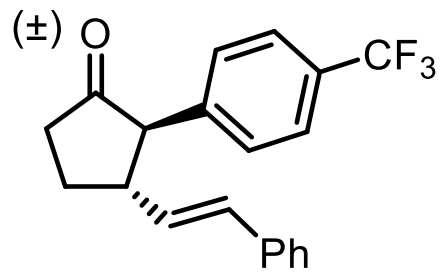


**3s**

$^{19}$F NMR (CDCl$_3$, 471 MHz)

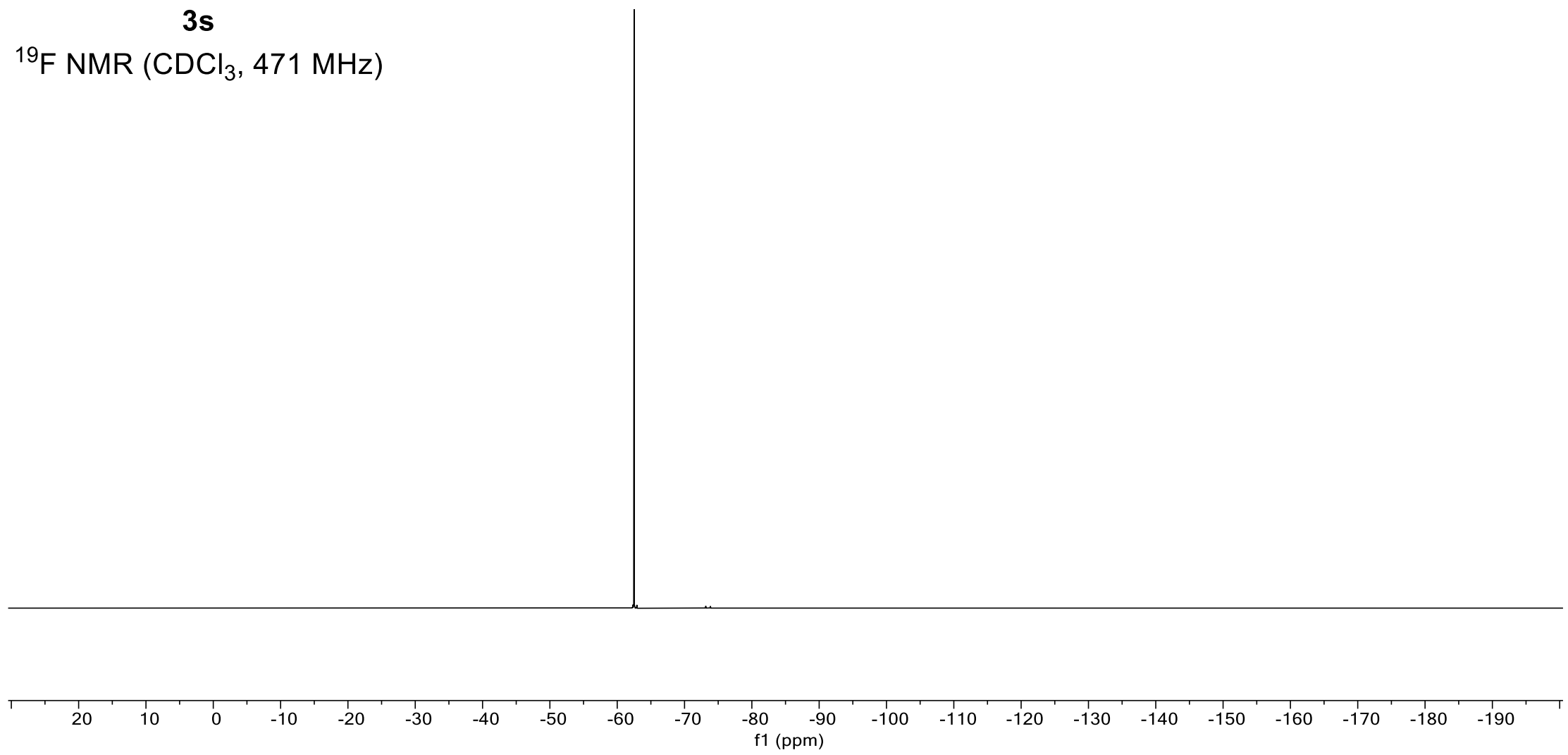

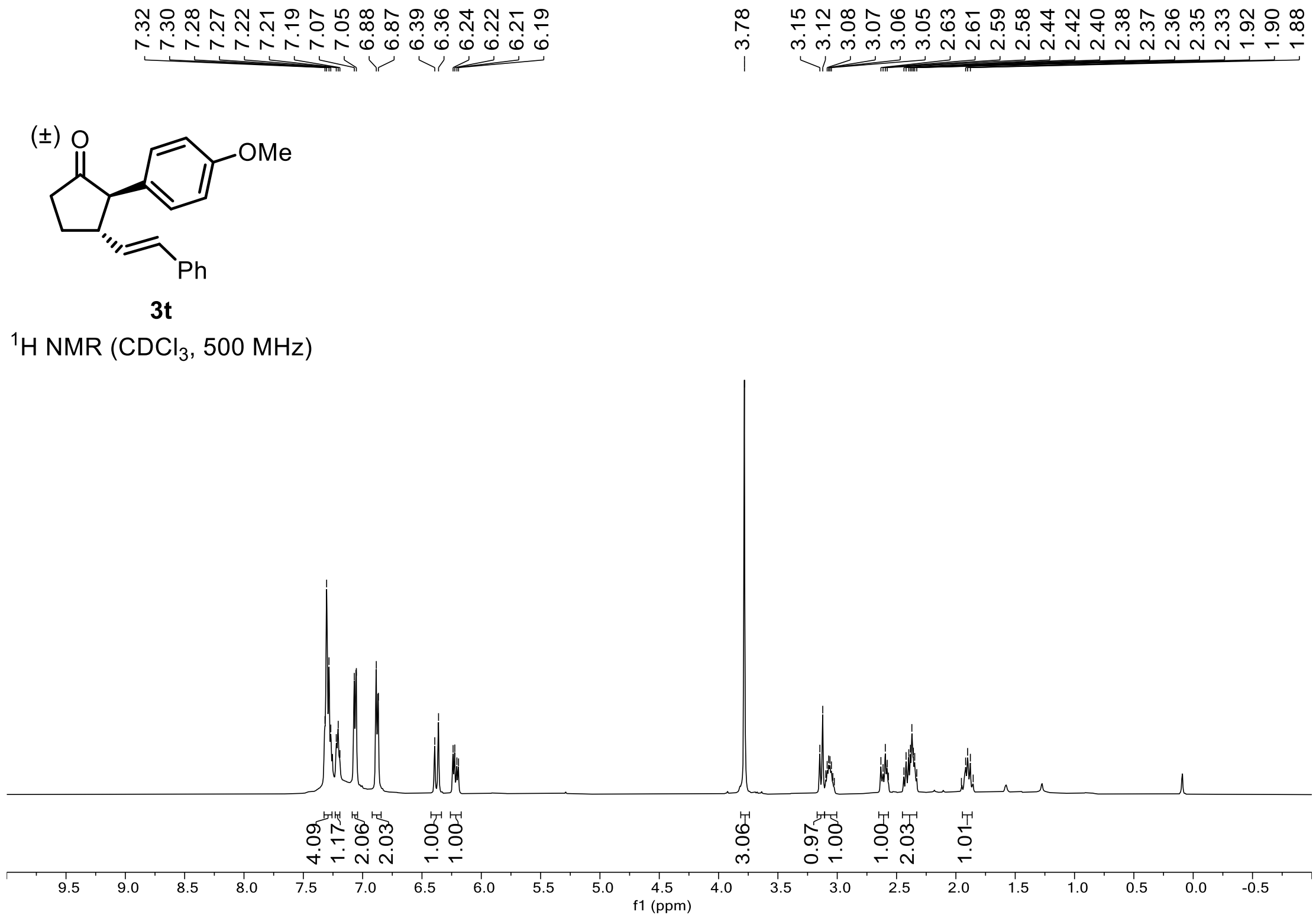
7.32 7.30 7.28 7.27 7.22 7.21 7.19 7.07 7.05 6.88 6.87 6.39 6.36 6.24 6.22 6.21 6.19
3.78
3.15 3.12 3.08 3.07 3.06 3.05 2.63 2.61 2.59 2.58 2.44 2.42 2.40 2.38 2.37 2.36 2.35 2.33 1.92 1.90 1.88
(±)
O
OMe
Ph
3t
1H NMR (CDCl3, 500 MHz)
4.09 1.17 2.06 2.03 1.00 1.00 3.06 0.97 1.00 1.00 2.03 1.01
9.5 9.0 8.5 8.0 7.5 7.0 6.5 6.0 5.5 5.0 4.5 4.0 3.5 3.0 2.5 2.0 1.5 1.0 0.5 0.0 -0.5
f1 (ppm)

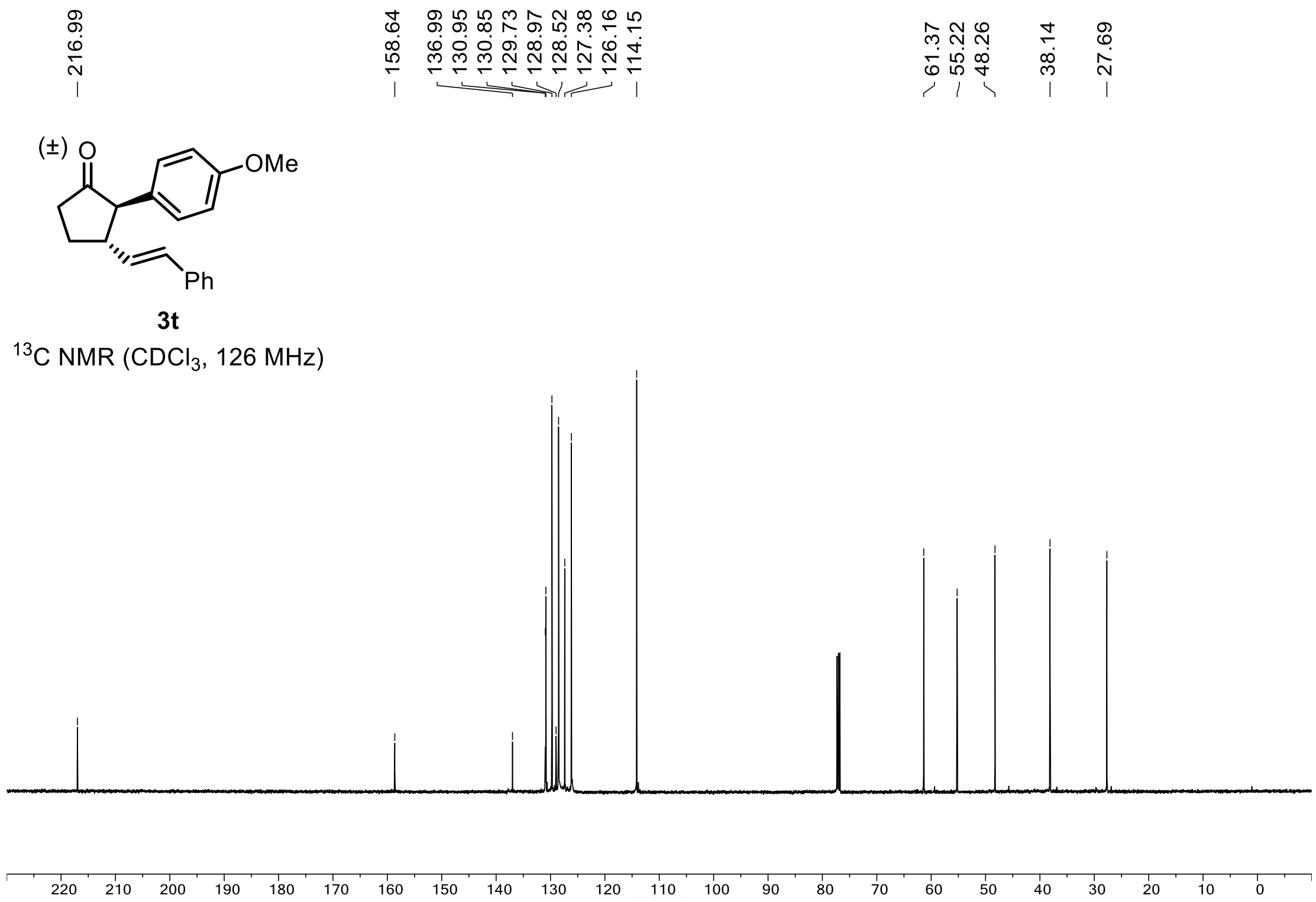
216.99
158.64
136.99 130.95 130.85 129.73 128.97 128.52 127.38 126.16
114.15
61.37 55.22 48.26
38.14
27.69
(±)
O
OMe
Ph
3t
13C NMR (CDCl3, 126 MHz)
220 210 200 190 180 170 160 150 140 130 120 110 100 90 80 70 60 50 40 30 20 10 0
f1 (ppm)

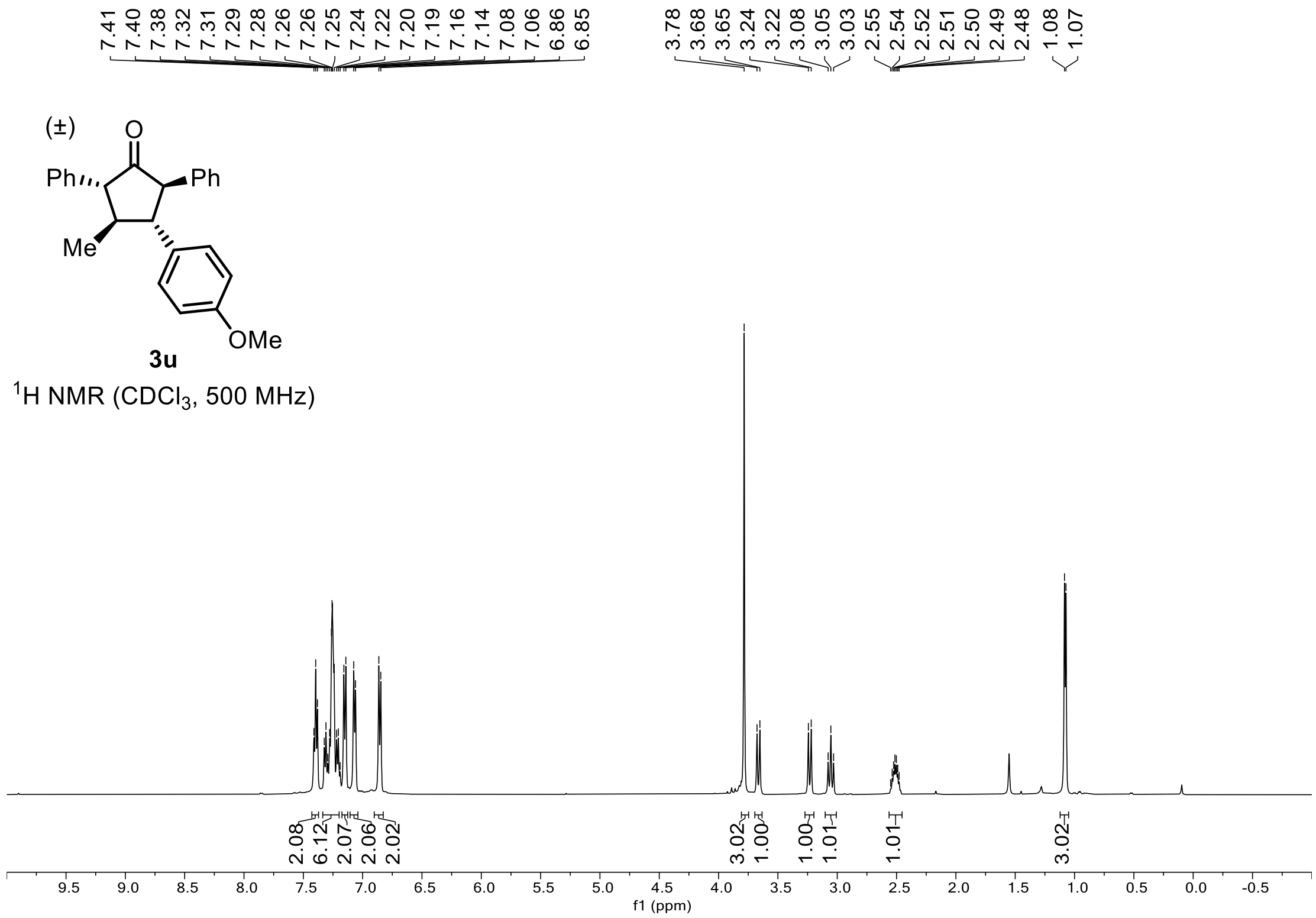
7.41 7.40 7.38 7.32 7.31 7.29 7.28 7.26 7.26 7.25 7.24 7.22 7.20 7.19 7.16 7.14 7.08 7.06 6.86 6.85
3.78 3.68 3.65 3.24 3.22 3.08 3.05 3.03 2.55 2.54 2.52 2.51 2.50 2.49 2.48 1.08 1.07
(±)
O
Ph
Ph
Me
OMe
3u
¹H NMR (CDCl₃, 500 MHz)
2.08 6.12 2.07 2.06 2.02
3.02 1.00 1.00 1.01 1.01 3.02
9.5 9.0 8.5 8.0 7.5 7.0 6.5 6.0 5.5 5.0 4.5 4.0 3.5 3.0 2.5 2.0 1.5 1.0 0.5 0.0 -0.5
f1 (ppm)

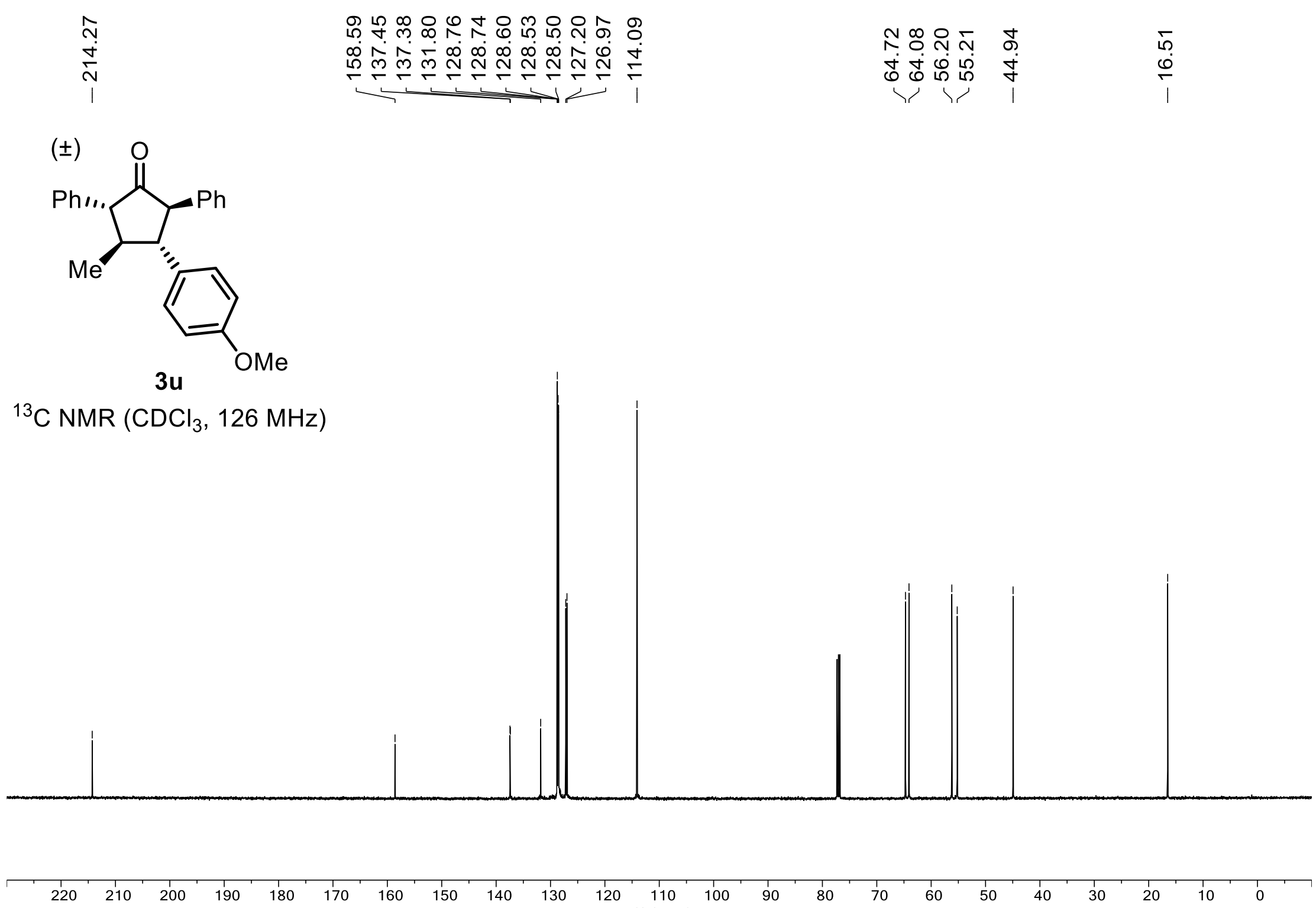
214.27
158.59 137.45 137.38 131.80 128.76 128.74 128.60 128.53 128.50 127.20 126.97 114.09
64.72 64.08 56.20 55.21 44.94 16.51
(±)
O
Ph
Ph
Me
OMe
3u
¹³C NMR (CDCl₃, 126 MHz)
220 210 200 190 180 170 160 150 140 130 120 110 100 90 80 70 60 50 40 30 20 10 0
f1 (ppm)

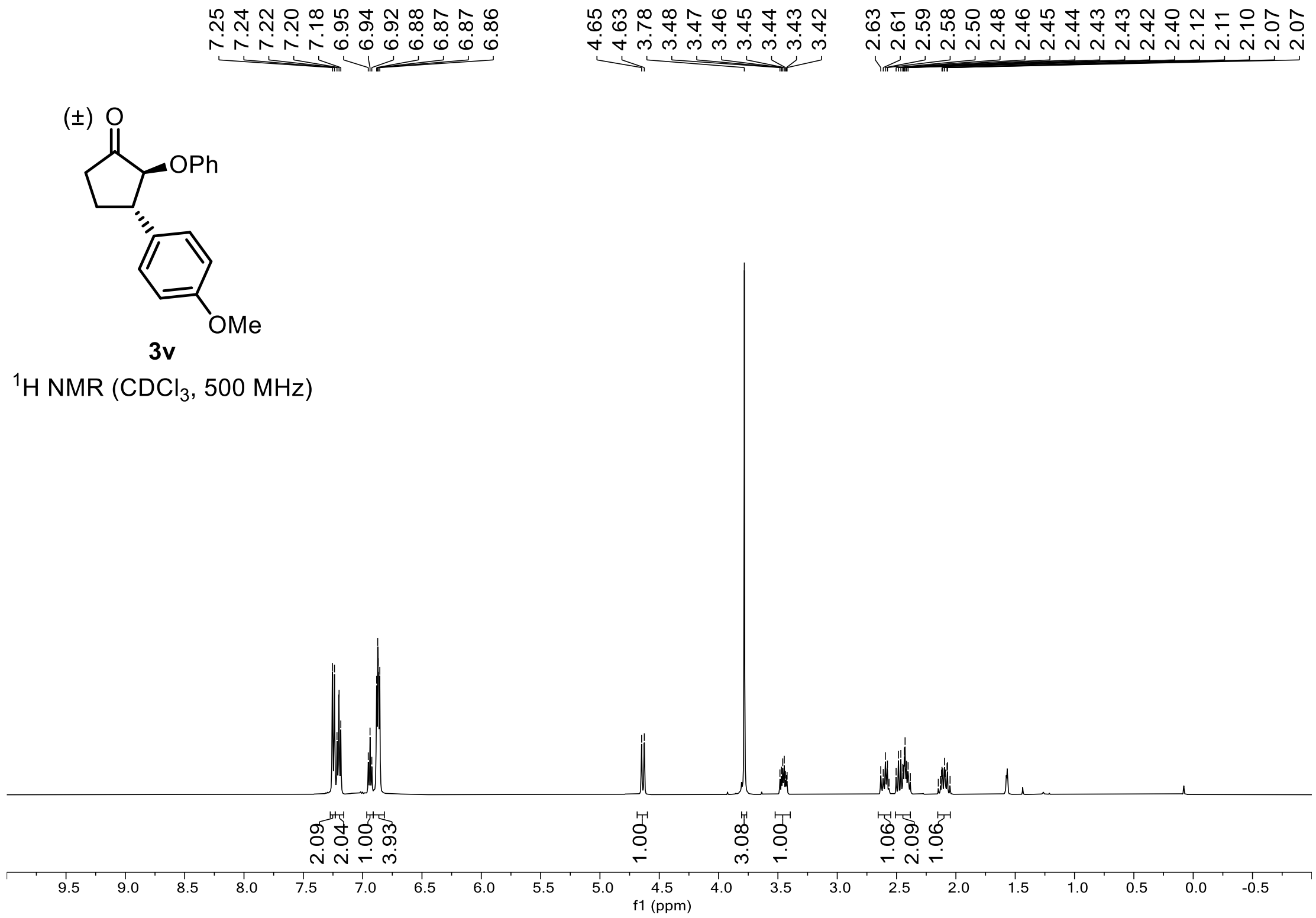
7.25 7.24 7.22 7.20 7.18 6.95 6.94 6.92 6.88 6.87 6.87 6.86
4.65 4.63 3.78 3.48 3.47 3.46 3.45 3.44 3.43 3.42
2.63 2.61 2.59 2.58 2.50 2.48 2.46 2.45 2.44 2.43 2.43 2.42 2.40 2.12 2.11 2.10 2.07 2.07
(±)
O
OPh
OMe
3v
¹H NMR (CDCl₃, 500 MHz)
2.09 2.04 1.00 3.93 1.00 3.08 1.00 1.06 2.09 1.06
9.5 9.0 8.5 8.0 7.5 7.0 6.5 6.0 5.5 5.0 4.5 4.0 3.5 3.0 2.5 2.0 1.5 1.0 0.5 0.0 -0.5
f1 (ppm)

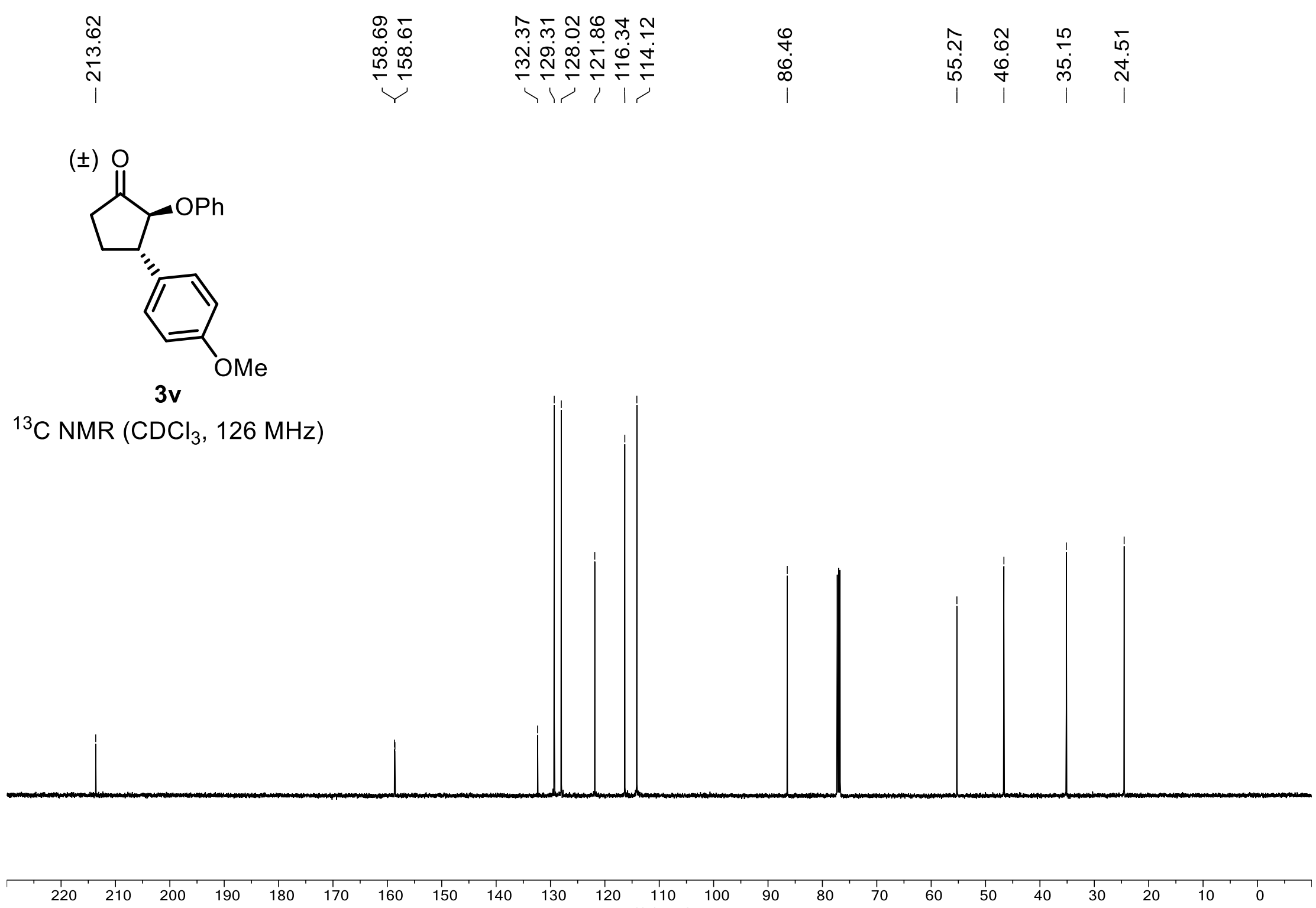
213.62
158.69 158.61
132.37 129.31 128.02 121.86 116.34 114.12
86.46
55.27 46.62 35.15 24.51
(±)
O
OPh
OMe
3v
¹³C NMR (CDCl₃, 126 MHz)
220 210 200 190 180 170 160 150 140 130 120 110 100 90 80 70 60 50 40 30 20 10 0
f1 (ppm)

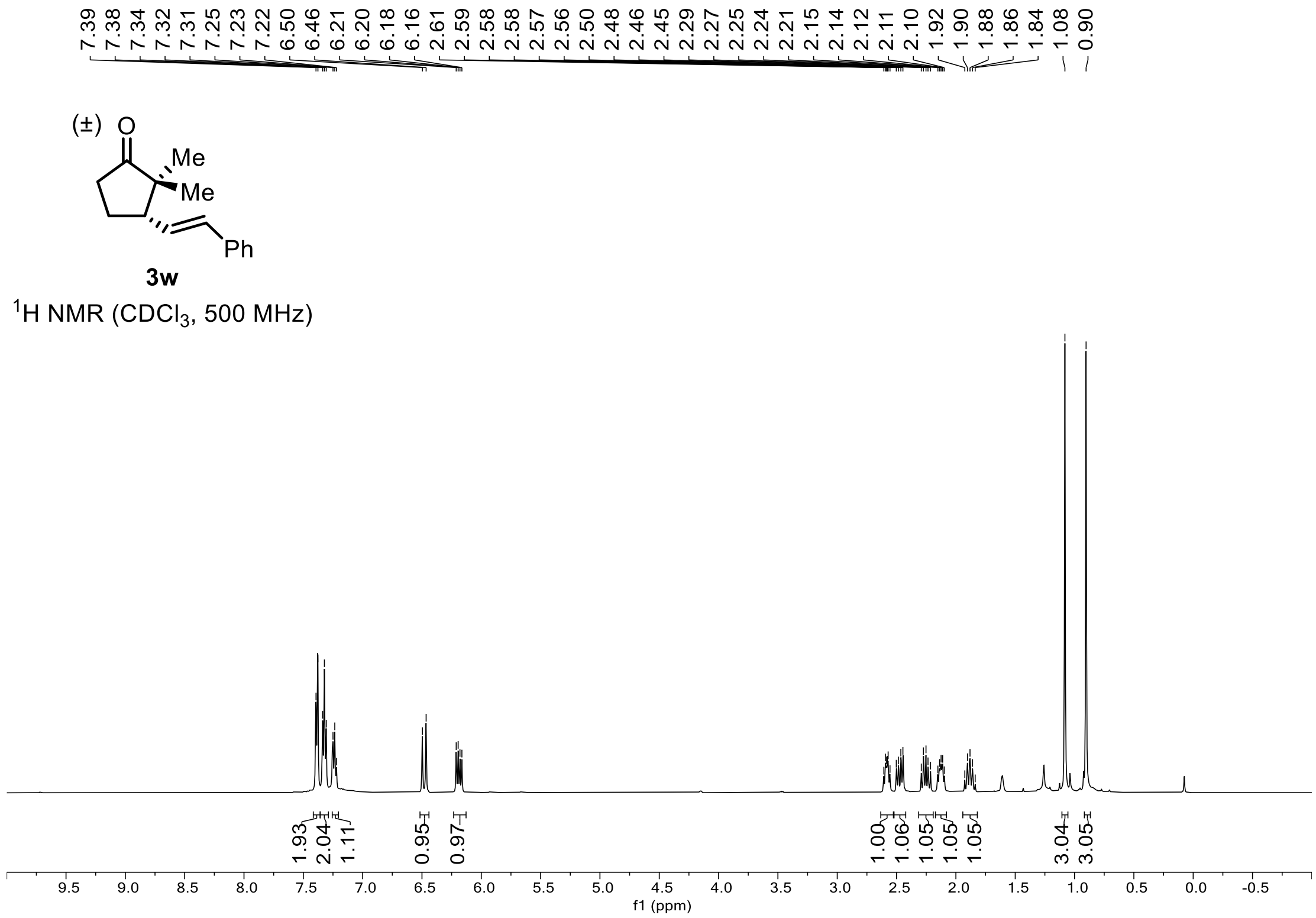


**3w**

$^{1}$H NMR ($CDCl_3$, 500 MHz)

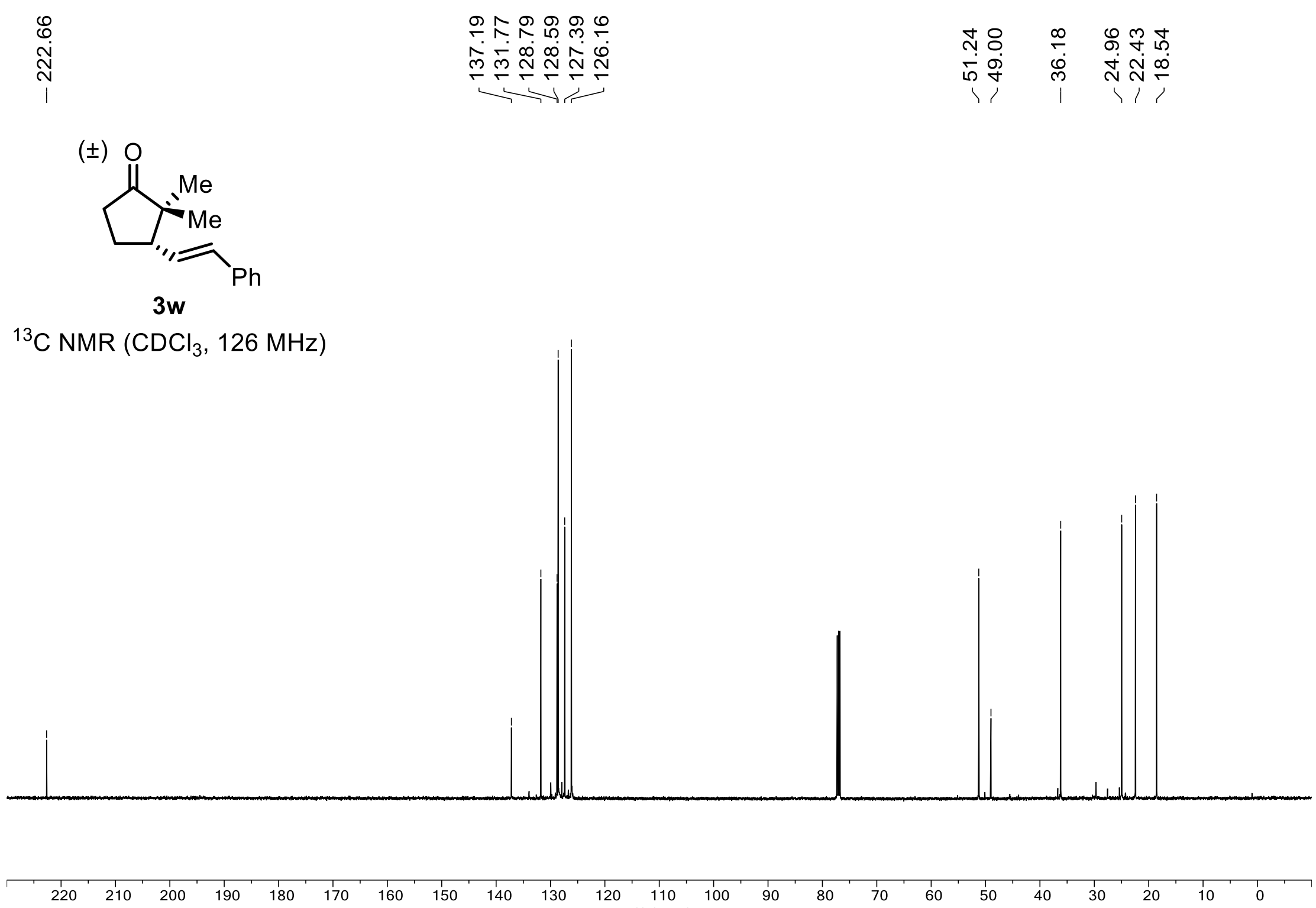


**3w**

$^{13}$C NMR ($CDCl_3$, 126 MHz)

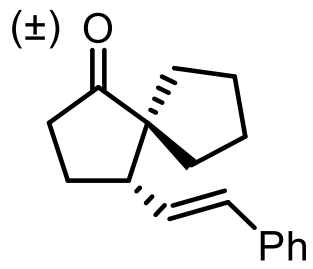


**3x**

$^{1}$H NMR ($CDCl_3$, 400 MHz)

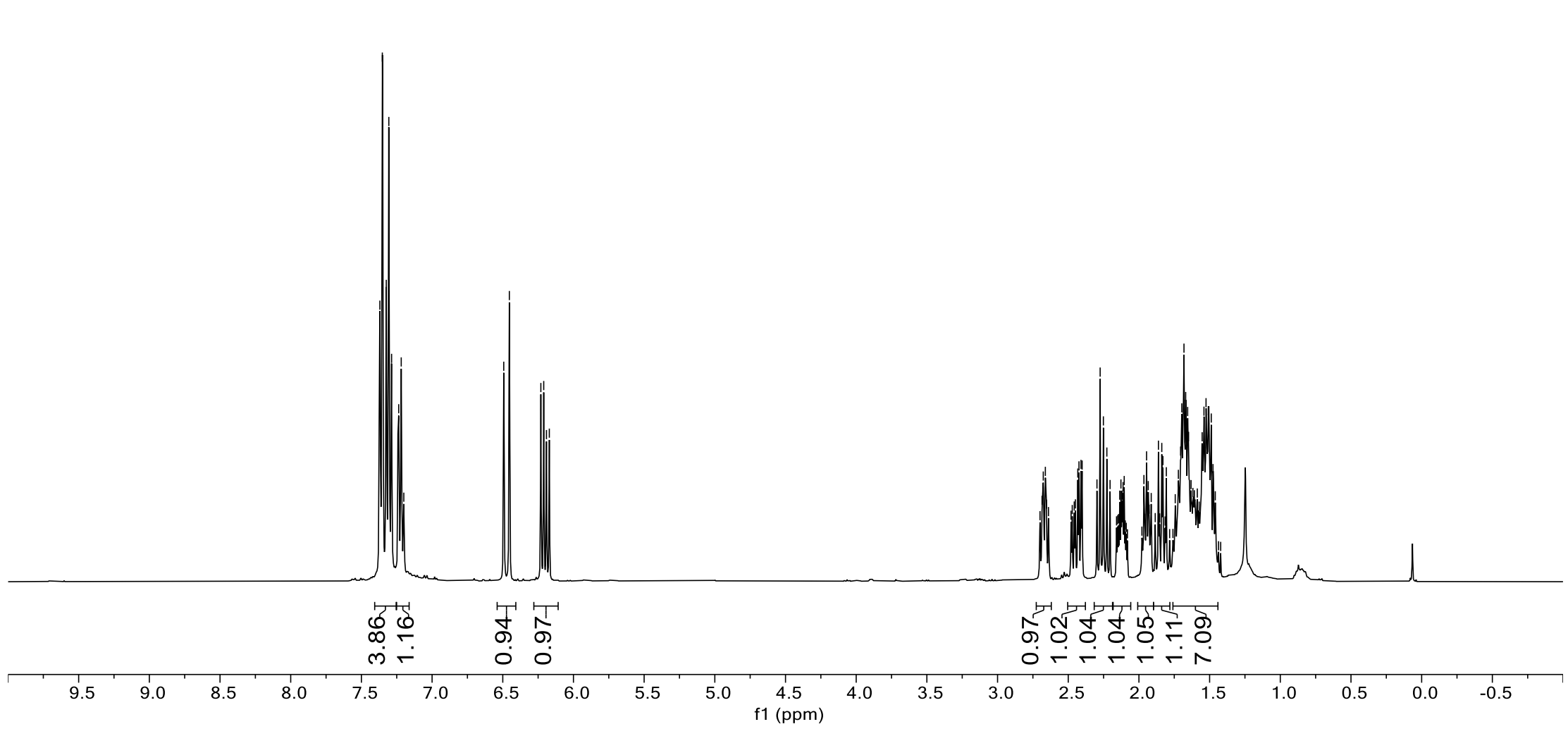


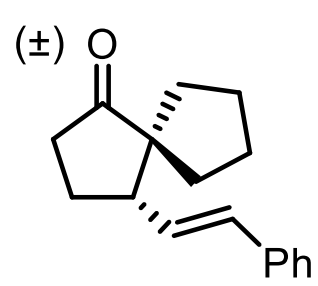


**3x**

$^{13}$C NMR ($CDCl_3$, 126 MHz)

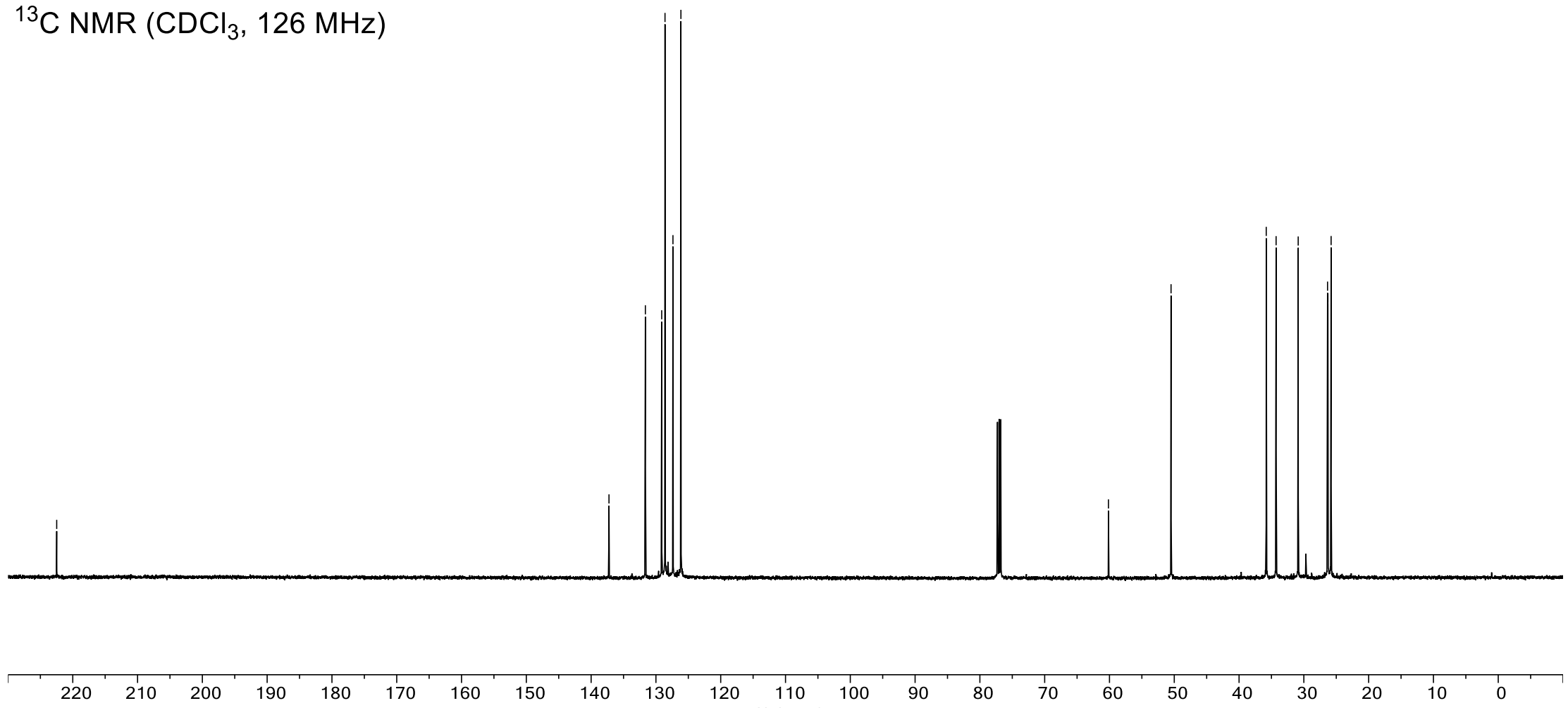

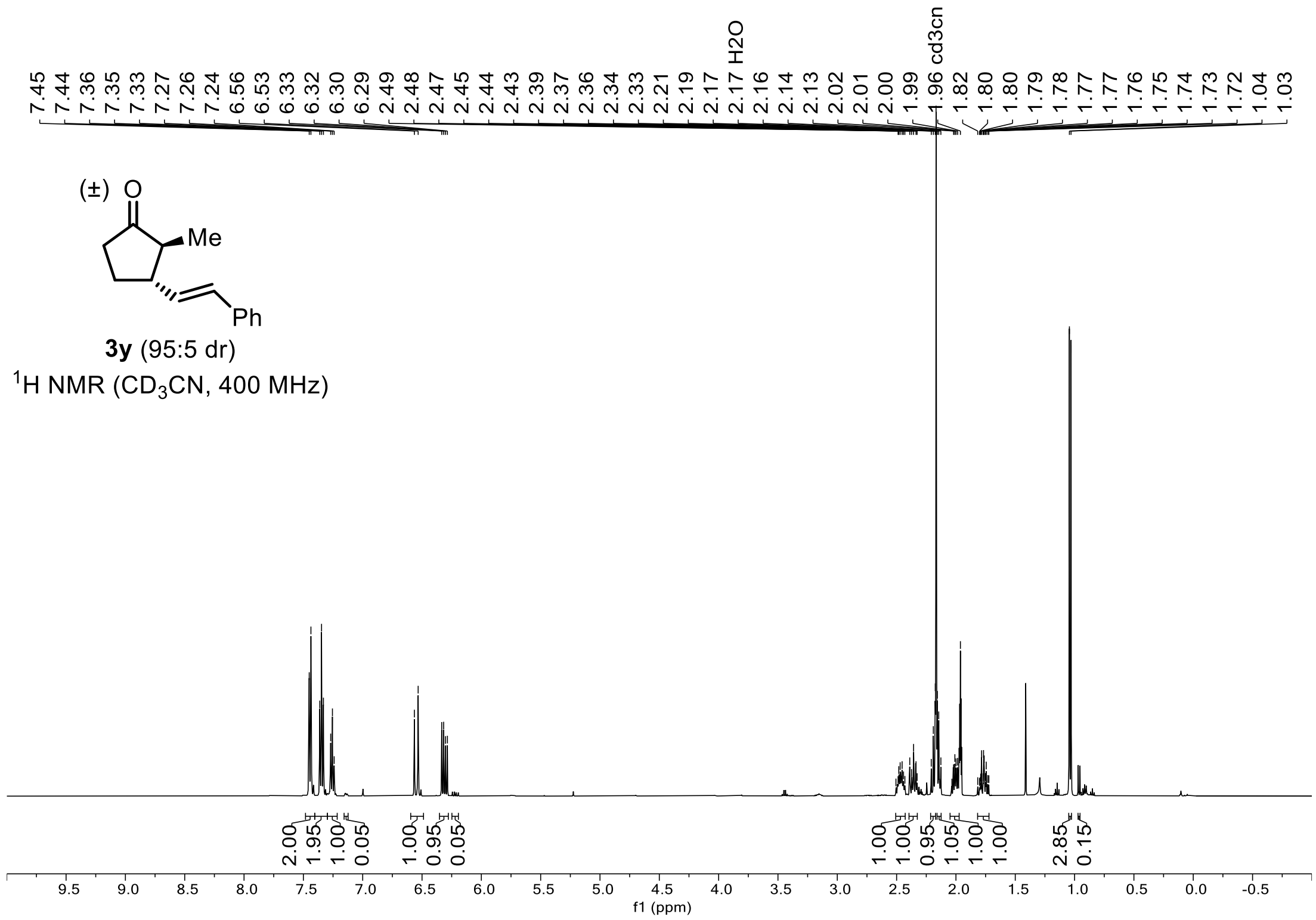


**3y** (95:5 dr)

$^{1}H$ NMR ($CD_3CN$, 400 MHz)

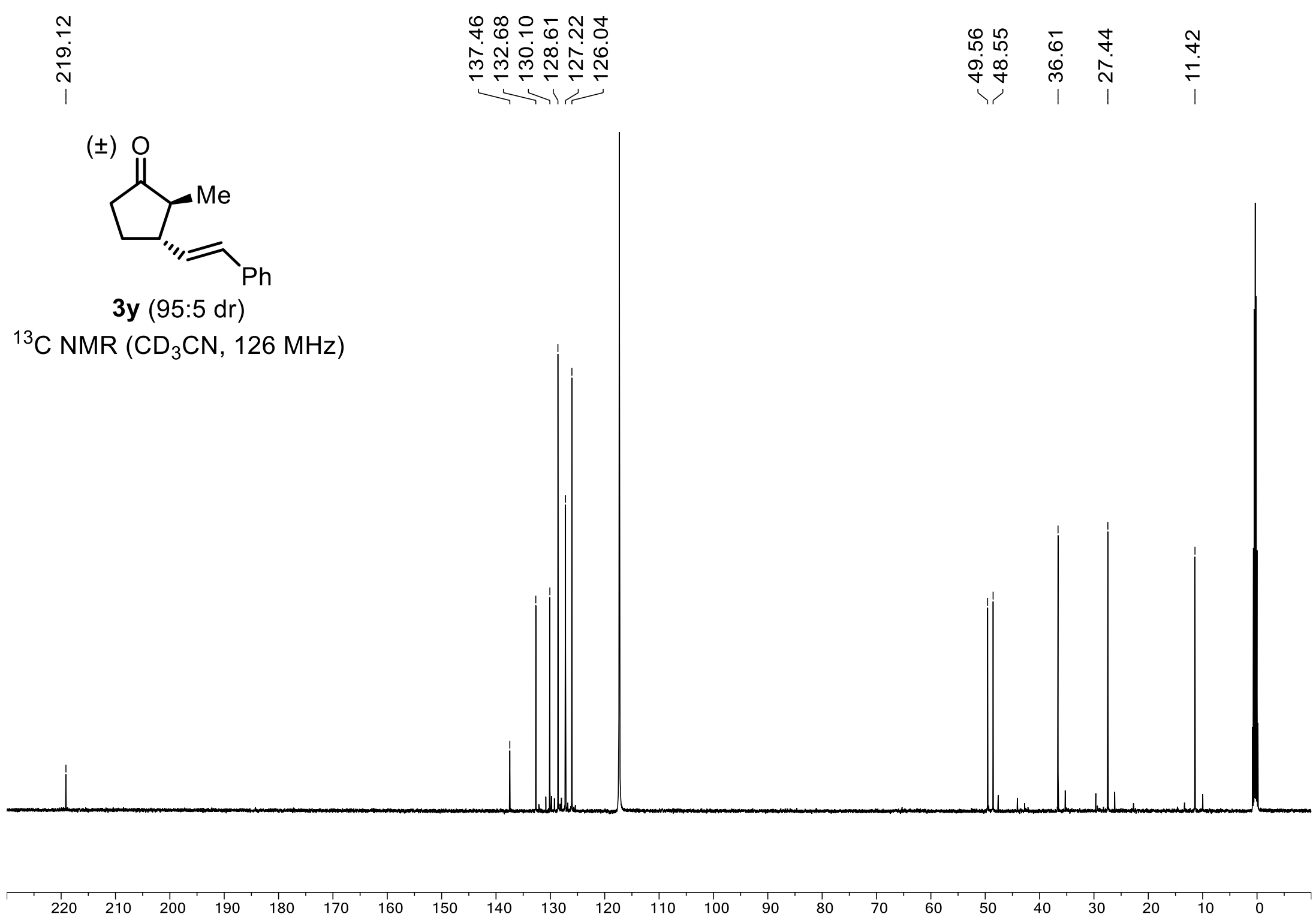


**3y** (95:5 dr)

$^{13}C$ NMR ($CD_3CN$, 126 MHz)

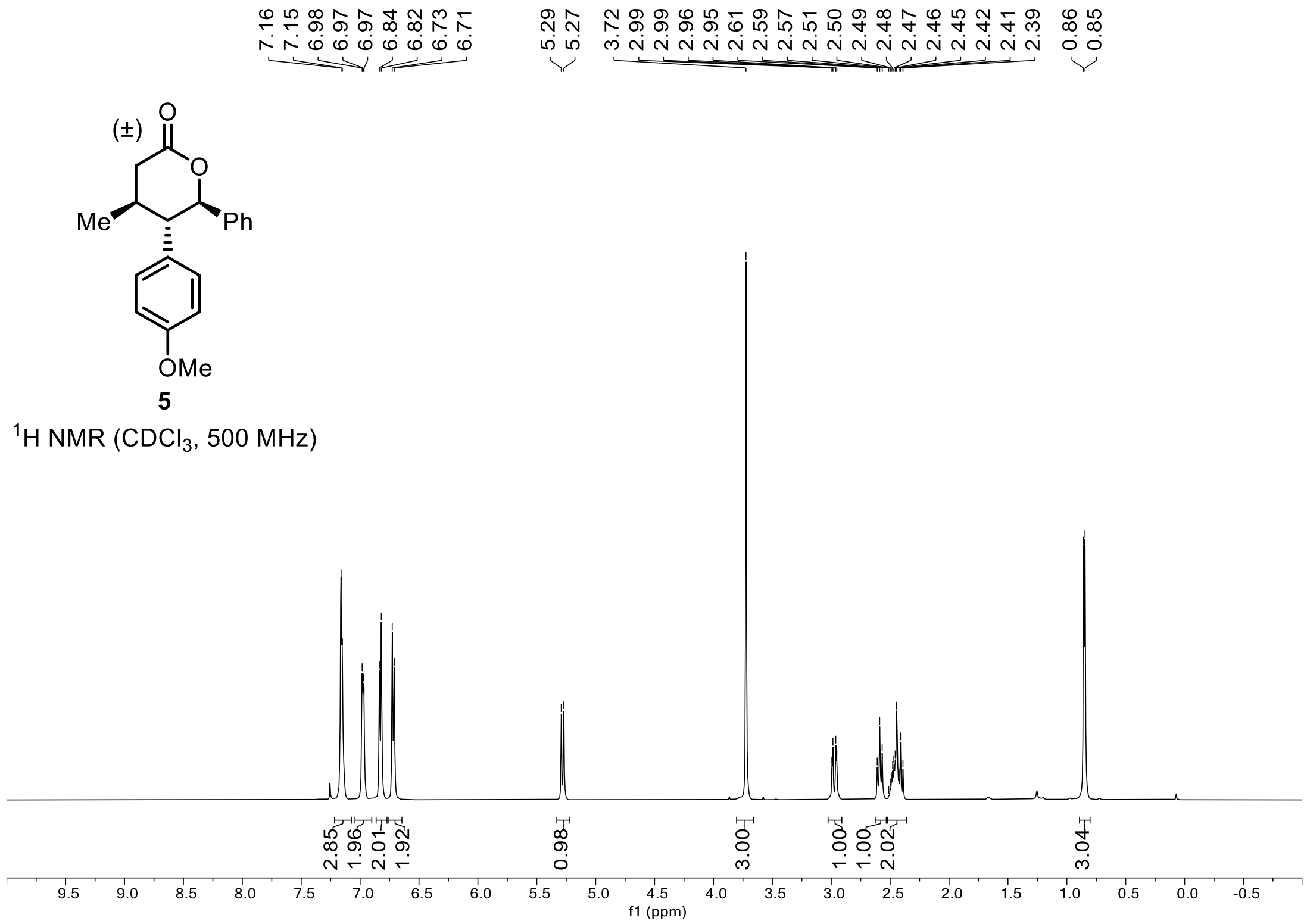


**5**

$^{1}H$ NMR ($CDCl_3$, 500 MHz)

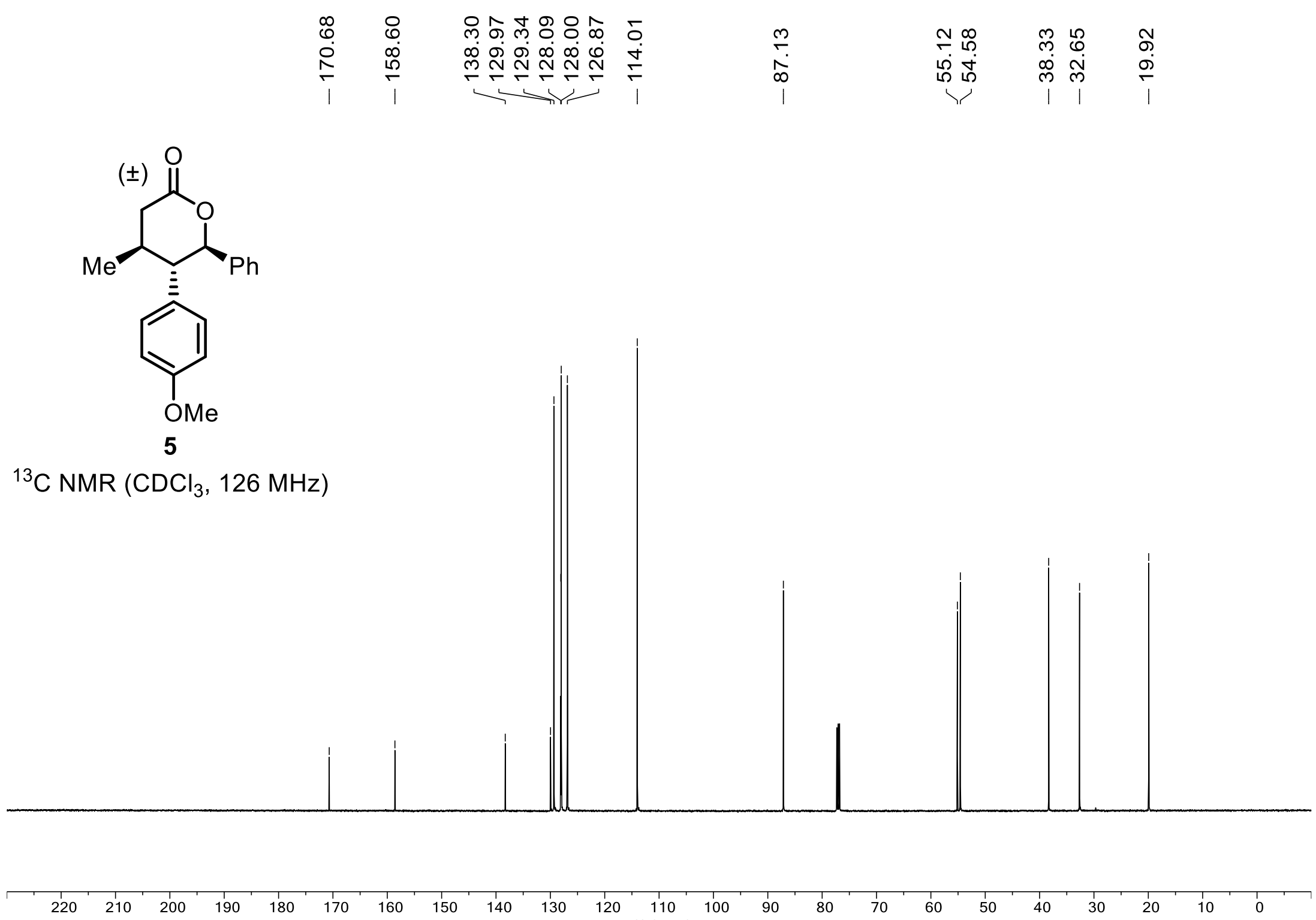


**5**

$^{13}C$ NMR ($CDCl_3$, 126 MHz)

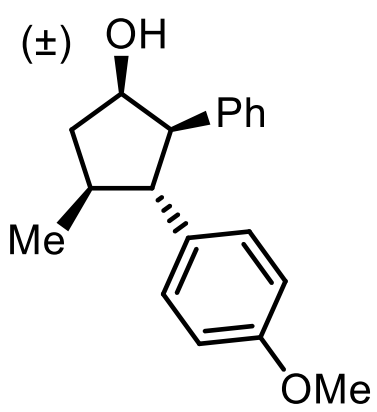

**6**

$^1$H NMR ($CDCl_3$, 500 MHz)

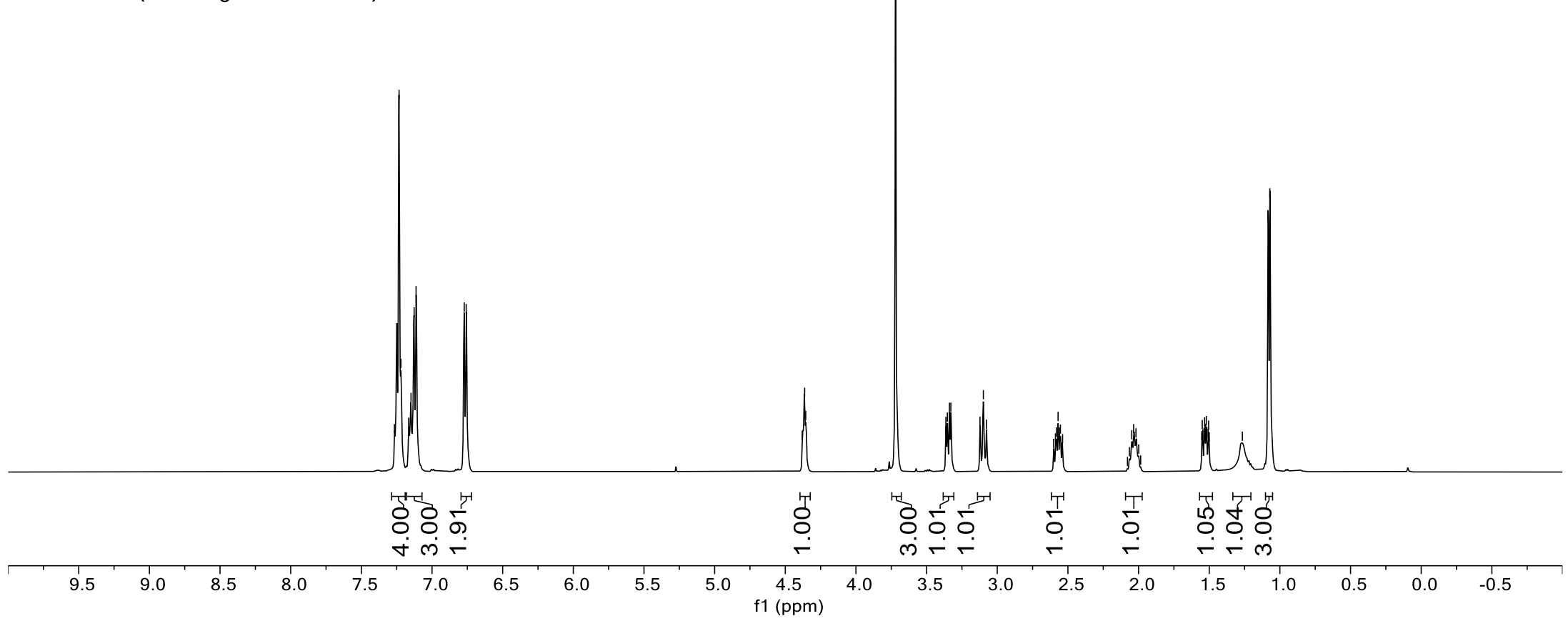


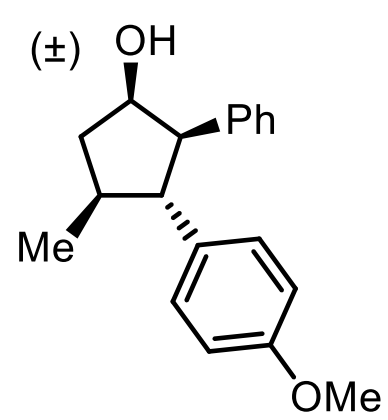

**6**

$^{13}$C NMR ($CDCl_3$, 126 MHz)

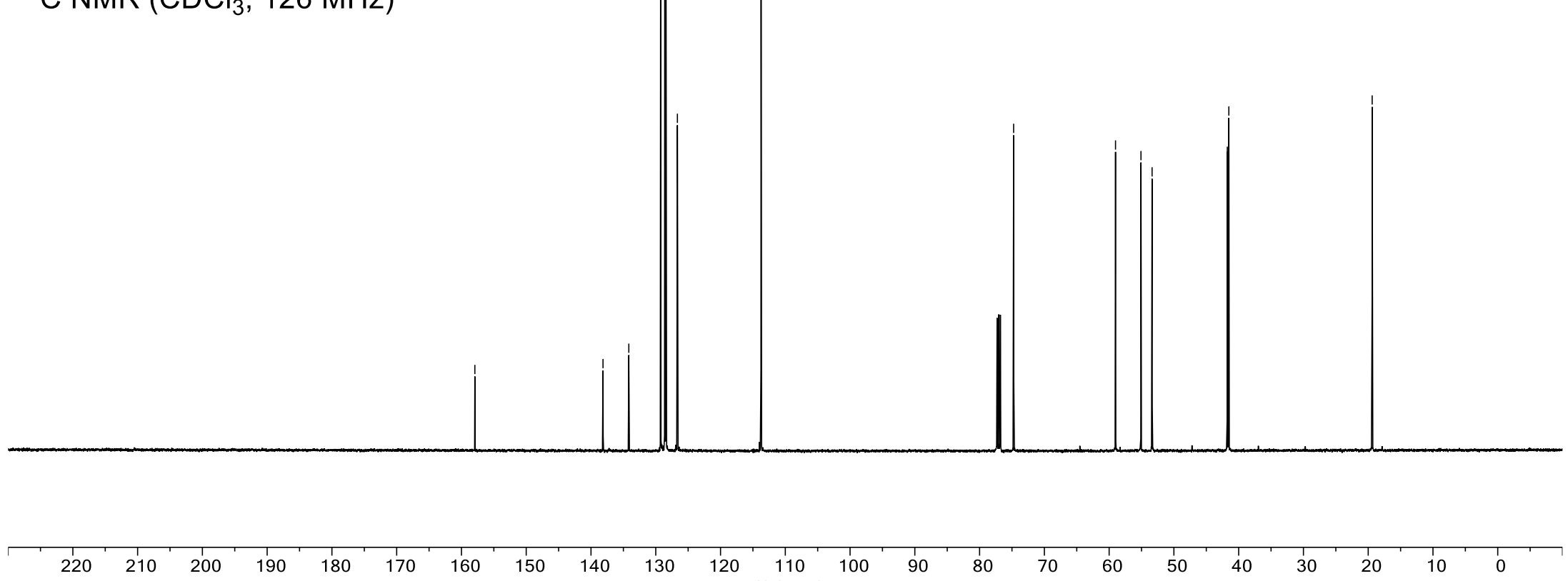

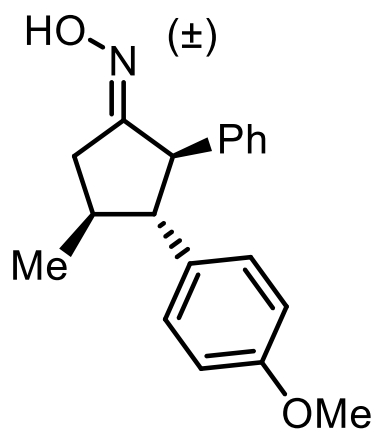


**7**

$^{1}H$ NMR ($CDCl_3$, 500 MHz)

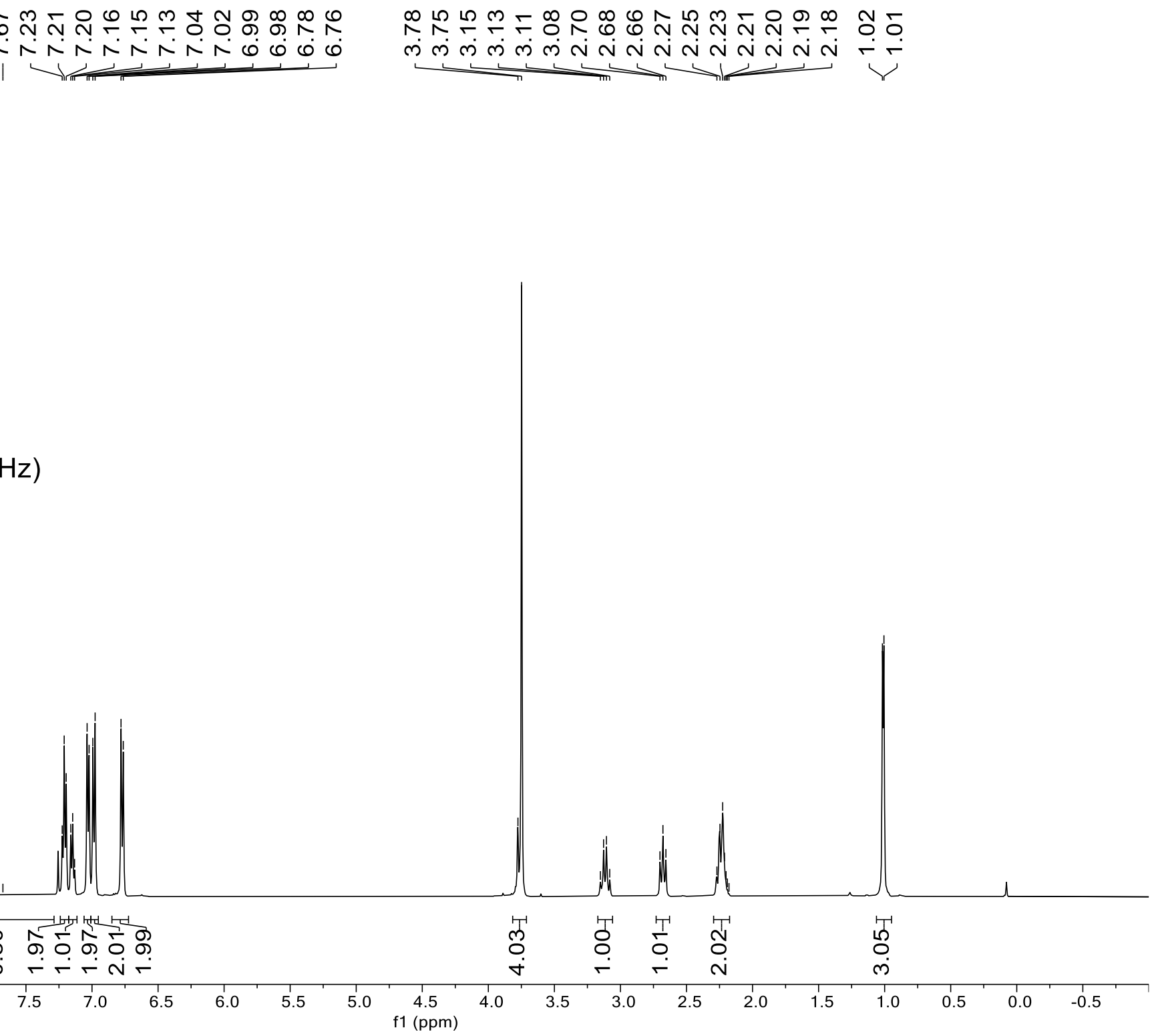


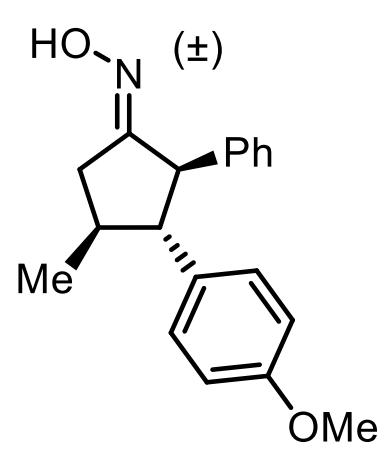


**7**

$^{13}C$ NMR ($CDCl_3$, 126 MHz)

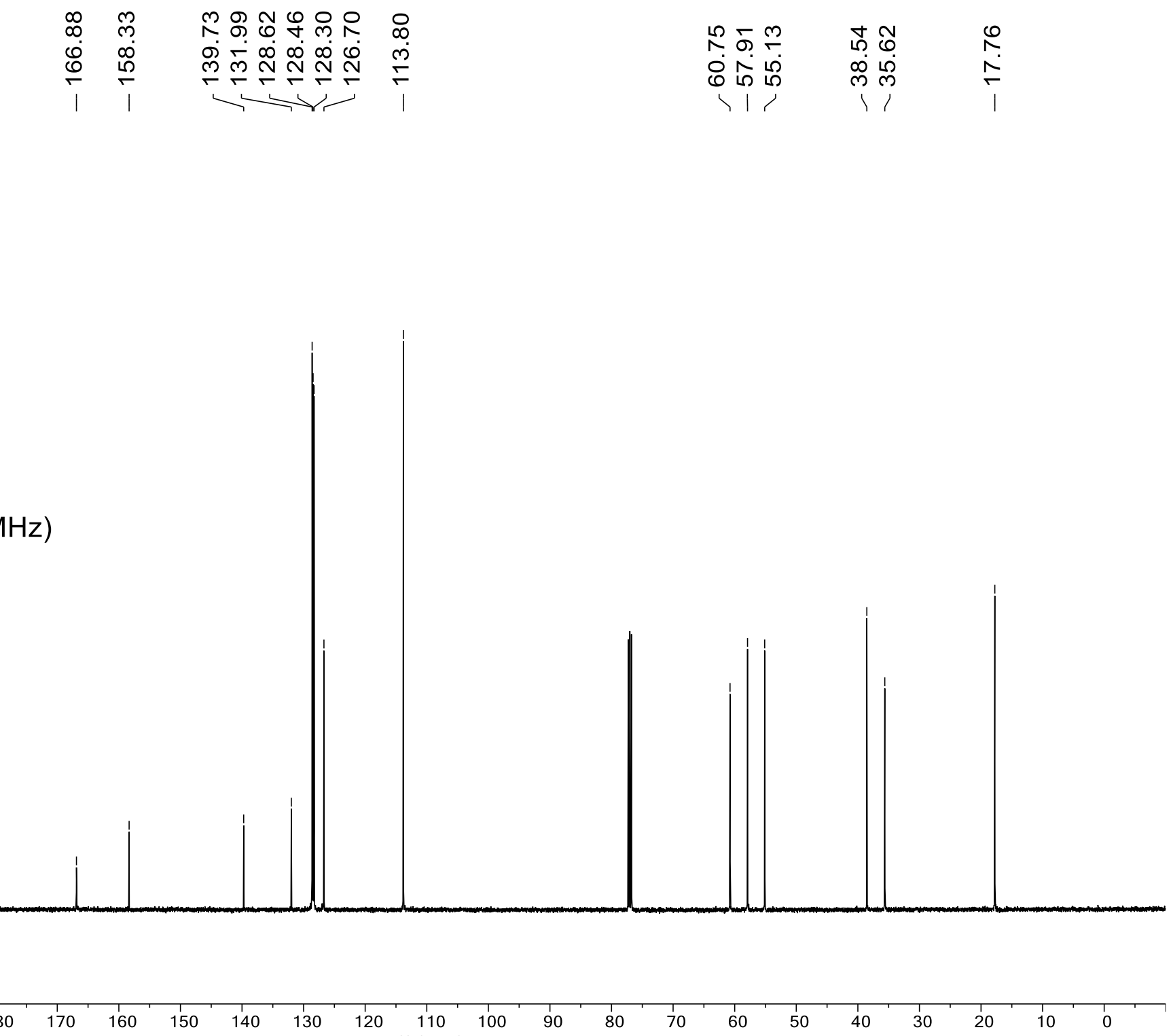

## 9. References


(1) Talero, A. G.; Martins, B. S.; Burtoloso, A. C. B. Coupling of Sulfoxonium Ylides with Arynes: A Direct Synthesis of Pro-Chiral Aryl Ketosulfoxonium Ylides and Its Application in the Preparation of α-Aryl Ketones. *Org. Lett*. **2018**, *20* (22), 7206–7211.

(2) Clare, D.; Dobson, B. C.; Inglesby, P. A.; Aïssa, C. Chemospecific Cyclizations of α-Carbonyl Sulfoxonium Ylides on Aryls and Heteroaryls. *Angew. Chem. Int. Ed.* **2019**, *58* (45), 16198–16202.

(3) Phelps, A. M.; Chan, V. S.; Napolitano, J. G.; Krabbe, S. W.; Schomaker, J. M.; Shekhar, S. Ligand-Controlled Synthesis of Azoles via Ir-Catalyzed Reactions of Sulfoxonium Ylides with 2-Amino Heterocycles. *J. Org. Chem.* **2016**, *81* (10), 4158–4169.

(4) Barday, M.; Janot, C.; Halcovitch, N. R.; Muir, J.; Aïssa, C. Cross-Coupling of a-Carbonyl Sulfoxonium Ylides with C–H Bonds. *Angew. Chem. Int. Ed*. **2017**, *56*, 13117–13121.

(5) Liu, L.; Lin, J.; Pang, M.; Jin, H.; Yu, X.; Wang, S. Photo-Thermo-Mechanochemical Approach to Synthesize Quinolines via Addition/Cyclization of Sulfoxonium Ylides with 2-Vinylanilines Catalyzed by Iron(II) Phthalocyanine. *Org. Lett*. **2022**, *24* (5), 1146–1151.

(6) Echemendía, R.; To, A. J.; Murphy, G. K.; Burtoloso, A. C. B. Copper-Catalyzed α-Arylation of Sulfoxonium Ylides Using Hypervalent Iodine Compounds. *Adv. Synth. Catal.* **2023**, *366* (3), 396–401.

(7) Bissonnier, T.; Brunard, E.; Andresini, M.; Poyer, S.; Saget, T.; Dauban, P. Synthesis of 1,*n*-Diamines via Selective Catalytic C–H Diamination. *J. Am. Chem. Soc*. **2025**, *147* (28), 24684–24690.

(8) Armarego, W. L. F.; Chai, C. L. L. Chapter 4 - Purification of Organic Chemicals. In *Purification of Laboratory Chemicals (Sixth Edition)*, Armarego, W. L. F.; Chai, C. L. L., Eds. Butterworth-Heinemann: Oxford, 2009, pp 88-444.

(9) Angle, S. R.; Choi, I.; Tham, F. S. Stereoselective Synthesis of 3-Alkyl-2-Aryltetrahydrofuran-4-Ols: Total Synthesis of (±)-Paulownin. *J. Org. Chem*. **2008**, *73* (16), 6268–6278.

(10) Mackay, W. D.; Fistikci, M.; Carris, R. M.; Johnson, J. S. Lewis Acid Catalyzed (3 + 2)-Annulations of Donor–Acceptor Cyclopropanes and Ynamides. *Org. Lett.* **2014**, *16* (6), 1626–1629.

(11) Gadwood, R. C.; Mallick, I. M.; DeWinter, A. J. Ring Expansion of Cyclobutanones to Cyclopentanones via Alpha-Lithioalkyl Aryl Sulfoxides and Selenoxides. *J. Org. Chem.* **1987**, *52* (5), 774–782.

(12) Janecký, L.; Markos, A.; Klepetářová, B.; Beier, P. Lewis-Acid-Mediated Intramolecular Cyclization of 4-Aryl-5-Allyl-1,2,3-Triazoles to Substituted Cyclopentene Derivatives. *J. Org. Chem.* **2022**, *88* (2), 1155–1167.

(13) Hayashi, M.; Shibuya, M.; Iwabuchi, Y. Oxidative Conversion of Silyl Enol Ethers to α,β-Unsaturated Ketones Employing Oxoammonium Salts. Org. Lett. 2011, 14 (1), 154–157.

(14) Xie, J.; Xu, P.; Zhu, Y.; Wang, J.; Lee, W.-C. C.; Zhang, X. P. New Catalytic Radical Process Involving 1,4-Hydrogen Atom Abstraction: Asymmetric Construction of Cyclobutanones. *J. Am. Chem. Soc*. **2021**, *143* (30), 11670–11678.

(15) Jacobsen, C. B.; Jensen, K. L.; Udmark, J.; Jørgensen, K. A. Organocatalytic Iminium Ion/Carbene Reaction Cascade for the Formation of Optically Active 2,4-Disubstituted Cyclopentenones. *Org. Lett*. **2011**, *13* (18), 4790–4793.

(16) Pan, J.-L.; Chen, T.; Zhang, Z.-Q.; Li, Y.-F.; Zhang, X.-M.; Zhang, F.-M. A Cu-Mediated One-Pot Michael Addition/α-Arylation Strategy Using a Diaryliodonium Salt: A Direct and Efficient Approach to α-Aryl-β-Substituted Cyclic Ketone Scaffolds. *Chem. Commun.* **2016**, *52* (11), 2382–2385.
